# Collaborative prediction of web service quality based on user preferences and services

**DOI:** 10.1371/journal.pone.0242089

**Published:** 2020-12-07

**Authors:** Yang Song

**Affiliations:** State Key Laboratory of Networking and Switching Technology, Beijing University of Posts and Telecommunications, Beijing, China; Victoria University, AUSTRALIA

## Abstract

The prediction of web service quality plays an important role in improving user services; it has been one of the most popular topics in the field of Internet services. In traditional collaborative filtering methods, differences in the personalization and preferences of different users have been ignored. In this paper, we propose a prediction method for web service quality based on different types of quality of service (QoS) attributes. Different extraction rules are applied to extract the user preference matrices from the original web data, and the negative value filtering-based top-K method is used to merge the optimization results into the collaborative prediction method. Thus, the individualized differences are fully exploited, and the problem of inconsistent QoS values is resolved. The experimental results demonstrate the validity of the proposed method. Compared with other methods, the proposed method performs better, and the results are closer to the real values.

## 1. Introduction

A web service is a low-coupling and reusable network software that is independent of programming languages and operating platforms. By displaying an application interface to the outside world for web invocation, developers can use web services without knowing the details of the software implementation. Starting from the concept of *software as a service*, the emergence of web services has also led to the innovation of the software application model, which has shifted from the traditional software development mode to the use of Web services to achieve maximum software integration and demonstrating great development potential. The emergence of web services, and their gradual innovation, has greatly steered the development of distributed computing in an efficient and accurate direction [1]. At the same time, the increasing popularity of service-oriented computing (SOC) [2] has brought new vitality to web services of different functions and the seamless connection between services and commercial software. Thus, web services have gradually become a popular research topic.

Developers can arbitrarily publish web services with various features. When users consider their own needs, they are faced with many service options, which may lead to selection difficulties. Thus, the accurate prediction and selection of web services are particularly important. As a performance description method, predicting the value of the quality of service (QoS) is the main method to select web services. Specifically, QoS generally uses the idea of collaborative filtering to determine the relationship between different users and their preferences. This idea was first proposed by Goldberg et al. [3] and has been applied in some e-commerce platforms such as Amazon [4], Time Network, and so on. Inspired by this idea, collaborative filtering technology has gradually entered the field of web service selection. Shao et al. [5] proposed a method for collaborative prediction utilizing the similarities between different users. This method first finds neighbors similar to the target users and then performs collaborative prediction based on the invocation records of the neighbors. Subsequently, Vadivelou et al. [6] and Liu et al. [7] also adopted the collaborative filtering method. Existing collaborative filtering methods can be grouped into memory-based and model-based methods [8–10]. The memory-based methods aim at utilizing similar users (or similar web services) for a target user (or a target web service) to predict unknown QoS values [11–13]. Although memory-based methods are straightforward and easy to implement, they usually suffer from the data sparsity problem.

Meanwhile, in recent years, many researchers have also worked on this topic, and context information, such as geographical, social, and trust information [14,15], have been introduced to existing methods for finding more similar neighbors to improve the QoS prediction accuracy of web services. Model-based methods are focused on building global prediction models based on existing QoS observations to predict unknown QoS values [16]. Internet of Things (IoT) predication uses a latent feature learning method to extract the feature vectors of users and services, which are then used to calculate the similarities between them [17,18]. Zhang [19] proposed model-based methods aimed at building global prediction models based on existing QoS observations to predict unknown QoS values. Li et al. [20] introduced a weighting factor to further improve the prediction of Web service performance. The data-aware latent factor (DALF) method clusters the QoS data using a density peak-based clustering algorithm, and uses the model to predict the QoS of a web service. The clustering discovers the neighborhood of the QoS data to improve prediction [21]. Trust-aware collaborative filtering (CF) approaches integrate task similarity into the k-means clustering algorithm to predict the QoS of similar users [22]. The model proposed in [23] used the multiplicative principle and alternative direction method for training to model the features of QoS prediction. Chen proposed a method for predicting QoS values using a similarity between the service and location of the service provider [24]. For avoiding the prediction errors caused by directly using the QoS data, existing method didn’t consider the individual differences among various users and the problem of inconsistent value ranges of QoS attributes.

The optimization methods mentioned above improve prediction accuracy to a certain extent. However, there are also some limitations, such as a lack of comprehensive utilization and detailed analysis, ignoring the differences in the acceptable ranges of different users for web services. Therefore, in this paper, we associate user preferences with web QoS values and propose a collaborative prediction method to improve further prediction accuracy. Our approach consists of the following key steps: 1) data preparation, 2) similarity calculation, and 3) prediction making. Data preparation for collecting QoS data is regarded as a special normalization process, and different extraction rules are applied to different types of QoS attributes. Similarity calculation is used to calculate the preference similarities between the users (services), and to sift out similar neighbors by using negative value filtering based on the top-k algorithm, thereby obtaining the set of nearest neighbors. Predictions are used to calculate the hybrid collaborative prediction results based on users and services, and reduction calculation is performed on the acquired prediction values to obtain the results based on original data.

The rest of this paper is organized as follows. Section 2 presents the problem description. Section 3 describes the preference data extraction rules. Section 4 introduces the collaborative prediction of web services based on user preferences. Section 5 presents the experiment and discussion.

## 2. Problem description

Conventional CF methods [5–7,25] have mostly focused on the algorithm itself while ignoring the individual differences and preferences of the users [26–28]. Here, individual preferences correspond to the acceptable ranges of QoS data for different users, which may also be referred to as the preference range [29]. For instance, two users may have experienced the same response time after invoking the same service. One user was very satisfied with the response time, while the other considered it as a timeout; this is because the two users have different preferences. Different users may have different QoS experiences due to network conditions and other factors. In general, the shorter the response time, the better the user satisfaction. For some bidding online shopping web services, if respond time is 3s, for the user A, who can endure the respond time for browsing. But for the user B, who is in the process of bidding for some stuff, the 3s respond time will miss some bidding opportunity, cause the deal failure.

Thus, the upper limit of the preference range is defined as the lowest response time record, and the lower limit of the preference range is defined as the highest response time record. Similarly, when a user invokes the service, better reliability leads to better the user satisfaction. Hence, the upper and lower limits of the preference range are defined as the highest and lowest reliability records, respectively. Users often prefer to have services with a shorter response time, lower prices, and greater reliability. However, different users may have different application backgrounds, geographical locations, and network conditions; their preference ranges in terms of QoS attributes may vary accordingly.

For example, there are three users under different network conditions, the response time records after accessing four different services are taken as an example, as shown in Table 1. Where each entry in this matrix represents the value of a certain response time of a Web service (e.g., *s*_1_ to *s*_4_) observed by a user (e.g., *u*_1_ to *u*_3_). We define the response time as the time duration between a service user sending a request and receiving a response. The range of response time is 1–0.5s. Due to the project involving some critical applications, the service should achieve the following QoS requirements: the response time should be shorter than 0.2 s. For example, regarding the response time for user u3, *s*_1_ was accessed because it has a faster response time than *s*_4_.

**Table 1:** Response time.

	*s*_1_	*s*_2_	*s*_3_	*s*_4_
*u*_1_	0.1	0.2	0.1	0.2
*u*_2_	0.1	0.2	*null*	0.2
*u*_3_	0.1	0.2	0.1	0.4

In this case, the invocation information of service *s*_3_ is missing for user *u*_2_. Therefore, we treat user *u*_2_ as the target user, and service *s*_3_ as the target service, to predict *u*_2_’s QoS value for response time with regard to service *s*_3_. To calculate the similarity, the conventional CF algorithms are applied to find the users who have visited service *s*_3_ and have invocation records in common with user *u*_2_. As mentioned earlier, the most commonly used similarity calculation method is the Pearson correlation coefficient. Here, the Pearson correlation coefficient was used to calculate the similarity between the users *u*_2_ and *u*_1_; the result is 1. The similarity between the users *u*_2_ and *u*_3_ is calculated in the same way, and the result is also 1. The reason for the identical results that the response time of the three users invoking all the services except the target service was exactly the same. Hence, based on the QoS values of the three services, user *u*_2_ has the same similarity as users *u*_1_ and *u*_3_. Therefore, the predicted QoS value for user *u*_2_ invoking service *s*_3_ is 0.1.

However, users and servers are scattered across the world and interact through the Internet. The uncertainty of multiple factors may cause different users to obtain different QoS feedback data when invoking the same service, and the same user may experience different QoS performance levels when invoking different services. This results in different preference ranges of QoS values for different users. For example, assume the preference ranges of response time for users *u*_1_, *u*_2_, and *u*_3_ are [0.1,1], [0.08,1.5], and [0.1,3], respectively (in seconds). As described above, if the original QoS data is directly used for prediction and the individualized differences of different users are ignored, the results of the similarity calculations will be inaccurate. The QoS preference range should be incorporated into the calculation, and different preference ranges should make different contributions to the prediction. Therefore, this paper proposes a collaborative preference prediction method (PFPre) that links the user preference ranges with QoS values to improve prediction accuracy.

### 3. Preference data extraction rules

Due to the different QoS attribute have the different data type or ratio type, in order to make data in different range be fair on making prediction, we consider QoS attribute into cost-based attributes and benefit-based attributes. Thus, we propose preference data extraction rules that are consistent with the standard collaborative filtering format.

In the following scenario, a user accessing a service will receive *K* QoS feedback values for response time, availability, and throughput. These QoS values can be encapsulated into a *K*-dimensional vector. Next, the access records of *M* users to *N* services can be expressed by the matrix *Matrix*_*MN*_:

$$Matrix_{MN}=(\begin{array}{lllll} l(u_{1},s_{1}), & l(u_{1},s_{2}), & l(u_{1},s_{3}), & \ldots & l(u_{1},s_{N})\\ l(u_{2},s_{1}), & l(u_{1},s_{2}), & l(u_{2},s_{3}), & \ldots & l(u_{2},s_{N})\\ \ldots & \ldots & \ldots & & \\ l(u_{M},s_{1}), & l(u_{M},s_{2}), & l(u_{M},s_{3}), & \ldots & l(u_{M},s_{N}) \end{array})$$ (1)

where *l*(*u*_*m*_, *s*_*n*_) represents a *K-*dimensional vector containing *K* QoS values perceived by the user *u*_*m*_ when accessing the service *s*_*n*_.

Each entry in *Matrix*_*MN*_ represents a vector of QoS values (e.g., response time, failure rate, etc.) that is observed by the service user *u* on the web service item *i*. If the user *u* has not previously invoked the web service item *i*, then *l*(*u*_*m*_, *s*_*n*_) = null. For each QoS attribute in *l*(*u*_*m*_, *s*_*n*_) has a corresponding two-dimensional matrix *M* × *N*. The response time matrix *Rtmatrix* can be written as:

$$Rtmatrix=(\begin{array}{lllll} r(u_{1},s_{1}), & r(u_{1},s_{2}), & r(u_{1},s_{3}), & \ldots & r(u_{1},s_{N})\\ r(u_{2},s_{1}), & r(u_{1},s_{2}), & r(u_{2},s_{3}), & \ldots & r(u_{2},s_{N})\\ \ldots & \ldots & \ldots & & \\ r(u_{M},s_{1}), & r(u_{M},s_{2}), & r(u_{M},s_{3}), & \ldots & r(u_{M},s_{N}) \end{array})$$ (2)

where *r*(*u*_*m*_, *s*_*n*_) represents the specific response time of the user *u*_*m*_ invoking service *s*_*n*_. If there is no invocation record, the QoS data has been lost, and this is expressed as *r*(*u*_*m*_, *s*_*n*_) *= null*. As important indicators when measuring the quality of service, different QoS attributes would have different impacts on service quality. For example, cost-based attributes such as service price and response time should be as low as possible; on the other hand, benefit-based attributes such as service availability and reliability should be as high as possible. The preferences range must be normalized so that for each QoS attribute the upper limit of the preference range indicates the highest user satisfaction, and the lower limit of the preference range indicates the lowest user satisfaction. Thus, the cost attributes and the benefit attributes should be considered separately. Therefore, based on the original QoS matrices, different extraction rules are applied to different types of attributes. The preference matrix formed from the extraction of cost-based attributes is defined as *PFMatrix*^*α*^, and the preference matrix formed from the extraction of benefit-based attributes is defined as *PFMatrix*^*β*^.

For cost-based attributes, taking the response time as an example, the extraction rules of the data *r*^*α*^(*u*_*i*_, *s*_*j*_) in the preference matrix *PFMatrix*^*α*^ can be expressed as:

ra(ui,sj)={1,r(ui,sj)=Min(ui)Max(ui)-r(ui,sj)Max(ui)-Min(ui)0,r(ui,sj)=Max(ui),Min(ui)<r(ui,sj)<Max(ui) (3)

For benefit-based attributes, taking the reliability as an example, the extraction rules of the data *r*^*β*^(*u*_*i*_, *s*_*j*_) in the preference matrix *PFMatrix*^*β*^ can be expressed as

rβ(ui,sj)={1,r(ui,sj)=Max(ui)Max(ui)-r(ui,sj)Max(ui)-Min(ui)0,r(ui,sj)=Max(ui),Min(ui)<r(ui,sj)<Max(ui) (4)

where *Min*(*ui*) and *Max*(*ui*) refer to the minimum and maximum QoS feedback values perceived by *u*_*i*_ for the services that the user has visited, respectively.

The values in the resulting preference matrix fall into the range of [0,1] regardless of the attribute types. The larger the value, the more satisfied the user. Therefore, the individual user’s preference in terms of QoS data is fully considered. In addition, this can also be regarded as a special normalization process, which avoids the similarity calculation error caused by the inconsistency of the preference ranges.

## 4. Collaborative prediction of web service based on user preferences

### 4.1 Similarity calculation based on user preferences

After the user preference matrix is extracted, it is used to calculate the user’s preference-based similarity. Similarity calculation is a core component of the CF algorithm. First, similarity calculation directly relates to the sifting of similar neighbors, which is fundamental for finding high-quality neighbors. Second, the weighted sum of similarities of similar neighbors is usually used in the prediction phase. Hence, the similarity calculation also determines the amount of weight that is given to similar neighbors during the prediction process.

In the field of collaborative prediction, the most commonly used similarity calculation methods are the Pearson correlation coefficient [30], Tanimoto coefficient [31], and Euclidean distance [32]. Euclidean distance is the simplest and most straightforward similarity algorithm and can reflect the absolute difference of individual numerical characteristics. The value ranges in the resulting preference matrices were standardized; thus, the similarity calculation can be performed according to the values of the preference data. Here, the Euclidean distance is chosen to perform the similarity calculation, and the preference similarity between users *u*_*i*_ and *u*_*j*_ can be calculated as

$${Sim}_{Euc}(u_{i},u_{j})\;=\;\frac{1}{1+\sqrt{\sum_{s\in S_{u_{ij}}}{\left(r^{a}(u_{i},s)-r^{a}(u_{j},s)\right)}^{2}}}$$ (5)

where Suij = Sui*Su*_*ij*_ = *Su*_*i*_∩*Su*_*j*_, *Su*_*i*_, and *Su*_*j*_ represent the service sets accessed by *u*_*i*_ and *u*_*j*_ and *Su*_*i j*_ represents the overlapping portion of the services accessed by *u*_*i*_ and *u*_*j*_, which is the intersection of two users’ historical access records. Moreover, *r*^*α*^(*u*_*i*_, *s*) and *r*^*α*^(*u*_*j*_, *s*) represent the QoS values in the preference matrix for *u*_*i*_ and *u*_*j*_ after accessing each service *s* in the intersection set *su*_*i*,*j*_, which is the extracted preference data. The similarity of service preferences can be calculated as

$${Sim}_{Euc}(s_{i},s_{j})\;=\;\frac{1}{1+\sqrt{\sum_{s\in U_{s_{ij}}}{\left(r^{a}({u,s}_{i})-r^{a}({u,s}_{j})\right)}^{2}}}$$ (6)

where *Us*_*ij*_ = *Us*_*i*_∩*Us*_*j*_, *Us*_*i*_, and *Us*_*j*_ represent the overlapping sets of the users who have invoked both the services *S*_*i*_ and *S*_*j*_, and *r*^*α*^(*u*, *s*_*i*_) and *r*^*α*^(*u*, *s*_*j*_) represent the QoS values in the preference matrix for user *u* after invoking the services *S*_*i*_ and *S*_*j*_, respectively.

In order to emphasize only the role of the preference matrix in the proposed algorithm, while ignoring any interference from other steps of the algorithm, this chapter uses the traditional top-*K* algorithm to select similar neighbors. The Euclidean distance-based similarities are ranked from high to low, and the top *K* members are selected as neighbors that will directly participate in the prediction. However, there is a loophole in this process. If the minimum similarities of the top *K* members are less than 0, then the weakly correlated users would participate in the prediction, thereby increasing the prediction error. Therefore, the proposed method adds a negative-value filtering policy to the neighbor selection process. Users with a similarity of less than 0 will be removed from the top *K* members to obtain the final set comprising the nearest neighbors.

### 4.2 Hybrid QoS collaborative prediction based on users and services

In this chapter, in order to predict the missing values as accurately as possible, we present a hybrid approach that systematically combines user-based and service-based methods to predict the QoS values. The specific steps are as follows: First, the final set of nearest neighbors is sifted. Following this, the prediction of the missing QoS attributes is performed based on the similarities between all users (services) and the target users (services) in this set.

Taking a cost-based attribute (e.g., response time) as an example, the collaborative prediction based on user preferences [33] can be written as

$$P_{U}^{a}(u_{i},s_{j})\;=\;{\overset{-}{r}}^{a}(u_{i})\;=\;\frac{\sum_{s\in {Sim(u}_{i})}\left(u_{i},u)(r^{a}(u,s_{j})-{\overset{-}{r}}^{a}(u)\right)}{\sum_{s\in {Sim(u}_{i})}Sim(u_{i},u)}$$ (7)

where ${\bar{r}}^{\alpha }(u_{i})$ and ${\bar{r}}^{\alpha }(u)$ are the mean values of the service feedback from users *u*_*i*_ and *u* in the preference matrix, respectively.

Similarly, the collaborative prediction based on the services can be written as

$$P_{S}^{a}(u_{i},s_{j})\;=\;{\overset{-}{r}}^{a}(s_{j})\;=\;\frac{\sum_{s\in {Sim(s}_{j})}{Sim}_{Euc}(s_{j},s)(r^{a}(u_{i},s)-{\overset{-}{r}}^{a}(s))}{\sum_{u\in {Sim(s}_{i})}Sim(s_{i},s)}$$ (8)

where ${\bar{r}}^{\alpha }(s_{j})$ and ${\bar{r}}^{\alpha }(s)$ are the mean values of the user feedback for using the services *s*_*j*_ and *s* in the preference matrix, respectively. *Sim*(*s*_*j*_) is the set of similar neighbors for service *s*_*j*_. The data sparsity problem during the collaborative sifting process can be effectively alleviated by incorporating the user-based and service-based CF algorithms to improve the accuracy of the QoS predictions. Therefore, this section also adopts a hybrid QoS collaborative prediction model based on users and services.

The final prediction based on user preferences can be written as

$$P^{a}(r(u_{i},s_{j}))\;=\;w_{u}\times P_{U}^{a}(u_{i},s_{j})+w_{s}\times P_{S}^{a}(u_{i},s_{j})$$ (9)

where *P*^*α*^_*U*_(*u*_*i*_, *s*_*j*_) is the prediction result of preference-based collaborative filtering and *P*^*α*^_*S*_(*u*_*i*_, *s*_*j*_) is the prediction result of service-based collaborative filtering; benefit-based attributes such as reliability can also be calculated in this manner.

### 4.3 Reduction calculation of QoS prediction values

Through the above discussion, we begin from the user preference information extracted from the original user-service QoS attribute matrix. The data are normalized in the range of [0,1] and the collaborative prediction is then performed. By considering the individual differences among various users that are inevitable in real-world scenarios, we avoid the problem of users with different preference ranges being treated equally in the prediction. Thus, prediction errors owing to the inconsistent fluctuation ranges of the QoS values have been lowered.

It is worth mentioning that both the user-based and service-based prediction values given by (6) and (7) are the results calculated with the user preference matrix. When analyzing the accuracy of the algorithm, they cannot be directly compared with the real values, but should be reduced to the predicted values under the original matrix and then compared with the real values.

To normalize the preference ranges, the original matrix data are extracted using Eqs (3) and (4), and mapped into a user preference matrix. Hence, the reduction calculation can be seen as the process of using the known image (i.e., the predicted values under the preference matrix) to find the inverse image (i.e., the predicted values under the original matrix) according to the corresponding map (preference extraction rules). Thus, for cost-based attributes such as response time, the reduction rules of the QoS prediction values calculated by the CF algorithm can be written as

$${Pre}^{a}(r(u_{i},s_{j}))\;=\;Max(u_{i})-P^{a}(r(u_{i},s_{j}))\times (Max(u_{i})-Min(u_{i}))$$ (10)

Similarly, for benefit-based attributes, the reduction rules of the QoS prediction values calculated by the CF algorithm can be expressed as

$${Pre}^{\beta }(r(u_{i},s_{j}))\;=\;Min(u_{i})-P^{\beta }(r(u_{i},s_{j}))\times (Max(u_{i})-Min(u_{i}))$$ (11)

where *P*^*α*^(*r*(*u*_*i*_, *s*_*j*_)) and *P*^*β*^(*r*(*u*_*i*_, *s*_*j*_)) are the predicted results of the cost-based attributes and benefit-based attributes, *Pre*^*α*^(*r*(*u*_*i*_, *s*_*j*_)) and *Pre*^*β*^(*r*(*u*_*i*_, *s*_*j*_)) are the prediction values of the cost-based attributes and benefit-based attributes after the reduction calculation, *Min*(*u*_*i*_) is the minimum QoS feedback value of the user *u*_*i*_ accessing the services, and *Max*(*u*_*i*_) is the maximum QoS feedback value of user *u*_*i*_ accessing the services.

Algorithm 1 demonstrates the collaborative prediction algorithm of web services based on user preferences. Algorithm 1 can be divided into four parts: Part 1 contains line 1, which is used to initialize and define the required variables; The response time as the required variables to be initialized. we begin from the user preference information extracted from the original user-service QoS attribute matrix. The data are normalized in the range of [0,1] and the collaborative prediction is then performed. Part 2 contains lines 2 to 4, which are used to extract the user preference matrix, to extract user response time by using the extraction rules. Part 3 contains lines 5 to 18, which are used to calculate the preference similarities between the users (services) and to sift out similar neighbors by using the negative value filtering-based top-K algorithm, thereby obtaining the set of nearest neighbors; Part 4 contains lines 19 to 21, which are used to calculate the hybrid collaborative prediction results based on the users and services, and to perform the reduction calculations on the acquired prediction values to obtain the results based on the original data.

**Algorithm 1. Collaborative prediction algorithm based on user preference range**.

 **Input**: user-service matrix *RTMatrix*_*MN*_, target user *u*_*i*_, target service *s*_*j*_, prediction tuning parameter λ, the number of nearest neighbors *neighbor*_*k*;

 **Output**: QoS prediction values *Pre*(*r*(*u*_*i*_, *s*_*j*_)).

(01) *Sim*[*u*_α_], *Sim*[*s*_*i*_], *N*(*u*_α_), *N*(*s*_*i*_)

 *Pre*^*α*^_*U*_ (*r*(*u*_*i*_, *s*_*j*_))←0;        // store the user-based collaborative prediction results.

 *Pre*^*α*^_*S*_ (*r*(*u*_*i*_, *s*_*j*_))←0;        // store the service-based collaborative prediction results.

 *Pre*^*α*^(*r*(*u*_*i*_, *s*_*j*_))←0.

 // Store the hybrid collaborative prediction results based on users and services

 *Pre*(*r*(*u*_*i*_, *s*_*j*_))←0;

 // store the reduction calculation results of the user preference-based prediction, that is, the final prediction values.

(02) **for each** *r*(*u*_*m*_, *s*_*n*_)∈*RTMatrix*_*MN*_ **do**

(03) *PFMatrix*^*α*^← *preference*(*r*(*u*_*m*_, *s*_*n*_));

  // extract user preference information by using the extraction rules.

(04) **end for**

(05) **for each** *u*_*i*_∈*U***do**.

(06) *sim*(*u*_*a*_, *u*_*i*_) ← *EucSimilarity*(*PFMatrix*^α^, *S*(*u*_*a*_, *u*_*i*_)).

  // Calculate the preference similarities among users based on the preference matrix.

(07) **end for**

(08) *N*(*u*_*a*_)←*Top* −*K*(*sim*(*u*_*a*_), *neighbor_k*)

 // sifting out the set of similar neighbors of users by using the negative-value filtering-based Top-K algorithm.

(09) **for each** (*r*^*α*^(*u*_*i*_, *s*_*j*_)←–1)∈*PFMatrix*^*α*^**do**

(10) *Pre*^*α*^_*U*_ (*r*(*u*_*i*_, *s*_*j*_)) ←*Predict*(*r*^*α*^(*u*_*i*_, *s*_*j*_), *N*(*u*_*a*_), *sim*(*u*_*a*_));

(11) **end for**

(12) **for** each *s*_*i*_∈*S*,

(13) *sim*(*s*_*a*_, *s*_*j*_)) ←*EucSimilarity*(*PFMatrix*^*α*^, *U*(*s*_*a*_, *s*_*i*_));

  // Calculate the preference similarities among services by using the Euclidean method based on the preference matrix.

(14) **end for**

(15) *N*(*s*_*a*_)←*Top* −*K*(*sim*(*s*_*a*_), *neighbor_k*)

 // sifting out the set of similar neighbors of services by using the negative-value filtering-based Top-K algorithm.

(16) **for each** (*r*^*α*^(*u*_*i*_, *s*_*j*_)←–1)∈*PFMatrix*^*α*^**do**,

(17) *Pre*^*α*^_*S*_ (*r*(*u*_*i*_, *s*_*j*_)) ←*Predict*(*r*^*α*^(*u*_*i*_, *s*_*j*_), *N*(*s*_*a*_), *sim*(*s*_*a*_));

(18) **end for**

(19) *Pre*(*r*(*u*_*i*_, *s*_*j*_)) ←*MixPredict*(*Pre*^*α*^_*U*_ (*r*(*u*_*i*_, *s*_*j*_)), *Pre*^*α*^_*S*_ (*r*(*u*_*i*_, *s*_*j*_))).

 //hybrid collaborative prediction based on users and services.

(20) *Pre*(*r*(*u*_*i*_, *s*_*j*_)) ←*ReductionCalculation*(*Pre*^*α*^(*r*(*u*_*i*_, *s*_*j*_)));

 // Calculation of the prediction results

(21) **return** *Pre*(*r*(*u*_*i*_, *s*_*j*_))

 **End**

## 5. Experiments and discussion

### 5.1 Experiment preparation

#### 1) Dataset

The experimental dataset was the QoSDataset2 from the publicly released WS-DREAM [34,35] and the Web service searching engines: xmethods.net. The experimental dataset was the QoSDataset2 from the publicly released WS-DREAM [34,35] and the Web service searching engines: xmethods.net. The dataset includes 5301 Web services. WSDL (Web Service Description Language) provides XML-based descriptions of Web service interfaces. In this paper, through crawling Web service, we obtain 7213 addresses of WSDL files, after analyzing WSDL files, by establishing HTTP connections to the 7213 WSDL addresses obtained, we successfully download 5301 (73.77%) WSDL files. Due to the network is dynamic and unpredictable and the Web service information on the Internet are out-of-date, which make some WSDL files failure. The WSDL download failures status are summarized in Table 2, there are totally 1912 failures. 69.1% of these failures are failures caused by network connection problems, File Not Found failures (12.7%) and Internal Server Error failures (18.2%).

**Table 2:** WSDL file download failure status.

Statistic	Web Service	Present
network connection problems	1321	
File Not Found	243	
Internal Server Error failures	348	
Total	1912	

Then, Using Axis 2 tool, we generate client-side Web service invocation codes to simulate the user sending the request, we record the respond time of the user invoking service, which is defined as the time duration between a service user sending a request and receiving the corresponding response, collect these as the experiment data. As shown in Table 3, totally 128932 Web service invocations are executed by 214 service users in this experiment.

**Table 3:** Statistic of the dataset.

Statistic	Value
Num. of Services Users	214
Num. of Web Service	5301
Num. of Web Service Invocation	128932

We constructed three user-service matrices with different of size = 100 × 100, size = 100 × 150, size = 150 × 100 by randomly extracting a certain number of users and services, where each entry in the matrix is a vector with response time. Response time represents the time duration between the client sending a request and receiving a response. To monitor Web service performance, we randomly select 50 Web services for our experiments to monitor and collect QoS information of the selected Web services.

#### 2) Performance metrics

The mean absolute error (MAE) and normalized mean absolute error (NMAE), which are most commonly used in the rating prediction field, were chosen to evaluate the accuracy of the proposed algorithm.

### 5.2 Comparison of the prediction methods

To evaluate the effectiveness and accuracy of the proposed PFPre method, we compared its performance with the most commonly used prediction methods, which are UPCC [36], IPCC [37], and WSPre [38]. In order to perform prediction, UPCC uses the Pearson correlation coefficient to calculate the similarities between users and find their similar neighbors, while IPCC uses the same method for the services. The WSPre model uses the weighted prediction results of UPCC and IPCC as the final prediction results.

The experimental parameters were set for the PFPre configuration. In this experiment, the density of the dataset started from 10% and ended at 50% with in increments of 10%. In addition, *neighbor*_*k* = 15, and *λ* = 0.3. The settings for the parameters *neighbor*_*k* and λ will be discussed in detail in Sections 5.3 and 5.4. To assess the adaptability of the proposed model, three user-service response time matrices with different sizes and structures of 100 × 100, 100 × 150, and 150 × 100 were constructed by randomly extracting a certain number of users and services. The MAE and NMAE were used to evaluate the accuracy of the algorithms. The experimental results are shown in Tables 4 and 5.

**Table 4:** Comparison of accuracy of PFPre and other prediction methods in terms of MAE (the smaller the MAE value, the higher the prediction accuracy).

datasets	method	MAE				
		d = 10%	d = 20%	d = 30%	d = 40%	d = 50%
100×100	UPCC	0.3460	0.3011	0.2890	0.2919	0.2824
	IPCC	0.3526	0.2934	0.2562	0.2532	0.2392
	WSRec	0.3348	0.2713	0.2601	0.2491	0.2412
	**PFPre**	**0.2752**	**0.2693**	**0.2412**	**0.2129**	**0.1991**
100×150	UPCC	0.4712	0.4001	0.3792	0.3564	0.3613
	IPCC	0.4582	0.3882	0.3512	0.3316	0.3294
	WSRec	0.4392	0.3912	0.3431	0.3367	0.3156
	**PFPre**	**0.3812**	**0.3719**	**0.3250**	**0.3101**	**0.2885**
150×100	UPCC	0.4792	0.3922	0.3752	0.3693	0.3578
	IPCC	0.4362	0.3847	0.3316	0.3361	0.3250
	WSRec	0.4213	0.3521	0.3231	0.3290	0.3109
	**PFPre**	**0.3412**	**0.3092**	**0.2614**	**0.2315**	**0.2187**

**Table 5:** Comparison of accuracy of PFPre and other prediction methods in terms of NMAE (the smaller the NMAE value, the higher the prediction accuracy).

datasets	method	MAE				
		d = 10%	d = 20%	d = 30%	d = 40%	d = 50%
100×100	UPCC	0.6392	0.5721	0.5174	0.5189	0.5023
	IPCC	0.6234	0.5214	0.4542	0.4432	0.4234
	WSRec	0.5921	0.5255	0.4611	0.4467	0.4111
	**PFPre**	**0.5124**	**0.4731**	**0.4021**	**0.3821**	**0.3621**
100×150	UPCC	0.7287	0.6012	0.5698	0.5592	0.5690
	IPCC	0.6853	0.5834	0.5241	0.5116	0.5014
	WSRec	0.6512	0.5623	0.5198	0.5091	0.4921
	**PFPre**	**0.5825**	**0.5201**	**0.4890**	**0.4509**	**0.4392**
150×100	UPCC	0.7214	0.6123	0.5756	0.5765	0.5211
	IPCC	0.6854	0.5609	0.5256	0.5027	0.4933
	WSRec	0.6423	0.5646	0.5022	0.4931	0.4824
	**PFPre**	**0.4921**	**0.4561**	**0.4235**	**0.3986**	**0.3563**

Tables 4 and 5 show that: (1) as the density of the matrix sparsity increases, the MAE values of the four methods decrease, indicating that as the data becomes denser, the prediction accuracy will increase. (2) Compared with other algorithms, the proposed PFPre method exhibits lower MAE and NMAE values with smaller errors under the same conditions, indicating that the algorithm outperforms other traditional algorithms in terms of prediction accuracy.

### 5.3 Parameter tuning for the Top-K algorithm

**Fig 1:**
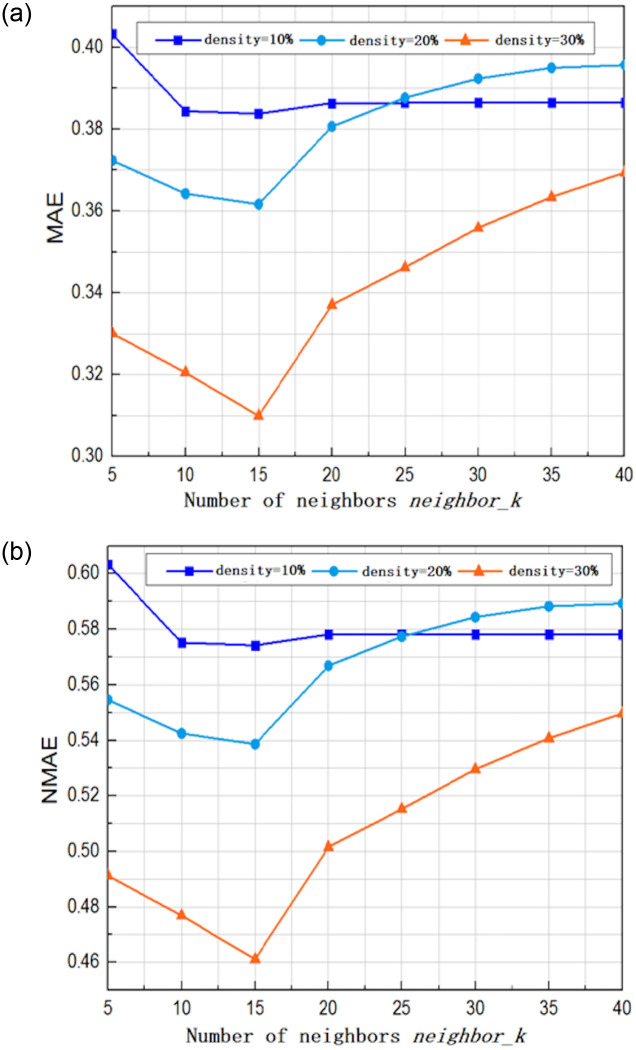
The influence of different values of the *neighbor*_*k* parameter in the top-K algorithm on the prediction accuracy. (a) Influence on the MAE (b) Influence on the NMAE.

When sifting the similar neighbors, the *neighbor*_*k* parameter controls the size of the nearest neighbor set. If *neighbor*_*k* is too small, there will not be a sufficient number of neighbors, resulting in lower prediction accuracy. If *neighbor* _*k* is too large, some neighbors with weak correlations will be placed into the nearest neighbor set [39], resulting in the similar neighbors making a lesser contribution to the prediction. Therefore, it is necessary to find an appropriate value for *neighbor*_*k* to improve the prediction performance. To evaluate the influences that are imposed on the results by changing *neighbor* _*k*, we constructed a 100×150 user-service matrix by randomly extracting a certain number of users and services, where λ = 0.3, the value of *neighbor*_*k* was increased from 5 to 40 in increments of 5, and the data density was within the range of 10% to 30%. The results are shown in Fig 1a and 1b.

The above figures show that when the value of *neighbor* _ *k* was 15, the minimum error was achieved under all the scenarios with different densities, indicating optimal prediction performance. Hence, the value of *neighbor* _ *k* was set to 15 for the rest of the experiments. When the sparsity density was 10%, the prediction results remain basically the same after the value of *neighbor* _ *k* became greater than 25. The reason for this result is that the negative value filtering strategy has been used during the sifting of similar neighbors. Under the scenarios with low density, the number of neighbors to be chosen was relatively small, and neighbors with a similarity less than 0 were excluded. Hence, after the value of *neighbor*_*k* was increased to a certain degree, the variations in the final nearest neighbor set were small, leading to a minute fluctuation in the prediction results.

### 5.4 Parameter tuning for hybrid prediction

The parameter *λ* is responsible for adjusting the proportion of the user-based and service-based predictions in the PFPre training model. We constructed a 100×150 user-service matrix by randomly extracting a certain number of users and services, where *neighbor* _ *k* was set to 15, the value of *λ* was in the range of 0 and 1 with an increment of 0.1, and the predictions were performed under scenarios of various densities (10%, 20%, and 30%).

**Fig 2:**
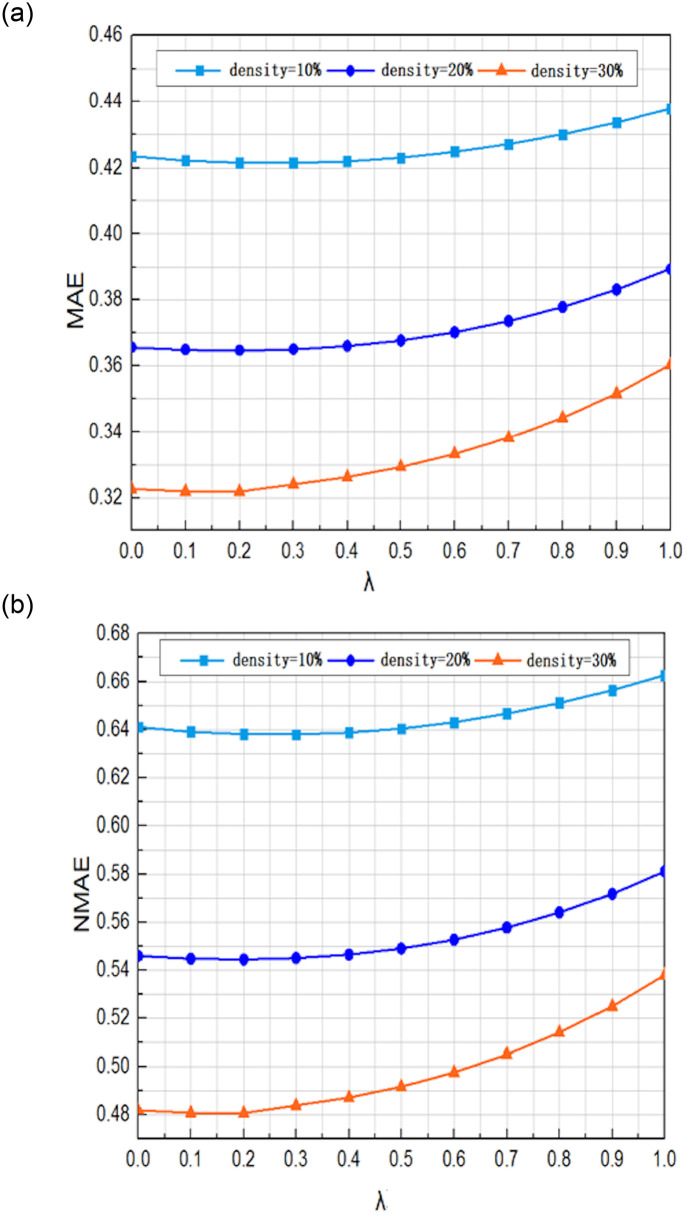
The influence of different values of the parameter λ on prediction accuracy. (a) Influence on the MAE (b) Influence on the NMAE.

It is evident that the most promising prediction results were achieved under different scenarios when the value of *λ* was set to 0.3. The influence of different values of λ on the prediction accuracy is shown in Fig 2.

### 5.5 Comparison of recommendation instances

To further verify the proposed method and its practical significance, this section introduces the experiments performed in specific scenarios. To analyze the advantages and disadvantages of each algorithm, different methods were used to recommend high-performance services for the same user, and the recommendation results were then compared. Consider the QoS attribute of response time as an example, where the density of the dataset was set to 30%. For PFPre, λ = 0.3 and *neighbor*_*k* = 15. For the conventional prediction method WSRec, λ = 0.3. The two algorithms were applied to provide a recommendation to the same user in the dataset based on service performance (in this case, the response time). Users commonly prefer to have high-performance services that are reliable and provide prompt responses. Subsequently, the actual response time values were ranked in ascending order to compare the differences in the recommendation instances obtained from each algorithm. The top 10 web services were chosen for comparison; the results are shown in Table 6.

**Table 6:** Recommendation results (in seconds).

No.	Address	Actual Value	PFPre prediction value (sorted)	WSRec predicted value (sorted)	PFPre error	WSRec error
86	http://forums.genom-e.com/_vti_bin/BusinessDataCatalog.asmx?wsdl	0.038	0.3298	1.5114	0.2918	1.4734
87	http://forums.genom-e.com/_vti_bin/SharepointEmailWS.asmx?wsdl	0.039	0.3366	2.1730	0.2976	2.1340
67	http://www.webxml.com.cn/WebServices/RandomFontsWebService.asmx?wsdl	0.047	0.3408	2.1960	0.2938	2.1490
100	http://www.arikan.at/axis/services/SOAPMonitorService?wsdl	0.087	0.3677	1.4537	0.2807	1.3667
38	http://ws.webxml.com.cn/WebServices/TraditionalSimplifiedWebService.asmx?wsdl	0.121	0.4257	1.5987	0.3047	1.4777
45	https://api.oceandrivers.com/static/resources.json?wsdl	0.121	0.5277	1.5559	0.4067	1.4349
95	http://www.arikan.at/axis/services/CountryService?wsd	0.122	0.4521	1.5524	0.3301	1.4304
62	http://www.stratapay.com.au/ecommservices.asmx?WSDL	0.142	0.6063	1.7462	0.4643	1.6042
58	https://www.netvibes.com/js/UWA/load.js.php?wsdl	0.145	0.6010	1.7015	0.456	1.5565
59	http://www.webxml.com.cn/WebServices/ChinaOpenFundWS.asmx?wsdl	0.145	0.5959	1.5525	0.4509	1.4075

It clear from the data in Table 6 that, compared with WSRec, the proposed PFPre performed better in terms of prediction errors. For the ranking lists based on the actual values of the response time in ascending order, the list of the predicted values obtained from the proposed PFPre method was more consistent with the ranking lists of the actual values. Thus, the proposed method is suitable for QoS-based web service recommendation platforms.

## 6 Summary

This paper analyzes the prediction errors in the conventional collaborative filtering methods that result from unknown differences in user preferences by introducing definitions of for user preference ranges. For different types of QoS attributes, different extraction rules were used to extract user preference matrices from the original QoS data. Based on this, the Euclidean similarity was used (instead of the conventional Pearson similarity) to perform the calculations. During the process of sifting the similar neighbors, the negative-value-based top-K method was used and all the optimized results were incorporated into the final collaborative prediction method. Finally, a reduction calculation was performed on the prediction results. The main advantage of the proposed algorithm is that it can fully extract the individual differences among various users and overcome the problem of inconsistent value ranges of QoS attributes, thereby avoiding the prediction errors caused by directly using the QoS data.

## Supporting information

S1 Dataset (DOCX)
