# Supplementary material for: Collaborative prediction of web service quality based on user preferences and services
Source: PLoS One. 2020 Dec 7;15(12):e0242089. doi: 10.1371/journal.pone.0242089 (PMC7721135; doi:10.1371/journal.pone.0242089)
Supplement: S1 Dataset — (DOCX) [file pone.0242089.s001.docx]

The experimental dataset was the QoSDataset2 from the publicly released WS-DREAM and the Web service searching engines: xmethods.net. The experimental dataset was the QoSDataset2 from the publicly released WS-DREAM, and the Web service searching engines: xmethods.net. The dataset includes 5301 Web services,214 Services Users and response time.

We constructed three user-service matrices with different of size = 100 × 100, size = 100 × 150, size = 150 × 100 by randomly extracting a certain number of users and services.

**5301 Web services**

[Service ID] [WSDL Address] [Service Provider] [IP Address] [Country] [IP No.] [AS] [Latitude] [Longitude]

=================================================================================

0 http://ewave.no-ip.com/ECallws/CinemaData.asmx?WSDL no-ip.com 8.23.224.110 United States 135782510 AS3356 Level 3 Communications 38 -97

1 http://ewave.no-ip.com/ECallws/StadiumSinchronization.asmx?WSDL no-ip.com 8.23.224.110 United States 135782510 AS3356 Level 3 Communications 38 -97

2 http://ewave.no-ip.com/EcallWS/CinemaSinchronization.asmx?WSDL no-ip.com 8.23.224.110 United States 135782510 AS3356 Level 3 Communications 38 -97

3 http://ewave.no-ip.com/ECallws/StadiumData.asmx?WSDL no-ip.com 8.23.224.110 United States 135782510 AS3356 Level 3 Communications 38 -97

4 http://ewave.no-ip.com/ECallws/BuyerData.asmx?WSDL no-ip.com 8.23.224.110 United States 135782510 AS3356 Level 3 Communications 38 -97

5 http://aquest.dyndns.org/CaptchaAudioWS/CaptchaAudio.asmx?WSDL dyndns.org 204.13.248.116 United States 3423467636 AS33517 Dynamic Network Services, Inc. 40.7904 -74.0246

6 http://www.utn.edu.ar/WebServices/ListaTics.asmx?WSDL utn.edu.ar null Argentina 0 null -45.5881 -69.07

7 http://www.utn.edu.ar/WebServices/WSAcademico.asmx?WSDL utn.edu.ar null Argentina 0 null -45.5881 -69.07

8 http://200.45.113.149/SC.BlackBerryClients.WS/Service.asmx?WSDL 200.45.113.149 200.45.113.149 Argentina 3358421397 AS7303 Telecom Argentina S.A. -34.5875 -58.6725

9 http://xml.dev.hoteldo.com/HotelDoInterface.asmx?WSDL hoteldo.com 200.59.145.167 Argentina 3359347111 AS11664 Techtel LMDS Comunicaciones Interactivas S.A. -34.4714 -58.5078

10 http://www.librosar.com.ar/portal/servicioonix.asmx?WSDL librosar.com.ar null Argentina 0 null null null

11 http://ba.mobilenik.com.ar/dbbrowser/dbbrowser.asmx?WSDL mobilenik.com.ar null Argentina 0 null null null

12 http://www.mslatam.com/latam/msdn/comunidad/dce/estadistica/webservices/FSD_Statistics_WS.asmx?WSDL mslatam.com 65.55.39.10 Argentina 1094133514 AS8075 Microsoft Corp 47.6801 -122.1206

13 http://www.neodynamic.com/Products/Demos/BCWebSampleWS/BarcodeProfessionalWS.asmx?wsdl neodynamic.com 190.2.60.187 Argentina 3187817659 AS16814 S.A. -32.9468 -60.6393

14 http://www.ssat.com.ar/googleearth1.1/service.asmx?wsdl ssat.com.ar 200.127.117.188 Argentina 3363796412 AS10481 Prima S.A. -34.7765 -58.4069

15 http://www.transportesjoselito.com/atlas/modal/TiempoService.asmx?WSDL transportesjoselito.com 200.58.118.130 Argentina 3359274626 AS27823 Dattatec.com -32.9468 -60.6393

16 http://wsatebara.com.ar/ServicioWeb.asmx?WSDL wsatebara.com.ar 190.247.40.16 Argentina 3203868688 AS10318 S.A. -34.5875 -58.6725

17 http://wsluisbal.com.ar/WebService.asmx?WSDL wsluisbal.com.ar 190.247.40.16 Argentina 3203868688 AS10318 S.A. -34.5875 -58.6725

18 http://www.enterpriseconnect.gov.au/_vti_bin/BusinessDataCatalog.asmx?wsdl enterpriseconnect.gov.au 210.193.179.225 Australia 3535909857 AS17477 Macquarie Telecom -27 133

19 http://www.enterpriseconnect.gov.au/_vti_bin/People.asmx?wsdl enterpriseconnect.gov.au 210.193.179.225 Australia 3535909857 AS17477 Macquarie Telecom -27 133

20 http://www.enterpriseconnect.gov.au/_vti_bin/Authentication.asmx?wsdl enterpriseconnect.gov.au 210.193.179.225 Australia 3535909857 AS17477 Macquarie Telecom -27 133

21 http://www.industry.gov.au/_vti_bin/BusinessDataCatalog.asmx?wsdl industry.gov.au 210.193.176.79 Australia 3535908943 AS17477 Macquarie Telecom -35.276 149.1344

22 http://www.industry.gov.au/_vti_bin/People.asmx?wsdl industry.gov.au 210.193.176.79 Australia 3535908943 AS17477 Macquarie Telecom -35.276 149.1344

23 http://www.industry.gov.au/_vti_bin/Authentication.asmx?wsdl industry.gov.au 210.193.176.79 Australia 3535908943 AS17477 Macquarie Telecom -35.276 149.1344

24 http://www.microsoftsharepoint.com/venue/_vti_bin/SharepointEmailWS.asmx?wsdl microsoftsharepoint.com 203.19.66.79 Australia 3407037007 AS9268 Over The Wire Pty Ltd -27 133

25 http://www.microsoftsharepoint.com/venue/_vti_bin/BusinessDataCatalog.asmx?wsdl microsoftsharepoint.com 203.19.66.79 Australia 3407037007 AS9268 Over The Wire Pty Ltd -27 133

26 http://www.microsoftsharepoint.com/agenda/_vti_bin/Authentication.asmx?wsdl microsoftsharepoint.com 203.19.66.79 Australia 3407037007 AS9268 Over The Wire Pty Ltd -27 133

27 http://www.microsoftsharepoint.com/agenda/_vti_bin/People.asmx?wsdl microsoftsharepoint.com 203.19.66.79 Australia 3407037007 AS9268 Over The Wire Pty Ltd -27 133

28 http://www.tourism.wa.gov.au/WA_Tourism_Network/_vti_bin/UserProfileService.asmx?wsdl tourism.wa.gov.au 203.153.245.135 Australia 3415864711 AS9822 Amnet IT Services Pty Ltd -31.9522 115.8614

29 http://www.tourism.wa.gov.au/WA_Tourism_Network/_vti_bin/Authentication.asmx?wsdl tourism.wa.gov.au 203.153.245.135 Australia 3415864711 AS9822 Amnet IT Services Pty Ltd -31.9522 115.8614

30 http://www.tourism.wa.gov.au/WA_Tourism_Network/_vti_bin/People.asmx?wsdl tourism.wa.gov.au 203.153.245.135 Australia 3415864711 AS9822 Amnet IT Services Pty Ltd -31.9522 115.8614

31 http://www.westernaustralia.com/de/_vti_bin/UserProfileService.asmx?wsdl westernaustralia.com 203.153.245.134 Germany 3415864710 AS9822 Amnet IT Services Pty Ltd -31.9522 115.8614

32 http://www.westernaustralia.com/de/_vti_bin/Authentication.asmx?wsdl westernaustralia.com 203.153.245.134 Germany 3415864710 AS9822 Amnet IT Services Pty Ltd -31.9522 115.8614

33 http://www.westernaustralia.com/de/_vti_bin/People.asmx?wsdl westernaustralia.com 203.153.245.134 Germany 3415864710 AS9822 Amnet IT Services Pty Ltd -31.9522 115.8614

34 http://www.stpatricks.qld.edu.au/_vti_bin/Authentication.asmx?wsdl stpatricks.qld.edu.au 110.232.140.115 Australia 1860734067 null -33.8615 151.2055

35 http://www.stpatricks.qld.edu.au/_vti_bin/People.asmx?wsdl stpatricks.qld.edu.au 110.232.140.115 Australia 1860734067 null -33.8615 151.2055

36 http://www.iress.com.au/AutoComplete.asmx?WSDL iress.com.au 203.32.184.246 Australia 3407919350 AS17755 BRIDGEDFS LIMITED -27 133

37 http://www.shn.org.au/UserAddModify.asmx?wsdl shn.org.au 203.12.31.159 Australia 3406569375 AS2764 AAPT Limited -37.8232 144.9729

38 http://services1.pharmx.com.au/order/supplierlist.asmx?WSDL pharmx.com.au 203.145.49.232 Australia 3415290344 AS17920 Ultra Serve Internet Pty Ltd - Transit AS - Hosting Provider, -33.4333 151.3833

39 http://www.abn.business.gov.au/abrxmlpubsub/ABRXMLPubSub.asmx?WSDL business.gov.au 210.193.176.122 Australia 3535908986 AS17477 Macquarie Telecom -35.276 149.1344

40 http://www.crickscore.com/services/Version?wsdl crickscore.com 208.73.211.152 Australia 3494499224 AS33626 Oversee.net 34.0533 -118.2549

41 http://www.crickscore.com/services/TeamAccess?wsdl crickscore.com 208.73.211.152 Australia 3494499224 AS33626 Oversee.net 34.0533 -118.2549

42 http://crickscore.com/services/Statistics?wsdl crickscore.com 208.73.211.152 Australia 3494499224 AS33626 Oversee.net 34.0533 -118.2549

43 http://au.gamecreate.com/admin/ExternalAuthentication.asmx?WSDL gamecreate.com 144.140.155.10 Australia 2425133834 AS1221 Telstra Pty Ltd -27 133

44 http://api.godo.com.au/soap/v1_00/productManager.cfc?WSDL godo.com.au 202.125.108.41 Australia 3397217321 AS55481 Wotif.com Holdings Limited -27 133

45 http://api.godo.com.au/soap/v1_00/product.cfc?WSDL godo.com.au 202.125.108.41 Australia 3397217321 AS55481 Wotif.com Holdings Limited -27 133

46 http://api.godo.com.au/soap/v1_00/licenceManager.cfc?WSDL godo.com.au 202.125.108.41 Australia 3397217321 AS55481 Wotif.com Holdings Limited -27 133

47 http://api.godo.com.au/soap/v1_00/order.cfc?WSDL godo.com.au 202.125.108.41 Australia 3397217321 AS55481 Wotif.com Holdings Limited -27 133

48 http://www.mp3.com.au/WebServices/PlayerService.asmx?WSDL mp3.com.au 50.23.104.22 Australia 840394774 null 37.3394 -121.895

49 http://www.mp3.com.au/WebServices/GalleryService.asmx?WSDL mp3.com.au 50.23.104.22 Australia 840394774 null 37.3394 -121.895

50 http://www.mp3.com.au/WebServices/MediaGroupService.asmx?WSDL mp3.com.au 50.23.104.22 Australia 840394774 null 37.3394 -121.895

51 http://www.mp3.com.au/WebServices/LocationService.asmx?WSDL mp3.com.au 50.23.104.22 Australia 840394774 null 37.3394 -121.895

52 http://webservices.seek.com.au/FastLanePlus.asmx?wsdl seek.com.au 202.58.38.95 Australia 3392808543 AS9667 Hostworks Pty Ltd -27 133

53 http://webct.swin.edu.au/webct/axis/Version?wsdl swin.edu.au 136.186.1.12 Australia 2293891340 AS7575 Australian Academic and Reasearch Network (AARNet) -27 133

54 http://soap.bondwireless.com/soap/services/SmsGateway?wsdl bondwireless.com 203.56.34.99 Australia 3409453667 AS9822 Amnet IT Services Pty Ltd -34.9286 138.6007

55 http://soap.bondwireless.com/soap/services/Enigma?wsdl bondwireless.com 203.56.34.99 Australia 3409453667 AS9822 Amnet IT Services Pty Ltd -34.9286 138.6007

56 http://maps.nsw.gov.au/wsdl/AddressSearch.wsdl maps.nsw.gov.au 203.57.8.27 Australia 3409512475 AS17668 Soul Pattinson Telecommunications Pty Ltd -33.4708 149.6033

57 http://maps.nsw.gov.au/wsdl/AddressImageWS.wsdl maps.nsw.gov.au 203.57.8.27 Australia 3409512475 AS17668 Soul Pattinson Telecommunications Pty Ltd -33.4708 149.6033

58 http://www.myboot.com.au/webservice/shared/galleries/resources.asmx?WSDL myboot.com.au 5.9.147.18 Australia 84513554 null 51 9

59 http://www.myboot.com.au/webservice/myboot/suburb/picker.asmx?WSDL myboot.com.au 5.9.147.18 Australia 84513554 null 51 9

60 http://www.originalsfestival.com.au/PublicService.asmx?WSDL originalsfestival.com.au null Australia 0 null null null

61 http://xml.redcoal.com/soapserver.dll/wsdl/ISoapServer redcoal.com 59.154.43.98 Australia 999959394 AS7474 SingTel Optus Pty Ltd -27 133

62 http://www.stratapay.com.au/ecommservices.asmx?WSDL stratapay.com.au 61.29.75.116 Australia 1025330036 AS2764 AAPT Limited -27.465 153.0234

63 http://sti.subaru.com.au/templates/global/cfc/banner_home.cfc?wsdl subaru.com.au 50.56.52.236 Australia 842544364 null 29.4889 -98.3987

64 http://sti.subaru.com.au/templates/global/cfc/banner_showrm.cfc?wsdl subaru.com.au 50.56.52.236 Australia 842544364 null 29.4889 -98.3987

65 http://direct.tab.com.au/LiveOdds/Services/Racing/Racing.wsdl tab.com.au 72.13.32.43 Australia 1208819755 AS26134 VeriSign Infrastructure & Operations 38.9599 -77.3428

66 http://direct.tab.com.au/liveodds/services/login/login.wsdl tab.com.au 72.13.32.43 Australia 1208819755 AS26134 VeriSign Infrastructure & Operations 38.9599 -77.3428

67 http://www.taet.com.au/Web+Service+Agent.nsf/RandomDirectoryWSDL taet.com.au null Australia 0 null null null

68 http://www.taet.com.au/Web+Service+Agent.nsf/DirectoryWSDL taet.com.au null Australia 0 null null null

69 http://staff.it.uts.edu.au/~chw/hellorpc.php?wsdl uts.edu.au 54.251.117.70 Australia 922449222 null 1.3667 103.8

70 http://blackboxlbs.com/BlackBoxWebservice/BlackBoxMiddleTier.asmx?WSDL blackboxlbs.com null Australia 0 null null null

71 http://www.connectingcare.com/webservice/ccereferral.asmx?wsdl connectingcare.com 203.94.158.108 Australia 3411975788 AS10223 Uecomm Ltd -37.8139 144.9634

72 http://webapp.genexis.com.au/twitterearth/Service.asmx?WSDL genexis.com.au 203.98.80.147 Australia 3412218003 AS45152 ASN Zone Networks -27 133

73 http://www.golfintelligence.com.au/course.asmx?WSDL golfintelligence.com.au 103.4.213.31 Australia 1728369951 null -27 133

74 http://engine.grunt.tv/2_1Discover/Discover.asmx?WSDL grunt.tv 203.14.199.246 Australia 3406743542 AS45427 Webzone Internet, Internet Service Provider, -27 133

75 http://hookmeup.com.au/Services/Service.asmx?WSDL hookmeup.com.au 111.67.13.116 Australia 1866665332 AS45454 Web24 Virtual & Dedicated hosting service provider, Melb, Australia -27 133

76 http://sp-s3g.iclp.com.au/ws/egapi/v1-0/egapi.cfc?wsdl iclp.com.au null Australia 0 null null null

77 http://www.interpro.com.au/JobsliveWS/RandomJobPicker.asmx?WSDL interpro.com.au 203.34.3.7 Australia 3408003847 AS17477 Macquarie Telecom -33.8615 151.2055

78 http://www.messagenet.com.au/dotnet/Lodge.asmx?wsdl messagenet.com.au 202.168.6.30 Australia 3400009246 AS9942 SOUL Converged Communications Australia -37.7667 144.9167

79 http://motormouth.net.au/Services/FuelPricingServices.asmx?wsdl motormouth.net.au 203.39.223.150 Australia 3408387990 AS1221 Telstra Pty Ltd -27.4833 152.9833

80 http://www.rugbyleaguelive.com/ContentService.asmx?wsdl rugbyleaguelive.com 50.16.232.234 Australia 839969002 AS14618 Amazon.com, Inc. 39.0437 -77.4875

81 http://www.secureparking.com.au/CarsService.asmx?WSDL secureparking.com.au 27.121.104.110 Australia 460941422 AS23992 Offis Pty Ltd -27 133

82 http://www.smsglobal.com.au/mobileworks/soapserver.php?wsdl smsglobal.com.au 203.89.199.52 Australia 3411658548 AS9328 Datacom Victoria -37.8139 144.9634

83 http://www.travelsim.net.au/phpincs/test/travelsim.wsdl travelsim.net.au 113.192.42.148 Australia 1908419220 null -33.8833 151.2167

84 http://webnet.usq.edu.au/USQProgramSearch/AutoCompleteSearch.asmx?WSDL usq.edu.au 139.86.7.80 Australia 2337670992 AS7575 Australian Academic and Reasearch Network (AARNet) -27 133

85 http://shop.virginvieathome.com/AddToBasketService.asmx?WSDL virginvieathome.com 194.176.199.129 Austria 3266365313 AS49572 Fujitsu Services Ltd. 51.5142 -0.0931

86 http://forums.genom-e.com/_vti_bin/BusinessDataCatalog.asmx?wsdl genom-e.com null Austria 0 null null null

87 http://forums.genom-e.com/_vti_bin/SharepointEmailWS.asmx?wsdl genom-e.com null Austria 0 null null null

88 http://forums.genom-e.com/_vti_bin/People.asmx?wsdl genom-e.com null Austria 0 null null null

89 http://forums.genom-e.com/_vti_bin/Authentication.asmx?wsdl genom-e.com null Austria 0 null null null

90 http://almighty.pri.univie.ac.at/~mangler/helloService.wsdl univie.ac.at 131.130.70.17 Austria 2206352913 AS760 University of Vienna, Austria 48.2 16.3667

91 http://almighty.pri.univie.ac.at/~mangler/ORA/ora.wsdl univie.ac.at 131.130.70.17 Austria 2206352913 AS760 University of Vienna, Austria 48.2 16.3667

92 http://www.pri.univie.ac.at/~mangler/CEWebS/Homework/ univie.ac.at 131.130.70.17 Austria 2206352913 AS760 University of Vienna, Austria 48.2 16.3667

93 http://www.arikan.at/axis/services/urn:xmltoday-delayed-quotes?wsdl arikan.at 64.15.156.118 Austria 1074764918 AS32613 iWeb Technologies Inc. 45.5 -73.5833

94 http://www.arikan.at/axis/services/BankService?wsdl arikan.at 64.15.156.118 Austria 1074764918 AS32613 iWeb Technologies Inc. 45.5 -73.5833

95 http://www.arikan.at/axis/services/CountryService?wsdl arikan.at 64.15.156.118 Austria 1074764918 AS32613 iWeb Technologies Inc. 45.5 -73.5833

96 http://www.arikan.at/axis/services/Version?wsdl arikan.at 64.15.156.118 Austria 1074764918 AS32613 iWeb Technologies Inc. 45.5 -73.5833

97 http://www.arikan.at/axis/services/DistrictService?wsdl arikan.at 64.15.156.118 Austria 1074764918 AS32613 iWeb Technologies Inc. 45.5 -73.5833

98 http://www.arikan.at/axis/services/InsuranceService?wsdl arikan.at 64.15.156.118 Austria 1074764918 AS32613 iWeb Technologies Inc. 45.5 -73.5833

99 http://www.arikan.at/axis/services/urn:cominfo?wsdl arikan.at 64.15.156.118 Austria 1074764918 AS32613 iWeb Technologies Inc. 45.5 -73.5833

100 http://www.arikan.at/axis/services/SOAPMonitorService?wsdl arikan.at 64.15.156.118 Austria 1074764918 AS32613 iWeb Technologies Inc. 45.5 -73.5833

101 http://office.faircheck.at/paraTesting/WebService/SVSR_Termin.asmx?WSDL faircheck.at 91.227.204.35 Austria 1541655587 null 47.3333 13.3333

102 http://office.faircheck.at/paraTesting/WebService/SVSR_Akt_Lookup.asmx?WSDL faircheck.at 91.227.204.35 Austria 1541655587 null 47.3333 13.3333

103 http://olymp.ifs.tuwien.ac.at:8088/SOMTrainer?wsdl tuwien.ac.at 128.130.35.76 Austria 2156012364 AS679 Vienna University of Technology 48.2 16.3667

104 http://olymp.ifs.tuwien.ac.at:8088/AudioFeatureExtractor?wsdl tuwien.ac.at 128.130.35.76 Austria 2156012364 AS679 Vienna University of Technology 48.2 16.3667

105 http://madrid.vitalab.tuwien.ac.at:8152/axis2/services/SkypeWebService?wsdl tuwien.ac.at 128.130.35.76 Austria 2156012364 AS679 Vienna University of Technology 48.2 16.3667

106 http://dconx.biz/messagestest.asmx?WSDL dconx.biz 193.56.137.12 France 3241707788 AS15846 Autonomous System 48.6328 2.4405

107 http://dconx.biz/messages.asmx?WSDL dconx.biz 193.56.137.12 France 3241707788 AS15846 Autonomous System 48.6328 2.4405

108 http://dotnet.jku.at/csbook/solutions/19/BookStoreService.asmx?WSDL jku.at 140.78.3.160 Austria 2353922976 AS1205 Johannes Kepler University 48.3 14.3

109 http://dotnet.jku.at/buch/samples/7/simple/TimeService1.asmx?WSDL jku.at 140.78.3.160 Austria 2353922976 AS1205 Johannes Kepler University 48.3 14.3

110 http://dotnet.jku.at/book/samples/7/encoding/PersonService.asmx?WSDL jku.at 140.78.3.160 Austria 2353922976 AS1205 Johannes Kepler University 48.3 14.3

111 http://dotnet.jku.at/book/samples/7/lifecycle/StateDemo.asmx?WSDL jku.at 140.78.3.160 Austria 2353922976 AS1205 Johannes Kepler University 48.3 14.3

112 http://www.elba.at/SOAP/SOAPServer.php?wsdl elba.at null Austria 0 null null null

113 http://travel.jet.at/JtrWebService/JtrWebService.asmx?WSDL jet.at 94.247.144.176 Austria 1593282736 AS42572 abaton EDV - Dienstleistungs GmbH 47.3333 13.3333

114 http://www.allesheute.at/kinoimport/import.asmx?WSDL allesheute.at 212.72.171.211 Austria 3561532371 AS8893 Artfiles New Media GmbH 51 9

115 http://www.noe.bauakademie.at/importexport/export.asmx?WSDL bauakademie.at null Austria 0 null null null

116 http://limitz.jugendsport.at/wsdl.php?wsdl jugendsport.at null Germany 0 null null null

117 http://jukebox.ch/Interpretenliste.asmx?WSDL jukebox.ch 193.33.216.10 Austria 3240220682 AS42871 Michael Gamsjaeger 47.7167 13.6167

118 http://www.nanonull.com/TimeService/TimeService.asmx?wsdl nanonull.com 62.218.28.242 Austria 1054481650 AS8437 UTA.AT Backbone 47.8 16.25

119 http://wartung.priso.net/Service1.asmx?WSDL priso.net null Austria 0 null null null

120 http://www.calstatela.edu/faculty/jmiller6/cs454-winter2003/notes/sort.wsdl mywebsite.com 216.250.121.107 Bahamas 3640293739 AS7296 Alchemy Communications, Inc. 40.0548 -75.4083

121 http://www.bisc.by/blank/WEB.WebServ.CLS?WSDL=1 bisc.by 212.98.168.182 Belarus 3563235510 AS12406 Business network j.v. 53 28

122 http://www.g-o.be/_vti_bin/BusinessDataCatalog.asmx?wsdl g-o.be null Belgium 0 null null null

123 http://www.g-o.be/_vti_bin/People.asmx?wsdl g-o.be null Belgium 0 null null null

124 http://www.g-o.be/_vti_bin/Authentication.asmx?wsdl g-o.be null Belgium 0 null null null

125 http://www.sra.nl/_vti_bin/SharepointEmailWS.asmx?wsdl sra.nl 213.154.250.200 Belgium 3583703752 AS12859 BIT BV 52.5 5.75

126 http://www.sra.nl/_vti_bin/BusinessDataCatalog.asmx?wsdl sra.nl 213.154.250.200 Belgium 3583703752 AS12859 BIT BV 52.5 5.75

127 http://www.sra.nl/_vti_bin/People.asmx?wsdl sra.nl 213.154.250.200 Belgium 3583703752 AS12859 BIT BV 52.5 5.75

128 http://www.sra.nl/_vti_bin/Authentication.asmx?wsdl sra.nl 213.154.250.200 Belgium 3583703752 AS12859 BIT BV 52.5 5.75

129 http://www.u2u.info/_vti_bin/BusinessDataCatalog.asmx?wsdl u2u.info null Belgium 0 null null null

130 http://www.u2u.info/_vti_bin/People.asmx?wsdl u2u.info null Belgium 0 null null null

131 http://www.u2u.info/_vti_bin/Authentication.asmx?wsdl u2u.info null Belgium 0 null null null

132 http://tomcat.esat.kuleuven.be/axis/services/Version?wsdl kuleuven.be 134.58.64.15 Belgium 2251964431 AS2611 AS for BELNET, The Belgian National Research and Education Network 50.8833 4.7

133 http://ariadne.cs.kuleuven.be/AWS/services/SessionManagementService?wsdl kuleuven.be 134.58.64.15 Belgium 2251964431 AS2611 AS for BELNET, The Belgian National Research and Education Network 50.8833 4.7

134 http://tomcat.esat.kuleuven.be/axis/ToucanSOAPService.jws?wsdl kuleuven.be 134.58.64.15 Belgium 2251964431 AS2611 AS for BELNET, The Belgian National Research and Education Network 50.8833 4.7

135 http://ariadne.cs.kuleuven.ac.be/SQI/wsdl/SessionManagement.wsdl kuleuven.ac.be 134.58.64.15 Belgium 2251964431 AS2611 AS for BELNET, The Belgian National Research and Education Network 50.8833 4.7

136 http://agiv.be/gis/localService.asmx?WSDL agiv.be 193.190.76.50 Belgium 3250474034 AS2611 AS for BELNET, The Belgian National Research and Education Network 51.05 3.7167

137 http://jure.juridat.just.fgov.be/PORTALSEARCH/PortalSearchWSSoapHttpPort?WSDL fgov.be 193.191.245.4 Belgium 3250582788 AS2611 AS for BELNET, The Belgian National Research and Education Network 50.8333 4

138 http://www.idtdna.com/AnalyzerService/AnalyzerService.asmx?WSDL idtdna.com 67.130.12.43 United States 1132596267 AS209 Qwest Communications Company, LLC 45.1708 -93.5758

139 http://eu.idtdna.com/Ajax/ModService.asmx?wsdl idtdna.com 67.130.12.43 Belgium 1132596267 AS209 Qwest Communications Company, LLC 45.1708 -93.5758

140 http://www.vlm.be/_vti_bin/WebPartPages.asmx?wsdl vlm.be 193.58.158.206 Belgium 3241844430 AS2611 AS for BELNET, The Belgian National Research and Education Network 50.8333 4

141 http://www.ebcs.be/iban/IBANBIC.asmx?WSDL ebcs.be 96.45.82.132 Belgium 1613582980 null 38.9311 -77.3489

142 http://www.ebcs.be/iban/Olo.asmx?WSDL ebcs.be 96.45.82.68 Belgium 1613582916 null 38.9311 -77.3489

143 http://www.ebcs.be/iban/Cijfers.asmx?WSDL ebcs.be 96.45.82.4 Belgium 1613582852 null 38.9311 -77.3489

144 http://www.webservices.etnic.be/ws_libelle/convert_libelle.wsdl etnic.be 193.190.96.74 Belgium 3250479178 AS2611 AS for BELNET, The Belgian National Research and Education Network 50.8333 4.3333

145 http://www.ibanbic.be/Cijfers.asmx?WSDL ibanbic.be 96.45.82.4 Belgium 1613582852 null 38.9311 -77.3489

146 http://www.ibanbic.be/Olo.asmx?WSDL ibanbic.be 96.45.82.196 Belgium 1613583044 null 38.9311 -77.3489

147 http://www.ibanbic.be/IBANBIC.asmx?WSDL ibanbic.be 96.45.82.132 Belgium 1613582980 null 38.9311 -77.3489

148 http://kbcactions.microsite.be/festivals09/service.asmx?WSDL microsite.be null Belgium 0 null null null

149 http://walibi.microsite.be/hero_service/hero_service.asmx?WSDL microsite.be null Belgium 0 null null null

150 http://mercedesdreamfactory.microsite.be/ws/ws.asmx?WSDL microsite.be null Belgium 0 null null null

151 http://kayak.environnement.wallonie.be/services/WSStationsNL?wsdl wallonie.be 157.164.136.250 Belgium 2644805882 AS2611 AS for BELNET, The Belgian National Research and Education Network 50.8333 4

152 http://www.4allsms.com/sendsms.asmx?WSDL 4allsms.com 31.24.112.60 Belgium 521695292 null 50.8333 4

153 http://budgetassistant.axionweb.be/service.asmx?WSDL axionweb.be 212.63.233.29 Belgium 3560958237 AS25367 For multihomed access of Banque Internationale a Luxembourg, in Luxemburg 50.8333 4

154 http://staging.blueridge.be/DSXMLSigner/main.asmx?WSDL blueridge.be null Belgium 0 null null null

155 http://clacpercussie.be/controls/flashnews/content.asmx?wsdl clacpercussie.be 46.30.211.60 Belgium 773772092 null 55.6667 12.5833

156 http://services.groupes.be/iMobilWebService/iMobilWS.asmx?WSDL groupes.be 194.7.30.151 Belgium 3255246487 AS702 Verizon Business EMEA - Commercial IP service provider in Europe 50.8333 4.3333

157 http://www.hotels-europe.com/webservices/General.asmx?WSDL hotels-europe.com 178.18.80.177 Belgium 2987544753 AS35470 XL Network 52.5 5.75

158 http://www.it-jobbank.co.uk/(A(QUnPf3I7XCdLYI5zAA44K-fQ2fl_9MR1yoaRY_lGCkUIBGbMs4gQe6BkO-dN0Lur9ViHHxr5PupUf1kyw3xbO3CRQS3TyJaeBPkLv8pIZ5U1))/WebServices/JobFeed.asmx?wsdl it-jobbank.co.uk 81.246.117.104 Belgium 1375106408 AS5432 Belgacom regional ASN 50.8333 4

159 http://marinespecies.org/aphia.php?p=soap&wsdl=1 marinespecies.org 193.191.134.5 Belgium 3250554373 AS2611 AS for BELNET, The Belgian National Research and Education Network 50.8333 4

160 http://fe.bcc.proximity.be/WS/BCC_LINK_2008.asmx?WSDL proximity.be 217.111.147.8 Belgium 3647968008 AS8220 COLT Technology Services 50.8333 4

161 http://www.spotter.be/Tech/MakeTickerData.asmx?wsdl spotter.be 212.123.12.136 Belgium 3564833928 AS6848 Telenet Operaties N.V. 50.9333 4.4333

162 http://www.tiensesuiker.com/service/service.asmx?WSDL tiensesuiker.com 212.113.74.56 Belgium 3564194360 AS9166 HostIT Hasselt Belgium 50.8667 4.25

163 http://www.vladars.net/_vti_bin/BusinessDataCatalog.asmx?wsdl vladars.net 80.87.254.4 Bosnia and Herzegovina 1347943940 AS16145 LANACO INECCO ISP 44.7784 17.1939

164 http://www.vladars.net/sr-SP-Cyrl/_vti_bin/People.asmx?wsdl vladars.net 80.87.254.4 Bosnia and Herzegovina 1347943940 AS16145 LANACO INECCO ISP 44.7784 17.1939

165 http://www.vladars.net/sr-SP-Cyrl/_vti_bin/Authentication.asmx?wsdl vladars.net 80.87.254.4 Bosnia and Herzegovina 1347943940 AS16145 LANACO INECCO ISP 44.7784 17.1939

166 http://www.blberza.com/services/blse/ticker.asmx?WSDL blberza.com null Bosnia and Herzegovina 0 null null null

167 http://www.innovatti.com.br/_vti_bin/search.asmx?wsdl innovatti.com.br 200.212.125.99 Brazil 3369368931 AS4230 Embratel -10 -55

168 http://www.innovatti.com.br/_vti_bin/AreaService.asmx?wsdl innovatti.com.br 200.212.125.99 Brazil 3369368931 AS4230 Embratel -10 -55

169 http://www.innovatti.com.br/_vti_bin/UserProfileService.asmx?wsdl innovatti.com.br 200.212.125.99 Brazil 3369368931 AS4230 Embratel -10 -55

170 http://www.innovatti.com.br/_vti_bin/Lists.asmx?wsdl innovatti.com.br 200.212.125.99 Brazil 3369368931 AS4230 Embratel -10 -55

171 http://seguro.brti.com.br/brtsi/services/ExternalAccountEventWS?wsdl brti.com.br null Brazil 0 null null null

172 http://seguro.brti.com.br/brtsi/services/CustomerSearchWS?wsdl brti.com.br null Brazil 0 null null null

173 http://desenv.tce.sc.gov.br:8080/axis/WSeO.jws?wsdl sc.gov.br 200.19.215.26 Brazil 3356743450 AS11802 CIASC -10 -55

174 http://sistemas.sefaz.ba.gov.br/webservices/CL/wsCFP.asmx?WSDL ba.gov.br null Brazil 0 null null null

175 http://centros.bvsalud.org/webservices/index.php?wsdl bvsalud.org 200.10.179.43 Brazil 3356144427 AS1251 Fundacao de Amparo a Pesquisa do Estado de Sao Pau -23.4733 -46.6658

176 http://www.bvsalud.org/apps/basesws/index.php?wsdl bvsalud.org 200.10.179.43 Brazil 3356144427 AS1251 Fundacao de Amparo a Pesquisa do Estado de Sao Pau -23.4733 -46.6658

177 http://cochrane.bvsalud.org/webservices/index.php?wsdl bvsalud.org 200.10.179.43 Brazil 3356144427 AS1251 Fundacao de Amparo a Pesquisa do Estado de Sao Pau -23.4733 -46.6658

178 http://hidroweb.ana.gov.br/fcthservices/mma.asmx?WSDL ana.gov.br 177.69.158.132 Brazil 2974129796 null -15.7833 -47.9167

179 http://estrela.angeloni.com.br:8888/estrela/ws/jws/PesquisaWS.jws?wsdl angeloni.com.br 186.251.38.9 Brazil 3137021449 null -10 -55

180 http://estrela.angeloni.com.br:8888/estrela/ws/jws/TesteWS.jws?wsdl angeloni.com.br 186.251.38.9 Brazil 3137021449 null -10 -55

181 http://www.checkexpress.com.br/ws_office_bacen/registration.asmx?WSDL checkexpress.com.br 200.162.48.134 Brazil 3366072454 AS15180 Diveo do Brasil Telecomunicacoes Ltda -23.4733 -46.6658

182 http://www.comunique-se.com.br/proveDados.asmx?WSDL comunique-se.com.br 200.255.218.70 Brazil 3372210758 AS4230 Embratel -10 -55

183 http://www.comunique-se.com.br/webDistribuidor.asmx?WSDL comunique-se.com.br 200.255.218.70 Brazil 3372210758 AS4230 Embratel -10 -55

184 http://www.sefaz.rs.gov.br/wsPIIFrs/wspiifrs.asmx?WSDL rs.gov.br null Brazil 0 null null null

185 http://www.scielo.br/webservices/indexBVS.php?wsdl www.scielo.br 200.136.72.9 Brazil 3364374537 AS1251 Fundacao de Amparo a Pesquisa do Estado de Sao Pau -10 -55

186 http://200.140.194.54/wspcceBarranquilla/wspcce.asmx?WSDL 200.140.194.54 200.140.194.54 Brazil 3364667958 AS8167 Telecomunicacoes de Santa Catarina SA -25.55 -54.5833

187 http://www.localizaanimal.com.br/wsdl.xml agricultura.gov.br 200.198.194.214 Brazil 3368469206 AS10954 Empresa do MinistÚAio da Fazenda -15.7833 -47.9167

188 http://sngpc.anvisa.gov.br/webservice/sngpc.asmx?WSDL anvisa.gov.br 200.198.201.69 Brazil 3368470853 AS10954 Empresa do MinistÚAio da Fazenda -15.7833 -47.9167

189 http://bases.bvs.br/webservices/index.php?wsdl bases.bvs.br 200.10.179.41 Brazil 3356144425 AS1251 Fundacao de Amparo a Pesquisa do Estado de Sao Pau -23.4733 -46.6658

190 http://www.bibliotecavirtualensalud.org/apps/basesws/index.php?wsdl bibliotecavirtualensalud.org null Brazil 0 null null null

191 http://www.bireme.org/apps/basesws/index.php?wsdl bireme.org 200.10.179.43 Brazil 3356144427 AS1251 Fundacao de Amparo a Pesquisa do Estado de Sao Pau -23.4733 -46.6658

192 http://www.bvsaude.org/apps/basesws/index.php?wsdl bvsaude.org null Brazil 0 null null null

193 http://www.clearsale.com.br/aplicacao/entrada.asmx?WSDL clearsale.com.br 98.129.108.201 United States 1652649161 AS33070 Rackspace Hosting 29.4889 -98.3987

194 http://cochrane.bireme.br/webservices/index.php?wsdl cochrane.bireme.br 200.10.179.18 Brazil 3356144402 AS1251 Fundacao de Amparo a Pesquisa do Estado de Sao Pau -23.4733 -46.6658

195 http://shopping.correios.com.br/wbm/shopping/script/consultarfrete.asmx?WSDL correios.com.br 200.252.60.80 Brazil 3371973712 AS4230 Embratel -10 -55

196 http://ws.cronostelemetria.com.br/WebServices/Service.asmx?wsdl cronostelemetria.com.br 97.74.214.1 Brazil 1632294401 AS26496 GoDaddy.com, Inc. 33.6119 -111.8906

197 http://www.digimess.com.br/ImportWS/ImportWS.asmx?wsdl digimess.com.br 200.234.196.163 Brazil 3370828963 AS27715 LocaWeb Ltda -10 -55

198 http://diretorios.bvs.br/webservices/index.php?wsdl diretorios.bvs.br 200.10.179.43 Brazil 3356144427 AS1251 Fundacao de Amparo a Pesquisa do Estado de Sao Pau -23.4733 -46.6658

199 http://suporte.infomed.inf.br/desenvtiss/Services/tissTransmiteMensagem.asmx?WSDL infomed.inf.br 200.249.177.3 Brazil 3371806979 AS4230 Embratel -7.1167 -34.8667

200 http://inf.unisinos.br/~swm/swservice.php?wsdl inf.unisinos.br 200.188.161.160 Brazil 3367805344 AS19611 Unisinos -10 -55

201 http://mercedes-benz.com.br/webservice/WSPalavra.asmx?WSDL mercedes-benz.com.br 187.86.211.2 Brazil 3143029506 AS53070 -10 -55

202 http://www.sicadi.com.br/mhouse/sicadi/webservices/indices.wsdl sicadi.com.br 187.8.150.202 Brazil 3137902282 AS10429 Telefonica Empresas SA -10 -55

203 http://www.smartwebsuite.net/webservice_meeting.asmx?WSDL smartwebsuite.net 186.202.184.108 Brazil 3133847660 null -23.4733 -46.6658

204 http://www.virtualhealthlibrary.org/apps/basesws/index.php?wsdl virtualhealthlibrary.org null Brazil 0 null null null

205 http://intranet.wappa.com.br/WebServices/Taxi.asmx?WSDL wappa.com.br 200.225.88.90 Brazil 3370211418 AS19089 Dedalus.com S/C Ltda -10 -55

206 http://www.wmbrasil.com.br/wmlicenca/webservice.asmx?WSDL wmbrasil.com.br 174.36.245.169 Brazil 2921657769 AS36351 SoftLayer Technologies Inc. 38.8951 -77.0364

207 http://www.bireme.br/apps/basesws/index.php?wsdl www.bireme.br 200.10.179.43 Brazil 3356144427 AS1251 Fundacao de Amparo a Pesquisa do Estado de Sao Pau -23.4733 -46.6658

208 http://www.bvs.br/apps/basesws/index.php?wsdl www.bvs.br 200.10.179.43 Brazil 3356144427 AS1251 Fundacao de Amparo a Pesquisa do Estado de Sao Pau -23.4733 -46.6658

209 http://www.zap.com.br/imoveis/CascadingDropServiceImoveis.asmx?WSDL zap.com.br 201.20.43.100 Brazil 3373542244 AS16397 Comdominio SA -10 -55

210 http://slk.icentres.net/_vti_bin/BusinessDataCatalog.asmx?wsdl icentres.net 69.43.161.168 Bulgaria 1160487336 AS22489 Castle Access Inc 32.8073 -117.1324

211 http://slk.icentres.net/_vti_bin/People.asmx?wsdl icentres.net 69.43.161.168 Bulgaria 1160487336 AS22489 Castle Access Inc 32.8073 -117.1324

212 http://slk.icentres.net/_vti_bin/Authentication.asmx?wsdl icentres.net 69.43.161.168 Bulgaria 1160487336 AS22489 Castle Access Inc 32.8073 -117.1324

213 http://www.law.uni-sofia.bg/_vti_bin/People.asmx?wsdl uni-sofia.bg 62.44.96.22 Bulgaria 1043095574 AS5421 Sofia University autonomous system 42.6833 23.3167

214 http://www.law.uni-sofia.bg/_vti_bin/Authentication.asmx?wsdl uni-sofia.bg 62.44.96.22 Bulgaria 1043095574 AS5421 Sofia University autonomous system 42.6833 23.3167

215 http://hs19.iccs.bas.bg/work/soap/books.php?wsdl bas.bg null Bulgaria 0 null null null

216 http://hs19.iccs.bas.bg/work/nusoap/nusoap.0.7.2/lib/addexample2.php?wsdl bas.bg null Bulgaria 0 null null null

217 http://www.postbank.bg/TransformationService.asmx?WSDL postbank.bg 195.242.126.251 Bulgaria 3287449339 AS41799 Eurobank EFG Bulgaria AD 42.6833 23.3167

218 http://biomoby.org/services/wsdl/icapture.ubc.ca/Gene2Ontology biordf.net 96.49.108.226 Canada 1613851874 AS6327 Shaw Communications Inc. 49.25 -123.1333

219 http://biomoby.org/services/wsdl/soaplab.icapture.ubc.ca/seqretPROTEINFASTA2NCBI biordf.net 96.49.108.226 Canada 1613851874 AS6327 Shaw Communications Inc. 49.25 -123.1333

220 http://biomoby.org/services/wsdl/soaplab.icapture.ubc.ca/seqretPROTEINSTADEN2FASTA biordf.net 96.49.108.226 Canada 1613851874 AS6327 Shaw Communications Inc. 49.25 -123.1333

221 http://biomoby.org/services/wsdl/soaplab.icapture.ubc.ca/seqretPROTEINRefNBRF2FASTA biordf.net 96.49.108.226 Canada 1613851874 AS6327 Shaw Communications Inc. 49.25 -123.1333

222 http://biomoby.org/services/wsdl/soaplab.icapture.ubc.ca/seqretPROTEINFASTA2CLUSTAL biordf.net 96.49.108.226 Canada 1613851874 AS6327 Shaw Communications Inc. 49.25 -123.1333

223 http://biomoby.org/services/wsdl/antirrhinum.net/parseDragonDBBlastText biordf.net 96.49.108.226 Canada 1613851874 AS6327 Shaw Communications Inc. 49.25 -123.1333

224 http://biomoby.org/services/wsdl/soaplab.icapture.ubc.ca/seqretDNAIG2FASTA biordf.net 96.49.108.226 Canada 1613851874 AS6327 Shaw Communications Inc. 49.25 -123.1333

225 http://biomoby.org/services/wsdl/atidb.org/getGOTermsByAGICode biordf.net 96.49.108.226 Canada 1613851874 AS6327 Shaw Communications Inc. 49.25 -123.1333

226 http://biomoby.org/services/wsdl/soaplab.icapture.ubc.ca/seqretPROTEINFASTA2NBRF biordf.net 96.49.108.226 Canada 1613851874 AS6327 Shaw Communications Inc. 49.25 -123.1333

227 http://biomoby.org/services/wsdl/soaplab.icapture.ubc.ca/seqretDNARefFASTA2NCBI biordf.net 96.49.108.226 Canada 1613851874 AS6327 Shaw Communications Inc. 49.25 -123.1333

228 http://biomoby.org/services/wsdl/soaplab.icapture.ubc.ca/seqretPROTEINTEXT2FASTA biordf.net 96.49.108.226 Canada 1613851874 AS6327 Shaw Communications Inc. 49.25 -123.1333

229 http://biomoby.org/services/wsdl/soaplab.icapture.ubc.ca/seqretPROTEINRefFASTA2PIR biordf.net 96.49.108.226 Canada 1613851874 AS6327 Shaw Communications Inc. 49.25 -123.1333

230 http://biomoby.org/services/wsdl/soaplab.icapture.ubc.ca/seqretDNARefFASTA2MSF biordf.net 96.49.108.226 Canada 1613851874 AS6327 Shaw Communications Inc. 49.25 -123.1333

231 http://biomoby.org/services/wsdl/soaplab.icapture.ubc.ca/revseqDNARefFASTA2FASTA biordf.net 96.49.108.226 Canada 1613851874 AS6327 Shaw Communications Inc. 49.25 -123.1333

232 http://biomoby.org/services/wsdl/soaplab.icapture.ubc.ca/seqretDNARefMEGA2FASTA biordf.net 96.49.108.226 Canada 1613851874 AS6327 Shaw Communications Inc. 49.25 -123.1333

233 http://biomoby.org/services/wsdl/bioinfo.icapture.ubc.ca/BIND_IdSearchGetFastaByEnsemblId biordf.net 96.49.108.226 Canada 1613851874 AS6327 Shaw Communications Inc. 49.25 -123.1333

234 http://biomoby.org/services/wsdl/soaplab.icapture.ubc.ca/seqretDNARefFASTA2RAW biordf.net 96.49.108.226 Canada 1613851874 AS6327 Shaw Communications Inc. 49.25 -123.1333

235 http://biomoby.org/services/wsdl/soaplab.icapture.ubc.ca/seqretPROTEINPIR2FASTA biordf.net 96.49.108.226 Canada 1613851874 AS6327 Shaw Communications Inc. 49.25 -123.1333

236 http://biomoby.org/services/wsdl/soaplab.icapture.ubc.ca/seqretDNAFASTA2IG biordf.net 96.49.108.226 Canada 1613851874 AS6327 Shaw Communications Inc. 49.25 -123.1333

237 http://biomoby.org/services/wsdl/atidb.org/getTargetPResultByTranscriptCode biordf.net 96.49.108.226 Canada 1613851874 AS6327 Shaw Communications Inc. 49.25 -123.1333

238 http://biomoby.org/services/wsdl/soaplab.icapture.ubc.ca/seqretDNARefFASTA2JACKKNIFERNON biordf.net 96.49.108.226 Canada 1613851874 AS6327 Shaw Communications Inc. 49.25 -123.1333

239 http://biomoby.org/services/wsdl/soaplab.icapture.ubc.ca/seqretPROTEINFASTA2GENBANK biordf.net 96.49.108.226 Canada 1613851874 AS6327 Shaw Communications Inc. 49.25 -123.1333

240 http://biomoby.org/services/wsdl/soaplab.icapture.ubc.ca/seqretPROTEINRefCLUSTAL2FASTA biordf.net 96.49.108.226 Canada 1613851874 AS6327 Shaw Communications Inc. 49.25 -123.1333

241 http://biomoby.org/services/wsdl/bioinfo.icapture.ubc.ca/BIND_IdSearchGetFastaBySmartId biordf.net 96.49.108.226 Canada 1613851874 AS6327 Shaw Communications Inc. 49.25 -123.1333

242 http://biomoby.org/services/wsdl/soaplab.icapture.ubc.ca/seqretDNARefFASTA2GENBANK biordf.net 96.49.108.226 Canada 1613851874 AS6327 Shaw Communications Inc. 49.25 -123.1333

243 http://biomoby.org/services/wsdl/soaplab.icapture.ubc.ca/seqretDNAFASTA2NEXUS biordf.net 96.49.108.226 Canada 1613851874 AS6327 Shaw Communications Inc. 49.25 -123.1333

244 http://biomoby.org/services/wsdl/soaplab.icapture.ubc.ca/seqretDNARefNBRF2FASTA biordf.net 96.49.108.226 Canada 1613851874 AS6327 Shaw Communications Inc. 49.25 -123.1333

245 http://biomoby.org/services/wsdl/soaplab.icapture.ubc.ca/revseqDNAFASTA2FASTA biordf.net 96.49.108.226 Canada 1613851874 AS6327 Shaw Communications Inc. 49.25 -123.1333

246 http://biomoby.org/services/wsdl/soaplab.icapture.ubc.ca/seqretPROTEINRefGCG2FASTA biordf.net 96.49.108.226 Canada 1613851874 AS6327 Shaw Communications Inc. 49.25 -123.1333

247 http://biomoby.org/services/wsdl/soaplab.icapture.ubc.ca/seqretDNARefNEXUSNON2FASTA biordf.net 96.49.108.226 Canada 1613851874 AS6327 Shaw Communications Inc. 49.25 -123.1333

248 http://biomoby.org/services/wsdl/soaplab.icapture.ubc.ca/seqretPROTEINRefTEXT2FASTA biordf.net 96.49.108.226 Canada 1613851874 AS6327 Shaw Communications Inc. 49.25 -123.1333

249 http://biomoby.org/services/wsdl/soaplab.icapture.ubc.ca/seqretPROTEINRefNEXUSNON2FASTA biordf.net 96.49.108.226 Canada 1613851874 AS6327 Shaw Communications Inc. 49.25 -123.1333

250 http://biomoby.org/services/wsdl/soaplab.icapture.ubc.ca/seqretDNAFASTA2EMBL biordf.net 96.49.108.226 Canada 1613851874 AS6327 Shaw Communications Inc. 49.25 -123.1333

251 http://biomoby.org/services/wsdl/soaplab.icapture.ubc.ca/seqretDNARefFASTA2STRIDER biordf.net 96.49.108.226 Canada 1613851874 AS6327 Shaw Communications Inc. 49.25 -123.1333

252 http://biomoby.org/services/wsdl/soaplab.icapture.ubc.ca/seqretPROTEINRefSWISS2FASTA biordf.net 96.49.108.226 Canada 1613851874 AS6327 Shaw Communications Inc. 49.25 -123.1333

253 http://biomoby.org/services/wsdl/soaplab.icapture.ubc.ca/seqretPROTEINCLUSTAL2FASTA biordf.net 96.49.108.226 Canada 1613851874 AS6327 Shaw Communications Inc. 49.25 -123.1333

254 http://biomoby.org/services/wsdl/bioinfo.icapture.ubc.ca/BIND_IdSearchGetGOTermsByOmimId biordf.net 96.49.108.226 Canada 1613851874 AS6327 Shaw Communications Inc. 49.25 -123.1333

255 http://biomoby.org/services/wsdl/soaplab.icapture.ubc.ca/seqretPROTEINFASTA2GCG8 biordf.net 96.49.108.226 Canada 1613851874 AS6327 Shaw Communications Inc. 49.25 -123.1333

256 http://biomoby.org/services/wsdl/soaplab.icapture.ubc.ca/seqretPROTEINNCBI2FASTA biordf.net 96.49.108.226 Canada 1613851874 AS6327 Shaw Communications Inc. 49.25 -123.1333

257 http://biomoby.org/services/wsdl/soaplab.icapture.ubc.ca/seqretPROTEINFASTA2JACKKNIFERNON biordf.net 96.49.108.226 Canada 1613851874 AS6327 Shaw Communications Inc. 49.25 -123.1333

258 http://biomoby.org/services/wsdl/soaplab.icapture.ubc.ca/seqretDNAFASTA2MEGA biordf.net 96.49.108.226 Canada 1613851874 AS6327 Shaw Communications Inc. 49.25 -123.1333

259 http://biomoby.org/services/wsdl/soaplab.icapture.ubc.ca/seqretPROTEINRefFASTA2CODATA biordf.net 96.49.108.226 Canada 1613851874 AS6327 Shaw Communications Inc. 49.25 -123.1333

260 http://biomoby.org/services/wsdl/soaplab.icapture.ubc.ca/seqretPROTEINFASTA2PHYLIP biordf.net 96.49.108.226 Canada 1613851874 AS6327 Shaw Communications Inc. 49.25 -123.1333

261 http://biomoby.org/services/wsdl/soaplab.icapture.ubc.ca/seqretDNAFASTA2NCBI biordf.net 96.49.108.226 Canada 1613851874 AS6327 Shaw Communications Inc. 49.25 -123.1333

262 http://biomoby.org/services/wsdl/soaplab.icapture.ubc.ca/seqretDNARefFASTA2NBRF biordf.net 96.49.108.226 Canada 1613851874 AS6327 Shaw Communications Inc. 49.25 -123.1333

263 http://biomoby.org/services/wsdl/soaplab.icapture.ubc.ca/seqretDNASTADEN2FASTA biordf.net 96.49.108.226 Canada 1613851874 AS6327 Shaw Communications Inc. 49.25 -123.1333

264 http://biomoby.org/services/wsdl/soaplab.icapture.ubc.ca/seqretPROTEINRefSTADEN2FASTA biordf.net 96.49.108.226 Canada 1613851874 AS6327 Shaw Communications Inc. 49.25 -123.1333

265 http://biomoby.org/services/wsdl/soaplab.icapture.ubc.ca/seqretPROTEINFASTA2NEXUS biordf.net 96.49.108.226 Canada 1613851874 AS6327 Shaw Communications Inc. 49.25 -123.1333

266 http://biomoby.org/services/wsdl/soaplab.icapture.ubc.ca/seqretDNARefACEDB2FASTA biordf.net 96.49.108.226 Canada 1613851874 AS6327 Shaw Communications Inc. 49.25 -123.1333

267 http://biomoby.org/services/wsdl/soaplab.icapture.ubc.ca/seqretDNAGCG82FASTA biordf.net 96.49.108.226 Canada 1613851874 AS6327 Shaw Communications Inc. 49.25 -123.1333

268 http://biomoby.org/services/wsdl/soaplab.icapture.ubc.ca/seqretDNARefFASTA2MEGA biordf.net 96.49.108.226 Canada 1613851874 AS6327 Shaw Communications Inc. 49.25 -123.1333

269 http://biomoby.org/services/wsdl/soaplab.icapture.ubc.ca/seqretDNARefFASTA2NEXUS biordf.net 96.49.108.226 Canada 1613851874 AS6327 Shaw Communications Inc. 49.25 -123.1333

270 http://biomoby.org/services/wsdl/soaplab.icapture.ubc.ca/seqretPROTEINRefFASTA2PHYLIP biordf.net 96.49.108.226 Canada 1613851874 AS6327 Shaw Communications Inc. 49.25 -123.1333

271 http://biomoby.org/services/wsdl/soaplab.icapture.ubc.ca/seqretPROTEINFASTA2EMBL biordf.net 96.49.108.226 Canada 1613851874 AS6327 Shaw Communications Inc. 49.25 -123.1333

272 http://biomoby.org/services/wsdl/soaplab.icapture.ubc.ca/seqretDNACODATA2FASTA biordf.net 96.49.108.226 Canada 1613851874 AS6327 Shaw Communications Inc. 49.25 -123.1333

273 http://biomoby.org/services/wsdl/bioinfo.icapture.ubc.ca/BIND_IdSearchGetGOTermsByPirId biordf.net 96.49.108.226 Canada 1613851874 AS6327 Shaw Communications Inc. 49.25 -123.1333

274 http://biomoby.org/services/wsdl/soaplab.icapture.ubc.ca/seqretPROTEINRefGEN2FASTA biordf.net 96.49.108.226 Canada 1613851874 AS6327 Shaw Communications Inc. 49.25 -123.1333

275 http://biomoby.org/services/wsdl/soaplab.icapture.ubc.ca/seqretPROTEINRefFASTA2NBRF biordf.net 96.49.108.226 Canada 1613851874 AS6327 Shaw Communications Inc. 49.25 -123.1333

276 http://biomoby.org/services/wsdl/soaplab.icapture.ubc.ca/seqretDNARefMSF2FASTA biordf.net 96.49.108.226 Canada 1613851874 AS6327 Shaw Communications Inc. 49.25 -123.1333

277 http://biomoby.org/services/wsdl/bioinfo.icapture.ubc.ca/BIND_IdSearchGetFastaByTairId biordf.net 96.49.108.226 Canada 1613851874 AS6327 Shaw Communications Inc. 49.25 -123.1333

278 http://biomoby.org/services/wsdl/soaplab.icapture.ubc.ca/seqretPROTEINRefNCBI2FASTA biordf.net 96.49.108.226 Canada 1613851874 AS6327 Shaw Communications Inc. 49.25 -123.1333

279 http://biomoby.org/services/wsdl/soaplab.icapture.ubc.ca/seqretDNANEXUS2FASTA biordf.net 96.49.108.226 Canada 1613851874 AS6327 Shaw Communications Inc. 49.25 -123.1333

280 http://biomoby.org/services/wsdl/soaplab.icapture.ubc.ca/seqretDNARefFASTA2IG biordf.net 96.49.108.226 Canada 1613851874 AS6327 Shaw Communications Inc. 49.25 -123.1333

281 http://biomoby.org/services/wsdl/soaplab.icapture.ubc.ca/seqretDNAFASTA2NBRF biordf.net 96.49.108.226 Canada 1613851874 AS6327 Shaw Communications Inc. 49.25 -123.1333

282 http://biomoby.org/services/wsdl/soaplab.icapture.ubc.ca/seqretPROTEINRefJACKNIFER2FASTA biordf.net 96.49.108.226 Canada 1613851874 AS6327 Shaw Communications Inc. 49.25 -123.1333

283 http://biomoby.org/services/wsdl/soaplab.icapture.ubc.ca/seqretDNARefGCG2FASTA biordf.net 96.49.108.226 Canada 1613851874 AS6327 Shaw Communications Inc. 49.25 -123.1333

284 http://biomoby.org/services/wsdl/soaplab.icapture.ubc.ca/seqretPROTEINJACKNIFERNON2FASTA biordf.net 96.49.108.226 Canada 1613851874 AS6327 Shaw Communications Inc. 49.25 -123.1333

285 http://biomoby.org/services/wsdl/soaplab.icapture.ubc.ca/seqretDNARefIG2FASTA biordf.net 96.49.108.226 Canada 1613851874 AS6327 Shaw Communications Inc. 49.25 -123.1333

286 http://biomoby.org/services/wsdl/soaplab.icapture.ubc.ca/seqretPROTEINFASTA2MSF biordf.net 96.49.108.226 Canada 1613851874 AS6327 Shaw Communications Inc. 49.25 -123.1333

287 http://biomoby.org/services/wsdl/soaplab.icapture.ubc.ca/seqretDNARefSWISS2FASTA biordf.net 96.49.108.226 Canada 1613851874 AS6327 Shaw Communications Inc. 49.25 -123.1333

288 http://biomoby.org/services/wsdl/soaplab.icapture.ubc.ca/seqretDNAFASTA2GCG biordf.net 96.49.108.226 Canada 1613851874 AS6327 Shaw Communications Inc. 49.25 -123.1333

289 http://biomoby.org/services/wsdl/soaplab.icapture.ubc.ca/seqretPROTEINRefFASTA2MSF biordf.net 96.49.108.226 Canada 1613851874 AS6327 Shaw Communications Inc. 49.25 -123.1333

290 http://biomoby.org/services/wsdl/soaplab.icapture.ubc.ca/seqretDNARefFASTA2CODATA biordf.net 96.49.108.226 Canada 1613851874 AS6327 Shaw Communications Inc. 49.25 -123.1333

291 http://biomoby.org/services/wsdl/soaplab.icapture.ubc.ca/seqretDNARefJACKNIFERNON2FASTA biordf.net 96.49.108.226 Canada 1613851874 AS6327 Shaw Communications Inc. 49.25 -123.1333

292 http://biomoby.org/services/wsdl/soaplab.icapture.ubc.ca/seqretPROTEINRefFASTA2JACKKNIFER biordf.net 96.49.108.226 Canada 1613851874 AS6327 Shaw Communications Inc. 49.25 -123.1333

293 http://biomoby.org/services/wsdl/bioinfo.icapture.ubc.ca/BIND_IdSearchGetGOTermsBySmartId biordf.net 96.49.108.226 Canada 1613851874 AS6327 Shaw Communications Inc. 49.25 -123.1333

294 http://biomoby.org/services/wsdl/soaplab.icapture.ubc.ca/revseqProteinFASTA2FASTA biordf.net 96.49.108.226 Canada 1613851874 AS6327 Shaw Communications Inc. 49.25 -123.1333

295 http://biomoby.org/services/wsdl/soaplab.icapture.ubc.ca/seqretPROTEINRefFASTA2GCG8 biordf.net 96.49.108.226 Canada 1613851874 AS6327 Shaw Communications Inc. 49.25 -123.1333

296 http://biomoby.org/services/wsdl/soaplab.icapture.ubc.ca/seqretDNAFASTA2RAW biordf.net 96.49.108.226 Canada 1613851874 AS6327 Shaw Communications Inc. 49.25 -123.1333

297 http://biomoby.org/services/wsdl/soaplab.icapture.ubc.ca/seqretPROTEINFASTA2SWISS biordf.net 96.49.108.226 Canada 1613851874 AS6327 Shaw Communications Inc. 49.25 -123.1333

298 http://biomoby.org/services/wsdl/antirrhinum.net/getDragonBlastText biordf.net 96.49.108.226 Canada 1613851874 AS6327 Shaw Communications Inc. 49.25 -123.1333

299 http://biomoby.org/services/wsdl/atidb.org/getInsertionsWithOffsetAsGFFByAGICode biordf.net 96.49.108.226 Canada 1613851874 AS6327 Shaw Communications Inc. 49.25 -123.1333

300 http://biomoby.org/services/wsdl/bioinfo.icapture.ubc.ca/snp2Frequencies biordf.net 96.49.108.226 Canada 1613851874 AS6327 Shaw Communications Inc. 49.25 -123.1333

301 http://biomoby.org/services/wsdl/bioinfo.icapture.ubc.ca/convertKeggGeneId2PDBId biordf.net 96.49.108.226 Canada 1613851874 AS6327 Shaw Communications Inc. 49.25 -123.1333

302 http://biomoby.org/services/wsdl/soaplab.icapture.ubc.ca/seqretPROTEINRefJACKNIFERNON2FASTA biordf.net null Canada 0 null null null

303 http://biomoby.org/services/wsdl/soaplab.icapture.ubc.ca/seqretPROTEINCODATA2FASTA biordf.net null Canada 0 null null null

304 http://biomoby.org/services/wsdl/soaplab.icapture.ubc.ca/seqretPROTEINGCG2FASTA biordf.net 96.49.108.226 Canada 1613851874 AS6327 Shaw Communications Inc. 49.25 -123.1333

305 http://biomoby.org/services/wsdl/soaplab.icapture.ubc.ca/seqretPROTEINTREECON2FASTA biordf.net 96.49.108.226 Canada 1613851874 AS6327 Shaw Communications Inc. 49.25 -123.1333

306 http://biomoby.org/services/wsdl/soaplab.icapture.ubc.ca/seqretDNANBRF2FASTA biordf.net 96.49.108.226 Canada 1613851874 AS6327 Shaw Communications Inc. 49.25 -123.1333

307 http://biomoby.org/services/wsdl/soaplab.icapture.ubc.ca/seqretDNARefFASTA2FASTA biordf.net 96.49.108.226 Canada 1613851874 AS6327 Shaw Communications Inc. 49.25 -123.1333

308 http://biomoby.org/services/wsdl/soaplab.icapture.ubc.ca/seqretDNAFASTA2CLUSTAL biordf.net 96.49.108.226 Canada 1613851874 AS6327 Shaw Communications Inc. 49.25 -123.1333

309 http://biomoby.org/services/wsdl/soaplab.icapture.ubc.ca/seqretDNARefFASTA2SWISS biordf.net 96.49.108.226 Canada 1613851874 AS6327 Shaw Communications Inc. 49.25 -123.1333

310 http://biomoby.org/services/wsdl/antirrhinum.net/DragonDB_TBLASTN biordf.net 96.49.108.226 Canada 1613851874 AS6327 Shaw Communications Inc. 49.25 -123.1333

311 http://biomoby.org/services/wsdl/soaplab.icapture.ubc.ca/seqretPROTEINRefSTRIDER2FASTA biordf.net 96.49.108.226 Canada 1613851874 AS6327 Shaw Communications Inc. 49.25 -123.1333

312 http://biomoby.org/services/wsdl/soaplab.icapture.ubc.ca/seqretPROTEINRefPHYLIP2FASTA biordf.net 96.49.108.226 Canada 1613851874 AS6327 Shaw Communications Inc. 49.25 -123.1333

313 http://biomoby.org/services/wsdl/soaplab.icapture.ubc.ca/seqretPROTEINFASTA2PIR biordf.net 96.49.108.226 Canada 1613851874 AS6327 Shaw Communications Inc. 49.25 -123.1333

314 http://biomoby.org/services/wsdl/soaplab.icapture.ubc.ca/seqretPROTEINFASTA2CODATA biordf.net 96.49.108.226 Canada 1613851874 AS6327 Shaw Communications Inc. 49.25 -123.1333

315 http://biomoby.org/services/wsdl/soaplab.icapture.ubc.ca/seqretPROTEINFASTA2TREECON biordf.net 96.49.108.226 Canada 1613851874 AS6327 Shaw Communications Inc. 49.25 -123.1333

316 http://biomoby.org/services/wsdl/soaplab.icapture.ubc.ca/seqretDNAFASTA2SWISS biordf.net 96.49.108.226 Canada 1613851874 AS6327 Shaw Communications Inc. 49.25 -123.1333

317 http://biomoby.org/services/wsdl/bioinfo.icapture.ubc.ca/BIND_IdSearchGetFastaByUniGeneId biordf.net 96.49.108.226 Canada 1613851874 AS6327 Shaw Communications Inc. 49.25 -123.1333

318 http://biomoby.org/services/wsdl/bioinfo.icapture.ubc.ca/BIND_IdSearchGetGOTermsByEmblId biordf.net 96.49.108.226 Canada 1613851874 AS6327 Shaw Communications Inc. 49.25 -123.1333

319 http://biomoby.org/services/wsdl/bioinfo.icapture.ubc.ca/BIND_IdSearchGetGOTermsByGi biordf.net 96.49.108.226 Canada 1613851874 AS6327 Shaw Communications Inc. 49.25 -123.1333

320 http://biomoby.org/services/wsdl/bioinfo.icapture.ubc.ca/BIND_IdSearchGetGOTermsByPfamId biordf.net 96.49.108.226 Canada 1613851874 AS6327 Shaw Communications Inc. 49.25 -123.1333

321 http://biomoby.org/services/wsdl/bioinfo.icapture.ubc.ca/BIND_IdSearchGetGOTermsByTrEmblId biordf.net 96.49.108.226 Canada 1613851874 AS6327 Shaw Communications Inc. 49.25 -123.1333

322 http://biomoby.org/services/wsdl/soaplab.icapture.ubc.ca/seqretDNAPHYLIP2FASTA biordf.net 96.49.108.226 Canada 1613851874 AS6327 Shaw Communications Inc. 49.25 -123.1333

323 http://biomoby.org/services/wsdl/soaplab.icapture.ubc.ca/seqretDNAJACKNIFERNON2FASTA biordf.net 96.49.108.226 Canada 1613851874 AS6327 Shaw Communications Inc. 49.25 -123.1333

324 http://biomoby.org/services/wsdl/soaplab.icapture.ubc.ca/seqretDNARefPIR2FASTA biordf.net 96.49.108.226 Canada 1613851874 AS6327 Shaw Communications Inc. 49.25 -123.1333

325 http://biomoby.org/services/wsdl/atidb.org/getGOAccsByAGICode biordf.net 96.49.108.226 Canada 1613851874 AS6327 Shaw Communications Inc. 49.25 -123.1333

326 http://biomoby.org/services/wsdl/soaplab.icapture.ubc.ca/seqretPROTEINIG2FASTA biordf.net 96.49.108.226 Canada 1613851874 AS6327 Shaw Communications Inc. 49.25 -123.1333

327 http://biomoby.org/services/wsdl/soaplab.icapture.ubc.ca/seqretPROTEINRefACEDB2FASTA biordf.net 96.49.108.226 Canada 1613851874 AS6327 Shaw Communications Inc. 49.25 -123.1333

328 http://biomoby.org/services/wsdl/soaplab.icapture.ubc.ca/seqretDNATEXT2FASTA biordf.net 96.49.108.226 Canada 1613851874 AS6327 Shaw Communications Inc. 49.25 -123.1333

329 http://biomoby.org/services/wsdl/soaplab.icapture.ubc.ca/seqretPROTEINMEGA2FASTA biordf.net 96.49.108.226 Canada 1613851874 AS6327 Shaw Communications Inc. 49.25 -123.1333

330 http://biomoby.org/services/wsdl/soaplab.icapture.ubc.ca/seqretDNARefTREECON2FASTA biordf.net 96.49.108.226 Canada 1613851874 AS6327 Shaw Communications Inc. 49.25 -123.1333

331 http://biomoby.org/services/wsdl/soaplab.icapture.ubc.ca/seqretDNANCBI2FASTA biordf.net 96.49.108.226 Canada 1613851874 AS6327 Shaw Communications Inc. 49.25 -123.1333

332 http://biomoby.org/services/wsdl/soaplab.icapture.ubc.ca/seqretDNAGEN2FASTA biordf.net 96.49.108.226 Canada 1613851874 AS6327 Shaw Communications Inc. 49.25 -123.1333

333 http://biomoby.org/services/wsdl/soaplab.icapture.ubc.ca/seqretPROTEINRefEMBL2FASTA biordf.net 96.49.108.226 Canada 1613851874 AS6327 Shaw Communications Inc. 49.25 -123.1333

334 http://biomoby.org/services/wsdl/soaplab.icapture.ubc.ca/seqretPROTEINRefCODATA2FASTA biordf.net 96.49.108.226 Canada 1613851874 AS6327 Shaw Communications Inc. 49.25 -123.1333

335 http://biomoby.org/services/wsdl/soaplab.icapture.ubc.ca/seqretPROTEINRefFASTA2NEXUS biordf.net 96.49.108.226 Canada 1613851874 AS6327 Shaw Communications Inc. 49.25 -123.1333

336 http://biomoby.org/services/wsdl/soaplab.icapture.ubc.ca/seqretDNAFASTA2STADEN biordf.net 96.49.108.226 Canada 1613851874 AS6327 Shaw Communications Inc. 49.25 -123.1333

337 http://biomoby.org/services/wsdl/atidb.org/getTranscriptCodesByAGICode biordf.net 96.49.108.226 Canada 1613851874 AS6327 Shaw Communications Inc. 49.25 -123.1333

338 http://biomoby.org/services/wsdl/soaplab.icapture.ubc.ca/seqretPROTEINRefMEGA2FASTA biordf.net 96.49.108.226 Canada 1613851874 AS6327 Shaw Communications Inc. 49.25 -123.1333

339 http://biomoby.org/services/wsdl/soaplab.icapture.ubc.ca/seqretPROTEINRefFASTA2MEGA biordf.net 96.49.108.226 Canada 1613851874 AS6327 Shaw Communications Inc. 49.25 -123.1333

340 http://biomoby.org/services/wsdl/soaplab.icapture.ubc.ca/seqretPROTEINRefFASTA2GCG biordf.net 96.49.108.226 Canada 1613851874 AS6327 Shaw Communications Inc. 49.25 -123.1333

341 http://biomoby.org/services/wsdl/soaplab.icapture.ubc.ca/seqretPROTEINRefFASTA2TREECON biordf.net 96.49.108.226 Canada 1613851874 AS6327 Shaw Communications Inc. 49.25 -123.1333

342 http://biomoby.org/services/wsdl/soaplab.icapture.ubc.ca/seqretDNARefFASTA2PHYLIP biordf.net 96.49.108.226 Canada 1613851874 AS6327 Shaw Communications Inc. 49.25 -123.1333

343 http://biomoby.org/services/wsdl/soaplab.icapture.ubc.ca/seqretPROTEINFASTA2STRIDER biordf.net 96.49.108.226 Canada 1613851874 AS6327 Shaw Communications Inc. 49.25 -123.1333

344 http://biomoby.org/services/wsdl/soaplab.icapture.ubc.ca/seqretDNAMEGA2FASTA biordf.net 96.49.108.226 Canada 1613851874 AS6327 Shaw Communications Inc. 49.25 -123.1333

345 http://biomoby.org/services/wsdl/soaplab.icapture.ubc.ca/seqretPROTEINFASTA2TEXT biordf.net 96.49.108.226 Canada 1613851874 AS6327 Shaw Communications Inc. 49.25 -123.1333

346 http://biomoby.org/services/wsdl/soaplab.icapture.ubc.ca/seqretPROTEINFASTA2RAW biordf.net 96.49.108.226 Canada 1613851874 AS6327 Shaw Communications Inc. 49.25 -123.1333

347 http://biomoby.org/services/wsdl/soaplab.icapture.ubc.ca/seqretPROTEINEMBL2FASTA biordf.net 96.49.108.226 Canada 1613851874 AS6327 Shaw Communications Inc. 49.25 -123.1333

348 http://biomoby.org/services/wsdl/soaplab.icapture.ubc.ca/seqretDNAFASTA2CODATA biordf.net 96.49.108.226 Canada 1613851874 AS6327 Shaw Communications Inc. 49.25 -123.1333

349 http://biomoby.org/services/wsdl/soaplab.icapture.ubc.ca/seqretPROTEINRefFASTA2CLUSTAL biordf.net 96.49.108.226 Canada 1613851874 AS6327 Shaw Communications Inc. 49.25 -123.1333

350 http://biomoby.org/services/wsdl/bioinfo.icapture.ubc.ca/getBase64SnpFrequencyImage biordf.net 96.49.108.226 Canada 1613851874 AS6327 Shaw Communications Inc. 49.25 -123.1333

351 http://biomoby.org/services/wsdl/soaplab.icapture.ubc.ca/seqretPROTEINRefFASTA2STADEN biordf.net 96.49.108.226 Canada 1613851874 AS6327 Shaw Communications Inc. 49.25 -123.1333

352 http://biomoby.org/services/wsdl/soaplab.icapture.ubc.ca/seqretDNAEMBL2FASTA biordf.net 96.49.108.226 Canada 1613851874 AS6327 Shaw Communications Inc. 49.25 -123.1333

353 http://biomoby.org/services/wsdl/soaplab.icapture.ubc.ca/seqretDNAFASTA2PIR biordf.net 96.49.108.226 Canada 1613851874 AS6327 Shaw Communications Inc. 49.25 -123.1333

354 http://biomoby.org/services/wsdl/soaplab.icapture.ubc.ca/seqretDNAFASTA2JACKKNIFERNON biordf.net 96.49.108.226 Canada 1613851874 AS6327 Shaw Communications Inc. 49.25 -123.1333

355 http://biomoby.org/services/wsdl/soaplab.icapture.ubc.ca/seqretPROTEINRefRAW2FASTA biordf.net 96.49.108.226 Canada 1613851874 AS6327 Shaw Communications Inc. 49.25 -123.1333

356 http://biomoby.org/services/wsdl/soaplab.icapture.ubc.ca/seqretPROTEINRefFASTA2FASTA biordf.net 96.49.108.226 Canada 1613851874 AS6327 Shaw Communications Inc. 49.25 -123.1333

357 http://biomoby.org/services/wsdl/soaplab.icapture.ubc.ca/seqretPROTEINRefFASTA2IG biordf.net 96.49.108.226 Canada 1613851874 AS6327 Shaw Communications Inc. 49.25 -123.1333

358 http://biomoby.org/services/wsdl/soaplab.icapture.ubc.ca/seqretPROTEINRefFASTA2ACEDB biordf.net 96.49.108.226 Canada 1613851874 AS6327 Shaw Communications Inc. 49.25 -123.1333

359 http://biomoby.org/services/wsdl/soaplab.icapture.ubc.ca/seqretPROTEINFASTA2FASTA biordf.net 96.49.108.226 Canada 1613851874 AS6327 Shaw Communications Inc. 49.25 -123.1333

360 http://biomoby.org/services/wsdl/soaplab.icapture.ubc.ca/revseqProteinRefFASTA2FASTA biordf.net 96.49.108.226 Canada 1613851874 AS6327 Shaw Communications Inc. 49.25 -123.1333

361 http://biomoby.org/services/wsdl/soaplab.icapture.ubc.ca/seqretDNAMSF2FASTA biordf.net 96.49.108.226 Canada 1613851874 AS6327 Shaw Communications Inc. 49.25 -123.1333

362 http://biomoby.org/services/wsdl/soaplab.icapture.ubc.ca/seqretDNARefFASTA2GCG biordf.net 96.49.108.226 Canada 1613851874 AS6327 Shaw Communications Inc. 49.25 -123.1333

363 http://biomoby.org/services/wsdl/soaplab.icapture.ubc.ca/seqretPROTEINJACKNIFER2FASTA biordf.net 96.49.108.226 Canada 1613851874 AS6327 Shaw Communications Inc. 49.25 -123.1333

364 http://biomoby.org/services/wsdl/soaplab.icapture.ubc.ca/seqretDNARefFASTA2JACKKNIFER biordf.net 96.49.108.226 Canada 1613851874 AS6327 Shaw Communications Inc. 49.25 -123.1333

365 http://biomoby.org/services/wsdl/soaplab.icapture.ubc.ca/seqretDNARAW2FASTA biordf.net 96.49.108.226 Canada 1613851874 AS6327 Shaw Communications Inc. 49.25 -123.1333

366 http://biomoby.org/services/wsdl/soaplab.icapture.ubc.ca/seqretDNARefSTRIDER2FASTA biordf.net 96.49.108.226 Canada 1613851874 AS6327 Shaw Communications Inc. 49.25 -123.1333

367 http://biomoby.org/services/wsdl/soaplab.icapture.ubc.ca/seqretPROTEINNEXUSNON2FASTA biordf.net 96.49.108.226 Canada 1613851874 AS6327 Shaw Communications Inc. 49.25 -123.1333

368 http://biomoby.org/services/wsdl/soaplab.icapture.ubc.ca/seqretDNARefSTADEN2FASTA biordf.net 96.49.108.226 Canada 1613851874 AS6327 Shaw Communications Inc. 49.25 -123.1333

369 http://biomoby.org/services/wsdl/soaplab.icapture.ubc.ca/seqretDNARefFASTA2TEXT biordf.net 96.49.108.226 Canada 1613851874 AS6327 Shaw Communications Inc. 49.25 -123.1333

370 http://biomoby.org/services/wsdl/soaplab.icapture.ubc.ca/seqretDNARefCLUSTAL2FASTA biordf.net 96.49.108.226 Canada 1613851874 AS6327 Shaw Communications Inc. 49.25 -123.1333

371 http://biomoby.org/services/wsdl/soaplab.icapture.ubc.ca/seqretDNARefGCG82FASTA biordf.net 96.49.108.226 Canada 1613851874 AS6327 Shaw Communications Inc. 49.25 -123.1333

372 http://biomoby.org/services/wsdl/soaplab.icapture.ubc.ca/seqretDNARefTEXT2FASTA biordf.net 96.49.108.226 Canada 1613851874 AS6327 Shaw Communications Inc. 49.25 -123.1333

373 http://biomoby.org/services/wsdl/soaplab.icapture.ubc.ca/seqretPROTEINNEXUS2FASTA biordf.net 96.49.108.226 Canada 1613851874 AS6327 Shaw Communications Inc. 49.25 -123.1333

374 http://biomoby.org/services/wsdl/soaplab.icapture.ubc.ca/seqretPROTEINRAW2FASTA biordf.net 96.49.108.226 Canada 1613851874 AS6327 Shaw Communications Inc. 49.25 -123.1333

375 http://biomoby.org/services/wsdl/soaplab.icapture.ubc.ca/seqretPROTEINRefFASTA2NCBI biordf.net 96.49.108.226 Canada 1613851874 AS6327 Shaw Communications Inc. 49.25 -123.1333

376 http://biomoby.org/services/wsdl/soaplab.icapture.ubc.ca/seqretPROTEINRefFASTA2STRIDER biordf.net 96.49.108.226 Canada 1613851874 AS6327 Shaw Communications Inc. 49.25 -123.1333

377 http://biomoby.org/services/wsdl/soaplab.icapture.ubc.ca/seqretPROTEINRefFASTA2EMBL biordf.net 96.49.108.226 Canada 1613851874 AS6327 Shaw Communications Inc. 49.25 -123.1333

378 http://biomoby.org/services/wsdl/soaplab.icapture.ubc.ca/seqretDNASWISS2FASTA biordf.net 96.49.108.226 Canada 1613851874 AS6327 Shaw Communications Inc. 49.25 -123.1333

379 http://biomoby.org/services/wsdl/soaplab.icapture.ubc.ca/seqretDNACLUSTAL2FASTA biordf.net 96.49.108.226 Canada 1613851874 AS6327 Shaw Communications Inc. 49.25 -123.1333

380 http://biomoby.org/services/wsdl/soaplab.icapture.ubc.ca/seqretPROTEINPHYLIP2FASTA biordf.net 96.49.108.226 Canada 1613851874 AS6327 Shaw Communications Inc. 49.25 -123.1333

381 http://biomoby.org/services/wsdl/soaplab.icapture.ubc.ca/seqretDNARefRAW2FASTA biordf.net 96.49.108.226 Canada 1613851874 AS6327 Shaw Communications Inc. 49.25 -123.1333

382 http://biomoby.org/services/wsdl/soaplab.icapture.ubc.ca/seqretPROTEINRefFASTA2TEXT biordf.net 96.49.108.226 Canada 1613851874 AS6327 Shaw Communications Inc. 49.25 -123.1333

383 http://biomoby.org/services/wsdl/soaplab.icapture.ubc.ca/seqretPROTEINRefFASTA2RAW biordf.net 96.49.108.226 Canada 1613851874 AS6327 Shaw Communications Inc. 49.25 -123.1333

384 http://biomoby.org/services/wsdl/atidb.org/getInterproResultsForAGICode biordf.net 96.49.108.226 Canada 1613851874 AS6327 Shaw Communications Inc. 49.25 -123.1333

385 http://biomoby.org/services/wsdl/soaplab.icapture.ubc.ca/seqretPROTEINFASTA2JACKKNIFER biordf.net 96.49.108.226 Canada 1613851874 AS6327 Shaw Communications Inc. 49.25 -123.1333

386 http://biomoby.org/services/wsdl/soaplab.icapture.ubc.ca/seqretPROTEINGCG82FASTA biordf.net 96.49.108.226 Canada 1613851874 AS6327 Shaw Communications Inc. 49.25 -123.1333

387 http://biomoby.org/services/wsdl/soaplab.icapture.ubc.ca/seqretPROTEINRefTREECON2FASTA biordf.net 96.49.108.226 Canada 1613851874 AS6327 Shaw Communications Inc. 49.25 -123.1333

388 http://biomoby.org/services/wsdl/soaplab.icapture.ubc.ca/seqretPROTEINMSF2FASTA biordf.net 96.49.108.226 Canada 1613851874 AS6327 Shaw Communications Inc. 49.25 -123.1333

389 http://biomoby.org/services/wsdl/soaplab.icapture.ubc.ca/seqretDNAPIR2FASTA biordf.net 96.49.108.226 Canada 1613851874 AS6327 Shaw Communications Inc. 49.25 -123.1333

390 http://biomoby.org/services/wsdl/soaplab.icapture.ubc.ca/seqretDNARefNEXUS2FASTA biordf.net 96.49.108.226 Canada 1613851874 AS6327 Shaw Communications Inc. 49.25 -123.1333

391 http://biomoby.org/services/wsdl/soaplab.icapture.ubc.ca/seqretPROTEINRefFASTA2SWISS biordf.net 96.49.108.226 Canada 1613851874 AS6327 Shaw Communications Inc. 49.25 -123.1333

392 http://biomoby.org/services/wsdl/soaplab.icapture.ubc.ca/seqretPROTEINNBRF2FASTA biordf.net 96.49.108.226 Canada 1613851874 AS6327 Shaw Communications Inc. 49.25 -123.1333

393 http://biomoby.org/services/wsdl/soaplab.icapture.ubc.ca/seqretDNAFASTA2ACEDB biordf.net 96.49.108.226 Canada 1613851874 AS6327 Shaw Communications Inc. 49.25 -123.1333

394 http://biomoby.org/services/wsdl/soaplab.icapture.ubc.ca/seqretDNARefFASTA2PIR biordf.net 96.49.108.226 Canada 1613851874 AS6327 Shaw Communications Inc. 49.25 -123.1333

395 http://biomoby.org/services/wsdl/soaplab.icapture.ubc.ca/seqretDNAFASTA2PHYLIP biordf.net 96.49.108.226 Canada 1613851874 AS6327 Shaw Communications Inc. 49.25 -123.1333

396 http://biomoby.org/services/wsdl/atidb.org/getInsertionsAsGFFByAGICode biordf.net 96.49.108.226 Canada 1613851874 AS6327 Shaw Communications Inc. 49.25 -123.1333

397 http://biomoby.org/services/wsdl/soaplab.icapture.ubc.ca/seqretPROTEINSWISS2FASTA biordf.net 96.49.108.226 Canada 1613851874 AS6327 Shaw Communications Inc. 49.25 -123.1333

398 http://biomoby.org/services/wsdl/soaplab.icapture.ubc.ca/seqretDNARefNCBI2FASTA biordf.net 96.49.108.226 Canada 1613851874 AS6327 Shaw Communications Inc. 49.25 -123.1333

399 http://biomoby.org/services/wsdl/soaplab.icapture.ubc.ca/seqretPROTEINFASTA2ACEDB biordf.net 96.49.108.226 Canada 1613851874 AS6327 Shaw Communications Inc. 49.25 -123.1333

400 http://biomoby.org/services/wsdl/soaplab.icapture.ubc.ca/seqretDNAACEDB2FASTA biordf.net 96.49.108.226 Canada 1613851874 AS6327 Shaw Communications Inc. 49.25 -123.1333

401 http://biomoby.org/services/wsdl/bioinfo.icapture.ubc.ca/BIND_IdSearchGetFastaBySwissProtId biordf.net 96.49.108.226 Canada 1613851874 AS6327 Shaw Communications Inc. 49.25 -123.1333

402 http://biomoby.org/services/wsdl/bioinfo.icapture.ubc.ca/BIND_IdSearchGetGOTermsByTairId biordf.net 96.49.108.226 Canada 1613851874 AS6327 Shaw Communications Inc. 49.25 -123.1333

403 http://biomoby.org/services/wsdl/bioinfo.icapture.ubc.ca/BIND_IdSearchGetGOTermsByPubMedId biordf.net 96.49.108.226 Canada 1613851874 AS6327 Shaw Communications Inc. 49.25 -123.1333

404 http://biomoby.org/services/wsdl/bioinfo.icapture.ubc.ca/BIND_IdSearchGetGOTermsByCog biordf.net 96.49.108.226 Canada 1613851874 AS6327 Shaw Communications Inc. 49.25 -123.1333

405 http://biomoby.org/services/wsdl/soaplab.icapture.ubc.ca/seqretDNAFASTA2JACKKNIFER biordf.net 96.49.108.226 Canada 1613851874 AS6327 Shaw Communications Inc. 49.25 -123.1333

406 http://biomoby.org/services/wsdl/soaplab.icapture.ubc.ca/seqretDNAGCG2FASTA biordf.net 96.49.108.226 Canada 1613851874 AS6327 Shaw Communications Inc. 49.25 -123.1333

407 http://biomoby.org/services/wsdl/soaplab.icapture.ubc.ca/seqretDNAJACKNIFER2FASTA biordf.net 96.49.108.226 Canada 1613851874 AS6327 Shaw Communications Inc. 49.25 -123.1333

408 http://biomoby.org/services/wsdl/bioinfo.icapture.ubc.ca/BIND_IdSearchGetFastaByPfamId biordf.net 96.49.108.226 Canada 1613851874 AS6327 Shaw Communications Inc. 49.25 -123.1333

409 http://biomoby.org/services/wsdl/soaplab.icapture.ubc.ca/seqretPROTEINFASTA2STADEN biordf.net null Canada 0 null null null

410 http://biomoby.org/services/wsdl/bioinfo.icapture.ubc.ca/BIND_IdSearchGetFastaByTrEmblId biordf.net 96.49.108.226 Canada 1613851874 AS6327 Shaw Communications Inc. 49.25 -123.1333

411 http://biomoby.org/services/wsdl/soaplab.icapture.ubc.ca/seqretPROTEINACEDB2FASTA biordf.net 96.49.108.226 Canada 1613851874 AS6327 Shaw Communications Inc. 49.25 -123.1333

412 http://biomoby.org/services/wsdl/bioinfo.icapture.ubc.ca/BIND_IdSearchGetGOTermsByWormBaseId biordf.net 96.49.108.226 Canada 1613851874 AS6327 Shaw Communications Inc. 49.25 -123.1333

413 http://biomoby.org/services/wsdl/bioinfo.icapture.ubc.ca/BIND_IdSearchGetFastaByEntrezGeneId biordf.net 96.49.108.226 Canada 1613851874 AS6327 Shaw Communications Inc. 49.25 -123.1333

414 http://biomoby.org/services/wsdl/soaplab.icapture.ubc.ca/seqretPROTEINRefMSF2FASTA biordf.net 96.49.108.226 Canada 1613851874 AS6327 Shaw Communications Inc. 49.25 -123.1333

415 http://biomoby.org/services/wsdl/soaplab.icapture.ubc.ca/seqretDNARefCODATA2FASTA biordf.net 96.49.108.226 Canada 1613851874 AS6327 Shaw Communications Inc. 49.25 -123.1333

416 http://biomoby.org/services/wsdl/bioinfo.icapture.ubc.ca/BIND_IdSearchGetGOTermsByUniProtId biordf.net 96.49.108.226 Canada 1613851874 AS6327 Shaw Communications Inc. 49.25 -123.1333

417 http://biomoby.org/services/wsdl/bioinfo.icapture.ubc.ca/BIND_IdSearchGetFastaByPubMedId biordf.net 96.49.108.226 Canada 1613851874 AS6327 Shaw Communications Inc. 49.25 -123.1333

418 http://biomoby.org/services/wsdl/bioinfo.icapture.ubc.ca/BIND_IdSearchGetGOTermsByUniGeneId biordf.net 96.49.108.226 Canada 1613851874 AS6327 Shaw Communications Inc. 49.25 -123.1333

419 http://biomoby.org/services/wsdl/soaplab.icapture.ubc.ca/seqretPROTEINFASTA2MEGA biordf.net 96.49.108.226 Canada 1613851874 AS6327 Shaw Communications Inc. 49.25 -123.1333

420 http://biomoby.org/services/wsdl/bioinfo.icapture.ubc.ca/BIND_IdSearchGetFastaByPirId biordf.net 96.49.108.226 Canada 1613851874 AS6327 Shaw Communications Inc. 49.25 -123.1333

421 http://biomoby.org/services/wsdl/soaplab.icapture.ubc.ca/seqretDNASTRIDER2FASTA biordf.net 96.49.108.226 Canada 1613851874 AS6327 Shaw Communications Inc. 49.25 -123.1333

422 http://biomoby.org/services/wsdl/soaplab.icapture.ubc.ca/seqretDNAFASTA2TREECON biordf.net 96.49.108.226 Canada 1613851874 AS6327 Shaw Communications Inc. 49.25 -123.1333

423 http://biomoby.org/services/wsdl/soaplab.icapture.ubc.ca/seqretDNAFASTA2MSF biordf.net 96.49.108.226 Canada 1613851874 AS6327 Shaw Communications Inc. 49.25 -123.1333

424 http://biomoby.org/services/wsdl/bioinfo.icapture.ubc.ca/BIND_IdSearchGetGOTermsBySwissProtId biordf.net 96.49.108.226 Canada 1613851874 AS6327 Shaw Communications Inc. 49.25 -123.1333

425 http://biomoby.org/services/wsdl/bioinfo.icapture.ubc.ca/BIND_IdSearchGetFastaByWormBaseId biordf.net null Canada 0 null null null

426 http://biomoby.org/services/wsdl/soaplab.icapture.ubc.ca/seqretDNAFASTA2GENBANK biordf.net 96.49.108.226 Canada 1613851874 AS6327 Shaw Communications Inc. 49.25 -123.1333

427 http://biomoby.org/services/wsdl/soaplab.icapture.ubc.ca/seqretDNAFASTA2FASTA biordf.net 96.49.108.226 Canada 1613851874 AS6327 Shaw Communications Inc. 49.25 -123.1333

428 http://biomoby.org/services/wsdl/soaplab.icapture.ubc.ca/seqretDNATREECON2FASTA biordf.net 96.49.108.226 Canada 1613851874 AS6327 Shaw Communications Inc. 49.25 -123.1333

429 http://biomoby.org/services/wsdl/bioinfo.icapture.ubc.ca/BIND_IdSearchGetGOTermsByEnsemblId biordf.net 96.49.108.226 Canada 1613851874 AS6327 Shaw Communications Inc. 49.25 -123.1333

430 http://biomoby.org/services/wsdl/bioinfo.icapture.ubc.ca/BIND_IdSearchGetFastaByCog biordf.net 96.49.108.226 Canada 1613851874 AS6327 Shaw Communications Inc. 49.25 -123.1333

431 http://biomoby.org/services/wsdl/soaplab.icapture.ubc.ca/seqretDNARefGEN2FASTA biordf.net 96.49.108.226 Canada 1613851874 AS6327 Shaw Communications Inc. 49.25 -123.1333

432 http://biomoby.org/services/wsdl/soaplab.icapture.ubc.ca/seqretDNARefPHYLIP2FASTA biordf.net 96.49.108.226 Canada 1613851874 AS6327 Shaw Communications Inc. 49.25 -123.1333

433 http://biomoby.org/services/wsdl/soaplab.icapture.ubc.ca/seqretPROTEINRefFASTA2GENBANK biordf.net 96.49.108.226 Canada 1613851874 AS6327 Shaw Communications Inc. 49.25 -123.1333

434 http://biomoby.org/services/wsdl/bioinfo.icapture.ubc.ca/BIND_IdSearchGetFastaByEmblId biordf.net 96.49.108.226 Canada 1613851874 AS6327 Shaw Communications Inc. 49.25 -123.1333

435 http://biomoby.org/services/wsdl/bioinfo.icapture.ubc.ca/BIND_IdSearchGetFastaByCdd biordf.net 96.49.108.226 Canada 1613851874 AS6327 Shaw Communications Inc. 49.25 -123.1333

436 http://biomoby.org/services/wsdl/bioinfo.icapture.ubc.ca/BIND_IdSearchGetFastaByGenbankId biordf.net 96.49.108.226 Canada 1613851874 AS6327 Shaw Communications Inc. 49.25 -123.1333

437 http://biomoby.org/services/wsdl/bioinfo.icapture.ubc.ca/BIND_IdSearchGetFastaByUniProtId biordf.net 96.49.108.226 Canada 1613851874 AS6327 Shaw Communications Inc. 49.25 -123.1333

438 http://biomoby.org/services/wsdl/soaplab.icapture.ubc.ca/seqretDNARefFASTA2STADEN biordf.net 96.49.108.226 Canada 1613851874 AS6327 Shaw Communications Inc. 49.25 -123.1333

439 http://biomoby.org/services/wsdl/soaplab.icapture.ubc.ca/seqretPROTEINSTRIDER2FASTA biordf.net 96.49.108.226 Canada 1613851874 AS6327 Shaw Communications Inc. 49.25 -123.1333

440 http://biomoby.org/services/wsdl/bioinfo.icapture.ubc.ca/BIND_IdSearchGetGOTermsByGenbankId biordf.net 96.49.108.226 Canada 1613851874 AS6327 Shaw Communications Inc. 49.25 -123.1333

441 http://biomoby.org/services/wsdl/bioinfo.icapture.ubc.ca/BIND_IdSearchGetFastaByOmimId biordf.net 96.49.108.226 Canada 1613851874 AS6327 Shaw Communications Inc. 49.25 -123.1333

442 http://biomoby.org/services/wsdl/soaplab.icapture.ubc.ca/seqretPROTEINRefPIR2FASTA biordf.net 96.49.108.226 Canada 1613851874 AS6327 Shaw Communications Inc. 49.25 -123.1333

443 http://biomoby.org/services/wsdl/bioinfo.icapture.ubc.ca/BIND_IdSearchGetGOTermsByEntrezGeneId biordf.net 96.49.108.226 Canada 1613851874 AS6327 Shaw Communications Inc. 49.25 -123.1333

444 http://biomoby.org/services/wsdl/bioinfo.icapture.ubc.ca/BIND_IdSearchGetFastaByGi biordf.net 96.49.108.226 Canada 1613851874 AS6327 Shaw Communications Inc. 49.25 -123.1333

445 http://biomoby.org/services/wsdl/bioinfo.icapture.ubc.ca/BIND_IdSearchGetGOTermsByCdd biordf.net 96.49.108.226 Canada 1613851874 AS6327 Shaw Communications Inc. 49.25 -123.1333

446 http://biomoby.org/services/wsdl/bioinfo.icapture.ubc.ca/PDB_id2RasMolScript biordf.net 96.49.108.226 Canada 1613851874 AS6327 Shaw Communications Inc. 49.25 -123.1333

447 http://biomoby.org/services/wsdl/icapture.ubc.ca/Gene2PubMed biordf.net 96.49.108.226 Canada 1613851874 AS6327 Shaw Communications Inc. 49.25 -123.1333

448 http://biomoby.org/services/wsdl/bioinfo.icapture.ubc.ca/convertSnp2EntrezGeneID biordf.net 96.49.108.226 Canada 1613851874 AS6327 Shaw Communications Inc. 49.25 -123.1333

449 http://biomoby.org/services/wsdl/bioinfo.icapture.ubc.ca/getGeneInformationByEntrezGeneID biordf.net 96.49.108.226 Canada 1613851874 AS6327 Shaw Communications Inc. 49.25 -123.1333

450 http://biomoby.org/services/wsdl/atidb.org/getAGICodesByGOAcc biordf.net 96.49.108.226 Canada 1613851874 AS6327 Shaw Communications Inc. 49.25 -123.1333

451 http://biomoby.org/services/wsdl/atidb.org/getInsertionNamesByAGICode biordf.net 96.49.108.226 Canada 1613851874 AS6327 Shaw Communications Inc. 49.25 -123.1333

452 http://biomoby.org/services/wsdl/atidb.org/getNASCCodeByATHNumber biordf.net 96.49.108.226 Canada 1613851874 AS6327 Shaw Communications Inc. 49.25 -123.1333

453 http://biomoby.org/services/wsdl/atidb.org/getPubmedIDsByAGICode biordf.net 96.49.108.226 Canada 1613851874 AS6327 Shaw Communications Inc. 49.25 -123.1333

454 http://biomoby.org/services/wsdl/soaplab.icapture.ubc.ca/seqretDNARefFASTA2ACEDB biordf.net 96.49.108.226 Canada 1613851874 AS6327 Shaw Communications Inc. 49.25 -123.1333

455 http://biomoby.org/services/wsdl/atidb.org/getSynonymsFromAGICode biordf.net 96.49.108.226 Canada 1613851874 AS6327 Shaw Communications Inc. 49.25 -123.1333

456 http://biomoby.org/services/wsdl/ualberta.ca/BacMapPNGMap ualberta.ca 129.128.98.86 Canada 2172674646 AS3359 University of Alberta 53.5248 -113.5334

457 http://biomoby.org/services/wsdl/ualberta.ca/getColiCardIDs_by_Cofactor ualberta.ca 129.128.98.86 Canada 2172674646 AS3359 University of Alberta 53.5248 -113.5334

458 http://biomoby.org/services/wsdl/ualberta.ca/getColiCardIDs_by_Cell_Location ualberta.ca 129.128.98.86 Canada 2172674646 AS3359 University of Alberta 53.5248 -113.5334

459 http://biomoby.org/services/wsdl/ualberta.ca/HMDBSequenceSearch ualberta.ca null Canada 0 null null null

460 http://biomoby.org/services/wsdl/ualberta.ca/BacMapGetRNAEncodingGeneSequences ualberta.ca 129.128.98.86 Canada 2172674646 AS3359 University of Alberta 53.5248 -113.5334

461 http://biomoby.org/services/wsdl/ualberta.ca/getColiCardIDs_by_GC_content ualberta.ca 129.128.98.86 Canada 2172674646 AS3359 University of Alberta 53.5248 -113.5334

462 http://biomoby.org/services/wsdl/ualberta.ca/BacMapGetGeneCard ualberta.ca 129.128.98.86 Canada 2172674646 AS3359 University of Alberta 53.5248 -113.5334

463 http://biomoby.org/services/wsdl/ualberta.ca/getColiCardIDs_by_PfamConservedDomainID ualberta.ca 129.128.98.86 Canada 2172674646 AS3359 University of Alberta 53.5248 -113.5334

464 http://biomoby.org/services/wsdl/ualberta.ca/DrugBankByCardAsText ualberta.ca 129.128.98.86 Canada 2172674646 AS3359 University of Alberta 53.5248 -113.5334

465 http://biomoby.org/services/wsdl/ualberta.ca/BacMapBlastSearch ualberta.ca 129.128.98.86 Canada 2172674646 AS3359 University of Alberta 53.5248 -113.5334

466 http://biomoby.org/services/wsdl/ualberta.ca/getColiCardIDs_by_QuaternaryStructure ualberta.ca 129.128.98.86 Canada 2172674646 AS3359 University of Alberta 53.5248 -113.5334

467 http://biomoby.org/services/wsdl/ualberta.ca/PlasMapperGenbankOutput ualberta.ca 129.128.98.86 Canada 2172674646 AS3359 University of Alberta 53.5248 -113.5334

468 http://biomoby.org/services/wsdl/ualberta.ca/DrugBankByCardAsHtml ualberta.ca 129.128.98.86 Canada 2172674646 AS3359 University of Alberta 53.5248 -113.5334

469 http://biomoby.org/services/wsdl/ualberta.ca/getColiCardIDs_by_SwissProtAcc ualberta.ca 129.128.98.86 Canada 2172674646 AS3359 University of Alberta 53.5248 -113.5334

470 http://biomoby.org/services/wsdl/ualberta.ca/getColiCardIDs_by_TranslatedMolecularWeight ualberta.ca 129.128.98.86 Canada 2172674646 AS3359 University of Alberta 53.5248 -113.5334

471 http://biomoby.org/services/wsdl/ualberta.ca/BacMapGetChromosomeSequence ualberta.ca 129.128.98.86 Canada 2172674646 AS3359 University of Alberta 53.5248 -113.5334

472 http://biomoby.org/services/wsdl/ualberta.ca/getColiCardIDs_by_MatureMolecularWeight ualberta.ca 129.128.98.86 Canada 2172674646 AS3359 University of Alberta 53.5248 -113.5334

473 http://biomoby.org/services/wsdl/ualberta.ca/getColiCardIDs_by_GeneName ualberta.ca 129.128.98.86 Canada 2172674646 AS3359 University of Alberta 53.5248 -113.5334

474 http://biomoby.org/services/wsdl/ualberta.ca/HMDBByCardAsText ualberta.ca 129.128.98.86 Canada 2172674646 AS3359 University of Alberta 53.5248 -113.5334

475 http://biomoby.org/services/wsdl/ualberta.ca/getColiCardIDs_by_EC_Number ualberta.ca 129.128.98.86 Canada 2172674646 AS3359 University of Alberta 53.5248 -113.5334

476 http://biomoby.org/services/wsdl/ualberta.ca/getColiCardIDs_by_ReactionProducts ualberta.ca 129.128.98.86 Canada 2172674646 AS3359 University of Alberta 53.5248 -113.5334

477 http://biomoby.org/services/wsdl/ualberta.ca/DrugBankBySequence ualberta.ca 129.128.98.86 Canada 2172674646 AS3359 University of Alberta 53.5248 -113.5334

478 http://biomoby.org/services/wsdl/ualberta.ca/BacMapHtmlMap ualberta.ca null Canada 0 null null null

479 http://biomoby.org/services/wsdl/ualberta.ca/BacMapGetProteinTable ualberta.ca 129.128.98.86 Canada 2172674646 AS3359 University of Alberta 53.5248 -113.5334

480 http://biomoby.org/services/wsdl/ualberta.ca/getColiCardIDs_by_Centisome_position ualberta.ca 129.128.98.86 Canada 2172674646 AS3359 University of Alberta 53.5248 -113.5334

481 http://biomoby.org/services/wsdl/ualberta.ca/getColiCardIDs_by_ReactionSubstrates ualberta.ca 129.128.98.86 Canada 2172674646 AS3359 University of Alberta 53.5248 -113.5334

482 http://biomoby.org/services/wsdl/ualberta.ca/getColiCardIDs_by_Translated_IsoelectricPoint ualberta.ca 129.128.98.86 Canada 2172674646 AS3359 University of Alberta 53.5248 -113.5334

483 http://biomoby.org/services/wsdl/ualberta.ca/Proteus2 ualberta.ca 129.128.98.86 Canada 2172674646 AS3359 University of Alberta 53.5248 -113.5334

484 http://biomoby.org/services/wsdl/ualberta.ca/getColiCardIDs_by_Mature_IsoelectricPoint ualberta.ca 129.128.98.86 Canada 2172674646 AS3359 University of Alberta 53.5248 -113.5334

485 http://biomoby.org/services/wsdl/ualberta.ca/getColiCardIDs_by_GenbankProtein ualberta.ca 129.128.98.86 Canada 2172674646 AS3359 University of Alberta 53.5248 -113.5334

486 http://biomoby.org/services/wsdl/ualberta.ca/getColiCardIDs_by_InteractingPartners ualberta.ca 129.128.98.86 Canada 2172674646 AS3359 University of Alberta 53.5248 -113.5334

487 http://biomoby.org/services/wsdl/ualberta.ca/BacMapTextSearch ualberta.ca 129.128.98.86 Canada 2172674646 AS3359 University of Alberta 53.5248 -113.5334

488 http://biomoby.org/services/wsdl/ualberta.ca/getColiCardIDs_by_Inhibitor ualberta.ca 129.128.98.86 Canada 2172674646 AS3359 University of Alberta 53.5248 -113.5334

489 http://biomoby.org/services/wsdl/ualberta.ca/getColiCardIDs_by_Homologous_Organism ualberta.ca 129.128.98.86 Canada 2172674646 AS3359 University of Alberta 53.5248 -113.5334

490 http://biomoby.org/services/wsdl/ualberta.ca/getColiCardIDs_by_Paralogues ualberta.ca 129.128.98.86 Canada 2172674646 AS3359 University of Alberta 53.5248 -113.5334

491 http://biomoby.org/services/wsdl/ualberta.ca/BacMapGetGenBankRecord ualberta.ca 129.128.98.86 Canada 2172674646 AS3359 University of Alberta 53.5248 -113.5334

492 http://biomoby.org/services/wsdl/ualberta.ca/getColiCardIDs_by_Operon_Components ualberta.ca 129.128.98.86 Canada 2172674646 AS3359 University of Alberta 53.5248 -113.5334

493 http://biomoby.org/services/wsdl/ualberta.ca/Proteus ualberta.ca 129.128.98.86 Canada 2172674646 AS3359 University of Alberta 53.5248 -113.5334

494 http://biomoby.org/services/wsdl/ualberta.ca/getColiCardIDs_by_PDBid ualberta.ca 129.128.98.86 Canada 2172674646 AS3359 University of Alberta 53.5248 -113.5334

495 http://biomoby.org/services/wsdl/ualberta.ca/PlasMapperTextMap ualberta.ca 129.128.98.86 Canada 2172674646 AS3359 University of Alberta 53.5248 -113.5334

496 http://biomoby.org/services/wsdl/ualberta.ca/getColiCardIDs_by_blattner_number ualberta.ca 129.128.98.86 Canada 2172674646 AS3359 University of Alberta 53.5248 -113.5334

497 http://biomoby.org/services/wsdl/ualberta.ca/BacMapGetSpeciesCard ualberta.ca 129.128.98.86 Canada 2172674646 AS3359 University of Alberta 53.5248 -113.5334

498 http://biomoby.org/services/wsdl/ualberta.ca/getColiCardIDs_by_Gi ualberta.ca 129.128.98.86 Canada 2172674646 AS3359 University of Alberta 53.5248 -113.5334

499 http://biomoby.org/services/wsdl/ualberta.ca/getColiCardIDsByKeyword ualberta.ca 129.128.98.86 Canada 2172674646 AS3359 University of Alberta 53.5248 -113.5334

500 http://biomoby.org/services/wsdl/ualberta.ca/DrugBankByName ualberta.ca 129.128.98.86 Canada 2172674646 AS3359 University of Alberta 53.5248 -113.5334

501 http://biomoby.org/services/wsdl/ualberta.ca/getColiCardIDs_by_SpecificFunction ualberta.ca 129.128.98.86 Canada 2172674646 AS3359 University of Alberta 53.5248 -113.5334

502 http://biomoby.org/services/wsdl/ualberta.ca/BacMapGetProteinEncodingGeneSequences ualberta.ca 129.128.98.86 Canada 2172674646 AS3359 University of Alberta 53.5248 -113.5334

503 http://biomoby.org/services/wsdl/ualberta.ca/BacMapGetProteinSequences ualberta.ca 129.128.98.86 Canada 2172674646 AS3359 University of Alberta 53.5248 -113.5334

504 http://biomoby.org/services/wsdl/ualberta.ca/getRNACardIDs_by_GeneName ualberta.ca 129.128.98.86 Canada 2172674646 AS3359 University of Alberta 53.5248 -113.5334

505 http://biomoby.org/services/wsdl/bioinfo.icapture.ubc.ca/GeneMarkHMM_Arabidopsis ucalgary.ca null Canada 0 null null null

506 http://www.nhcgov.com/_vti_bin/BusinessDataCatalog.asmx?wsdl nhcgov.com 152.31.192.25 United States 2552217625 AS81 NCREN - MCNC 34.2432 -78.0996

507 http://etax.nhcgov.com/Help/Services/HelpService.asmx?WSDL nhcgov.com 152.31.192.24 Canada 2552217624 AS81 NCREN - MCNC 34.2432 -78.0996

508 http://etax.nhcgov.com/Admin/Services/Login.asmx?WSDL nhcgov.com 152.31.192.25 Canada 2552217625 AS81 NCREN - MCNC 34.2432 -78.0996

509 http://www.nhcgov.com/_vti_bin/People.asmx?wsdl nhcgov.com 152.31.192.24 United States 2552217624 AS81 NCREN - MCNC 34.2432 -78.0996

510 http://www.nhcgov.com/_vti_bin/Authentication.asmx?wsdl nhcgov.com 152.31.192.25 United States 2552217625 AS81 NCREN - MCNC 34.2432 -78.0996

511 http://netpub.cstudies.ubc.ca/dotnet/flashgame/gameScores.asmx?wsdl ubc.ca 137.82.130.49 Canada 2303885873 AS271 BCnet 49.2765 -123.2177

512 http://netpub.cstudies.ubc.ca/dotnet/models/cs/webservices/HelloService.asmx?WSDL ubc.ca 137.82.130.49 Canada 2303885873 AS271 BCnet 49.2765 -123.2177

513 http://netpub.cstudies.ubc.ca/dotnet/WebServices/KlickageService.asmx?wsdl ubc.ca 137.82.130.49 Canada 2303885873 AS271 BCnet 49.2765 -123.2177

514 http://netpub.cstudies.ubc.ca/dotnet/webservices/chineseastrology.asmx?WSDL ubc.ca 137.82.130.49 Canada 2303885873 AS271 BCnet 49.2765 -123.2177

515 http://netpub.cstudies.ubc.ca/dotnet/WebServices/MovieService.asmx?wsdl ubc.ca 137.82.130.49 Canada 2303885873 AS271 BCnet 49.2765 -123.2177

516 http://netpub.cstudies.ubc.ca/dotnet/WebServices/WebService1.asmx?wsdl ubc.ca 137.82.130.49 Canada 2303885873 AS271 BCnet 49.2765 -123.2177

517 http://netpub.cstudies.ubc.ca/dotnet/WebServices/TimeService.asmx?wsdl ubc.ca 137.82.130.49 Canada 2303885873 AS271 BCnet 49.2765 -123.2177

518 http://netpub.cstudies.ubc.ca/ws01/wscs01/conversionService.asmx?WSDL ubc.ca 137.82.130.49 Canada 2303885873 AS271 BCnet 49.2765 -123.2177

519 http://netpub.cstudies.ubc.ca/dotnet/WebServices/FuelCalculator.asmx?wsdl ubc.ca 137.82.130.49 Canada 2303885873 AS271 BCnet 49.2765 -123.2177

520 http://netpub.cstudies.ubc.ca/dotnet/WebServices/Footer.asmx?wsdl ubc.ca 137.82.130.49 Canada 2303885873 AS271 BCnet 49.2765 -123.2177

521 http://netpub.cstudies.ubc.ca/dotnet/egCS01/WebServices/HelloServiceCS.asmx?WSDL ubc.ca 137.82.130.49 Canada 2303885873 AS271 BCnet 49.2765 -123.2177

522 http://netpub.cstudies.ubc.ca/dotnet/webservices/conversions.asmx?WSDL ubc.ca 137.82.130.49 Canada 2303885873 AS271 BCnet 49.2765 -123.2177

523 http://netpub.cstudies.ubc.ca/dotnet/WebServices/ServerUsageService.asmx?wsdl ubc.ca null Canada 0 null null null

524 http://netpub.cstudies.ubc.ca/dotnet/WebServices/FinancialFunctions.asmx?wsdl ubc.ca 137.82.130.49 Canada 2303885873 AS271 BCnet 49.2765 -123.2177

525 http://netpub.cstudies.ubc.ca/dotnet/WebServices/FinanceService.asmx?wsdl ubc.ca 137.82.130.49 Canada 2303885873 AS271 BCnet 49.2765 -123.2177

526 http://netpub.cstudies.ubc.ca/dotnet/WebServices/WeatherService.asmx?wsdl ubc.ca 137.82.130.49 Canada 2303885873 AS271 BCnet 49.2765 -123.2177

527 http://netpub.cstudies.ubc.ca/dotnet/WebServices/strings.asmx?wsdl ubc.ca 137.82.130.49 Canada 2303885873 AS271 BCnet 49.2765 -123.2177

528 http://biomoby.org/services/wsdl/bioinfo.icapture.ubc.ca/GeneticMap2GFF ubc.ca 137.82.130.49 Canada 2303885873 AS271 BCnet 49.2765 -123.2177

529 http://www.alphamosaik.com/_vti_bin/BusinessDataCatalog.asmx?wsdl alphamosaik.com 174.142.62.85 Canada 2928557653 AS32613 iWeb Technologies Inc. 45.5 -73.5833

530 http://www.alphamosaik.com/_vti_bin/SharepointEmailWS.asmx?wsdl alphamosaik.com 174.142.62.85 Canada 2928557653 AS32613 iWeb Technologies Inc. 45.5 -73.5833

531 http://www.alphamosaik.com/_vti_bin/Authentication.asmx?wsdl alphamosaik.com 174.142.62.85 Canada 2928557653 AS32613 iWeb Technologies Inc. 45.5 -73.5833

532 http://www.alphamosaik.com/_vti_bin/People.asmx?wsdl alphamosaik.com 174.142.62.85 Canada 2928557653 AS32613 iWeb Technologies Inc. 45.5 -73.5833

533 http://demos5.dundas.com/_vti_bin/BusinessDataCatalog.asmx?wsdl dundas.com 207.219.70.14 Canada 3487254030 AS852 Telus Advanced Communications 43.6667 -79.4167

534 http://demos5.dundas.com/_vti_bin/People.asmx?wsdl dundas.com 207.219.70.14 Canada 3487254030 AS852 Telus Advanced Communications 43.6667 -79.4167

535 http://demos5.dundas.com/_vti_bin/Authentication.asmx?wsdl dundas.com 207.219.70.14 Canada 3487254030 AS852 Telus Advanced Communications 43.6667 -79.4167

536 http://www.envisionit.com/_vti_bin/BusinessDataCatalog.asmx?wsdl envisionit.com 216.13.141.34 Canada 3624766754 AS15290 Allstream Corp. 43.6 -79.65

537 http://www.envisionit.com/_vti_bin/People.asmx?wsdl envisionit.com 216.13.141.34 Canada 3624766754 AS15290 Allstream Corp. 43.6 -79.65

538 http://www.envisionit.com/_vti_bin/Authentication.asmx?wsdl envisionit.com 216.13.141.34 Canada 3624766754 AS15290 Allstream Corp. 43.6 -79.65

539 http://www.etfo.ca/_vti_bin/BusinessDataCatalog.asmx?wsdl etfo.ca 69.46.105.70 Canada 1160669510 AS36031 Q9 Networks Inc. 43.6469 -79.3823

540 http://www.etfo.ca/_vti_bin/People.asmx?wsdl etfo.ca 69.46.105.70 Canada 1160669510 AS36031 Q9 Networks Inc. 43.6469 -79.3823

541 http://www.etfo.ca/_vti_bin/Authentication.asmx?wsdl etfo.ca 69.46.105.70 Canada 1160669510 AS36031 Q9 Networks Inc. 43.6469 -79.3823

542 http://home.infusionblogs.com/_vti_bin/BusinessDataCatalog.asmx?wsdl infusionblogs.com 66.241.131.32 Canada 1123124000 AS23498 Cogeco Data Services Inc. 43.6667 -79.4167

543 http://home.infusionblogs.com/_vti_bin/People.asmx?wsdl infusionblogs.com 66.241.131.32 Canada 1123124000 AS23498 Cogeco Data Services Inc. 43.6667 -79.4167

544 http://sp.sd76.ab.ca/_vti_bin/BusinessDataCatalog.asmx?wsdl sd76.ab.ca 199.216.215.16 Canada 3352876816 AS6327 Shaw Communications Inc. 53.5421 -113.4989

545 http://sp.sd76.ab.ca/_vti_bin/People.asmx?wsdl sd76.ab.ca 199.216.215.16 Canada 3352876816 AS6327 Shaw Communications Inc. 53.5421 -113.4989

546 http://sp.sd76.ab.ca/_vti_bin/Authentication.asmx?wsdl sd76.ab.ca 199.216.215.16 Canada 3352876816 AS6327 Shaw Communications Inc. 53.5421 -113.4989

547 http://cisa.ca/_vti_bin/SharepointEmailWS.asmx?wsdl cisa.ca 69.10.128.156 Canada 1158316188 AS19875 IPWorld Networks 49.8803 -119.5004

548 http://ws.cisa.ca/WehireWS/JobsWs.asmx?wsdl cisa.ca 69.10.128.156 Canada 1158316188 AS19875 IPWorld Networks 49.8803 -119.5004

549 http://cisa.ca/_vti_bin/People.asmx?wsdl cisa.ca 69.10.128.156 Canada 1158316188 AS19875 IPWorld Networks 49.8803 -119.5004

550 http://cisa.ca/_vti_bin/Authentication.asmx?wsdl cisa.ca 69.10.128.156 Canada 1158316188 AS19875 IPWorld Networks 49.8803 -119.5004

551 http://support.colligo.com/_vti_bin/People.asmx?wsdl colligo.com 50.56.114.112 Canada 842560112 null 29.4889 -98.3987

552 http://support.colligo.com/_vti_bin/Authentication.asmx?wsdl colligo.com 50.56.114.112 Canada 842560112 null 29.4889 -98.3987

553 http://aquatic.pyr.ec.gc.ca/wqwebservices/waterquality.asmx?WSDL gc.ca 205.193.117.158 Canada 3452007838 AS2669 Government Telecommunications and Informatics Services 45.4207 -75.7023

554 http://www.cobbtax.org/Help/Services/HelpService.asmx?WSDL cobbtax.org 199.15.172.159 Canada 3339693215 AS15085 Immedion, LLC 34.8004 -82.3221

555 http://www.cobbtax.org/Reports/Services/RptServices.asmx?WSDL cobbtax.org 199.15.172.159 Canada 3339693215 AS15085 Immedion, LLC 34.8004 -82.3221

556 http://www.nsassessmentonline.ca/Admin/Services/Login.asmx?WSDL nsassessmentonline.ca null Canada 0 null null null

557 http://www.nsassessmentonline.ca/Help/Services/HelpService.asmx?WSDL nsassessmentonline.ca null Canada 0 null null null

558 http://www.admtl.com/UserControls/VEControls/Localisateur.asmx?WSDL admtl.com 64.26.178.213 Canada 1075491541 AS7788 Magma Communications Ltd. 45.4167 -75.7

559 http://m43.ensemsys.com/jboss-net/services/Version?wsdl ensemsys.com 208.66.252.229 Canada 3494051045 AS14642 Morewave Communication Inc. 49.1667 -122.9667

560 http://m43.ensemsys.com/jboss-net/services/PDFCollaboration?wsdl ensemsys.com 208.66.252.229 Canada 3494051045 AS14642 Morewave Communication Inc. 49.1667 -122.9667

561 http://netservice.fossware.com/Articles.asmx?wsdl fossware.com 23.23.249.237 Canada 387447277 null 39.0437 -77.4875

562 http://netservice.fossware.com/ArticlesSecure.asmx?WSDL fossware.com 23.23.249.237 Canada 387447277 null 39.0437 -77.4875

563 http://netservice.fossware.com/Forum.asmx?wsdl fossware.com 23.23.249.237 Canada 387447277 null 39.0437 -77.4875

564 http://netservice.fossware.com/ForumSecure.asmx?wsdl fossware.com 23.23.249.237 Canada 387447277 null 39.0437 -77.4875

565 http://lightswitch2.irc.nrc.ca/lightswitch/webservice/web_api.php?wsdl nrc.ca 132.246.11.80 Canada 2230717264 AS25689 National Research Council of Canada 45.4207 -75.7023

566 http://www.domainsbot.com/help/submission.asmx?WSDL domainsbot.com 64.40.101.127 Canada 1076389247 AS14280 NetNation Communications Inc 49.2857 -123.1142

567 http://www.domainsbot.com/LiveService.asmx?WSDL domainsbot.com 64.40.101.127 Canada 1076389247 AS14280 NetNation Communications Inc 49.2857 -123.1142

568 http://www.optimizationservices.org/wsdl/OScL.wsdl optimizationservices.org 108.60.15.23 Canada 1815875351 null 49.2591 -123.0226

569 http://www.comriesoftware.net/WSCart/cart.asmx?WSDL comriesoftware.net 184.70.74.158 Canada 3091614366 AS6327 Shaw Communications Inc. 53.6199 -113.6377

570 http://www.comriesoftware.net/cshitcounter/hitcounter.asmx?WSDL comriesoftware.net 184.70.74.158 Canada 3091614366 AS6327 Shaw Communications Inc. 53.6199 -113.6377

571 http://icare.fairfaxcounty.gov/Help/Services/HelpService.asmx?WSDL fairfaxcounty.gov 166.94.9.135 Canada 2791180679 AS3926 Fairfax County Dept of Information Technology 38.8557 -77.3616

572 http://servicesclients.rdprm.gouv.qc.ca/Diagnostic/Service/WebServiceDiagnostic.asmx?WSDL gouv.qc.ca 142.213.160.134 Canada 2396364934 AS11489 Bell Canada 45.5 -73.5833

573 http://bmjupdates.mcmaster.ca/cews/ceservice.asmx?WSDL mcmaster.ca 130.113.64.65 Canada 2188460097 AS23237 McMaster University 43.2604 -79.8961

574 http://216.81.11.13/whiteboxeyes/validate.asmx?WSDL 216.81.11.13 216.81.11.13 Canada 3629189901 AS15290 Allstream Corp. 60 -95

575 http://www.astrogeek.org/services/SolarSystem.asmx?WSDL astrogeek.org null Canada 0 null null null

576 http://canflix.com/product/AddToCartService.asmx?WSDL canflix.com null Canada 0 null null null

577 http://www.championsway.net/OnlineService/CheckCCAvailability.asmx?WSDL championsway.net 204.244.9.199 Canada 3438545351 AS5071 WesTel Telecommunications 49.25 -123.1333

578 http://new.cineplex.com/Services/AjaxServices.asmx?WSDL cineplex.com 174.90.122.198 Canada 2925165254 AS603 Bell Canada 43.6667 -79.4167

579 http://ws.cirquedusoleil.com/LUXOR/LuxorService.asmx?wsdl cirquedusoleil.com 64.254.20.190 Canada 1090393278 AS18563 CGI Inc 45.5652 -73.6444

580 http://www.cruiseshipcenters.ca/search.asmx?WSDL cruiseshipcenters.ca 64.254.150.158 Canada 1090426526 AS16941 RoundHeaven Communications Canada Inc. 43.5892 -79.7239

581 http://www.kamloops.cruiseshipcenters.com/search.asmx-WSDL cruiseshipcenters.com 64.254.150.158 Canada 1090426526 AS16941 RoundHeaven Communications Canada Inc. 43.5892 -79.7239

582 http://cybertvpartner.com/tv/service.asmx?wsdl cybertvpartner.com 74.86.197.160 Canada 1247200672 AS36351 SoftLayer Technologies Inc. 32.9299 -96.8353

583 http://www.digitalphoto.ca/orderservice.asmx?wsdl digitalphoto.ca 209.251.177.155 United States 3522933147 AS23148 Terremark 32.8795 -96.9398

584 http://epresence.tv/webservices/submitservice.asmx?WSDL epresence.tv 184.73.252.245 United States 3091856629 AS14618 Amazon.com, Inc. 39.0437 -77.4875

585 http://equipementsgagnon.ca/ui/s_public/datas/getProjectImages.asmx?WSDL equipementsgagnon.ca 204.80.6.181 Canada 3427796661 AS16438 Satelcom Internet Inc. 46.034 -73.441

586 http://greg.froh.ca/fun/random_bushism/soap/?wsdl froh.ca 199.167.129.35 Canada 3349643555 null 45.4129 -75.6901

587 http://www.greenpoint.ca/update/ISAPI_LiveUpdate.dll/wsdl/IWSDLPublish greenpoint.ca 66.46.108.254 Canada 1110338814 AS15290 Allstream Corp. 53.55 -113.5

588 http://hcsbi.com/IntrNetAppDev/webService/service.asmx?WSDL hcsbi.com 8.27.163.71 Canada 136028999 AS3356 Level 3 Communications 33.4223 -111.8226

589 http://intool.ices.on.ca/MenuPopulating.asmx?WSDL ices.on.ca 199.212.114.10 Canada 3352588810 AS549 GTAnet Networking 43.7168 -79.3998

590 http://www.jardindeprovence.ca/WebServiceFlash.asmx?wsdl jardindeprovence.ca null Canada 0 null null null

591 http://www.news2news.com/vfp/wsdl/webservices.WSDL news2news.com 216.201.96.67 Canada 3637076035 AS18650 Korax Inc. 43.3479 -79.7593

592 http://www.protegez-vous.ca/WebServices/WebServiceCars.asmx?WSDL protegez-vous.ca 72.55.128.81 Canada 1211596881 AS32613 iWeb Technologies Inc. 45.5 -73.5833

593 http://www.saveabuck.com.au/Webservice/AutoComplete.asmx?WSDL saveabuck.com.au null Canada 0 null null null

594 http://www.saveagreenback.com/Webservice/AutoComplete.asmx?WSDL saveagreenback.com 50.63.202.42 Canada 843041322 null 33.6119 -111.8906

595 http://www.saveapound.co.uk/Webservice/AutoComplete.asmx?WSDL saveapound.co.uk 64.46.36.87 Canada 1076765783 AS19875 IPWorld Networks 49.8803 -119.5004

596 http://demo.savian.net/SasWebServicesDemo/Service.asmx?WSDL savian.net 75.71.171.12 Canada 1262988044 AS33652 Comcast Cable Communications, Inc. 38.8339 -104.8214

597 http://softticket.net/ActivationServer.asmx?WSDL softticket.net 69.70.6.251 Canada 1162217211 AS5769 Videotron Telecom Ltee 51.4667 -57.7167

598 http://www.streetperfect.net/webservice/streetperfect.asmx?wsdl streetperfect.net 216.13.141.34 Canada 3624766754 AS15290 Allstream Corp. 43.6 -79.65

599 http://www1.ccni.cl/wsRegistroLog/ws/services/wsRegistroLog?wsdl ccni.cl null Chile 0 null null null

600 http://www1.ccni.cl/WS_CIPP/services/WsCipp/wsdl/WsCipp.wsdl ccni.cl null Chile 0 null null null

601 http://www7.ccni.cl/inlandTariff/ws/services/wsInlandTariff?wsdl ccni.cl null Chile 0 null null null

602 http://www1.ccni.cl/adminCCNINetwork/ws/services/wsCCNINetwork?wsdl ccni.cl null Chile 0 null null null

603 http://www1.ccni.cl/CCNI_WAR/services/Itinerario/wsdl/Itinerario.wsdl ccni.cl null Chile 0 null null null

604 http://www7.ccni.cl/inlandTariff/ws/services/Version?wsdl ccni.cl null Chile 0 null null null

605 http://www1.ccni.cl/wsGetFolioControl/ws/services/wsGetFolioControl?wsdl ccni.cl null Chile 0 null null null

606 http://www7.ccni.cl/inlandTariff/ws/EchoHeaders.jws?wsdl ccni.cl null Chile 0 null null null

607 http://ws.databusiness.cl/ws_frmes.asmx?WSDL databusiness.cl null Chile 0 null null null

608 http://ws.databusiness.cl/ws_frmsys.asmx?WSDL databusiness.cl null Chile 0 null null null

609 http://ws.databusiness.cl/ws_frmsy2.asmx?WSDL databusiness.cl null Chile 0 null null null

610 http://ws.databusiness.cl/ws_bic.asmx?WSDL databusiness.cl null Chile 0 null null null

611 http://www.signature.cl/sign@webservices/Core.asmx?WSDL signature.cl 200.6.96.5 Chile 3355860997 AS27659 INGENIERIA LTDA. -33.45 -70.6667

612 http://www.vtr.cl/ws/ruu.php?wsdl vtr.cl 200.83.4.60 Chile 3360883772 AS22047 BANDA ANCHA S.A. -33.45 -70.6667

613 http://www.vtr.cl/ws/mtvs.php?wsdl vtr.cl 200.83.4.60 Chile 3360883772 AS22047 BANDA ANCHA S.A. -33.45 -70.6667

614 http://200.27.220.54/WSSNA/tps.asmx?WSDL 200.27.220.54 200.27.220.54 Chile 3357269046 AS6429 Telmex Chile Internet S.A. -33.45 -70.6667

615 http://200.72.242.111:9080/penta-ws/services/SoapManager/wsdl/SoapManager.wsdl 200.72.242.111 200.72.242.111 Chile 3360223855 AS6471 CHILE S.A. -33.45 -70.6667

616 http://201.238.215.90/STGSOPws/MDisp.asmx?WSDL 201.238.215.90 201.238.215.90 Chile 3387873114 AS14259 Gtd Internet S.A. -33.45 -70.6667

617 http://rodeo.lemontech.cl/rodeo/app/webservices.php?wsdl lemontech.cl 201.238.192.115 Chile 3387867251 AS14259 Gtd Internet S.A. -33.45 -70.6667

618 http://www.liberty.cl/ColemontFusionWS/wsdl/cl/liberty/cvws/WS/StringWSColemontFusionService.wsdl liberty.cl null Chile 0 null null null

619 http://www.softdata.cl/wslme/lme.asmx?WSDL softdata.cl 200.54.151.132 Chile 3359020932 AS16629 CTC. CORP S.A. (TELEFONICA EMPRESAS) -36.8333 -73.05

620 http://www.pulmonary-rehabilitation.com.cn/WebService/Cme.asmx?WSDL pulmonary-rehabilitation.com.cn null China 0 null null null

621 http://www.pulmonary-rehabilitation.com.cn/WebService/Friend.asmx?WSDL pulmonary-rehabilitation.com.cn null China 0 null null null

622 http://www.pulmonary-rehabilitation.com.cn/WebService/Feed.asmx?WSDL pulmonary-rehabilitation.com.cn null China 0 null null null

623 http://www.pulmonary-rehabilitation.com.cn/WebService/Express.asmx?WSDL pulmonary-rehabilitation.com.cn null China 0 null null null

624 http://www.pulmonary-rehabilitation.com.cn/WebService/Game.asmx?WSDL pulmonary-rehabilitation.com.cn null China 0 null null null

625 http://www.webxml.com.cn/WebServices/IpAddressSearchWebService.asmx?wsdl webxml.com.cn 61.147.124.120 China 1033075832 AS23650 AS Number for CHINANET jiangsu province backbone 32.0617 118.7778

626 http://www.webxml.com.cn/webservices/ChinaTVprogramWebService.asmx?WSDL webxml.com.cn 61.147.124.120 China 1033075832 AS23650 AS Number for CHINANET jiangsu province backbone 32.0617 118.7778

627 http://www.webxml.com.cn/WebServices/WeatherWebService.asmx?WSDL webxml.com.cn 61.147.124.120 China 1033075832 AS23650 AS Number for CHINANET jiangsu province backbone 32.0617 118.7778

628 http://www.webxml.com.cn/WebServices/ChinaOpenFundWS.asmx?wsdl webxml.com.cn 61.147.124.120 China 1033075832 AS23650 AS Number for CHINANET jiangsu province backbone 32.0617 118.7778

629 http://www.webxml.com.cn/WebServices/ExchangeRateWebService.asmx?wsdl webxml.com.cn 61.147.124.120 China 1033075832 AS23650 AS Number for CHINANET jiangsu province backbone 32.0617 118.7778

630 http://www.webxml.com.cn/WebServices/ValidateCodeWebService.asmx?wsdl webxml.com.cn 61.147.124.120 China 1033075832 AS23650 AS Number for CHINANET jiangsu province backbone 32.0617 118.7778

631 http://www.webxml.com.cn/WebServices/TraditionalSimplifiedWebService.asmx?wsdl webxml.com.cn 61.147.124.120 China 1033075832 AS23650 AS Number for CHINANET jiangsu province backbone 32.0617 118.7778

632 http://www.webxml.com.cn/WebServices/RandomFontsWebService.asmx?wsdl webxml.com.cn 61.147.124.120 China 1033075832 AS23650 AS Number for CHINANET jiangsu province backbone 32.0617 118.7778

633 http://www.webxml.com.cn/WebServices/ValidateEmailWebService.asmx?wsdl webxml.com.cn 61.147.124.120 China 1033075832 AS23650 AS Number for CHINANET jiangsu province backbone 32.0617 118.7778

634 http://www.webxml.com.cn/WebServices/ForexRmbRateWebService.asmx?wsdl webxml.com.cn 61.147.124.120 China 1033075832 AS23650 AS Number for CHINANET jiangsu province backbone 32.0617 118.7778

635 http://www.webxml.com.cn/WebServices/TranslatorWebService.asmx?wsdl webxml.com.cn 61.147.124.120 China 1033075832 AS23650 AS Number for CHINANET jiangsu province backbone 32.0617 118.7778

636 http://www.webxml.com.cn/webservices/DomesticAirline.asmx?wsdl webxml.com.cn 61.147.124.120 China 1033075832 AS23650 AS Number for CHINANET jiangsu province backbone 32.0617 118.7778

637 http://www.webxml.com.cn/webservices/qqOnlineWebService.asmx?wsdl webxml.com.cn 61.147.124.120 China 1033075832 AS23650 AS Number for CHINANET jiangsu province backbone 32.0617 118.7778

638 http://www.webxml.com.cn/WebServices/ChinaStockWebService.asmx?wsdl webxml.com.cn 61.147.124.120 China 1033075832 AS23650 AS Number for CHINANET jiangsu province backbone 32.0617 118.7778

639 http://www.webxml.com.cn/webservices/ChinaStockSmallImageWS.asmx?wsdl webxml.com.cn 61.147.124.120 China 1033075832 AS23650 AS Number for CHINANET jiangsu province backbone 32.0617 118.7778

640 http://www.webxml.com.cn/WebServices/ChinaZipSearchWebService.asmx?wsdl webxml.com.cn 61.147.124.120 China 1033075832 AS23650 AS Number for CHINANET jiangsu province backbone 32.0617 118.7778

641 http://www.webxml.com.cn/WebServices/MobileCodeWS.asmx?wsdl webxml.com.cn 61.147.124.120 China 1033075832 AS23650 AS Number for CHINANET jiangsu province backbone 32.0617 118.7778

642 http://www.webxml.com.cn/WebServices/WeatherWS.asmx?wsdl webxml.com.cn 61.147.124.120 China 1033075832 AS23650 AS Number for CHINANET jiangsu province backbone 32.0617 118.7778

643 http://www.webxml.com.cn/WebServices/StockInfoWS.asmx?wsdl webxml.com.cn 61.147.124.120 China 1033075832 AS23650 AS Number for CHINANET jiangsu province backbone 32.0617 118.7778

644 http://www.webxml.com.cn/WebServices/TrainTimeWebService.asmx?wsdl webxml.com.cn 61.147.124.120 China 1033075832 AS23650 AS Number for CHINANET jiangsu province backbone 32.0617 118.7778

645 http://fy.webxml.com.cn/webservices/EnglishChinese.asmx?wsdl webxml.com.cn 61.147.124.120 China 1033075832 AS23650 AS Number for CHINANET jiangsu province backbone 32.0617 118.7778

646 http://www.oceanstudio.net/_vti_bin/Authentication.asmx?wsdl oceanstudio.net 222.76.216.91 China 3729578075 AS4134 Chinanet 24.4798 118.0819

647 http://www.oceanstudio.net/_vti_bin/People.asmx?wsdl oceanstudio.net 222.76.216.91 China 3729578075 AS4134 Chinanet 24.4798 118.0819

648 http://www.yi-qiao.cn/WebService/QA.asmx?WSDL yi-qiao.cn 114.141.180.91 China 1921889371 AS45061 Shanghai Information Network Co.,Ltd. 31.0456 121.3997

649 http://www.yi-qiao.cn/WebService/Favorite.asmx?WSDL yi-qiao.cn 114.141.180.91 China 1921889371 AS45061 Shanghai Information Network Co.,Ltd. 31.0456 121.3997

650 http://www.yi-qiao.cn/WebService/Score.asmx?WSDL yi-qiao.cn 114.141.180.91 China 1921889371 AS45061 Shanghai Information Network Co.,Ltd. 31.0456 121.3997

651 http://www.yi-qiao.cn/WebService/Wiki.asmx?WSDL yi-qiao.cn 114.141.180.91 China 1921889371 AS45061 Shanghai Information Network Co.,Ltd. 31.0456 121.3997

652 http://www.yi-qiao.cn/WebService/File.asmx?WSDL yi-qiao.cn 114.141.180.91 China 1921889371 AS45061 Shanghai Information Network Co.,Ltd. 31.0456 121.3997

653 http://www.yi-qiao.cn/WebService/Module.asmx?WSDL yi-qiao.cn 114.141.180.91 China 1921889371 AS45061 Shanghai Information Network Co.,Ltd. 31.0456 121.3997

654 http://www.yi-qiao.cn/WebService/Message.asmx?WSDL yi-qiao.cn 114.141.180.91 China 1921889371 AS45061 Shanghai Information Network Co.,Ltd. 31.0456 121.3997

655 http://www.yi-qiao.cn/WebService/Register.asmx?WSDL yi-qiao.cn 114.141.180.91 China 1921889371 AS45061 Shanghai Information Network Co.,Ltd. 31.0456 121.3997

656 http://www.yi-qiao.cn/Lecture/WebService/Lecture.asmx?WSDL yi-qiao.cn 114.141.180.91 China 1921889371 AS45061 Shanghai Information Network Co.,Ltd. 31.0456 121.3997

657 http://www.yi-qiao.cn/WebService/Group.asmx?WSDL yi-qiao.cn 114.141.180.91 China 1921889371 AS45061 Shanghai Information Network Co.,Ltd. 31.0456 121.3997

658 http://www.yi-qiao.cn/WebService/Touch.asmx?WSDL yi-qiao.cn 114.141.180.91 China 1921889371 AS45061 Shanghai Information Network Co.,Ltd. 31.0456 121.3997

659 http://www.yi-qiao.cn/WebService/Friend.asmx?WSDL yi-qiao.cn 114.141.180.91 China 1921889371 AS45061 Shanghai Information Network Co.,Ltd. 31.0456 121.3997

660 http://config.shutter.cdream.com/webservice/service/GreetingCard4Flash?wsdl cdream.com 59.64.112.184 China 994078904 AS4538 China Education and Research Network Center 35 105

661 http://config.shutter.cdream.com/webservice/service/FeeCardAddBalance4Flash?wsdl cdream.com 59.64.112.184 China 994078904 AS4538 China Education and Research Network Center 35 105

662 http://58.53.194.67/services/AddressbookService?wsdl 58.53.194.67 58.53.194.67 China 976601667 AS4134 Chinanet 30.5801 114.2734

663 http://58.53.194.67/services/TradeService?wsdl 58.53.194.67 58.53.194.67 China 976601667 AS4134 Chinanet 30.5801 114.2734

664 http://58.53.194.67/services/Version?wsdl 58.53.194.67 58.53.194.67 China 976601667 AS4134 Chinanet 30.5801 114.2734

665 http://58.53.194.67/services/CTCQService?wsdl 58.53.194.67 58.53.194.67 China 976601667 AS4134 Chinanet 30.5801 114.2734

666 http://58.53.194.67/services/MessageService?wsdl 58.53.194.67 58.53.194.67 China 976601667 AS4134 Chinanet 30.5801 114.2734

667 http://58.53.194.67/services/FlowService?wsdl 58.53.194.67 58.53.194.67 China 976601667 AS4134 Chinanet 30.5801 114.2734

668 http://58.53.194.67/services/CusAddBookService?wsdl 58.53.194.67 58.53.194.67 China 976601667 AS4134 Chinanet 30.5801 114.2734

669 http://58.53.194.67/services/MailService?wsdl 58.53.194.67 58.53.194.67 China 976601667 AS4134 Chinanet 30.5801 114.2734

670 http://japan.eleva.cn/_vti_bin/Lists.asmx?wsdl eleva.cn 61.135.175.209 China 1032302545 AS4808 CNCGROUP IP network China169 Beijing Province Network 39.9289 116.3883

671 http://search.72ec.com/WebServices/SearchWebService.asmx?wsdl 72ec.com 119.254.69.161 China 2013152673 AS23844 Beijing Guanghuan Xinwang Digital 39.9289 116.3883

672 http://soft.72ec.com/WebService/RegisterService.asmx?WSDL 72ec.com 119.254.69.161 China 2013152673 AS23844 Beijing Guanghuan Xinwang Digital 39.9289 116.3883

673 http://soft.72ec.com/WebService/ECToolsService.asmx?WSDL 72ec.com 119.254.69.161 China 2013152673 AS23844 Beijing Guanghuan Xinwang Digital 39.9289 116.3883

674 http://security.72ec.com/XRegSvr.asmx?WSDL 72ec.com 119.254.69.161 China 2013152673 AS23844 Beijing Guanghuan Xinwang Digital 39.9289 116.3883

675 http://soft.72ec.com/webservice/Ssoservice.asmx?WSDL 72ec.com 119.254.69.161 China 2013152673 AS23844 Beijing Guanghuan Xinwang Digital 39.9289 116.3883

676 http://soft.72ec.com/WebService/BIMService.asmx?WSDL 72ec.com 119.254.69.161 China 2013152673 AS23844 Beijing Guanghuan Xinwang Digital 39.9289 116.3883

677 http://manage.topfo.com/GetAuditInfo.asmx?WSDL topfo.com 202.105.135.48 China 3395913520 AS4134 Chinanet 22.5333 114.1333

678 http://www1.topfo.com/ws/wsLoginM.asmx?WSDL topfo.com 202.105.135.48 China 3395913520 AS4134 Chinanet 22.5333 114.1333

679 http://rz.topfo.com/webservice/UserData.asmx?WSDL topfo.com 202.105.135.48 China 3395913520 AS4134 Chinanet 22.5333 114.1333

680 http://60.209.7.69/Lottery/WebService/HotCode.asmx?WSDL 60.209.7.69 60.209.7.69 China 1020331845 AS4837 CNCGROUP China169 Backbone 36.6683 116.9972

681 http://60.209.7.69/account/WebService/WSCommon.asmx?WSDL 60.209.7.69 60.209.7.69 China 1020331845 AS4837 CNCGROUP China169 Backbone 36.6683 116.9972

682 http://60.209.7.69/lottery/WebService/MultiTermBuy.asmx?WSDL 60.209.7.69 60.209.7.69 China 1020331845 AS4837 CNCGROUP China169 Backbone 36.6683 116.9972

683 http://60.209.7.69/Lottery/WebService/quickBuy.asmx?WSDL 60.209.7.69 60.209.7.69 China 1020331845 AS4837 CNCGROUP China169 Backbone 36.6683 116.9972

684 http://60.209.7.69/lottery/WebService/MultiBuyService.asmx?WSDL 60.209.7.69 60.209.7.69 China 1020331845 AS4837 CNCGROUP China169 Backbone 36.6683 116.9972

685 http://60.209.7.71/Lottery/WebService/HotCode.asmx?WSDL 60.209.7.71 60.209.7.71 China 1020331847 AS4837 CNCGROUP China169 Backbone 36.6683 116.9972

686 http://60.209.7.71/account/WebService/WSCommon.asmx?WSDL 60.209.7.71 60.209.7.71 China 1020331847 AS4837 CNCGROUP China169 Backbone 36.6683 116.9972

687 http://60.209.7.71/Lottery/WebService/quickBuy.asmx?WSDL 60.209.7.71 60.209.7.71 China 1020331847 AS4837 CNCGROUP China169 Backbone 36.6683 116.9972

688 http://60.209.7.71/lottery/WebService/MultiTermBuy.asmx?WSDL 60.209.7.71 60.209.7.71 China 1020331847 AS4837 CNCGROUP China169 Backbone 36.6683 116.9972

689 http://60.209.7.71/lottery/WebService/MultiBuyService.asmx?WSDL 60.209.7.71 60.209.7.71 China 1020331847 AS4837 CNCGROUP China169 Backbone 36.6683 116.9972

690 http://buy.96511.com/Lottery/WebService/HotCode.asmx?WSDL 96511.com null China 0 null null null

691 http://buy.96511.com/account/WebService/WSCommon.asmx?WSDL 96511.com null China 0 null null null

692 http://buy.96511.com/Lottery/WebService/quickBuy.asmx?WSDL 96511.com null China 0 null null null

693 http://buy.96511.com/Lottery/WebService/MultiBuyService.asmx?WSDL 96511.com null China 0 null null null

694 http://buy.96511.com/Lottery/WebService/MultiTermBuy.asmx?WSDL 96511.com null China 0 null null null

695 http://ws.foloda.com/wslocation.asmx?WSDL foloda.com 211.144.40.99 China 3549440099 AS9811 srit corp.,beijing. 39.9289 116.3883

696 http://club.foloda.com/webservice/wsGroupTopic.asmx?WSDL foloda.com 211.144.40.99 China 3549440099 AS9811 srit corp.,beijing. 39.9289 116.3883

697 http://ws.foloda.com/wsip.asmx?WSDL foloda.com 211.144.40.99 China 3549440099 AS9811 srit corp.,beijing. 39.9289 116.3883

698 http://www.njfcj.gov.cn/NetOffice/WebService/PublicHouseService.asmx?WSDL njfcj.gov.cn null China 0 null null null

699 http://www.njfcj.gov.cn/NetOffice/WebService/InterfaceService.asmx?WSDL njfcj.gov.cn null China 0 null null null

700 http://fucai.qd.sd.cn/Lottery/WebService/HotCode.asmx?WSDL qd.sd.cn 61.156.12.2 China 1033636866 AS4837 CNCGROUP China169 Backbone 36.6683 116.9972

701 http://fucai.qd.sd.cn/account/WebService/WSCommon.asmx?WSDL qd.sd.cn 61.156.12.2 China 1033636866 AS4837 CNCGROUP China169 Backbone 36.6683 116.9972

702 http://fucai.qd.sd.cn/Lottery/WebService/MultiBuyService.asmx?WSDL qd.sd.cn 61.156.12.2 China 1033636866 AS4837 CNCGROUP China169 Backbone 36.6683 116.9972

703 http://fucai.qd.sd.cn/Lottery/WebService/quickBuy.asmx?WSDL qd.sd.cn 61.156.12.2 China 1033636866 AS4837 CNCGROUP China169 Backbone 36.6683 116.9972

704 http://fucai.qd.sd.cn/Lottery/WebService/MultiTermBuy.asmx?WSDL qd.sd.cn 61.156.12.2 China 1033636866 AS4837 CNCGROUP China169 Backbone 36.6683 116.9972

705 http://ecs.com.cn/ECSWebSite/WS/Support/clsFaqSearchWS.asmx?WSDL ecs.com.cn null China 0 null null null

706 http://ecs.com.cn/ECSWebSite/WS/Support/clsCpuModelSearchWS.asmx?WSDL ecs.com.cn null China 0 null null null

707 http://passport.sme.gov.cn/passport/services/Passport?wsdl sme.gov.cn 218.249.210.10 China 3673805322 AS17816 China Unicom IP network China169 Guangdong province 39.9289 116.3883

708 http://passport.sme.gov.cn/services/Version?wsdl sme.gov.cn 218.249.210.10 China 3673805322 AS17816 China Unicom IP network China169 Guangdong province 39.9289 116.3883

709 http://ws365.net/ws/mobilephone.asmx?WSDL ws365.net 67.222.147.241 China 1138660337 AS30496 Colo4Dallas LP 32.8148 -96.8705

710 http://ws365.net/ws/weather.asmx?WSDL ws365.net 67.222.147.241 China 1138660337 AS30496 Colo4Dallas LP 32.8148 -96.8705

711 http://www.ws365.net/ws/zipcode.asmx?WSDL ws365.net 67.222.147.241 China 1138660337 AS30496 Colo4Dallas LP 32.8148 -96.8705

712 http://ws365.net/ws/peopleid.asmx?WSDL ws365.net 67.222.147.241 China 1138660337 AS30496 Colo4Dallas LP 32.8148 -96.8705

713 http://ws365.net/ws/ip.asmx?WSDL ws365.net 67.222.147.241 China 1138660337 AS30496 Colo4Dallas LP 32.8148 -96.8705

714 http://61.156.8.195:8080/axis/services/Version?wsdl 61.156.8.195 61.156.8.195 China 1033636035 AS4837 CNCGROUP China169 Backbone 36.6683 116.9972

715 http://61.156.8.195:8080/axis/AuthWS.jws?wsdl 61.156.8.195 61.156.8.195 China 1033636035 AS4837 CNCGROUP China169 Backbone 36.6683 116.9972

716 http://abkk.com/cn/train/asmx/returncheci.asmx?WSDL abkk.com 218.25.10.252 China 3659074300 AS4837 CNCGROUP China169 Backbone 41.7922 123.4328

717 http://system.fjjs.gov.cn/signonwebservice2005/JSService.asmx?wsdl fjjs.gov.cn 61.154.12.151 China 1033505943 AS4134 Chinanet 26.0614 119.3061

718 http://friend.jctrans.com/fold/WebServices/DealSP.asmx?WSDL jctrans.com 117.79.88.99 China 1968134243 AS23724 IDC, China Telecommunications Corporation 39.9289 116.3883

719 http://friend.jctrans.com/fold/WebServices/foldIndexPageDeal.asmx?WSDL jctrans.com 117.79.88.99 China 1968134243 AS23724 IDC, China Telecommunications Corporation 39.9289 116.3883

720 http://mjzz.sdmz.gov.cn:8080/axis/AuthWS.jws?wsdl sdmz.gov.cn 218.56.48.169 China 3661115561 AS4837 CNCGROUP China169 Backbone 36.6683 116.9972

721 http://mjzz.sdmz.gov.cn:8080/axis/services/Version?wsdl sdmz.gov.cn 218.56.48.169 China 3661115561 AS4837 CNCGROUP China169 Backbone 36.6683 116.9972

722 http://room.xiu.la/WebServices/MyHouseModel.asmx?WSDL xiu.la 222.216.171.243 China 3738741747 AS4134 Chinanet 22.8167 108.3167

723 http://room.xiu.la/WebServices/MyRoomSet.asmx?WSDL xiu.la 222.216.171.243 China 3738741747 AS4134 Chinanet 22.8167 108.3167

724 http://room.xiu.la/WebServices/PersionFitment.asmx?WSDL xiu.la 222.216.171.243 China 3738741747 AS4134 Chinanet 22.8167 108.3167

725 http://room.xiu.la/WebServices/HouseShop_Products.asmx?WSDL xiu.la 222.216.171.243 China 3738741747 AS4134 Chinanet 22.8167 108.3167

726 http://ynumis.ynu.edu.cn/ynumis/TeachClass/WSYJSTeachClass.asmx?WSDL ynu.edu.cn null China 0 null null null

727 http://ynumis.ynu.edu.cn/ynumis/TeachClass/WSTeachClass.asmx?WSDL ynu.edu.cn null China 0 null null null

728 http://ynumis.ynu.edu.cn/ynumis/teachPlan/WSTeachPlan.asmx?WSDL ynu.edu.cn null China 0 null null null

729 http://ynumis.ynu.edu.cn/ynumis/teachPlan/WSYJSPYJH.asmx?wsdl ynu.edu.cn null China 0 null null null

730 http://gd.100mo.cn/y/nbalive.asmx?WSDL 100mo.cn 205.164.24.44 China 3450083372 null 37.3721 -121.8643

731 http://gd.100mo.cn/y/lysc.asmx?WSDL 100mo.cn 50.117.116.204 China 846558412 null 37.3721 -121.8643

732 http://gd.100mo.cn/y/tckx.asmx?WSDL 100mo.cn 216.172.154.34 China 3635190306 null 37.3721 -121.8643

733 http://esmsws.139130.cn/services/EsmsService?wsdl 139130.cn null China 0 null null null

734 http://esmsws.139130.cn/services/Version?wsdl 139130.cn null China 0 null null null

735 http://career.cnool.net/careerService/service1.asmx?WSDL cnool.net 61.174.68.133 China 1034830981 AS4134 Chinanet 35 105

736 http://webservice.e1000e.com/flightService.asmx?WSDL e1000e.com 82.98.86.178 China 1382176434 AS12306 Plus.Line AG 51 9

737 http://admin.ips.com.cn/IpsServices/IpsServices.asmx?wsdl ips.com.cn null China 0 null null null

738 http://pay.ips.com.cn/webservice/IpsCheckTrade.asmx?WSDL ips.com.cn null China 0 null null null

739 http://oklx.com/asmx/cityservice.asmx?WSDL oklx.com 218.25.10.252 China 3659074300 AS4837 CNCGROUP China169 Backbone 41.7922 123.4328

740 http://www.smartclub.com.cn/webservice/B2C.asmx?WSDL smartclub.com.cn 210.51.21.25 China 3526563097 AS9929 China Netcom Corp. 31.0456 121.3997

741 http://www.smesy.gov.cn/services/Version?wsdl smesy.gov.cn null China 0 null null null

742 http://www.thinkpage.cn/weather/WeatherService.asmx?WSDL thinkpage.cn 42.121.112.55 China 712601655 AS237 Merit Network Inc. 30.2936 120.1614

743 http://221.238.16.68/vnetInterface/VNetForSP/VNetCenterInterfaceForSP.asmx?wsdl 221.238.16.68 221.238.16.68 China 3723366468 AS17638 ASN for TIANJIN Provincial Net of CT 39.1422 117.1767

744 http://www.35card.com/WebService/ValidateUser.asmx?WSDL 35card.com 63.156.206.202 China 1067241162 AS209 Qwest Communications Company, LLC 38 -97

745 http://168.35card.com/webservice/CardRequest.asmx?WSDL 35card.com 63.156.206.202 China 1067241162 AS209 Qwest Communications Company, LLC 38 -97

746 http://www.91very.com/WebServices/webservice.asmx?wsdl 91very.com 60.190.99.136 China 1019110280 AS4134 Chinanet 27.9994 120.6668

747 http://www.91very.com/WebServices/PaimaiMessage.asmx?WSDL 91very.com 60.190.99.136 China 1019110280 AS4134 Chinanet 27.9994 120.6668

748 http://user.9588.com/User.asmx?WSDL 9588.com 59.151.60.26 China 999767066 AS9308 Abitcool(China) Inc. 39.9289 116.3883

749 http://www.anysou.com:8888/NO6/AnySouMIS.asmx?WSDL anysou.com 211.102.90.208 China 3546700496 AS4134 Chinanet 23.1167 113.25

750 http://www.anysou.com:8888/NO8/AnySouMIS.asmx?WSDL anysou.com 211.102.90.208 China 3546700496 AS4134 Chinanet 23.1167 113.25

751 http://c8show.com/webService/C8WebService.asmx?WSDL c8show.com 58.215.81.27 China 987189531 AS4134 Chinanet 32.0617 118.7778

752 http://english.chinavalue.net/WebService/Profile.asmx?wsdl chinavalue.net 118.194.2.101 China 1992426085 AS17431 Beijing TONEK Information Technology Development Company 31.0456 121.3997

753 http://www.chinavalue.net/webservice/newsdigpublisher.asmx?WSDL chinavalue.net 118.194.2.101 China 1992426085 AS17431 Beijing TONEK Information Technology Development Company 31.0456 121.3997

754 http://quotes.api.cnfol.net/quote.wsdl cnfol.net null China 0 null null null

755 http://news.csi.com.cn/face/SmallFrame/hyrc_zw_zhss.asmx?wsdl csi.com.cn 220.189.207.19 China 3703426835 AS4134 Chinanet 35 105

756 http://114.csi.com.cn/manage/Public/getArea.asmx?wsdl csi.com.cn 220.189.207.19 China 3703426835 AS4134 Chinanet 35 105

757 http://union.ding9.com/ucpservices/services/CooperateInfoService?wsdl ding9.com 61.143.165.136 China 1032824200 AS4134 Chinanet 23.0333 113.7167

758 http://union.ding9.com/ucpservices/services/CooperateUserService?wsdl ding9.com 61.143.165.136 China 1032824200 AS4134 Chinanet 23.0333 113.7167

759 http://dp2003.com/dp2libraryws/gcat.asmx?wsdl dp2003.com 106.3.40.105 China 1778591849 null 39.9289 116.3883

760 http://www.haoqiantu.cn/WebService/SearchLeftBox.asmx?WSDL haoqiantu.cn 222.73.230.197 China 3729385157 AS4812 China Telecom (Group) 31.0456 121.3997

761 http://www.highwaydata.com.cn/cecws/services/ClassQueryWS?wsdl highwaydata.com.cn 218.249.201.143 China 3673803151 AS17816 China Unicom IP network China169 Guangdong province 39.9289 116.3883

762 http://i-dno.com/WebService.asmx?WSDL i-dno.com 221.122.114.10 China 3715789322 AS4808 CNCGROUP IP network China169 Beijing Province Network 35 105

763 http://www.iposei.com/idiomdictionary/idiomdictionary.asmx?WSDL iposei.com 173.201.119.169 China 2915661737 AS26496 GoDaddy.com, Inc. 33.6119 -111.8906

764 http://webservice.ips.net.cn/ipschecktrade.asmx?WSDL ips.net.cn 61.129.72.82 China 1031882834 AS4812 China Telecom (Group) 35 105

765 http://webservice.ips.net.cn/IPS_Payment_Confirm.asmx?WSDL ips.net.cn 61.129.72.82 China 1031882834 AS4812 China Telecom (Group) 35 105

766 http://blog.mpdaogou.com/webservice.asmx?WSDL mpdaogou.com 61.155.211.202 China 1033622474 AS4134 Chinanet 32.0617 118.7778

767 http://couplet.msra.cn/app/CoupletsWS_V2.asmx?WSDL msra.cn 202.96.51.203 China 3395302347 AS4808 CNCGROUP IP network China169 Beijing Province Network 39.9289 116.3883

768 http://www.sdws.gov.cn/Services/CommonService.asmx?WSDL sdws.gov.cn null China 0 null null null

769 http://www.sdws.gov.cn/Services/Default/DefaultService.asmx?WSDL sdws.gov.cn null China 0 null null null

770 http://hzn.secondhouse.soufun.com/HouseService/Estimate/projname.asmx?WSDL soufun.com 202.108.253.57 China 3396140345 AS4808 CNCGROUP IP network China169 Beijing Province Network 39.9289 116.3883

771 http://service.studyez.com/clientservice/clientservice.asmx?WSDL studyez.com 116.213.69.28 China 1960133916 AS24134 CNLink Networks Ltd. 35 105

772 http://fwd.studyez.com/FwdService.asmx?wsdl studyez.com 116.213.69.28 China 1960133916 AS24134 CNLink Networks Ltd. 35 105

773 http://sms.sugoo.com/UpWebService1.asmx?WSDL sugoo.com 211.103.155.103 China 3546782567 AS4808 CNCGROUP IP network China169 Beijing Province Network 39.9289 116.3883

774 http://sms.sugoo.com/DownService1.asmx?WSDL sugoo.com 211.103.155.103 China 3546782567 AS4808 CNCGROUP IP network China169 Beijing Province Network 39.9289 116.3883

775 http://www.szdoland.com/Common/SystemHandler/mailSendor.asmx?wsdl szdoland.com 123.100.4.121 China 2070152313 AS24426 Beijing CE Huatong Information Technology Co., Ltd. 35 105

776 http://msg.tiexue.net/Service/NoteBox.asmx?WSDL tiexue.net 202.85.220.197 China 3394624709 AS4847 China Networks Inter-Exchange 35 105

777 http://msg.tiexue.net/Service/UserInfo.asmx?WSDL tiexue.net 202.85.220.197 China 3394624709 AS4847 China Networks Inter-Exchange 35 105

778 http://www.uestc.edu.cn/web3/NewsCenterService.asmx?WSDL uestc.edu.cn null China 0 null null null

779 http://www.news.uestc.edu.cn/NewsCenterUESTCWebService.asmx?WSDL uestc.edu.cn null China 0 null null null

780 http://www.uobuy.com/WebService.asmx?WSDL uobuy.com null China 0 null null null

781 http://lm.uobuy.com/AutoCompleteService.asmx?WSDL uobuy.com null China 0 null null null

782 http://sms.utnet.cn/smsservice2/v2/smssend2.asmx?WSDL utnet.cn 61.157.217.123 China 1033755003 AS4134 Chinanet 30.6667 104.0667

783 http://sms.utnet.cn/SmsService2/SmsSend.asmx?WSDL utnet.cn 61.157.217.123 China 1033755003 AS4134 Chinanet 30.6667 104.0667

784 http://community.veivo.com/DesktopModules/IWeb/webservice.asmx?WSDL veivo.com 59.151.44.25 China 999762969 AS9308 Abitcool(China) Inc. 39.9289 116.3883

785 http://community.veivo.com/DesktopModules/IWebLite/ExampleWS.asmx?WSDL veivo.com 59.151.44.25 China 999762969 AS9308 Abitcool(China) Inc. 39.9289 116.3883

786 http://www.wapit.cn/WebServices/Weather.asmx?WSDL wapit.cn 211.137.251.71 China 3549035335 AS9808 Guangdong Mobile Communication Co.Ltd. 45.75 126.65

787 http://www.wapit.cn/WebServices/Mobile.asmx?wsdl wapit.cn 211.137.251.71 China 3549035335 AS9808 Guangdong Mobile Communication Co.Ltd. 45.75 126.65

788 http://www.xba.com.cn/WebService/RegService.asmx?WSDL xba.com.cn 113.31.88.230 China 1897879782 null 39.1422 117.1767

789 http://www.zhiga.net/admin/Verify/Verify.asmx?wsdl zhiga.net null China 0 null null null

790 http://www.zhiga.net/admin/service.asmx?WSDL zhiga.net null China 0 null null null

791 http://cs.zzbs.com.cn/WebService/Commodity_Online.asmx?WSDL zzbs.com.cn 122.193.18.133 China 2059473541 AS4837 CNCGROUP China169 Backbone 32.0617 118.7778

792 http://so.0316.cn/Inc/AutoComplete.asmx?WSDL 0316.cn 42.120.40.195 China 712517827 AS237 Merit Network Inc. 29.8782 121.5495

793 http://www.10156201.com/SearchAutoComplete.asmx?WSDL 10156201.com null China 0 null null null

794 http://218.1.73.12/NecVBService/Service1.asmx?wsdl 218.1.73.12 218.1.73.12 China 3657517324 AS4812 China Telecom (Group) 31.0456 121.3997

795 http://218.1.73.8/SMSService/Service1.asmx?WSDL 218.1.73.8 218.1.73.8 China 3657517320 AS4812 China Telecom (Group) 31.0456 121.3997

796 http://www.21our.com/WSForOut.asmx?wsdl 21our.com 118.192.10.47 China 1992297007 AS18245 FOUNDERBN CNNIC 39.9289 116.3883

797 http://220.201.8.29/SearchAutoComplete.asmx?WSDL 220.201.8.29 220.201.8.29 China 3704162333 AS4837 CNCGROUP China169 Backbone 39.9289 116.3883

798 http://www.35bao.net/sms.asmx?WSDL 35bao.net 61.136.198.218 China 1032373978 AS4134 Chinanet 30.5801 114.2734

799 http://51waibao.net/SNWebService.asmx?WSDL 51waibao.net 218.240.151.203 China 3673200587 AS4847 China Networks Inter-Exchange 39.9289 116.3883

800 http://www.5308.com.cn/RssWebSer.asmx?WSDL 5308.com.cn 175.41.21.2 China 2938705154 AS45753 Unit 1205-1207 22.25 114.1667

801 http://59.151.1.234/Default.asmx?wsdl 59.151.1.234 59.151.1.234 China 999752170 AS9308 Abitcool(China) Inc. 39.9289 116.3883

802 http://59.36.99.178:8888/NO6/AnySouMIS.asmx?WSDL 59.36.99.178 59.36.99.178 China 992240562 AS4134 Chinanet 23.1167 113.25

803 http://5c8.net/GetService.asmx?WSDL 5c8.net 119.40.135.178 China 1999144882 AS4837 CNCGROUP China169 Backbone 22.2769 113.5678

804 http://xiek1.anyp.cn/services/WS_BlogArticle.asmx?WSDL anyp.cn null China 0 null null null

805 http://www.ayandy.com/Service.asmx?WSDL ayandy.com 141.8.225.38 China 2366169382 null 47 8

806 http://www.bjwater.gov.cn/DesktopModules/C_Info/WebService/C_InfoService.asmx?WSDL bjwater.gov.cn 210.73.64.54 China 3528015926 AS18239 Beijing Capital Public Information Platform 35 105

807 http://www1.bjxch.gov.cn:81/VServer/MapService.asmx?WSDL bjxch.gov.cn null China 0 null null null

808 http://www.casagt.net/Services/CASIBE.asmx?WSDL casagt.net null China 0 null null null

809 http://sms.ccfei.net.cn/BigMobile.asmx?WSDL ccfei.net.cn 210.5.152.8 China 3523581960 AS24141 Shanghai DMT Information Network cor.,LTD. 31.0456 121.3997

810 http://jii1.ceping.com/service.asmx?WSDL ceping.com null China 0 null null null

811 http://xba.cga.com.cn/WebService/CoinService.asmx?WSDL cga.com.cn 61.152.103.213 China 1033398229 AS4812 China Telecom (Group) 31.0456 121.3997

812 http://search.chinalaw.net:6000/rls.asmx?WSDL chinalaw.net 203.207.226.77 China 3419398733 AS17964 Beijing Dian-Xin-Tong Network Technologies Co., Ltd. 39.9289 116.3883

813 http://cax.chinaport.gov.cn:8090/DXP/services/DXPService?wsdl chinaport.gov.cn null China 0 null null null

814 http://cisis.com.cn/ServiceControl/WebServicePic.asmx?WSDL cisis.com.cn 218.24.180.3 China 3659052035 AS4837 CNCGROUP China169 Backbone 41.7922 123.4328

815 http://group2.clozone.com/webservice/group.asmx?WSDL clozone.com 61.129.251.178 China 1031928754 AS4812 China Telecom (Group) 35 105

816 http://www.cnblogs.com/WS/AjaxWS.asmx?WSDL cnblogs.com 42.121.252.58 China 712637498 AS237 Merit Network Inc. 30.2936 120.1614

817 http://www.cnwust.com/WustNews.asmx?WSDL cnwust.com 202.114.255.13 China 3396534029 AS4538 China Education and Research Network Center 30.5801 114.2734

818 http://xfile.coopen.cn/fileservice.asmx?wsdl coopen.cn 211.100.75.5 China 3546565381 AS9803 Beijing Jingxun Public Information Technology Co., Ltd 35 105

819 http://www.digibook.cn/WebService/FlashResearch.asmx?WSDL digibook.cn 218.1.73.244 China 3657517556 AS4812 China Telecom (Group) 31.0456 121.3997

820 http://www.etoon.cn/newsystem/jtb/web/manage/getPro.asmx?WSDL etoon.cn 222.73.41.146 China 3729336722 AS4812 China Telecom (Group) 31.0456 121.3997

821 http://bbs.ezhun.com/Tools/Post.asmx?wsdl ezhun.com 123.196.117.235 China 2076472811 AS4847 China Networks Inter-Exchange 39.9289 116.3883

822 http://www.fasc-e.com/FASCService.asmx?WSDL fasc-e.com 210.235.188.162 China 3538664610 AS7679 Kyushu Telecommunication Network Co.,Inc. 33.1592 129.7228

823 http://www.fcxx.cc/main/RZTJservice.asmx?WSDL fcxx.cc 222.133.189.133 China 3733306757 AS4837 CNCGROUP China169 Backbone 35.3903 119.5458

824 http://www.fsou.com/SPS/default.asmx?WSDL fsou.com 119.57.52.16 China 2000237584 AS4808 CNCGROUP IP network China169 Beijing Province Network 39.9289 116.3883

825 http://www.huilan.com/DesktopModules/C_Info/WebService/C_InfoService.asmx?wsdl huilan.com 125.208.3.67 China 2110784323 AS24416 Beijing Primezone Technologies Inc. 39.9289 116.3883

826 http://www.hunt007.com/webservices/etiersoft.asmx?wsdl hunt007.com 61.143.225.12 China 1032839436 AS4134 Chinanet 23.1167 113.25

827 http://ninejab-sz.itownet.cn/ninejab/ServiceController.jws?wsdl itownet.cn null China 0 null null null

828 http://www.itvnet.cn/uniinterface/SCUniWebServiceForTM.asmx?WSDL itvnet.cn 222.211.66.6 China 3738386950 AS38283 CHINANET SiChuan Telecom Internet Data Center 30.6667 104.0667

829 http://www.kitesoft.cn/services/kiteservice.asmx?WSDL kitesoft.cn null China 0 null null null

830 http://ws.kxun.name/Eas_ws/EasWebSet.asmx?WSDL kxun.name 118.123.15.105 China 1987776361 AS4134 Chinanet 30.6667 104.0667

831 http://webservice.k-zone.cn/WeatherService/WeatherService.asmx?WSDL k-zone.cn 74.82.171.76 China 1246931788 AS20248 Take 2 Hosting, Inc. 37.3501 -121.9854

832 http://blog.lishewen.com.cn/api/BlogImporter.asmx?WSDL lishewen.com.cn 205.164.24.44 China 3450083372 null 37.3721 -121.8643

833 http://track.mainone.com/TraceService.asmx?wsdl mainone.com 114.80.115.145 China 1917875089 AS4812 China Telecom (Group) 31.0456 121.3997

834 http://mb345.com/WS/linkWS.asmx?wsdl mb345.com 125.69.81.40 China 2101694760 AS4134 Chinanet 30.6667 104.0667

835 http://my-cen.gov.cn/myjw/MyJwWebservices.asmx?WSDL my-cen.gov.cn null China 0 null null null

836 http://smc.myvo.cn/SMSWebService/Service.asmx?wsdl myvo.cn 122.224.70.252 China 2061518588 AS4134 Chinanet 30.2936 120.1614

837 http://www.mzdol.com/QueryVIP/VipInfo.asmx?WSDL mzdol.com null China 0 null null null

838 http://ip.npc6.com/IpSearch.asmx?WSDL npc6.com null China 0 null null null

839 http://qr.oucsoft.com/service/Decoder.asmx?WSDL oucsoft.com 71.18.74.2 China 1192380930 AS32392 Ecommerce Corporation 39.9649 -83.1383

840 http://www.pepo.cn/page/pblog/dwr/BlogWebService.asmx?WSDL pepo.cn 121.14.37.210 China 2030970322 AS4134 Chinanet 23.1167 113.25

841 http://i.qu114.com/member.asmx?WSDL qu114.com 211.151.52.200 China 3549902024 AS9308 Abitcool(China) Inc. 39.9289 116.3883

842 http://www.rzfgj.com/main/RZTJservice.asmx?WSDL rzfgj.com 222.174.148.180 China 3735983284 AS17633 ASN for Shandong Provincial Net of CT 35.3903 119.5458

843 http://www.sdzztv.com/InterAction/Service/User.asmx?WSDL sdzztv.com 218.58.228.196 China 3661292740 AS4837 CNCGROUP China169 Backbone 36.6683 116.9972

844 http://tvims.shangdu.com/SoapInterface/ServiceCheck.asmx?WSDL shangdu.com 182.118.3.166 China 3061187494 AS4837 CNCGROUP China169 Backbone 34.6836 113.5325

845 http://www.shengjing360.com/Common.asmx?WSDL shengjing360.com 121.101.216.234 China 2036717802 AS4808 CNCGROUP IP network China169 Beijing Province Network 39.9289 116.3883

846 http://www.shoufubao.com/public/BindUser.asmx?WSDL shoufubao.com 202.91.244.21 China 3395023893 AS4134 Chinanet 35 105

847 http://www.shuaiche.com/ws/Doc.asmx?WSDL shuaiche.com 210.14.146.237 China 3524170477 null 39.9289 116.3883

848 http://buycar.auto.sina.com.cn/ForeWeb/Correct_Bug_Comment/Service1.asmx?wsdl sina.com.cn 202.108.33.60 China 3396084028 AS4808 CNCGROUP IP network China169 Beijing Province Network 39.9289 116.3883

849 http://bj.auto.sohu.com/ForeWeb/Correct_Bug_Comment/Service1.asmx?wsdl sohu.com 61.135.181.176 China 1032304048 AS4808 CNCGROUP IP network China169 Beijing Province Network 39.9289 116.3883

850 http://demo.stu.edu.cn:8080/StudentInfoWebService/StudentInfoWebService.asmx?WSDL stu.edu.cn 202.104.245.188 China 3395876284 AS4134 Chinanet 23.1167 113.25

851 http://www.szrc.cn/Services/Service.asmx?WSDL szrc.cn null China 0 null null null

852 http://www.topdriver.cn/ws/userservice.asmx?WSDL topdriver.cn 218.57.241.35 China 3661230371 AS4837 CNCGROUP China169 Backbone 36.6683 116.9972

853 http://www.trip-x.com/Member/Webserice/AreaCodeWS.asmx?WSDL trip-x.com 58.211.139.82 China 986942290 AS23650 AS Number for CHINANET jiangsu province backbone 32.0617 118.7778

854 http://www.bioinfo.tsinghua.edu.cn/~tigerchen/SubLoc.wsdl tsinghua.edu.cn null China 0 null null null

855 http://sh.w8m.com.cn/Service.asmx?WSDL w8m.com.cn null China 0 null null null

856 http://www.wanguolawyer.com/DesktopModules/C_Info/WebService/C_InfoService.asmx?WSDL wanguolawyer.com 218.97.241.115 China 3663851891 AS4847 China Networks Inter-Exchange 39.9289 116.3883

857 http://www.wanguoschool.net/DesktopModules/C_Info/WebService/C_InfoService.asmx?WSDL wanguoschool.net 118.144.80.238 China 1989169390 AS4847 China Networks Inter-Exchange 39.9289 116.3883

858 http://www.web086.com/WebService.asmx?WSDL web086.com 184.168.221.91 China 3098074459 AS26496 GoDaddy.com, Inc. 33.6119 -111.8906

859 http://wupeng.cn/api/BlogImporter.asmx?WSDL wupeng.cn 173.212.197.252 China 2916402684 AS21788 Network Operations Center Inc. 41.409 -75.6624

860 http://reg.wuxi.cn/ws/renzheng.asmx?WSDL wuxi.cn 202.102.2.132 China 3395682948 AS4134 Chinanet 32.0617 118.7778

861 http://yishan.cc/QueryAutoComplete.asmx?WSDL yishan.cc 106.186.16.91 China 1790578779 null 35.69 139.69

862 http://course.zhikao365.net/drm/HaihaisoftDRMService.asmx?wsdl zhikao365.net 222.73.230.220 China 3729385180 AS4812 China Telecom (Group) 31.0456 121.3997

863 http://www.extensio.com/ExtensioInfoServer/ExtensioWebServices/Currency_Code_Search.jws?wsdl extensio.com 69.60.114.57 United States 1161589305 AS15083 Infolink 40.7934 -77.86

864 http://www.extensio.com/ExtensioInfoServer/ExtensioWebServices/Stock_Quote_Detailed.jws?wsdl extensio.com 69.60.114.57 United States 1161589305 AS15083 Infolink 40.7934 -77.86

865 http://www.extensio.com/ExtensioInfoServer/ExtensioWebServices/Customer_List.jws?wsdl extensio.com 69.60.114.57 United States 1161589305 AS15083 Infolink 40.7934 -77.86

866 http://www.extensio.com/ExtensioInfoServer/ExtensioWebServices/Customer_Update.jws?wsdl extensio.com 69.60.114.57 United States 1161589305 AS15083 Infolink 40.7934 -77.86

867 http://www.extensio.com/ExtensioInfoServer/ExtensioWebServices/Currency_Converter.jws?wsdl extensio.com 69.60.114.57 United States 1161589305 AS15083 Infolink 40.7934 -77.86

868 http://www.extensio.com/ExtensioInfoServer/ExtensioWebServices/Product_Search_Yahoo.jws?wsdl extensio.com 69.60.114.57 United States 1161589305 AS15083 Infolink 40.7934 -77.86

869 http://www.extensio.com/ExtensioInfoServer/ExtensioWebServices/Gold_Future.jws?wsdl extensio.com 69.60.114.57 United States 1161589305 AS15083 Infolink 40.7934 -77.86

870 http://www.extensio.com/ExtensioInfoServer/ExtensioWebServices/Customer_Details.jws?wsdl extensio.com 69.60.114.57 United States 1161589305 AS15083 Infolink 40.7934 -77.86

871 http://www.extensio.com/ExtensioInfoServer/ExtensioWebServices/Stock_Quotes.jws?wsdl extensio.com 69.60.114.57 United States 1161589305 AS15083 Infolink 40.7934 -77.86

872 http://www.extensio.com/ExtensioInfoServer/ExtensioWebServices/Stock_History_Current_Week.jws?wsdl extensio.com 69.60.114.57 United States 1161589305 AS15083 Infolink 40.7934 -77.86

873 http://www.extensio.com/ExtensioInfoServer/ExtensioWebServices/Product_Discount_Update.jws?wsdl extensio.com 69.60.114.57 United States 1161589305 AS15083 Infolink 40.7934 -77.86

874 http://www.extensio.com/ExtensioInfoServer/ExtensioWebServices/Stock_Quote_Yesterday.jws?wsdl extensio.com 69.60.114.57 United States 1161589305 AS15083 Infolink 40.7934 -77.86

875 http://www.extensio.com/ExtensioInfoServer/ExtensioWebServices/Stock_Ticker_Search_non_US_CAN.jws?wsdl extensio.com 69.60.114.57 United States 1161589305 AS15083 Infolink 40.7934 -77.86

876 http://www.extensio.com/ExtensioInfoServer/ExtensioWebServices/Yahoo_Business_Headlines.jws?wsdl extensio.com 69.60.114.57 United States 1161589305 AS15083 Infolink 40.7934 -77.86

877 http://www.extensio.com/ExtensioInfoServer/ExtensioWebServices/Yahoo_Top_Stories.jws?wsdl extensio.com 69.60.114.57 United States 1161589305 AS15083 Infolink 40.7934 -77.86

878 http://www.extensio.com/ExtensioInfoServer/ExtensioWebServices/News_for_give_Stock_Ticker.jws?wsdl extensio.com 69.60.114.57 United States 1161589305 AS15083 Infolink 40.7934 -77.86

879 http://www.extensio.com/ExtensioInfoServer/ExtensioWebServices/Yahoo_Technology_Headlines.jws?wsdl extensio.com 69.60.114.57 United States 1161589305 AS15083 Infolink 40.7934 -77.86

880 http://www.extensio.com/ExtensioInfoServer/ExtensioWebServices/Product_Discount.jws?wsdl extensio.com 69.60.114.57 United States 1161589305 AS15083 Infolink 40.7934 -77.86

881 http://www.extensio.com/ExtensioInfoServer/ExtensioWebServices/Product_List.jws?wsdl extensio.com 69.60.114.57 United States 1161589305 AS15083 Infolink 40.7934 -77.86

882 http://www.extensio.com/ExtensioInfoServer/ExtensioWebServices/Product_Qty_ATP.jws?wsdl extensio.com 69.60.114.57 United States 1161589305 AS15083 Infolink 40.7934 -77.86

883 http://www.extensio.com/ExtensioInfoServer/ExtensioWebServices/Citywise_Customers.jws?wsdl extensio.com 69.60.114.57 United States 1161589305 AS15083 Infolink 40.7934 -77.86

884 http://www.extensio.com/ExtensioInfoServer/ExtensioWebServices/Stock_History_Previous_Week.jws?wsdl extensio.com null United States 0 null null null

885 http://www.extensio.com/ExtensioInfoServer/ExtensioWebServices/Product_Update.jws?wsdl extensio.com 69.60.114.57 United States 1161589305 AS15083 Infolink 40.7934 -77.86

886 http://www.extensio.com/ExtensioInfoServer/ExtensioWebServices/Stock_Ticker_Search_US_CAN.jws?wsdl extensio.com 69.60.114.57 United States 1161589305 AS15083 Infolink 40.7934 -77.86

887 http://www.extensio.com/ExtensioInfoServer/ExtensioWebServices/Product_Price_Update.jws?wsdl extensio.com 69.60.114.57 United States 1161589305 AS15083 Infolink 40.7934 -77.86

888 http://www.extensio.com/ExtensioInfoServer/ExtensioWebServices/News_Search.jws?wsdl extensio.com 69.60.114.57 United States 1161589305 AS15083 Infolink 40.7934 -77.86

889 http://www.extensio.com/ExtensioInfoServer/ExtensioWebServices/Silver_Future.jws?wsdl extensio.com 69.60.114.57 United States 1161589305 AS15083 Infolink 40.7934 -77.86

890 http://www.extensio.com/ExtensioInfoServer/ExtensioWebServices/Product_Details.jws?wsdl extensio.com 69.60.114.57 United States 1161589305 AS15083 Infolink 40.7934 -77.86

891 http://www.extensio.com/ExtensioInfoServer/ExtensioWebServices/Customer_Order_Status.jws?wsdl extensio.com 69.60.114.57 United States 1161589305 AS15083 Infolink 40.7934 -77.86

892 http://servicios.ccc.org.co/CCS_WebService/PR03.asmx?wsdl ccc.org.co 198.101.197.217 Colombia 3328558553 null 29.4889 -98.3987

893 http://service.ssn.gov.ar/ws_kausay/CodigosDeSeguimiento.asmx?WSDL ssn.gov.ar 200.61.190.10 Colombia 3359489546 AS16814 S.A. -34.5875 -58.6725

894 http://www.fundes.org/_vti_bin/BusinessDataCatalog.asmx?wsdl fundes.org 46.4.63.195 Costa rica 772030403 AS24940 Hetzner Online AG RZ 51 9

895 http://www.fundes.org/_vti_bin/Authentication.asmx?wsdl fundes.org 46.4.63.195 Costa rica 772030403 AS24940 Hetzner Online AG RZ 51 9

896 http://www.fundes.org/_vti_bin/People.asmx?wsdl fundes.org 46.4.63.195 Costa rica 772030403 AS24940 Hetzner Online AG RZ 51 9

897 http://www.innovatechnology.net/_vti_bin/SharepointEmailWS.asmx?wsdl innovatechnology.net 201.201.187.221 Costa rica 3385441245 AS11830 Instituto Costarricense de Electricidad y Telecom. 10 -84

898 http://www.innovatechnology.net/_vti_bin/People.asmx?wsdl innovatechnology.net 201.201.187.221 Costa rica 3385441245 AS11830 Instituto Costarricense de Electricidad y Telecom. 10 -84

899 http://www.innovatechnology.net/_vti_bin/Authentication.asmx?wsdl innovatechnology.net 201.201.187.221 Costa rica 3385441245 AS11830 Instituto Costarricense de Electricidad y Telecom. 10 -84

900 http://196.40.87.163/PolarWebService/PolarWebService.asmx?WSDL 196.40.87.163 196.40.87.163 Costa rica 3290978211 AS3790 RADIOGRAFICA COSTARRICENSE 10.0162 -84.2116

901 http://feed.horizon20.com:2007/h20getfeed.asmx?WSDL horizon20.com null Costa rica 0 null null null

902 http://api.mycasinoaccounts.com/ProgressiveStats.asmx?WSDL mycasinoaccounts.com 66.212.227.124 Costa rica 1121248124 AS14537 Mohawk Internet Technologies 45.5 -73.5833

903 http://services.skybook.com/linesservice/gamelinesservice.asmx?WSDL skybook.com 194.28.158.132 Costa rica 3256655492 AS51006 ZEN Network Technologies Ltd 51.5 -0.13

904 http://www.perihel.hr/_vti_bin/SharepointEmailWS.asmx?wsdl perihel.hr 188.129.68.236 Croatia 3162588396 AS29485 Amis Telekom d.o.o 45.8 16

905 http://www.perihel.hr/_vti_bin/People.asmx?wsdl perihel.hr 188.129.68.236 Croatia 3162588396 AS29485 Amis Telekom d.o.o 45.8 16

906 http://www.perihel.hr/_vti_bin/Authentication.asmx?wsdl perihel.hr 188.129.68.236 Croatia 3162588396 AS29485 Amis Telekom d.o.o 45.8 16

907 http://x2.cap.srce.hr/slike/service.asmx?WSDL srce.hr null Croatia 0 null null null

908 http://www.jutarnji.hr/Modules/Comments/ForumWS.asmx?WSDL jutarnji.hr 91.214.104.22 Croatia 1540778006 AS49498 S&T Hrvatska d.o.o 45.1667 15.5

909 http://autoklub.jutarnji.hr/Modules/Cars/CarWS.asmx?WSDL jutarnji.hr 91.214.104.22 Croatia 1540778006 AS49498 S&T Hrvatska d.o.o 45.1667 15.5

910 http://service.adriatic-gate.hr/Dispozicija.asmx?wsdl adriatic-gate.hr 212.91.124.189 Croatia 3562765501 AS12810 VIPnet d.o.o. 45.1667 15.5

911 http://www.poup.hr/ws/ws.asmx?WSDL poup.hr 213.202.100.36 Croatia 3586810916 AS13046 ISKON 45.1667 15.5

912 http://demo.speedcard.com/MerchantServiceV3NonWSE/MerchantService.asmx?WSDL speedcard.com null Cyprus 0 null null null

913 http://www.emris.cz/_vti_bin/BusinessDataCatalog.asmx?wsdl emris.cz 93.185.104.20 Czech Republic 1572431892 AS43541 VSHosting s.r.o. 49.75 15.5

914 http://www.emris.cz/_vti_bin/People.asmx?wsdl emris.cz 93.185.104.20 Czech Republic 1572431892 AS43541 VSHosting s.r.o. 49.75 15.5

915 http://www.emris.cz/_vti_bin/Authentication.asmx?wsdl emris.cz 93.185.104.20 Czech Republic 1572431892 AS43541 VSHosting s.r.o. 49.75 15.5

916 http://www.ums.cz/_vti_bin/People.asmx?wsdl ums.cz 89.185.231.140 Czech Republic 1505355660 AS24971 Master Internet s.r.o / Czech Republic / www.master.cz 49.75 15.5

917 http://www.ums.cz/_vti_bin/Authentication.asmx?wsdl ums.cz 89.185.231.140 Czech Republic 1505355660 AS24971 Master Internet s.r.o / Czech Republic / www.master.cz 49.75 15.5

918 http://www.cs.mfcr.cz/tariccz/tariccz.asmx?wsdl mfcr.cz 193.86.123.147 Czech Republic 3243670419 AS2819 GTS NOVERA (GTS CZ) 49.75 15.5

919 http://internetliberec.cz/_vti_bin/Lists.asmx?wsdl internetliberec.cz 77.104.217.230 Czech Republic 1298717158 AS39906 CoProSys a.s. 49.75 15.5

920 http://data.plzensky-kraj.cz/report/_vti_bin/Lists.asmx?wsdl plzensky-kraj.cz 195.113.166.157 Czech Republic 3279005341 AS2852 CESNET, z.s.p.o. 49.75 13.3667

921 http://www.internetcz.info/_vti_bin/Lists.asmx?wsdl internetcz.info null Czech Republic 0 null null null

922 http://www.premis.cz/PremisWS/MeteorologyWS.asmx?WSDL premis.cz null Czech Republic 0 null null null

923 http://www.premis.cz/PremisWS/WeatherWS.asmx?WSDL premis.cz null Czech Republic 0 null null null

924 http://www.premis.cz/PremisWS/ImmissionWS.asmx?WSDL premis.cz null Czech Republic 0 null null null

925 http://www.premis.cz/PremisWS/WarningWS.asmx?WSDL premis.cz null Czech Republic 0 null null null

926 http://www.premis.cz/PremisWS/HydrologyWS.asmx?WSDL premis.cz null Czech Republic 0 null null null

927 http://atcomp.cz/webservices/DetailZbozi.asmx?WSDL atcomp.cz 81.30.226.135 Czech Republic 1360978567 AS15935 ha-vel internet spol. s r.o. 49.75 15.5

928 http://atcomp.cz/webservices/dokumenty.asmx?WSDL atcomp.cz 81.30.226.135 Czech Republic 1360978567 AS15935 ha-vel internet spol. s r.o. 49.75 15.5

929 http://atcomp.cz/webservices/ciselniky.asmx?WSDL atcomp.cz 81.30.226.135 Czech Republic 1360978567 AS15935 ha-vel internet spol. s r.o. 49.75 15.5

930 http://atcomp.sk/webservices/ciselniky.asmx?WSDL atcomp.sk 81.30.226.135 Czech Republic 1360978567 AS15935 ha-vel internet spol. s r.o. 49.75 15.5

931 http://atcomp.sk/webservices/DetailZbozi.asmx?WSDL atcomp.sk 81.30.226.135 Czech Republic 1360978567 AS15935 ha-vel internet spol. s r.o. 49.75 15.5

932 http://atcomp.sk/webservices/dokumenty.asmx?WSDL atcomp.sk 81.30.226.135 Czech Republic 1360978567 AS15935 ha-vel internet spol. s r.o. 49.75 15.5

933 http://www.atcomputers.cz/webservices/dokumenty.asmx?WSDL atcomputers.cz 81.30.226.144 Czech Republic 1360978576 AS15935 ha-vel internet spol. s r.o. 49.75 15.5

934 http://www.atcomputers.cz/webservices/DetailZbozi.asmx?WSDL atcomputers.cz 81.30.226.144 Czech Republic 1360978576 AS15935 ha-vel internet spol. s r.o. 49.75 15.5

935 http://www.atcomputers.cz/webservices/ciselniky.asmx?WSDL atcomputers.cz 81.30.226.144 Czech Republic 1360978576 AS15935 ha-vel internet spol. s r.o. 49.75 15.5

936 http://transcat.vsb.cz/tc_webServices/ws.php?wsdl vsb.cz null Czech Republic 0 null null null

937 http://transcat.vsb.cz/soap/wsdl/transform_coord.wsdl vsb.cz null Czech Republic 0 null null null

938 http://gis.vsb.cz/webcastledev/scripts/postgis.wsdl vsb.cz null Czech Republic 0 null null null

939 http://vbnet.aspweb.cz/cs2vb.asmx?WSDL aspweb.cz 217.31.49.20 Czech Republic 3642700052 AS29134 Ignum s.r.o. 50.0833 14.4667

940 http://nestws.aspweb.cz/nestws.asmx?WSDL aspweb.cz 217.31.49.20 Czech Republic 3642700052 AS29134 Ignum s.r.o. 50.0833 14.4667

941 http://www.codoma.cz/codomabox_kovotour/AppService.asmx?WSDL codoma.cz null Czech Republic 0 null null null

942 http://flash-01.geewa.com/2.1/service/game.asmx?wsdl geewa.com 88.86.109.87 Czech Republic 1482059095 AS39392 SuperNetwork s.r.o. 49.75 15.5

943 http://inphotoweb.itsnet.cz/Service/PhotoServer.asmx?WSDL itsnet.cz 193.105.159.124 Czech Republic 3244924796 AS24971 Master Internet s.r.o / Czech Republic / www.master.cz 49.75 15.5

944 http://public.ws.cz.elinkx.biz/service.asmx?WSDL elinkx.biz null Czech Republic 0 null null null

945 http://public.ws.elx.cz/service.asmx?wsdl elx.cz null Czech Republic 0 null null null

946 http://www.env.cz/ippc/wsdl env.cz 193.179.186.84 Czech Republic 3249781332 AS2819 GTS NOVERA (GTS CZ) 50.0833 14.4667

947 http://ima.infomapservices.com/IMAWebService.asmx?WSDL infomapservices.com 93.91.29.44 United Kingdom 1566252332 AS24958 The Bunker Secure Hosting Limited 51.2667 0.2

948 http://infomapservices.cz/infomapservices.asmx?WSDL infomapservices.cz 93.91.29.41 United Kingdom 1566252329 AS24958 The Bunker Secure Hosting Limited 51.2667 0.2

949 http://inphoto.cz/Service/PhotoServer.asmx?WSDL inphoto.cz 193.105.159.124 Czech Republic 3244924796 AS24971 Master Internet s.r.o / Czech Republic / www.master.cz 49.75 15.5

950 http://neptun.newstin.com/newstin-api?wsdl newstin.com 81.177.139.23 Czech Republic 1370589975 AS8342 OJSC RTComm.RU 60 100

951 http://webservices.dotnet.nexum.cz/HelpDesk.asmx?WSDL nexum.cz 195.250.146.78 Czech Republic 3287978574 AS6706 Volny a.s. 49.75 15.5

952 http://ekronika.olportal.cz/Services/AutoComplete.asmx?WSDL olportal.cz 195.113.183.3 Czech Republic 3279009539 AS2852 CESNET, z.s.p.o. 49.594 17.2512

953 http://www.patria.cz/onlinedataprovider.asmx?WSDL patria.cz 89.233.174.25 Czech Republic 1508486681 AS12767 T-Systems Czech Republic a.s. 49.75 15.5

954 http://visualprog.cz/Database/service1.asmx?wsdl visualprog.cz 88.86.104.5 Czech Republic 1482057733 AS39392 SuperNetwork s.r.o. 49.75 15.5

955 http://www.moviatrafik.dk/_vti_bin/People.asmx?wsdl moviatrafik.dk 129.142.32.126 Denmark 2173575294 AS6785 Cybercity A/S 56 10

956 http://www.moviatrafik.dk/_vti_bin/Authentication.asmx?wsdl moviatrafik.dk 129.142.32.126 Denmark 2173575294 AS6785 Cybercity A/S 56 10

957 http://event.peoplenet.dk/_vti_bin/People.asmx?wsdl peoplenet.dk 77.221.227.221 Denmark 1306387421 AS42876 T26 Technology A/S 55.6667 12.5833

958 http://event.peoplenet.dk/_vti_bin/Authentication.asmx?wsdl peoplenet.dk 77.221.227.221 Denmark 1306387421 AS42876 T26 Technology A/S 55.6667 12.5833

959 http://www.barnaland.is/dev/phonebook.asmx?WSDL barnaland.is 80.248.30.70 Iceland 1358437958 AS39418 Nyherji hf 64.136 -21.9203

960 http://barnaland.is/dev/Authentication.asmx?WSDL barnaland.is 80.248.30.70 Iceland 1358437958 AS39418 Nyherji hf 64.136 -21.9203

961 http://www.barnaland.is/dev/names.asmx?WSDL barnaland.is 80.248.30.70 Iceland 1358437958 AS39418 Nyherji hf 64.136 -21.9203

962 http://www.barnaland.is/dev/puki.asmx?WSDL barnaland.is 80.248.30.70 Iceland 1358437958 AS39418 Nyherji hf 64.136 -21.9203

963 http://www.barnaland.is/dev/sms.asmx?WSDL barnaland.is 80.248.30.70 Iceland 1358437958 AS39418 Nyherji hf 64.136 -21.9203

964 http://xmltools.oio.dk/AuthorityCode/AuthorityCodeConversion.asmx?wsdl oio.dk 79.125.10.213 Denmark 1333594837 AS39111 Amazon EU DC AS 53 -8

965 http://vbn.aau.dk/ws/services/PureWebService?wsdl aau.dk 130.225.63.2 Denmark 2195799810 AS1835 Forskningsnettet - Danish network for Research and Education 56.17 10.0426

966 http://babyverden.dk/dev/Authentication.asmx?WSDL babyverden.dk 62.80.102.40 Iceland 1045456424 AS8218 AS Confederation of Neotelecoms, euNetworks AG and Upstreamnet gmbh 51 9

967 http://babyworld.net/dev/Authentication.asmx?WSDL babyworld.net 80.248.30.70 Iceland 1358437958 AS39418 Nyherji hf 64.136 -21.9203

968 http://barnaland.fo/dev/Authentication.asmx?WSDL barnaland.fo 212.30.229.5 Iceland 3558794501 AS44515 EJS hf 65 -18

969 http://bloggland.is/dev/Authentication.asmx?WSDL bloggland.is 80.248.30.70 Iceland 1358437958 AS39418 Nyherji hf 64.136 -21.9203

970 http://dyraland.is/dev/Authentication.asmx?WSDL dyraland.is 80.248.30.70 Iceland 1358437958 AS39418 Nyherji hf 64.136 -21.9203

971 http://frontur.com/dev/Authentication.asmx?WSDL frontur.com 80.248.30.70 Iceland 1358437958 AS39418 Nyherji hf 64.136 -21.9203

972 http://www.molberg.dk/_vti_bin/Lists.asmx?wsdl molberg.dk null Denmark 0 null null null

973 http://mypet.dk/dev/Authentication.asmx?WSDL mypet.dk 92.61.150.78 Iceland 1547540046 AS29671 Servage GmbH 47 8

974 http://netbib.statsbiblioteket.dk/netbib/services/Clusters?wsdl statsbiblioteket.dk 130.225.24.24 Denmark 2195789848 AS1835 Forskningsnettet - Danish network for Research and Education 56.3155 10.3204

975 http://webservice.statsbiblioteket.dk/ws-ekopicopydan/services/CopyDanServicePort?wsdl statsbiblioteket.dk 130.225.24.24 Denmark 2195789848 AS1835 Forskningsnettet - Danish network for Research and Education 56.3155 10.3204

976 http://jupiter.geus.dk/ws_read.1.0/services/read?wsdl geus.dk 89.221.166.176 Denmark 1507698352 AS34932 Fuzion is a Danish Internet Service Provider 56 10

977 http://jupiter.geus.dk/ws_read.1.0/services/select?wsdl geus.dk 89.221.166.176 Denmark 1507698352 AS34932 Fuzion is a Danish Internet Service Provider 56 10

978 http://webservices.masterpiece.dk/webservices/MpService/Artikler.asmx?WSDL masterpiece.dk 178.23.177.76 Denmark 2987897164 AS196724 Lynero ApS 56 10

979 http://webservices.masterpiece.dk/webservices/SitePictureSeries/PictureSeries.asmx?WSDL masterpiece.dk 178.23.177.76 Denmark 2987897164 AS196724 Lynero ApS 56 10

980 http://dwi01.dandomain.dk/shop5/2.0/dwiwebservice.asmx?WSDL dandomain.dk 194.150.112.10 Denmark 3264638986 AS43220 Web hotels, domain sales, etc 56.4607 10.0364

981 http://pay.dandomain.dk/service/payservice.asmx?WSDL dandomain.dk 194.150.112.10 Denmark 3264638986 AS43220 Web hotels, domain sales, etc 56.4607 10.0364

982 http://www.datanom.net/~mir/freedb/FreeDBServer.php?wsdl datanom.net 90.184.68.2 Denmark 1522025474 AS39554 Fullrate A/S 55.6352 12.6489

983 http://www.datanom.net/~mir/server/uddiServer.php?wsdl datanom.net 90.184.68.2 Denmark 1522025474 AS39554 Fullrate A/S 55.6352 12.6489

984 http://www.forsikringsguiden.dk/_ControlTemplates/FGFrontend/WebServices/OccupationWS.asmx?WSDL forsikringsguiden.dk 188.244.66.72 Denmark 3170124360 AS41045 BUTLERnetworks 55.6667 12.5833

985 http://www.fedest.dk/ws/WSPostBy.asmx?WSDL fedest.dk 195.128.175.2 Denmark 3279990530 AS43059 Talk Active Autonomous System 56 10

986 http://www.fedest.dk/ws/WSRenteBeregning.asmx?wsdl fedest.dk 195.128.175.2 Denmark 3279990530 AS43059 Talk Active Autonomous System 56 10

987 http://eksb.jm-media.dk/App_Services/ContentProviderService.asmx?WSDL jm-media.dk null Denmark 0 null null null

988 http://autregwebservice.sst.dk/autregservice.asmx?WSDL sst.dk 193.163.131.25 Denmark 3248718617 null 55.6785 12.5221

989 http://www.vejlebib.dk/webservices/mathservice.asmx?WSDL vejlebib.dk 193.111.162.49 Denmark 3245318705 AS24853 Danish Bibliographic Centre A/S 55.7198 12.352

990 http://billeder.visitdenmark.com/dzsearchWs/dzSearch.asmx?WSDL visitdenmark.com 109.238.51.38 Denmark 1844327206 AS12617 Armada Hosting ApS 56 10

991 http://www.airtiki.com/AutoComplete.asmx?WSDL airtiki.com 195.249.147.85 Denmark 3287913301 AS3292 TDC Data Networks 55.6581 12.5241

992 http://airtiki.dk/AutoComplete.asmx?WSDL airtiki.dk 195.249.147.85 Denmark 3287913301 AS3292 TDC Data Networks 55.6581 12.5241

993 http://airtiki.eu/AutoComplete.asmx?WSDL airtiki.eu 195.249.147.85 Denmark 3287913301 AS3292 TDC Data Networks 55.6581 12.5241

994 http://airtiki.net/AutoComplete.asmx?WSDL airtiki.net 195.249.147.85 Denmark 3287913301 AS3292 TDC Data Networks 55.6581 12.5241

995 http://airtiki.se/AutoComplete.asmx?WSDL airtiki.se 195.249.147.85 Denmark 3287913301 AS3292 TDC Data Networks 55.6581 12.5241

996 http://webservice.fdm-travel.anet.dk/productws/product.asmx?WSDL anet.dk null Denmark 0 null null null

997 http://www.billedblog.dk/section/user/profile/FriendsService.asmx?WSDL billedblog.dk 194.152.39.225 Denmark 3264751585 AS47199 Freeway ApS 56 10

998 http://bips.dk/BipsWebApp/Service/BipsProjectWS.asmx?WSDL bips.dk 81.7.134.254 Denmark 1359447806 AS16095 jay.net a/s 55.6667 12.5833

999 http://www.borgerblanketter.dk/BBWS/BBWS.asmx?WSDL borgerblanketter.dk 193.201.39.156 Denmark 3251185564 AS24969 DIR A/S DK 56 10

1000 http://www.carlsberg.com/gateway.asmx?WSDL carlsberg.com 217.116.236.143 Denmark 3648318607 AS16245 NetGroup A/S 56 10

1001 http://cvr.dk/Site/WebServices/SuggestionsService.asmx?WSDL cvr.dk 92.43.125.48 Denmark 1546353968 AS44398 TDC Hosting 56 10

1002 http://www.dalumuc.dk/AutoComplete.asmx?WSDL dalumuc.dk 195.41.131.69 Denmark 3274277701 AS3292 TDC Data Networks 56.0313 9.9317

1003 http://vdk.digizuite.dk/dmm3fws/default.asmx?WSDL digizuite.dk 87.54.40.31 Denmark 1463167007 AS3292 TDC Data Networks 56 10

1004 http://diaform.dotnet.dir.dk/service.asmx?wsdl dir.dk 178.23.242.10 Denmark 2987913738 AS24969 DIR A/S DK 56 10

1005 http://dk-camp.dk/Webservices/search.asmx?wsdl dk-camp.dk 178.23.177.66 Denmark 2987897154 AS196724 Lynero ApS 56 10

1006 http://www.dotbot.dk/webservice/dotbot.asmx?WSDL dotbot.dk null Denmark 0 null null null

1007 http://www.dotnetblogengine.net/api/BlogImporter.asmx?WSDL dotnetblogengine.net 208.78.30.79 Denmark 3494780495 AS29838 Atlantic Metro Communications 40.7143 -74.006

1008 http://opgaver.eavest.dk/hk19/palindrome.asmx?WSDL eavest.dk 37.128.213.163 Denmark 629200291 null 55.4703 8.4519

1009 http://ehsrv.electronichousekeeper.com/HouseKeeperService2/HousekeeperWebSrv.asmx?WSDL electronichousekeeper.com 80.161.31.26 Denmark 1352736538 AS3292 TDC Data Networks 55.7718 12.506

1010 http://www.fair.dk/fair/services/CarPriceService?wsdl fair.dk 93.90.118.100 Denmark 1566209636 AS45032 Fab:IT ApS AS 56 10

1011 http://www.fasil.dk/CalculationService.asmx?WSDL fasil.dk 212.97.133.63 Denmark 3563160895 AS9120 Cohaesio A/S 55.6667 12.5833

1012 http://www.flsmidth.com/Services/LocalNavigation.asmx?WSDL flsmidth.com 91.220.7.43 Denmark 1541146411 null 55.6785 12.5221

1013 http://www.food-net.dk/Service.asmx?WSDL food-net.dk null Denmark 0 null null null

1014 http://www.forsikringslup.dk/_ControlTemplates/FGFrontend/WebServices/OccupationWS.asmx?WSDL forsikringslup.dk 147.29.62.50 Denmark 2468167218 AS5624 Computer Management and CSC Denmark 56 10

1015 http://www.forsikringsluppen.dk/_ControlTemplates/FGFrontend/WebServices/OccupationWS.asmx?WSDL forsikringsluppen.dk 188.244.66.72 Denmark 3170124360 AS41045 BUTLERnetworks 55.6667 12.5833

1016 http://www.fr-tech.dk/service.asmx?WSDL fr-tech.dk 81.19.230.18 Denmark 1360258578 AS16095 jay.net a/s 56 10

1017 http://www.frtech.dk/service.asmx?WSDL frtech.dk 81.19.230.18 Denmark 1360258578 AS16095 jay.net a/s 56 10

1018 http://webservices.jubii.dk/jubiiuser/jubiiuser.asmx?WSDL jubii.dk 83.221.146.0 Denmark 1407029760 AS16245 NetGroup A/S 55.6667 12.5833

1019 http://tv2.momondo.com/Momondo.asmx?WSDL momondo.com 82.211.112.11 Denmark 1389588491 AS24867 Adapt Services Ltd 51.5 -0.13

1020 http://search.mondosoft.com/webservice/researchservice.asmx?wsdl mondosoft.com null Denmark 0 null null null

1021 http://www.musikbibliotek.dk/wsdl.php?wsdl musikbibliotek.dk 195.215.127.185 Denmark 3285680057 AS3292 TDC Data Networks 56 10

1022 http://proxy.peoplegroup.dk/erez3/erez?cmd=expand&vtl=soap/erez3.wsdl peoplegroup.dk 109.202.140.211 Denmark 1841990867 AS28717 Zen Systems 55.6667 12.5833

1023 http://ao.sa.dk/LAView/ImageServer/Service1.asmx?WSDL sa.dk 130.225.250.114 Denmark 2195847794 AS1835 Forskningsnettet - Danish network for Research and Education 56.17 10.0426

1024 http://www.validate.sbi.dk/Be06Service.asmx?wsdl sbi.dk 130.225.195.38 Denmark 2195833638 AS1835 Forskningsnettet - Danish network for Research and Education 56.17 10.0426

1025 http://sms.site-test.dk/RC/service.asmx?wsdl site-test.dk null Denmark 0 null null null

1026 http://www.skybrud.dk/webservice/frontendservice.asmx?WSDL skybrud.dk 81.95.241.105 Denmark 1365242217 AS42418 Hostnordic A/S 55.3935 10.3949

1027 http://wwwdata.sologstrand.dk/webservice/ServiceHouseId.asmx?wsdl sologstrand.dk 195.41.183.162 Denmark 3274291106 AS3292 TDC Data Networks 56 10

1028 http://d4710185.u63.surftown.dk/Advertising.asmx?WSDL surftown.dk 212.97.133.78 Denmark 3563160910 AS9120 Cohaesio A/S 55.6667 12.5833

1029 http://todoho.dk/gwstat3d_ws_2_0.asmx?WSDL todoho.dk null Denmark 0 null null null

1030 http://www.seescyt.gov.do/_vti_bin/SharepointEmailWS.asmx?wsdl seescyt.gov.do 200.88.113.252 Dominican Republic 3361239548 AS6400 Compa?a Dominicana de TelÒSonos, C. por A. - CODETEL 18.4667 -69.9

1031 http://wss3.seescyt.gov.do/_vti_bin/lists.asmx?WSDL seescyt.gov.do 200.88.113.252 Dominican Republic 3361239548 AS6400 Compa?a Dominicana de TelÒSonos, C. por A. - CODETEL 18.4667 -69.9

1032 http://www.seescyt.gov.do/_vti_bin/People.asmx?wsdl seescyt.gov.do 200.88.113.252 Dominican Republic 3361239548 AS6400 Compa?a Dominicana de TelÒSonos, C. por A. - CODETEL 18.4667 -69.9

1033 http://www.seescyt.gov.do/_vti_bin/Authentication.asmx?wsdl seescyt.gov.do 200.88.113.252 Dominican Republic 3361239548 AS6400 Compa?a Dominicana de TelÒSonos, C. por A. - CODETEL 18.4667 -69.9

1034 http://81.10.16.210/UCProductWebServiceAPP/UCProductWebService.asmx?WSDL 81.10.16.210 81.10.16.210 Egypt 1359614162 AS8452 TE-AS 30.05 31.25

1035 http://www.reform.ee/ReformExport.php?wsdl reform.ee 87.98.44.234 Estonia 1466051818 AS3327 Linxtelecom 58.3661 26.7361

1036 http://www.gasum.com/_vti_bin/BusinessDataCatalog.asmx?wsdl gasum.com null Finland 0 null null null

1037 http://www.gasum.com/_vti_bin/Authentication.asmx?wsdl gasum.com null Finland 0 null null null

1038 http://www.gasum.com/_vti_bin/People.asmx?wsdl gasum.com null Finland 0 null null null

1039 http://www.gasum.fi/_vti_bin/BusinessDataCatalog.asmx?wsdl gasum.fi null Finland 0 null null null

1040 http://www.gasum.fi/_vti_bin/Authentication.asmx?wsdl gasum.fi null Finland 0 null null null

1041 http://www.gasum.fi/_vti_bin/People.asmx?wsdl gasum.fi null Finland 0 null null null

1042 http://lutzstefan.dyndns.org/WebServiceTest/Service1.asmx?WSDL dyndns.org 204.13.248.116 Switzerland 3423467636 AS33517 Dynamic Network Services, Inc. 40.7904 -74.0246

1043 http://ubu.math.helsinki.fi:8080/NLG_service/services/NLGTranslator?wsdl helsinki.fi 128.214.222.4 Finland 2161565188 AS1741 FUNET autonomous system 60.1756 24.9342

1044 http://webalt.math.helsinki.fi:8080/NLG_service/services/Version?wsdl helsinki.fi 128.214.222.4 Finland 2161565188 AS1741 FUNET autonomous system 60.1756 24.9342

1045 http://profinderb2b.fonecta.com/WebServices/Selections.asmx?WSDL fonecta.com 94.199.116.116 Finland 1590129780 AS39193 Javerdel Oy 64 26

1046 http://bisqwit.iki.fi/jutut/kuvat/ajaxsoapdemo/demo.wsdl iki.fi 195.140.195.194 Finland 3280782274 AS29432 TREX Tampere Region Exchange Oy 64 26

1047 http://metalmaker.net/metalmaker.asmx?WSDL metalmaker.net 168.63.22.137 Finland 2822706825 null 38 -97

1048 http://profinderb2b.fi/WebServices/Selections.asmx?WSDL profinderb2b.fi 131.207.87.174 Finland 2211403694 AS375 Finnish State Computer Centre 60.1756 24.9342

1049 http://biomoby.org/services/wsdl/iant.toulouse.inra.fr/iANT_filter genopole-toulouse.prd.fr 147.99.108.39 France 2472766503 AS2200 Reseau National de telecommunications pour la Technologie 48.86 2.35

1050 http://biomoby.org/services/wsdl/testing.bioinfo.genopole-toulouse.prd.fr/CGView genopole-toulouse.prd.fr 147.99.108.39 France 2472766503 AS2200 Reseau National de telecommunications pour la Technologie 48.86 2.35

1051 http://biomoby.org/services/wsdl/iant.toulouse.inra.fr/iANT_tmpred genopole-toulouse.prd.fr 147.99.108.39 France 2472766503 AS2200 Reseau National de telecommunications pour la Technologie 48.86 2.35

1052 http://biomoby.org/services/wsdl/iant.toulouse.inra.fr/iANT_seg genopole-toulouse.prd.fr 147.99.108.39 France 2472766503 AS2200 Reseau National de telecommunications pour la Technologie 48.86 2.35

1053 http://biomoby.org/services/wsdl/testing.bioinfo.genopole-toulouse.prd.fr/Gbk2CGView genopole-toulouse.prd.fr 147.99.108.39 France 2472766503 AS2200 Reseau National de telecommunications pour la Technologie 48.86 2.35

1054 http://biomoby.org/services/wsdl/test_toppred.toulouse.inra.fr/protpars genopole-toulouse.prd.fr 147.99.108.39 France 2472766503 AS2200 Reseau National de telecommunications pour la Technologie 48.86 2.35

1055 http://biomoby.org/services/wsdl/iant.toulouse.inra.fr/iANTMultalinNucleic inra.fr 178.237.110.205 France 3001904845 null 48.86 2.35

1056 http://biomoby.org/services/wsdl/lipm-bioinfo.toulouse.inra.fr/EMBOSS_vectorstrip inra.fr 178.237.110.205 France 3001904845 null 48.86 2.35

1057 http://biomoby.org/services/wsdl/iant.toulouse.inra.fr/iANTXmlToAccessions inra.fr 178.237.110.205 France 3001904845 null 48.86 2.35

1058 http://biomoby.org/services/wsdl/iant.toulouse.inra.fr/iANTPatScan inra.fr 178.237.110.205 France 3001904845 null 48.86 2.35

1059 http://biomoby.org/services/wsdl/lipm-bioinfo.toulouse.inra.fr/EMBOSS_water inra.fr 178.237.110.205 France 3001904845 null 48.86 2.35

1060 http://biomoby.org/services/wsdl/lipm-bioinfo.toulouse.inra.fr/FASTA_AA_multiToFASTA_AACollection inra.fr 178.237.110.205 France 3001904845 null 48.86 2.35

1061 http://biomoby.org/services/wsdl/lipm-bioinfo.toulouse.inra.fr/EMBOSS_transeq inra.fr 178.237.110.205 France 3001904845 null 48.86 2.35

1062 http://biomoby.org/services/wsdl/iant.toulouse.inra.fr/iANTBlastFromXml inra.fr 178.237.110.205 France 3001904845 null 48.86 2.35

1063 http://biomoby.org/services/wsdl/iant.toulouse.inra.fr/iANTSortEntries inra.fr 178.237.110.205 France 3001904845 null 48.86 2.35

1064 http://biomoby.org/services/wsdl/iant.toulouse.inra.fr/iANT_entry_dbToFasta inra.fr 178.237.110.205 France 3001904845 null 48.86 2.35

1065 http://biomoby.org/services/wsdl/lipm-bioinfo.toulouse.inra.fr/ListToStringCollection inra.fr 178.237.110.205 France 3001904845 null 48.86 2.35

1066 http://biomoby.org/services/wsdl/iant.toulouse.inra.fr/iANTSortFeatures inra.fr 178.237.110.205 France 3001904845 null 48.86 2.35

1067 http://biomoby.org/services/wsdl/lipm-bioinfo.toulouse.inra.fr/ObjectCollectionToStringCollection inra.fr 178.237.110.205 France 3001904845 null 48.86 2.35

1068 http://biomoby.org/services/wsdl/iant.toulouse.inra.fr/BlastToIds inra.fr 178.237.110.205 France 3001904845 null 48.86 2.35

1069 http://biomoby.org/services/wsdl/lipm-bioinfo.toulouse.inra.fr/StringToList_Text inra.fr 178.237.110.205 France 3001904845 null 48.86 2.35

1070 http://biomoby.org/services/wsdl/lipm-bioinfo.toulouse.inra.fr/MsfToPhylip inra.fr 178.237.110.205 France 3001904845 null 48.86 2.35

1071 http://biomoby.org/services/wsdl/iant.toulouse.inra.fr/iANTMultalinProteic inra.fr 178.237.110.205 France 3001904845 null 48.86 2.35

1072 http://biomoby.org/services/wsdl/iant.toulouse.inra.fr/BlastToList inra.fr 178.237.110.205 France 3001904845 null 48.86 2.35

1073 http://biomoby.org/services/wsdl/iant.toulouse.inra.fr/iANTFastaReformat inra.fr 178.237.110.205 France 3001904845 null 48.86 2.35

1074 http://biomoby.org/services/wsdl/lipm-bioinfo.toulouse.inra.fr/MsfToPsiblast inra.fr 178.237.110.205 France 3001904845 null 48.86 2.35

1075 http://biomoby.org/services/wsdl/iant.toulouse.inra.fr/iANTautoSNP inra.fr 178.237.110.205 France 3001904845 null 48.86 2.35

1076 http://biomoby.org/services/wsdl/iant.toulouse.inra.fr/iANT_entryToFasta inra.fr 178.237.110.205 France 3001904845 null 48.86 2.35

1077 http://biomoby.org/services/wsdl/lipm-bioinfo.toulouse.inra.fr/iANT_entry_dbToiANT_entryCollection inra.fr 178.237.110.205 France 3001904845 null 48.86 2.35

1078 http://biomoby.org/services/wsdl/iant.toulouse.inra.fr/BlastToLocus_tags inra.fr 178.237.110.205 France 3001904845 null 48.86 2.35

1079 http://biomoby.org/services/wsdl/lipm-bioinfo.toulouse.inra.fr/ObjectCollectionToList inra.fr 178.237.110.205 France 3001904845 null 48.86 2.35

1080 http://biomoby.org/services/wsdl/lipm-bioinfo.toulouse.inra.fr/FASTA_NA_multiToFASTA_NACollection inra.fr 178.237.110.205 France 3001904845 null 48.86 2.35

1081 http://www.hametbenoit.info/_vti_bin/SharepointEmailWS.asmx?wsdl hametbenoit.info 81.64.183.192 France 1363195840 AS21502 NUMERICABLE is a cable network operator in France, offering TV,VOICE and Internet services 48.8667 2.3333

1082 http://www.hametbenoit.info/_vti_bin/BusinessDataCatalog.asmx?wsdl hametbenoit.info 81.64.183.192 France 1363195840 AS21502 NUMERICABLE is a cable network operator in France, offering TV,VOICE and Internet services 48.8667 2.3333

1083 http://www.hametbenoit.info/_vti_bin/People.asmx?wsdl hametbenoit.info 81.64.183.192 France 1363195840 AS21502 NUMERICABLE is a cable network operator in France, offering TV,VOICE and Internet services 48.8667 2.3333

1084 http://www.hametbenoit.info/_vti_bin/Authentication.asmx?wsdl hametbenoit.info 81.64.183.192 France 1363195840 AS21502 NUMERICABLE is a cable network operator in France, offering TV,VOICE and Internet services 48.8667 2.3333

1085 http://inspire.brgm.fr/_vti_bin/BusinessDataCatalog.asmx?wsdl brgm.fr null France 0 null null null

1086 http://inspire.brgm.fr/_vti_bin/Authentication.asmx?wsdl brgm.fr null France 0 null null null

1087 http://inspire.brgm.fr/_vti_bin/People.asmx?wsdl brgm.fr null France 0 null null null

1088 http://www.cafepedagogique.net/_vti_bin/BusinessDataCatalog.asmx?wsdl cafepedagogique.net 85.90.49.60 United Kingdom 1431974204 AS39116 Telehouse Inter. Corp. of Europe Ltd As Number 48.5476 2.399

1089 http://www.cafepedagogique.net/_vti_bin/SharepointEmailWS.asmx?wsdl cafepedagogique.net 85.90.49.60 United Kingdom 1431974204 AS39116 Telehouse Inter. Corp. of Europe Ltd As Number 48.5476 2.399

1090 http://www.cafepedagogique.net/_vti_bin/People.asmx?wsdl cafepedagogique.net 85.90.49.60 United Kingdom 1431974204 AS39116 Telehouse Inter. Corp. of Europe Ltd As Number 48.5476 2.399

1091 http://www.cafepedagogique.net/_vti_bin/Authentication.asmx?wsdl cafepedagogique.net 85.90.49.60 United Kingdom 1431974204 AS39116 Telehouse Inter. Corp. of Europe Ltd As Number 48.5476 2.399

1092 http://www.demosaustralia.com/_vti_bin/BusinessDataCatalog.asmx?wsdl demosaustralia.com 217.33.26.208 France 3642825424 AS2856 BTnet UK Regional network 50.3964 -4.1386

1093 http://www.demosaustralia.com/_vti_bin/People.asmx?wsdl demosaustralia.com 217.33.26.208 France 3642825424 AS2856 BTnet UK Regional network 50.3964 -4.1386

1094 http://www.demosaustralia.com/_vti_bin/Authentication.asmx?wsdl demosaustralia.com 217.33.26.208 France 3642825424 AS2856 BTnet UK Regional network 50.3964 -4.1386

1095 http://www.demos.fr/_vti_bin/BusinessDataCatalog.asmx?wsdl demos.fr 195.5.203.66 France 3271936834 AS13193 Nerim SAS 48.8667 2.3333

1096 http://www.demos.fr/_vti_bin/People.asmx?wsdl demos.fr 195.5.203.66 France 3271936834 AS13193 Nerim SAS 48.8667 2.3333

1097 http://www.demos.fr/_vti_bin/Authentication.asmx?wsdl demos.fr 195.5.203.66 France 3271936834 AS13193 Nerim SAS 48.8667 2.3333

1098 http://www.demosgroup.com/_vti_bin/BusinessDataCatalog.asmx?wsdl demosgroup.com 93.17.234.74 France 1561455178 AS12626 AS12626 9TELECOM 48.7939 2.4932

1099 http://www.demosgroup.com/_vti_bin/Authentication.asmx?wsdl demosgroup.com 93.17.234.74 France 1561455178 AS12626 AS12626 9TELECOM 48.7939 2.4932

1100 http://www.demosgroup.com/_vti_bin/People.asmx?wsdl demosgroup.com 93.17.234.74 France 1561455178 AS12626 AS12626 9TELECOM 48.7939 2.4932

1101 http://www.revue-d-etudes.fr/_vti_bin/BusinessDataCatalog.asmx?wsdl revue-d-etudes.fr 93.17.234.74 France 1561455178 AS12626 AS12626 9TELECOM 48.7939 2.4932

1102 http://www.revue-d-etudes.fr/_vti_bin/People.asmx?wsdl revue-d-etudes.fr 93.17.234.74 France 1561455178 AS12626 AS12626 9TELECOM 48.7939 2.4932

1103 http://www.revue-d-etudes.fr/_vti_bin/Authentication.asmx?wsdl revue-d-etudes.fr 93.17.234.74 France 1561455178 AS12626 AS12626 9TELECOM 48.7939 2.4932

1104 http://www.slformation.com/_vti_bin/People.asmx?wsdl slformation.com 88.190.219.31 France 1488902943 AS12322 Free SAS 48.8667 2.3333

1105 http://www.slformation.com/_vti_bin/Authentication.asmx?wsdl slformation.com 88.190.219.31 France 1488902943 AS12322 Free SAS 48.8667 2.3333

1106 http://www.viamichelin.com/ws/services/Version?wsdl viamichelin.com 212.11.63.254 France 3557507070 AS4589 Easynet Global Services 48.86 2.35

1107 http://gbio-pbil.ibcp.fr/ws/PcProfWS.wsdl ibcp.fr 193.51.160.243 France 3241386227 AS2200 Reseau National de telecommunications pour la Technologie 48.86 2.35

1108 http://gbio-pbil.ibcp.fr/ws/ClustalwWS.wsdl ibcp.fr 193.51.160.243 France 3241386227 AS2200 Reseau National de telecommunications pour la Technologie 48.86 2.35

1109 http://gbio-pbil.ibcp.fr/ws/PredatorWS.wsdl ibcp.fr 193.51.160.243 France 3241386227 AS2200 Reseau National de telecommunications pour la Technologie 48.86 2.35

1110 http://gbio-pbil.ibcp.fr/ws/Simpa96WS.wsdl ibcp.fr 193.51.160.243 France 3241386227 AS2200 Reseau National de telecommunications pour la Technologie 48.86 2.35

1111 http://gbio-pbil.ibcp.fr/ws/GorIVWS.wsdl ibcp.fr 193.51.160.243 France 3241386227 AS2200 Reseau National de telecommunications pour la Technologie 48.86 2.35

1112 http://gbio-pbil.ibcp.fr/ws/GorIIIWS.wsdl ibcp.fr 193.51.160.243 France 3241386227 AS2200 Reseau National de telecommunications pour la Technologie 48.86 2.35

1113 http://gbio-pbil.ibcp.fr/ws/GorIWS.wsdl ibcp.fr 193.51.160.243 France 3241386227 AS2200 Reseau National de telecommunications pour la Technologie 48.86 2.35

1114 http://gbio-pbil.ibcp.fr/ws/DscWS.wsdl ibcp.fr 193.51.160.243 France 3241386227 AS2200 Reseau National de telecommunications pour la Technologie 48.86 2.35

1115 http://gbio-pbil.ibcp.fr/ws/MultalinWS.wsdl ibcp.fr 193.51.160.243 France 3241386227 AS2200 Reseau National de telecommunications pour la Technologie 48.86 2.35

1116 http://gbio-pbil.ibcp.fr/ws/FastaWS.wsdl ibcp.fr 193.51.160.243 France 3241386227 AS2200 Reseau National de telecommunications pour la Technologie 48.86 2.35

1117 http://gbio-pbil.ibcp.fr/ws/SSearchWS.wsdl ibcp.fr 193.51.160.243 France 3241386227 AS2200 Reseau National de telecommunications pour la Technologie 48.86 2.35

1118 http://gbio-pbil.ibcp.fr/ws/BlastWS.wsdl ibcp.fr 193.51.160.243 France 3241386227 AS2200 Reseau National de telecommunications pour la Technologie 48.86 2.35

1119 http://gbio-pbil.ibcp.fr/ws/ConsensusWS.wsdl ibcp.fr 193.51.160.243 France 3241386227 AS2200 Reseau National de telecommunications pour la Technologie 48.86 2.35

1120 http://gbio-pbil.ibcp.fr/ws/CoilsWS.wsdl ibcp.fr 193.51.160.243 France 3241386227 AS2200 Reseau National de telecommunications pour la Technologie 48.86 2.35

1121 http://synergiciel.cecam.net/Cecam.SynergicielV2.Ws/Ingenieur.asmx?WSDL cecam.net null France 0 null null null

1122 http://synergiciel.cecam.net/Cecam.SynergicielV2.Ws/Wsession.asmx?WSDL cecam.net null France 0 null null null

1123 http://cdsws.u-strasbg.fr/axis/services/VizieR?wsdl u-strasbg.fr null France 0 null null null

1124 http://cdsws.u-strasbg.fr/axis/services/UCDResolver?wsdl u-strasbg.fr null France 0 null null null

1125 http://cdsws.u-strasbg.fr/axis/services/VizieRBeta?wsdl u-strasbg.fr null France 0 null null null

1126 http://cdsws.u-strasbg.fr/axis/services/Version?wsdl u-strasbg.fr null France 0 null null null

1127 http://cdsws.u-strasbg.fr/axis/UCDList.jws?wsdl u-strasbg.fr null France 0 null null null

1128 http://cdsws.u-strasbg.fr/axis/services/UCD?wsdl u-strasbg.fr null France 0 null null null

1129 http://cdsws.u-strasbg.fr/axis/services/Sesame?wsdl u-strasbg.fr null France 0 null null null

1130 http://www.icare-service.com/axis/services/Version?wsdl icare-service.com null France 0 null null null

1131 http://www.gpomaster.com/_vti_bin/Lists.asmx?wsdl gpomaster.com null France 0 null null null

1132 http://quisque.com/fr/chasses/crypto/cesar.asmx?WSDL quisque.com 213.246.49.120 France 3589681528 AS21409 IKOULA European Backbone AS 48.86 2.35

1133 http://quisque.com/fr/chasses/crypto/hill.asmx?WSDL quisque.com 213.246.49.120 France 3589681528 AS21409 IKOULA European Backbone AS 48.86 2.35

1134 http://quisque.com/fr/chasses/crypto/vigenere1.asmx?WSDL quisque.com 213.246.49.120 France 3589681528 AS21409 IKOULA European Backbone AS 48.86 2.35

1135 http://quisque.com/fr/chasses/blasons/search.asmx?WSDL quisque.com 213.246.49.120 France 3589681528 AS21409 IKOULA European Backbone AS 48.86 2.35

1136 http://quisque.com/fr/techno/eqImage/eqimage.asmx?WSDL quisque.com 213.246.49.120 France 3589681528 AS21409 IKOULA European Backbone AS 48.86 2.35

1137 http://www.bourse-immobilier.fr/Agences.asmx?WSDL bourse-immobilier.fr null France 0 null null null

1138 http://biomoby.org/services/wsdl/www.legoo.org/LegooNicknamesSearch legoo.org 147.99.102.41 France 2472764969 AS2200 Reseau National de telecommunications pour la Technologie 43.5278 1.4824

1139 http://biomoby.org/services/wsdl/www.legoo.org/LegooBlast legoo.org 147.99.102.41 France 2472764969 AS2200 Reseau National de telecommunications pour la Technologie 43.5278 1.4824

1140 http://biomoby.org/services/wsdl/www.legoo.org/MensGetExpressionPattern legoo.org 147.99.102.41 France 2472764969 AS2200 Reseau National de telecommunications pour la Technologie 43.5278 1.4824

1141 http://www.looneo.fr/WebServices/ExistsMember.asmx?WSDL looneo.fr 93.93.184.204 France 1566423244 AS34235 AS for ASPSERVEUR - La Ciotat, France 43.176 5.6079

1142 http://www.looneo.fr/WebServices/TrackingService.asmx?WSDL looneo.fr 93.93.184.204 France 1566423244 AS34235 AS for ASPSERVEUR - La Ciotat, France 43.176 5.6079

1143 http://www.looneo.fr/WebServices/FullTextSearch.asmx?WSDL looneo.fr 93.93.184.204 France 1566423244 AS34235 AS for ASPSERVEUR - La Ciotat, France 43.176 5.6079

1144 http://www.memo.fr/Partenaires/MWLavService.asmx?WSDL memo.fr 213.11.172.215 France 3574312151 AS702 Verizon Business EMEA - Commercial IP service provider in Europe 48.86 2.35

1145 http://www.memo.fr/Partenaires/MWInfoDecService.asmx?WSDL memo.fr 213.11.172.215 France 3574312151 AS702 Verizon Business EMEA - Commercial IP service provider in Europe 48.86 2.35

1146 http://www.memo.fr/Partenaires/MWGroupeService.asmx?WSDL memo.fr 213.11.172.215 France 3574312151 AS702 Verizon Business EMEA - Commercial IP service provider in Europe 48.86 2.35

1147 http://www.memo.fr/Partenaires/MWResaService.asmx?WSDL memo.fr 213.11.172.215 France 3574312151 AS702 Verizon Business EMEA - Commercial IP service provider in Europe 48.86 2.35

1148 http://gwladysg.free.fr/services/serveur/service_methode.php?wsdl free.fr 212.27.48.10 France 3558551562 AS12322 Free SAS 48.86 2.35

1149 http://gwladysg.free.fr/services/serveur/ConstruireUnModeleDesCasUtilisationGraphiquement_serveur.php?wsdl free.fr 212.27.48.10 France 3558551562 AS12322 Free SAS 48.86 2.35

1150 http://biomoby.org/services/wsdl/www.heliagene.org/HeliageneGetEntry heliagene.org 147.99.102.41 France 2472764969 AS2200 Reseau National de telecommunications pour la Technologie 43.5278 1.4824

1151 http://biomoby.org/services/wsdl/www.heliagene.org/HeliageneBlastFromXml heliagene.org 147.99.102.41 France 2472764969 AS2200 Reseau National de telecommunications pour la Technologie 43.5278 1.4824

1152 http://biomoby.org/services/wsdl/www.heliagene.org/HeliageneBlast heliagene.org 147.99.102.41 France 2472764969 AS2200 Reseau National de telecommunications pour la Technologie 43.5278 1.4824

1153 http://biomoby.org/services/wsdl/www.heliagene.org/HeliageneGetSequence heliagene.org 147.99.102.41 France 2472764969 AS2200 Reseau National de telecommunications pour la Technologie 43.5278 1.4824

1154 http://www.mescorrespondances.fr/WebServices/RelationManager.asmx?WSDL mescorrespondances.fr 62.210.150.161 France 1053988513 AS12322 Free SAS 48.86 2.35

1155 http://www.mescorrespondances.fr/WebServices/NewsService.asmx?WSDL mescorrespondances.fr 62.210.150.161 France 1053988513 AS12322 Free SAS 48.86 2.35

1156 http://www.mescorrespondances.fr/WebServices/SuggEnLigne.asmx?WSDL mescorrespondances.fr 62.210.150.161 France 1053988513 AS12322 Free SAS 48.86 2.35

1157 http://www.mescorrespondances.fr/WebServices/AutoCompletePseudo.asmx?WSDL mescorrespondances.fr 62.210.150.161 France 1053988513 AS12322 Free SAS 48.86 2.35

1158 http://www.partenairedejeu.fr/WebServices/RelationManager.asmx?WSDL partenairedejeu.fr 62.210.150.161 France 1053988513 AS12322 Free SAS 48.86 2.35

1159 http://www.partenairedejeu.fr/WebServices/AutoCompletePseudo.asmx?WSDL partenairedejeu.fr 62.210.150.161 France 1053988513 AS12322 Free SAS 48.86 2.35

1160 http://www.partenairedejeu.fr/WebServices/NewsService.asmx?WSDL partenairedejeu.fr 62.210.150.161 France 1053988513 AS12322 Free SAS 48.86 2.35

1161 http://www.partenairedejeu.fr/WebServices/SuggEnLigne.asmx?WSDL partenairedejeu.fr 62.210.150.161 France 1053988513 AS12322 Free SAS 48.86 2.35

1162 http://ws.c2r-bourgogne.org/services/Version?wsdl c2r-bourgogne.org 217.109.10.133 France 3647801989 AS3215 France Telecom - Orange 48.86 2.35

1163 http://wsshipping.chronopost.fr/wsQuickcost/services/ServiceQuickCost?wsdl chronopost.fr 84.37.93.134 France 1411734918 AS4589 Easynet Global Services 48.7999 2.3326

1164 http://wsshipping.chronopost.fr/shipping/services/services/ServiceEProcurement?wsdl chronopost.fr 84.37.93.134 France 1411734918 AS4589 Easynet Global Services 48.7999 2.3326

1165 http://www.edf-bleuciel.fr/FRONT/EDF_PART/include/fr/webServices/CIA/webServiceCIA.wsdl edf-bleuciel.fr 93.188.170.32 France 1572645408 AS25593 Linkbynet S.A 48.86 2.35

1166 http://www.edf-bleuciel.fr/FRONT/EDF_PART/include/fr/webServices/CIB/webServiceCIB.wsdl edf-bleuciel.fr 93.188.170.32 France 1572645408 AS25593 Linkbynet S.A 48.86 2.35

1167 http://www.edf-bleuciel.fr/FRONT/EDF_PART/include/fr/webServices/CPA/webServiceCPA.wsdl edf-bleuciel.fr 93.188.170.32 France 1572645408 AS25593 Linkbynet S.A 48.86 2.35

1168 http://particuliers.edf.fr/FRONT/EDF_PART/include/fr/webServices/CPA/webServiceCPA.wsdl edf.fr 93.188.170.32 France 1572645408 AS25593 Linkbynet S.A 48.86 2.35

1169 http://particuliers.edf.fr/FRONT/EDF_PART/include/fr/webServices/CIA/webServiceCIA.wsdl edf.fr 93.188.170.32 France 1572645408 AS25593 Linkbynet S.A 48.86 2.35

1170 http://stats.oecd.org/OECDStatWS_Authentication/OECDStatWS_Authentication.asmx?WSDL oecd.org null France 0 null null null

1171 http://triptoyou.com/MWGroupeService.asmx?WSDL triptoyou.com 213.11.172.100 France 3574312036 AS702 Verizon Business EMEA - Commercial IP service provider in Europe 48.86 2.35

1172 http://www.triptoyou.com/MWLavService.asmx?WSDL triptoyou.com 213.11.172.100 France 3574312036 AS702 Verizon Business EMEA - Commercial IP service provider in Europe 48.86 2.35

1173 http://www.triptoyou.com/MWMyPageService.asmx?WSDL triptoyou.com 213.11.172.100 France 3574312036 AS702 Verizon Business EMEA - Commercial IP service provider in Europe 48.86 2.35

1174 http://195.6.140.178/ws/ModbusXmlDa?wsdl 195.6.140.178 195.6.140.178 France 3271986354 AS3215 France Telecom - Orange 48.86 2.35

1175 http://ws.dp.advences.com/productList.asmx?WSDL advences.com 194.50.77.20 France 3258076436 AS39606 Advences 48.86 2.35

1176 http://job.advences.com/XML/echo.cfc?wsdl advences.com 194.50.77.20 France 3258076436 AS39606 Advences 48.86 2.35

1177 http://www.cma-cgm.com/App_Ajax/eBusiness/Schedules/ScheduleWebService.asmx?WSDL cma-cgm.com 193.109.119.8 France 3245176584 AS21203 FR-CMA-CGM 43.2854 5.3761

1178 http://www.essec.fr/myessec/services/jws-myessec?wsdl essec.fr 194.254.137.78 France 3271461198 AS2200 Reseau National de telecommunications pour la Technologie 49.0364 2.0761

1179 http://www.mytaratata.com/Artist.asmx?WSDL mytaratata.com 62.23.11.200 France 1041697736 AS8220 COLT Technology Services 51.5 -0.13

1180 http://www.mytaratata.com/MixService.asmx?WSDL mytaratata.com 62.23.11.200 France 1041697736 AS8220 COLT Technology Services 51.5 -0.13

1181 http://vmfr.netsizeonline.com/NsEndUserProFileApi/EndUserProfileService.asmx?WSDL netsizeonline.com 213.41.67.130 France 3576251266 AS8220 COLT Technology Services 51.5 -0.13

1182 http://vm.netsizeonline.com/nsvmpublishingapiV3/nsvmpublishingapiV3.asmx?wsdl netsizeonline.com 213.41.67.130 France 3576251266 AS8220 COLT Technology Services 51.5 -0.13

1183 http://www.thomasbouche.com/WS/Print/WSPrint.asmx?WSDL thomasbouche.com 62.39.253.247 France 1042808311 AS15557 NEUF CEGETEL (formerly LDCOM NETWORKS) 48.9117 2.2847

1184 http://80.118.147.154/WS-orderStatut/serveur/WS-Statut-Order-Compiere-Web.php?wsdl 80.118.147.154 80.118.147.154 France 1349948314 AS15557 NEUF CEGETEL (formerly LDCOM NETWORKS) 48.86 2.35

1185 http://www.appeldulivre.com/ws/adl_service.wsdl appeldulivre.fr 217.109.18.66 France 3647803970 AS3215 France Telecom - Orange 48.86 2.35

1186 http://www.aspfr.com/WS/CSWS.asmx?WSDL aspfr.com 213.251.145.123 France 3590033787 AS16276 OVH OVH 48.86 2.35

1187 http://wsproxy1.b3g-telecom.com/webmethodes/call.wsdl b3g-telecom.com 195.167.195.204 France 3282551756 AS12670 Completel Autonomous System in France 48.86 2.35

1188 http://www.bluturtles.fr/ws/wsbt/wscommunes.asmx?wsdl bluturtles.fr 213.186.33.87 France 3585745239 AS16276 OVH OVH 48.86 2.35

1189 http://partenaires.capitol.fr/tpBsdSoap.php?wsdl capitol.fr null France 0 null null null

1190 http://mifiddatabase.cesr.eu/ws/MifId.asmx?WSDL cesr.eu 217.174.207.108 France 3652112236 AS16128 AGARIK provides WEB and Servers Hosting 48.86 2.35

1191 http://www.cppfrance.com/WS/CSWS.asmx?WSDL cppfrance.com 213.251.145.123 France 3590033787 AS16276 OVH OVH 48.86 2.35

1192 http://cgi.dolist.fr/members/service.asmx?WSDL dolist.fr 185.14.228.31 France 3104760863 null 48.86 2.35

1193 http://www.cerimes.education.fr/hello-service.php?wsdl education.fr 160.92.166.231 France 2690426599 AS8677 Atos Worldline (Atos Origin Group) Autonomous System 48.86 2.35

1194 http://www.e-leclerc.com/leclercappnet/wEnergeo/mainEnergeo.asmx?WSDL e-leclerc.com 91.213.242.230 France 1540748006 AS39542 SIGMA-INFORMATIQUE 47.4131 -1.9686

1195 http://rangiroa.essi.fr:8080/dotnet/mon-premier-web-service.asmx?wsdl essi.fr null France 0 null null null

1196 http://www.explore.fr/businessimmo/Webservices/WsBusinessImmo.asmx?wsdl explore.fr null France 0 null null null

1197 http://api.eyeka.com/api/0.04/wsdl eyeka.com 193.164.150.129 France 3248789121 AS31216 BSO Communication Network 48.86 2.35

1198 http://maquette.gecip.net/vivezlightws/service.asmx?wsdl gecip.net null France 0 null null null

1199 http://www.globe-access.fr/service1.asmx?WSDL globe-access.fr 94.247.177.56 France 1593291064 AS41186 AZURA NETWORKS 48.86 2.35

1200 http://www.handicap.fr/server_hanproducts.php?wsdl handicap.fr 188.165.34.178 France 3164938930 AS16276 OVH OVH 48.86 2.35

1201 http://ic.hetic.net/hetic.wsdl hetic.net 213.186.33.17 France 3585745169 AS16276 OVH OVH 48.86 2.35

1202 http://www.kiosque-edu.com/knewebservice2/knews.asmx?WSDL kiosque-edu.com 195.81.225.150 France 3276923286 AS8928 Interoute Communications Ltd 51.5 -0.13

1203 http://igsee.l9c.org/soap.php?wsdl l9c.org 109.234.161.12 France 1844093196 AS50474 o2switch SARL 48.86 2.35

1204 http://wbs.logi-pro.net/wslogipro.asmx?wsdl logi-pro.net 213.246.47.3 France 3589680899 AS21409 IKOULA European Backbone AS 48.86 2.35

1205 http://www.loreal.fr/_fr/_fr/WS/rss.asmx?WSDL loreal.fr 193.67.161.217 France 3242435033 AS702 Verizon Business EMEA - Commercial IP service provider in Europe 52.35 4.9167

1206 http://www.micropaiement.com/RestrictedCheckTickets.asmx?wsdl micropaiement.com 194.0.255.28 France 3254845212 null 48.86 2.35

1207 http://www.mmm-maintenance.com/service/webservice1/service1.asmx?WSDL mmm-maintenance.com null France 0 null null null

1208 http://www.msfrancedev.net/TechDays2007.asmx?WSDL msfrancedev.net 85.31.208.159 France 1428148383 AS34235 AS for ASPSERVEUR - La Ciotat, France 51.5 -0.13

1209 http://nesapp01.nes-france.com/ws/cdiscount?wsdl nes-france.com 81.252.75.141 France 1375488909 AS3215 France Telecom - Orange 47.8318 1.9667

1210 http://testv2.netassur.fr/WebServices/WebHyperAssur.asmx?WSDL netassur.fr 46.105.55.166 France 778647462 null 48.86 2.35

1211 http://blog.odelmotte.fr/api/BlogImporter.asmx?WSDL odelmotte.fr 213.246.49.115 France 3589681523 AS21409 IKOULA European Backbone AS 48.86 2.35

1212 http://www.openesub.org/jeu/asynchrone/site/soap/soap.php?wsdl openesub.org 91.121.90.108 France 1534679660 AS16276 OVH OVH 48.86 2.35

1213 http://www.opsys.fr/WebSrvAloes160/ServiceRecherche.asmx?WSDL opsys.fr null France 0 null null null

1214 http://www.pdafr.com/WS/CSWS.asmx?WSDL pdafr.com 213.251.145.123 France 3590033787 AS16276 OVH OVH 48.86 2.35

1215 http://ws.rte-com.com/rtecomwebservice.asmx?WSDL rte-com.com null France 0 null null null

1216 http://www.supplychainserver.com/webservices/CommuneInfoservice.asmx?wsdl supplychainserver.com 62.39.109.51 France 1042771251 AS15557 NEUF CEGETEL (formerly LDCOM NETWORKS) 48.86 2.35

1217 http://cdt47.tourinsoft.com/soft/RechercheDynamique/Syndication/controle/syndication.asmx?WSDL tourinsoft.com 195.95.168.50 France 3277826098 AS35625 ATE AVENIR-TELEMATIQUE 48.86 2.35

1218 http://www.unit.eu/ori-oai-vocabulary/xfire/OriVocabularyService?WSDL unit.eu null France 0 null null null

1219 http://mobile.vidal.fr/backend/service.wsdl vidal.fr 89.107.171.250 France 1500228602 AS25593 Linkbynet S.A 48.86 2.35

1220 http://www.xdcpro.com/src/ws/PropertyDescription/Propertydescription.asmx?WSDL xdcpro.com 89.185.33.188 France 1505305020 AS8426 ClaraNET 48.86 2.35

1221 http://www.yataka.com/NSVMCachingAPI/NSVMCachingAPI.asmx?WSDL yataka.com null France 0 null null null

1222 http://biomoby.org/services/wsdl/mpiz-koeln.mpg.de/get_other_database_accessions_by_database_accession mpg.de 134.76.31.209 Germany 2253135825 AS680 service G-WiN 51.5333 9.9333

1223 http://biomoby.org/services/wsdl/mpiz-koeln.mpg.de/Blast_Against_RefSeq_Complete_Sequenced_Organisms mpg.de 134.76.31.209 Germany 2253135825 AS680 service G-WiN 51.5333 9.9333

1224 http://biomoby.org/services/wsdl/mpiz-koeln.mpg.de/getAFAWEProteinIDByGOTerm mpg.de 134.76.31.209 Germany 2253135825 AS680 service G-WiN 51.5333 9.9333

1225 http://biomoby.org/services/wsdl/mpiz-koeln.mpg.de/EBI_InterproScan mpg.de 134.76.31.209 Germany 2253135825 AS680 service G-WiN 51.5333 9.9333

1226 http://biomoby.org/services/wsdl/mpiz-koeln.mpg.de/getAutomaticAndManualAnnotationByAFAWE_ID mpg.de 134.76.31.209 Germany 2253135825 AS680 service G-WiN 51.5333 9.9333

1227 http://biomoby.org/services/wsdl/mpiz-koeln.mpg.de/runAFAWEAnalysesBySequenceAndOrganismAndGetAFAWE_URL mpg.de 134.76.31.209 Germany 2253135825 AS680 service G-WiN 51.5333 9.9333

1228 http://biomoby.org/services/wsdl/mpiz-koeln.mpg.de/get_go_information_by_go_term mpg.de 134.76.31.209 Germany 2253135825 AS680 service G-WiN 51.5333 9.9333

1229 http://biomoby.org/services/wsdl/mpiz-koeln.mpg.de/EBI_WU_Blast mpg.de 134.76.31.209 Germany 2253135825 AS680 service G-WiN 51.5333 9.9333

1230 http://biomoby.org/services/wsdl/mpiz-koeln.mpg.de/getAFAWEProteinIDBySequenceAndOrganism mpg.de 134.76.31.209 Germany 2253135825 AS680 service G-WiN 51.5333 9.9333

1231 http://biomoby.org/services/wsdl/mpiz-koeln.mpg.de/buildMultipleAlignmentWithMAFFT mpg.de 134.76.31.209 Germany 2253135825 AS680 service G-WiN 51.5333 9.9333

1232 http://biomoby.org/services/wsdl/mpiz-koeln.mpg.de/GetInAndOrthologsFromRefSeq mpg.de 134.76.31.209 Germany 2253135825 AS680 service G-WiN 51.5333 9.9333

1233 http://biomoby.org/services/wsdl/mpiz-koeln.mpg.de/RunSifter mpg.de 134.76.31.209 Germany 2253135825 AS680 service G-WiN 51.5333 9.9333

1234 http://biomoby.org/services/wsdl/mpiz-koeln.mpg.de/BuildPhylogeneticTreeFromFastaAlignment mpg.de 134.76.31.209 Germany 2253135825 AS680 service G-WiN 51.5333 9.9333

1235 http://biomoby.org/services/wsdl/mpiz-koeln.mpg.de/get_agi_code_by_keyword mpg.de 134.76.31.209 Germany 2253135825 AS680 service G-WiN 51.5333 9.9333

1236 http://biomoby.org/services/wsdl/mpiz-koeln.mpg.de/GetConservedDomainsFromFastaAlignment mpg.de 134.76.31.209 Germany 2253135825 AS680 service G-WiN 51.5333 9.9333

1237 http://biomoby.org/services/wsdl/mpiz-koeln.mpg.de/get_GO_Term_by_Database_ID mpg.de 134.76.31.209 Germany 2253135825 AS680 service G-WiN 51.5333 9.9333

1238 http://www.migenas.mpg.de/webservices/migenasWS/ProteinInfo?wsdl mpg.de 134.76.31.209 Germany 2253135825 AS680 service G-WiN 51.5333 9.9333

1239 http://live.sharepointcommunity.de/_vti_bin/BusinessDataCatalog.asmx?wsdl sharepointcommunity.de 217.110.108.161 Germany 3647892641 AS8220 COLT Technology Services 52.5167 13.4

1240 http://live.sharepointcommunity.de/_vti_bin/People.asmx?wsdl sharepointcommunity.de 217.110.108.161 Germany 3647892641 AS8220 COLT Technology Services 52.5167 13.4

1241 http://live.sharepointcommunity.de/_vti_bin/Authentication.asmx?wsdl sharepointcommunity.de 217.110.108.161 Germany 3647892641 AS8220 COLT Technology Services 52.5167 13.4

1242 http://sps.ikg-rt.de/_vti_bin/People.asmx?wsdl ikg-rt.de null Germany 0 null null null

1243 http://sps.ikg-rt.de/_vti_bin/Authentication.asmx?wsdl ikg-rt.de null Germany 0 null null null

1244 http://www.nteam.de/_vti_bin/BusinessDataCatalog.asmx?wsdl nteam.de 217.70.137.42 Germany 3645278506 AS15366 DNS:NET GmbH & 1st communications GmbH Autonomous System 52.2833 13.6167

1245 http://www.nteam.de/_vti_bin/SharepointEmailWS.asmx?wsdl nteam.de 217.70.137.42 Germany 3645278506 AS15366 DNS:NET GmbH & 1st communications GmbH Autonomous System 52.2833 13.6167

1246 http://www.nteam.de/_vti_bin/Authentication.asmx?wsdl nteam.de 217.70.137.42 Germany 3645278506 AS15366 DNS:NET GmbH & 1st communications GmbH Autonomous System 52.2833 13.6167

1247 http://www.nteam.de/_vti_bin/People.asmx?wsdl nteam.de 217.70.137.42 Germany 3645278506 AS15366 DNS:NET GmbH & 1st communications GmbH Autonomous System 52.2833 13.6167

1248 http://sps.ikg.rt.bw.schule.de/_vti_bin/People.asmx?wsdl schule.de 192.76.176.140 Germany 3226251404 AS680 service G-WiN 52.5167 13.4

1249 http://sps.ikg.rt.bw.schule.de/_vti_bin/Authentication.asmx?wsdl schule.de 192.76.176.140 Germany 3226251404 AS680 service G-WiN 52.5167 13.4

1250 http://blogs.sqlserverfaq.de/_vti_bin/BusinessDataCatalog.asmx?wsdl sqlserverfaq.de 216.8.179.25 Germany 3624448793 AS13727 NEXT DIMENSION INC 42.3188 -82.965

1251 http://blogs.sqlserverfaq.de/_vti_bin/Authentication.asmx?wsdl sqlserverfaq.de 216.8.179.25 Germany 3624448793 AS13727 NEXT DIMENSION INC 42.3188 -82.965

1252 http://blogs.sqlserverfaq.de/_vti_bin/People.asmx?wsdl sqlserverfaq.de 216.8.179.25 Germany 3624448793 AS13727 NEXT DIMENSION INC 42.3188 -82.965

1253 http://wortschatz.uni-leipzig.de:8100/axis/services/Version?wsdl uni-leipzig.de 139.18.1.45 Germany 2333212973 AS680 service G-WiN 51.3 12.3333

1254 http://register.viacom.com/_vti_bin/Authentication.asmx?wsdl viacom.com 206.220.43.92 United States 3470535516 AS7256 Viacom Inc. 40.7605 -73.9933

1255 http://register.viacom.com/_vti_bin/People.asmx?wsdl viacom.com null United States 0 null null null

1256 http://bibiserv.techfak.uni-bielefeld.de/wsdl/roci.wsdl uni-bielefeld.de 129.70.240.4 Germany 2168909828 AS680 service G-WiN 52.0333 8.5333

1257 http://bibiwsserv.techfak.uni-bielefeld.de/RNAhybrid/axis/RNAhybridPort?wsdl uni-bielefeld.de 129.70.240.4 Germany 2168909828 AS680 service G-WiN 52.0333 8.5333

1258 http://bibiserv.techfak.uni-bielefeld.de/wsdl/decomp.wsdl uni-bielefeld.de 129.70.240.4 Germany 2168909828 AS680 service G-WiN 52.0333 8.5333

1259 http://www.hezser.de/_vti_bin/SharepointEmailWS.asmx?wsdl hezser.de 217.91.87.221 Germany 3646642141 AS3320 Deutsche Telekom AG 51 9

1260 http://www.hezser.de/_vti_bin/People.asmx?wsdl hezser.de 217.91.87.221 Germany 3646642141 AS3320 Deutsche Telekom AG 51 9

1261 http://www.hezser.de/_vti_bin/Authentication.asmx?wsdl hezser.de 217.91.87.221 Germany 3646642141 AS3320 Deutsche Telekom AG 51 9

1262 http://www2.mycema.com/_vti_bin/Authentication.asmx?wsdl mycema.com null Germany 0 null null null

1263 http://www2.mycema.com/_vti_bin/People.asmx?wsdl mycema.com null Germany 0 null null null

1264 http://www.mycema.de/_vti_bin/People.asmx?wsdl mycema.de 195.144.22.201 Germany 3281000137 AS15830 TELECITYGROUP INTERNATIONAL LIMITED 51 9

1265 http://www.mycema.de/_vti_bin/Authentication.asmx?wsdl mycema.de 195.144.22.201 Germany 3281000137 AS15830 TELECITYGROUP INTERNATIONAL LIMITED 51 9

1266 http://radio-dialog.de/_vti_bin/Authentication.asmx?wsdl radio-dialog.de 87.106.66.59 Germany 1466581563 AS8560 1&1 Internet AG 51 9

1267 http://radio-dialog.de/_vti_bin/People.asmx?wsdl radio-dialog.de 87.106.66.59 Germany 1466581563 AS8560 1&1 Internet AG 51 9

1268 http://www.serviceportals.de/_vti_bin/SharepointEmailWS.asmx?wsdl serviceportals.de 81.20.82.14 United Kingdom 1360286222 AS25260 QualityHosting AG 51 9

1269 http://www.serviceportals.de/_vti_bin/People.asmx?wsdl serviceportals.de 81.20.82.14 United Kingdom 1360286222 AS25260 QualityHosting AG 51 9

1270 http://www.serviceportals.de/_vti_bin/Authentication.asmx?wsdl serviceportals.de 81.20.82.14 United Kingdom 1360286222 AS25260 QualityHosting AG 51 9

1271 http://hnsp.inf-bb.uni-jena.de:8080/axis/WeaselInvokePriceService.jws?wsdl uni-jena.de null Germany 0 null null null

1272 http://hnsp.inf-bb.uni-jena.de:8080/axis/services/Version?wsdl uni-jena.de null Germany 0 null null null

1273 http://hnsp.inf-bb.uni-jena.de:8080/axis/services/RosettaToDSD2?wsdl uni-jena.de null Germany 0 null null null

1274 http://lcdnl.co.uk/_vti_bin/DspStswsdl.aspx lcdnl.co.uk 212.227.124.24 Germany 3571678232 AS8560 1&1 Internet AG 51 9

1275 http://lcdnl.co.uk/_vti_bin/Imagingwsdl.aspx lcdnl.co.uk 212.227.124.24 Germany 3571678232 AS8560 1&1 Internet AG 51 9

1276 http://lcdnl.co.uk/_vti_bin/DWSwsdl.aspx lcdnl.co.uk 212.227.124.24 Germany 3571678232 AS8560 1&1 Internet AG 51 9

1277 http://lcdnl.co.uk/_vti_bin/Meetingswsdl.aspx lcdnl.co.uk 212.227.124.24 Germany 3571678232 AS8560 1&1 Internet AG 51 9

1278 http://lcdnl.co.uk/_vti_bin/versionswsdl.aspx lcdnl.co.uk 212.227.124.24 Germany 3571678232 AS8560 1&1 Internet AG 51 9

1279 http://lcdnl.co.uk/_vti_bin/UserGroupwsdl.aspx lcdnl.co.uk 212.227.124.24 Germany 3571678232 AS8560 1&1 Internet AG 51 9

1280 http://lcdnl.co.uk/_vti_bin/Formswsdl.aspx lcdnl.co.uk 212.227.124.24 Germany 3571678232 AS8560 1&1 Internet AG 51 9

1281 http://lcdnl.co.uk/_vti_bin/webpartpageswsdl.aspx lcdnl.co.uk 212.227.124.24 Germany 3571678232 AS8560 1&1 Internet AG 51 9

1282 http://lcdnl.co.uk/_vti_bin/Webswsdl.aspx lcdnl.co.uk 212.227.124.24 Germany 3571678232 AS8560 1&1 Internet AG 51 9

1283 http://lcdnl.co.uk/_vti_bin/alertswsdl.aspx lcdnl.co.uk 212.227.124.24 Germany 3571678232 AS8560 1&1 Internet AG 51 9

1284 http://lcdnl.co.uk/_vti_bin/Listswsdl.aspx lcdnl.co.uk 212.227.124.24 Germany 3571678232 AS8560 1&1 Internet AG 51 9

1285 http://lcdnl.co.uk/_vti_bin/Permissionswsdl.aspx lcdnl.co.uk 212.227.124.24 Germany 3571678232 AS8560 1&1 Internet AG 51 9

1286 http://lcdnl.co.uk/_vti_bin/Viewswsdl.aspx lcdnl.co.uk 212.227.124.24 Germany 3571678232 AS8560 1&1 Internet AG 51 9

1287 http://www.zimalimited.co.uk/_vti_bin/Imagingwsdl.aspx zimalimited.co.uk 212.227.124.24 Germany 3571678232 AS8560 1&1 Internet AG 51 9

1288 http://www.zimalimited.co.uk/_vti_bin/versionswsdl.aspx zimalimited.co.uk 212.227.124.24 Germany 3571678232 AS8560 1&1 Internet AG 51 9

1289 http://www.zimalimited.co.uk/_vti_bin/DWSwsdl.aspx zimalimited.co.uk 212.227.124.24 Germany 3571678232 AS8560 1&1 Internet AG 51 9

1290 http://www.zimalimited.co.uk/_vti_bin/Permissionswsdl.aspx zimalimited.co.uk 212.227.124.24 Germany 3571678232 AS8560 1&1 Internet AG 51 9

1291 http://www.zimalimited.co.uk/_vti_bin/Listswsdl.aspx zimalimited.co.uk 212.227.124.24 Germany 3571678232 AS8560 1&1 Internet AG 51 9

1292 http://www.zimalimited.co.uk/_vti_bin/UserGroupwsdl.aspx zimalimited.co.uk 212.227.124.24 Germany 3571678232 AS8560 1&1 Internet AG 51 9

1293 http://www.zimalimited.co.uk/_vti_bin/alertswsdl.aspx zimalimited.co.uk 212.227.124.24 Germany 3571678232 AS8560 1&1 Internet AG 51 9

1294 http://www.zimalimited.co.uk/_vti_bin/Webswsdl.aspx zimalimited.co.uk 212.227.124.24 Germany 3571678232 AS8560 1&1 Internet AG 51 9

1295 http://www.zimalimited.co.uk/_vti_bin/Meetingswsdl.aspx zimalimited.co.uk 212.227.124.24 Germany 3571678232 AS8560 1&1 Internet AG 51 9

1296 http://www.zimalimited.co.uk/_vti_bin/Viewswsdl.aspx zimalimited.co.uk 212.227.124.24 Germany 3571678232 AS8560 1&1 Internet AG 51 9

1297 http://www.zimalimited.co.uk/_vti_bin/webpartpageswsdl.aspx zimalimited.co.uk 212.227.124.24 Germany 3571678232 AS8560 1&1 Internet AG 51 9

1298 http://www.zimalimited.co.uk/_vti_bin/Formswsdl.aspx zimalimited.co.uk 212.227.124.24 Germany 3571678232 AS8560 1&1 Internet AG 51 9

1299 http://www.zimalimited.co.uk/_vti_bin/DspStswsdl.aspx zimalimited.co.uk 212.227.124.24 Germany 3571678232 AS8560 1&1 Internet AG 51 9

1300 http://mathertel.de/AJAXEngine/S01_AsyncSamples/CalcService.asmx?WSDL mathertel.de 188.64.60.91 Germany 3158326363 AS8741 AS - ecore Kommunikations AG 51 9

1301 http://mathertel.de/AJAXEngine/S02_AJAXCoreSamples/OrteLookup.asmx?WSDL mathertel.de 188.64.60.91 Germany 3158326363 AS8741 AS - ecore Kommunikations AG 51 9

1302 http://mathertel.de/AJAXEngine/S03_AJAXControls/ValidatorDemo.asmx?WSDL mathertel.de 188.64.60.91 Germany 3158326363 AS8741 AS - ecore Kommunikations AG 51 9

1303 http://www.mathertel.de/AJAXEngine/S02_AJAXCoreSamples/AJAXComparison.asmx?WSDL mathertel.de 188.64.60.91 Germany 3158326363 AS8741 AS - ecore Kommunikations AG 51 9

1304 http://mathertel.de/AJAXEngine/S03_AJAXControls/CountryLookup.asmx?WSDL mathertel.de 188.64.60.91 Germany 3158326363 AS8741 AS - ecore Kommunikations AG 51 9

1305 http://mathertel.de/AJAXEngine/S03_AJAXControls/CarData.asmx?WSDL mathertel.de 188.64.60.91 Germany 3158326363 AS8741 AS - ecore Kommunikations AG 51 9

1306 http://mathertel.de/AJAXEngine/S06_AJAXForms/OpenAjaxChat.asmx?WSDL mathertel.de 188.64.60.91 Germany 3158326363 AS8741 AS - ecore Kommunikations AG 51 9

1307 http://webservices.affili.net/pdws/ProductDataV1.asmx?WSDL affili.net 195.189.236.35 Germany 3284003875 AS24679 Hostway Deutschland GmbH 51 9

1308 http://cms1.b-es.de/DesktopModules/eContent-Events/AjaxEventCoreService.asmx?WSDL b-es.de 82.100.207.234 Germany 1382338538 AS25394 AS for MK Netzdienste GmbH & Co. KG 51 9

1309 http://fhrg.first.fraunhofer.de:8080/linuxtoolbox/services/Calculator?wsdl fraunhofer.de 192.54.34.244 Germany 3224773364 AS680 service G-WiN 51 9

1310 http://fhrg.first.fraunhofer.de:8080/linuxtoolbox/services/Sort?wsdl fraunhofer.de 192.54.34.244 Germany 3224773364 AS680 service G-WiN 51 9

1311 http://fhrg.first.fraunhofer.de:8080/linuxtoolbox/services/Tail?wsdl fraunhofer.de 192.54.34.244 Germany 3224773364 AS680 service G-WiN 51 9

1312 http://fhrg.first.fraunhofer.de:8080/linuxtoolbox/services/Version?wsdl fraunhofer.de 192.54.34.244 Germany 3224773364 AS680 service G-WiN 51 9

1313 http://fhrg.first.fraunhofer.de:8080/linuxtoolbox/services/Wait?wsdl fraunhofer.de 192.54.34.244 Germany 3224773364 AS680 service G-WiN 51 9

1314 http://service.oli-it.com/StammService.asmx?wsdl oli-it.com 62.112.137.53 Germany 1047562549 AS5464 Netdiscounter GmbH autonomous system 49.4667 10.9667

1315 http://service.oli-it.com/WortraumService.asmx?wsdl oli-it.com 62.112.137.53 Germany 1047562549 AS5464 Netdiscounter GmbH autonomous system 49.4667 10.9667

1316 http://cms.bad-goegging.de/DesktopModules/eContent-Events/AjaxEventCoreService.asmx?WSDL bad-goegging.de 82.100.207.234 Germany 1382338538 AS25394 AS for MK Netzdienste GmbH & Co. KG 51 9

1317 http://cms.tourismus-landkreis-kelheim.de/DesktopModules/eContent-Events/AjaxEventCoreService.asmx?WSDL tourismus-landkreis-kelheim.de 82.100.207.234 Germany 1382338538 AS25394 AS for MK Netzdienste GmbH & Co. KG 51 9

1318 http://econtent.bayerischerjura.de/DesktopModules/eContent-Events/AjaxEventCoreService.asmx?WSDL bayerischerjura.de 188.93.15.79 Germany 3160215375 AS34011 domainfactory GmbH 51 9

1319 http://econtent.bayerischer-wald.de/DesktopModules/eContent-Events/AjaxEventCoreService.asmx?WSDL bayerischer-wald.de 46.51.184.76 Germany 775141452 AS39111 Amazon EU DC AS 53 -8

1320 http://www.ilearn24.net/sphereWebserviceAdapter/services/ExtendedUserDataExchangeService?wsdl ilearn24.net null Germany 0 null null null

1321 http://www.ilearn24.net/sphereWebserviceAdapter/services/urn:UserDataExchangeService?wsdl ilearn24.net null Germany 0 null null null

1322 http://www.ilearn24.net/sphereWebserviceAdapter/services/urn:StatusReportService?wsdl ilearn24.net null Germany 0 null null null

1323 http://www.ilearn24.net/sphereWebserviceAdapter/services/Version?wsdl ilearn24.net null Germany 0 null null null

1324 http://www.ilearn24.net/sphereWebserviceAdapter/services/SOAPMonitorService?wsdl ilearn24.net null Germany 0 null null null

1325 http://cms.tv-keh.de/DesktopModules/eContent-Events/AjaxEventCoreService.asmx?WSDL tv-keh.de 82.100.207.234 Germany 1382338538 AS25394 AS for MK Netzdienste GmbH & Co. KG 51 9

1326 http://edoc3.bibliothek.uni-halle.de:8080/axis/vascoda.wsdl uni-halle.de 141.48.3.149 Germany 2368734101 AS680 service G-WiN 51.5 12

1327 http://edoc3.bibliothek.uni-halle.de:8080/axis/services/MyService?wsdl uni-halle.de 141.48.3.149 Germany 2368734101 AS680 service G-WiN 51.5 12

1328 http://edoc3.bibliothek.uni-halle.de:8080/axis/services/Version?wsdl uni-halle.de 141.48.3.149 Germany 2368734101 AS680 service G-WiN 51.5 12

1329 http://link.caseport.de/axis/services/urn:cp-accountmanager?wsdl caseport.de 83.243.58.142 Germany 1408449166 AS25504 Vautron AG 51 9

1330 http://link.caseport.de/axis/services/urn:cp-sessionmanager?wsdl caseport.de 83.243.58.142 Germany 1408449166 AS25504 Vautron AG 51 9

1331 http://link.caseport.de/axis/services/urn:cp-coursemanager?wsdl caseport.de 83.243.58.142 Germany 1408449166 AS25504 Vautron AG 51 9

1332 http://link.caseport.de/axis/services/Version?wsdl caseport.de 83.243.58.142 Germany 1408449166 AS25504 Vautron AG 51 9

1333 http://globplot.embl.de/webservice/globplot.wsdl embl.de 194.94.44.212 Germany 3260951764 AS680 service G-WiN 49.4167 8.7

1334 http://phospho.elm.eu.org/webservice/phosphoELMdb.wsdl embl.de 194.94.44.212 Germany 3260951764 AS680 service G-WiN 49.4167 8.7

1335 http://econtent.oberpfaelzerwald.de/DesktopModules/eContent-Events/AjaxEventCoreService.asmx?WSDL oberpfaelzerwald.de 193.58.245.86 Germany 3241866582 AS25394 AS for MK Netzdienste GmbH & Co. KG 48.9167 12.6833

1336 http://econtent.ostbayern-tourismus.de/DesktopModules/eContent-Events/AjaxEventCoreService.asmx?WSDL ostbayern-tourismus.de 193.58.245.68 Germany 3241866564 AS25394 AS for MK Netzdienste GmbH & Co. KG 48.9167 12.6833

1337 http://cms.cha.ostby.de/DesktopModules/eContent-Events/AjaxEventCoreService.asmx?WSDL ostby.de 82.100.207.234 Germany 1382338538 AS25394 AS for MK Netzdienste GmbH & Co. KG 51 9

1338 http://demo.touricoholidays.com/ws/AmendmentServices.asmx?WSDL touricoholidays.com 204.136.10.65 United States 3431467585 null 38 -97

1339 http://validator2.addressdoctor.com/addFastCompletion/FastCompletion.asmx?wsdl addressdoctor.com 64.27.57.29 Germany 1075525917 AS30475 WeHostWebSites.com 39.7525 -104.9995

1340 http://www.addressdoctor.com/Lookup/DQC.asmx?WSDL addressdoctor.com 64.27.57.24 Germany 1075525912 AS30475 WeHostWebSites.com 39.7525 -104.9995

1341 http://validator2.addressdoctor.com/addBatch/Batch.asmx?WSDL addressdoctor.com 64.27.57.29 Germany 1075525917 AS30475 WeHostWebSites.com 39.7525 -104.9995

1342 http://validator2.addressdoctor.com/addinteractive/interactive.asmx?WSDL addressdoctor.com 64.27.57.24 Germany 1075525912 AS30475 WeHostWebSites.com 39.7525 -104.9995

1343 http://www.agentcopp.de/AgentCoPP/services/Version?wsdl agentcopp.de 85.25.120.47 Germany 1427732527 AS8972 PlusServer AG, Germany 51 9

1344 http://www.agentcopp.de/AgentCoPP/services/Test1?wsdl agentcopp.de 85.25.120.47 Germany 1427732527 AS8972 PlusServer AG, Germany 51 9

1345 http://services.dschini.org/Bookstore.php?WSDL dschini.org 81.169.165.29 Germany 1370072349 AS6724 STRATO STRATO AG 52.5167 13.4

1346 http://dev.dschini.org/tests/Services_Webservice/test_01.php?WSDL dschini.org 81.169.165.29 Germany 1370072349 AS6724 STRATO STRATO AG 52.5167 13.4

1347 http://services.dschini.org/bot.php?WSDL dschini.org 81.169.165.29 Germany 1370072349 AS6724 STRATO STRATO AG 52.5167 13.4

1348 http://soap.fmui.de/statistics.wsdl fmui.de 87.230.106.24 Germany 1474718232 AS20773 AS of Hosteurope Germany / Cologne 51.65 6.1833

1349 http://soap.fmui.de/chemistry.wsdl fmui.de 87.230.106.24 Germany 1474718232 AS20773 AS of Hosteurope Germany / Cologne 51.65 6.1833

1350 http://soap.fmui.de/christmas.wsdl fmui.de 87.230.106.24 Germany 1474718232 AS20773 AS of Hosteurope Germany / Cologne 51.65 6.1833

1351 http://www.garantie-service-gmbh.de/lb_script/ge_srv/wsdl/IAppServer garantie-service-gmbh.de null Germany 0 null null null

1352 http://services.getpos.de/ip2loc.asmx?wsdl getpos.de 93.104.209.164 Germany 1567150500 AS8767 M-net AS 51 9

1353 http://services.getpos.de/radsearch.asmx?WSDL getpos.de 93.104.209.164 Germany 1567150500 AS8767 M-net AS 51 9

1354 http://services.getpos.de/ziplocation.asmx?WSDL getpos.de 93.104.209.164 Germany 1567150500 AS8767 M-net AS 51 9

1355 http://blog.getpos.de/api/BlogImporter.asmx?WSDL getpos.de 93.104.209.164 Germany 1567150500 AS8767 M-net AS 51 9

1356 http://www.hunderttausend.de/shared/webservice/cart.asmx?WSDL hunderttausend.de 194.25.167.153 Germany 3256461209 AS3320 Deutsche Telekom AG 49.75 6.6333

1357 http://api.iclear.de/XMLBuyTool/xmlbuytool.php?wsdl iclear.de 78.35.17.113 Germany 1310921073 AS8422 NETCOLOGNE NETCOLOGNE AS 51 9

1358 http://www.iclear.de/ICOrderServices.wsdl iclear.de 78.35.17.113 Germany 1310921073 AS8422 NETCOLOGNE NETCOLOGNE AS 51 9

1359 http://www.iclear.de/ICUserServices.wsdl iclear.de 78.35.17.113 Germany 1310921073 AS8422 NETCOLOGNE NETCOLOGNE AS 51 9

1360 http://www.luego.de:8080/rabs/services/Version?wsdl luego.de 176.28.53.80 Germany 2954638672 null 51.65 6.1833

1361 http://mietwagenmarkt.de/webServices/WsCustomerSaved.asmx?WSDL mietwagenmarkt.de 213.128.143.40 Germany 3581972264 AS12731 IPHH Internet Port Hamburg GmbH 53.55 10

1362 http://mietwagenmarkt.de/webServices/RegionService.asmx?WSDL mietwagenmarkt.de 213.128.143.40 Germany 3581972264 AS12731 IPHH Internet Port Hamburg GmbH 53.55 10

1363 http://www.rewisit.de:8080/axis/RewisitAdd.jws?wsdl rewisit.de 85.214.115.28 Germany 1440117532 AS6724 STRATO STRATO AG 52.5167 13.4

1364 http://www.rewisit.de:8080/axis/services/Version?wsdl rewisit.de 85.214.115.28 Germany 1440117532 AS6724 STRATO STRATO AG 52.5167 13.4

1365 http://www.testor.de/cgi-bin/msn testor.de 62.27.5.108 Germany 1041958252 AS12312 ecotel communication ag 51 9

1366 http://a3.testor.de/cgi-bin/bork/ testor.de 62.27.5.108 Germany 1041958252 AS12312 ecotel communication ag 51 9

1367 http://a3.testor.de/cgi-bin/eliza/ testor.de 62.27.5.108 Germany 1041958252 AS12312 ecotel communication ag 51 9

1368 http://www.testor.de/cgi-bin/serverType testor.de 62.27.5.108 Germany 1041958252 AS12312 ecotel communication ag 51 9

1369 http://projekt.wifo.uni-mannheim.de/elmar/api/SOAPMonitorService?wsdl uni-mannheim.de null Germany 0 null null null

1370 http://projekt.wifo.uni-mannheim.de/elmar/api/Version?wsdl uni-mannheim.de null Germany 0 null null null

1371 http://www.js.vertriebsunion.de/VuJournalService/Authentifizierung.asmx?WSDL vertriebsunion.de 85.214.130.78 Germany 1440121422 AS6724 STRATO STRATO AG 52.5167 13.4

1372 http://www.js.vertriebsunion.de/VuJournalService/GP/GP.asmx?wsdl vertriebsunion.de 85.214.130.78 Germany 1440121422 AS6724 STRATO STRATO AG 52.5167 13.4

1373 http://www.js.vertriebsunion.de/VuJournalService/Fibu/Fibu.asmx?wsdl vertriebsunion.de 85.214.130.78 Germany 1440121422 AS6724 STRATO STRATO AG 52.5167 13.4

1374 http://www.js.vertriebsunion.de/VuJournalService/Abo/Abo.asmx?wsdl vertriebsunion.de 85.214.130.78 Germany 1440121422 AS6724 STRATO STRATO AG 52.5167 13.4

1375 http://shark.bertelsmann.de/sharkws/services/wspaymentvalidation?wsdl bertelsmann.de 213.83.55.200 Germany 3579000776 AS12306 Plus.Line AG 51 9

1376 http://shark.bertelsmann.de/sharkws/services/wsfacade?wsdl bertelsmann.de 213.83.55.200 Germany 3579000776 AS12306 Plus.Line AG 51 9

1377 http://www.carhiremarket.com/webServices/WsCustomerSaved.asmx?WSDL carhiremarket.com 213.128.143.43 Germany 3581972267 AS12731 IPHH Internet Port Hamburg GmbH 53.55 10

1378 http://www.carhiremarket.com/webServices/RegionService.asmx?WSDL carhiremarket.com 213.128.143.43 Germany 3581972267 AS12731 IPHH Internet Port Hamburg GmbH 53.55 10

1379 http://vops1.hq.eso.org:8080/vospace/services/Version?wsdl eso.org 134.171.75.1 Germany 2259372801 AS680 service G-WiN 48.25 11.65

1380 http://www.findpeoplefree.co.uk/findpeoplefree.asmx?wsdl findpeoplefree.co.uk 212.227.102.68 Germany 3571672644 AS8560 1&1 Internet AG 51 9

1381 http://l8ms.co.uk/services/files.asmx?WSDL l8ms.co.uk 87.106.246.86 Germany 1466627670 AS8560 1&1 Internet AG 51 9

1382 http://meinsport.de/Services/LogService.asmx?WSDL meinsport.de 85.14.218.228 Germany 1427036900 AS13301 Autonomous System of unitedcolo.de 51 9

1383 http://meinsport.de/Services/SearchService.asmx?WSDL meinsport.de 85.14.218.228 Germany 1427036900 AS13301 Autonomous System of unitedcolo.de 51 9

1384 http://meinsport.de/Services/PostData.asmx?WSDL meinsport.de 85.14.218.228 Germany 1427036900 AS13301 Autonomous System of unitedcolo.de 51 9

1385 http://ws.openmusicsource.net/1.4/Platform/ArtistData?WSDL openmusicsource.net 80.190.99.2 Germany 1354654466 AS15598 IP Exchange GmbH 51 9

1386 http://ws.openmusicsource.net/1.4/Platform/ToolsData?WSDL openmusicsource.net 80.190.99.2 Germany 1354654466 AS15598 IP Exchange GmbH 51 9

1387 http://ws.openmusicsource.net/1.4/Platform/UserData?WSDL openmusicsource.net 80.190.99.2 Germany 1354654466 AS15598 IP Exchange GmbH 51 9

1388 http://app2.richshop.de/RPCatalogServices.wsdl richshop.de 78.35.17.84 Germany 1310921044 AS8422 NETCOLOGNE NETCOLOGNE AS 51 9

1389 http://app2.richshop.de/RPOrderServices.wsdl richshop.de 78.35.17.84 Germany 1310921044 AS8422 NETCOLOGNE NETCOLOGNE AS 51 9

1390 http://soap.smscreator.de/send.asmx?WSDL smscreator.de 195.243.107.145 Germany 3287509905 AS3320 Deutsche Telekom AG 50.1167 8.6833

1391 http://soap.smscreator.de/vcard.asmx?WSDL smscreator.de 195.243.107.145 Germany 3287509905 AS3320 Deutsche Telekom AG 50.1167 8.6833

1392 http://mhp-kdb.s3.uni-essen.de/jboss-net/services/TestCenterAuthentication?wsdl uni-essen.de null Germany 0 null null null

1393 http://sabio.bioquant.uni-heidelberg.de/sabiork?wsdl uni-heidelberg.de null Germany 0 null null null

1394 http://sabio.bioquant.uni-heidelberg.de/sabiows/sabiork.jws?wsdl uni-heidelberg.de null Germany 0 null null null

1395 http://asp.uniserv-online.de/axis/services/PhoneBrowser?wsdl uniserv-online.de null Germany 0 null null null

1396 http://asp.uniserv-online.de/axis/services/InternationalPostalValidation?wsdl uniserv-online.de null Germany 0 null null null

1397 http://asp.uniserv-online.de/axis/services/Geocoding?wsdl uniserv-online.de null Germany 0 null null null

1398 http://ukwplan.klinik.uni-wuerzburg.de/bais/MapServices.asmx?WSDL uni-wuerzburg.de 132.187.1.114 Germany 2226848114 AS680 service G-WiN 49.7878 9.9361

1399 http://pegelonline.wsv.de/webservices/version2_0/2006/07/26/PegelonlineWebservice?WSDL wsv.de null Germany 0 null null null

1400 http://www.pegelonline.wsv.de/webservices/PegelonlineWebservice?WSDL wsv.de null Germany 0 null null null

1401 http://146.107.217.178/web/services/ALOGPS?wsdl 146.107.217.178 146.107.217.178 Germany 2456541618 AS680 service G-WiN 48.15 11.5833

1402 http://146.107.217.178/web/services/Version?wsdl 146.107.217.178 146.107.217.178 Germany 2456541618 AS680 service G-WiN 48.15 11.5833

1403 http://service.aconti.net/externalCAP.asmx?wsdl aconti.net 89.31.3.82 Germany 1495204690 AS33808 I.T.E.N.O.S. GmbH 51.2167 6.7667

1404 http://www.advantech.be/webservice/AdvantechWebServiceLocal.asmx?WSDL advantech.be 87.248.203.253 United Kingdom 1475922941 AS22822 Limelight Networks, Inc. 42.8333 12.8333

1405 http://www.advantech.be/webservice/AdvantechWebService.asmx?WSDL advantech.be 87.248.203.253 United Kingdom 1475922941 AS22822 Limelight Networks, Inc. 42.8333 12.8333

1406 http://www.advantech.de/webservice/AdvantechWebService.asmx?WSDL advantech.de 87.248.203.253 United Kingdom 1475922941 AS22822 Limelight Networks, Inc. 42.8333 12.8333

1407 http://www.advantech.de/webservice/AdvantechWebServiceLocal.asmx?WSDL advantech.de 87.248.203.253 United Kingdom 1475922941 AS22822 Limelight Networks, Inc. 42.8333 12.8333

1408 http://www.advantech.fr/webservice/AdvantechWebService.asmx?WSDL advantech.fr 87.248.203.253 United Kingdom 1475922941 AS22822 Limelight Networks, Inc. 42.8333 12.8333

1409 http://www.advantech.fr/webservice/AdvantechWebServiceLocal.asmx?WSDL advantech.fr 87.248.203.253 United Kingdom 1475922941 AS22822 Limelight Networks, Inc. 42.8333 12.8333

1410 http://www.advantech.it/webservice/AdvantechWebServiceLocal.asmx?WSDL advantech.it 62.149.128.160 United Kingdom 1049985184 AS31034 Aruba S.p.A. - Network 42.8333 12.8333

1411 http://www.advantech.it/webservice/AdvantechWebService.asmx?WSDL advantech.it 62.149.128.157 United Kingdom 1049985181 AS31034 Aruba S.p.A. - Network 42.8333 12.8333

1412 http://www.advantech.nl/webservice/AdvantechWebService.asmx?WSDL advantech.nl 87.248.203.253 United Kingdom 1475922941 AS22822 Limelight Networks, Inc. 42.8333 12.8333

1413 http://www.advantech.nl/webservice/AdvantechWebServiceLocal.asmx?WSDL advantech.nl 87.248.203.253 United Kingdom 1475922941 AS22822 Limelight Networks, Inc. 42.8333 12.8333

1414 http://www.advantech-uk.com/webservice/AdvantechWebService.asmx?WSDL advantech-uk.com 87.248.203.253 United Kingdom 1475922941 AS22822 Limelight Networks, Inc. 42.8333 12.8333

1415 http://www.advantech-uk.com/webservice/AdvantechWebServiceLocal.asmx?WSDL advantech-uk.com 87.248.203.253 United Kingdom 1475922941 AS22822 Limelight Networks, Inc. 42.8333 12.8333

1416 http://www.biathlonresults.com/WebServices/TextServices.asmx?wsdl biathlonresults.com 54.228.244.71 Germany 920974407 null 53 -8

1417 http://code-developer.de/opensourceprojects/shoppingmap/AjaxWebservice.asmx?WSDL code-developer.de 188.64.60.112 Germany 3158326384 AS8741 AS - ecore Kommunikations AG 51 9

1418 http://www.dassel2000.de/service/GDwsTipps.asmx?WSDL dassel2000.de 85.197.113.10 Germany 1439002890 AS29471 WebJanssen ISP ltd & Co KG 51 9

1419 http://www.dassel2000.de/service/GDwsBundesliga.asmx?WSDL dassel2000.de 85.197.113.10 Germany 1439002890 AS29471 WebJanssen ISP ltd & Co KG 51 9

1420 http://mlb.dvod.de/dl/control.asmx?WSDL dvod.de 127.0.0.1 Germany 2130706433 null null null

1421 http://cds.dvod.de/cds/cds.asmx?wsdl dvod.de 127.0.0.1 Germany 2130706433 null null null

1422 http://gms.givit.de/webservice/dbconnector.cfc?wsdl givit.de 46.30.63.112 Germany 773734256 null 52.3833 8.6333

1423 http://gms.givit.de/webservice/rgswag/1.cfc?wsdl givit.de 46.30.63.112 Germany 773734256 null 52.3833 8.6333

1424 http://igodemo2.infsoft.org/MapServices.asmx?WSDL infsoft.org 85.214.157.215 Germany 1440128471 AS6724 STRATO STRATO AG 52.5167 13.4

1425 http://iq-gmbh.com/WebService/webservices.nsf/IQUpdate?WSDL iq-gmbh.com 94.16.11.73 Germany 1578109769 AS15598 IP Exchange GmbH 49.015 12.0956

1426 http://iq-gmbh.com/WebService/webservices.nsf/iqGenerateDownloadLink?WSDL iq-gmbh.com 94.16.11.73 Germany 1578109769 AS15598 IP Exchange GmbH 49.015 12.0956

1427 http://konzentrik.de/vod/lib/soap/asset.wsdl konzentrik.de 85.214.22.71 Germany 1440093767 AS6724 STRATO STRATO AG 52.5167 13.4

1428 http://webservices.newcomer.de/newcomerglobal/functions.asmx?WSDL newcomer.de 62.146.186.166 Germany 1049803430 AS15598 IP Exchange GmbH 51 9

1429 http://www.online-mitgliederverwaltung.de/bds/Kreisverbaende/Kreisverband.asmx?WSDL online-mitgliederverwaltung.de 213.174.57.204 Germany 3584965068 AS21499 internet24 GmbH 51 9

1430 http://canoe.siwidata.info/WebServices/TextServices.asmx?WSDL siwidata.info 87.119.211.204 Germany 1467470796 AS25074 INET-People Provider Services 51 9

1431 http://canoe.siwidata.info/WebServices/Basic.asmx?WSDL siwidata.info 87.119.211.204 Germany 1467470796 AS25074 INET-People Provider Services 51 9

1432 http://www.sw-consultant.de/webservices/zipcodes.asmx?WSDL sw-consultant.de 188.64.60.112 Germany 3158326384 AS8741 AS - ecore Kommunikations AG 51 9

1433 http://www.sw-consultant.de/webservices/AreaCodes.asmx?WSDL sw-consultant.de 188.64.60.112 Germany 3158326384 AS8741 AS - ecore Kommunikations AG 51 9

1434 http://www.teamletter.de/services/NewsletterService?wsdl teamletter.de 212.29.6.154 Germany 3558672026 AS8208 Teamware GmbH 51 9

1435 http://www.teamletter.de/services/NewsletterUserService?wsdl teamletter.de 212.29.6.154 Germany 3558672026 AS8208 Teamware GmbH 51 9

1436 http://www.trostonline.de/autobest/tolwebservice.dll/wsdl/ITOLServiceInterface trostonline.de 94.100.247.106 Germany 1583675242 AS34086 T-Systems Enterprise Services GmbH 48.8333 10.1

1437 http://www.inf.tu-dresden.de/service/?wsdl tu-dresden.de 141.30.2.2 Germany 2367554050 AS680 service G-WiN 51.05 13.75

1438 http://vcclab.org/web/services/Version?wsdl vcclab.org 146.107.217.178 Germany 2456541618 AS680 service G-WiN 48.15 11.5833

1439 http://vcclab.org/web/services/ALOGPS?wsdl vcclab.org 146.107.217.178 Germany 2456541618 AS680 service G-WiN 48.15 11.5833

1440 http://sabio.villa-bosch.de/sabiows/sabiork.jws?wsdl villa-bosch.de 212.126.215.84 Germany 3565082452 AS5409 toplink GmbH 49.4167 8.7

1441 http://sabio.villa-bosch.de/sabiork?wsdl villa-bosch.de 212.126.215.84 Germany 3565082452 AS5409 toplink GmbH 49.4167 8.7

1442 http://virtuallaboratory.org/web/services/ALOGPS?wsdl virtuallaboratory.org 146.107.217.178 Germany 2456541618 AS680 service G-WiN 48.15 11.5833

1443 http://virtuallaboratory.org/web/services/Version?wsdl virtuallaboratory.org 146.107.217.178 Germany 2456541618 AS680 service G-WiN 48.15 11.5833

1444 http://www.yachtbooker.de/CMSTemplates/cms_yb2/MapService.asmx?WSDL yachtbooker.de 87.106.144.23 Germany 1466601495 AS8560 1&1 Internet AG 51 9

1445 http://www.yachtbooker.de/YStatsService.asmx?WSDL yachtbooker.de 87.106.144.23 Germany 1466601495 AS8560 1&1 Internet AG 51 9

1446 http://www.yucata.de/Services/RankingFunctions.asmx?WSDL yucata.de 188.64.57.2 Germany 3158325506 AS8741 AS - ecore Kommunikations AG 51 9

1447 http://zeta-software.de/Translator/TranslationService.asmx?wsdl zeta-software.de 217.160.24.237 Germany 3651148013 AS8560 1&1 Internet AG 51 9

1448 http://141.47.75.234/~soap/hswetter.php?wsdl 141.47.75.234 141.47.75.234 Germany 2368687082 AS553 Landeshochschulnetz Baden-Wuerttemberg (BelWue) 48.8833 8.7

1449 http://195.37.165.98/webservices/PegelonlineWebservice?WSDL 195.37.165.98 195.37.165.98 Germany 3274024290 AS680 service G-WiN 51 9

1450 http://213.252.14.229/nLP/GPSConnector/trial/gpsconnector.asmx?WSDL 213.252.14.229 213.252.14.229 Germany 3590065893 AS12907 ip&more GmbH 48.15 11.5833

1451 http://87.106.63.42/kapix/kapix.asmx?wsdl 87.106.63.42 87.106.63.42 Germany 1466580778 AS8560 1&1 Internet AG 51 9

1452 http://alx-development.de/fileadmin/projects/babylon/Babylon/src/SOAP/index.php?wsdl alx-development.de 176.221.42.131 Germany 2967284355 null 51 9

1453 http://autostadt.de/ecard-war/services/ECardSOAP?wsdl autostadt.de 194.114.76.187 Germany 3262270651 AS13130 Autostadt Wolfsburg 52.4333 10.8

1454 http://secure.ceneo.pl/bartek/main/trunk/current/allegro-interop/repo/ceneo-api/ws/internal-ws-pics/Service1.asmx?WSDL ceneo.pl 178.21.159.11 Germany 2987761419 AS31621 QXL Poland sp. z o.o. 53.5076 18.6358

1455 http://www.centraline.com/wservice/authenticateUser.wsdl centraline.com 213.185.82.132 Germany 3585692292 AS29354 PCE-NET Rhein Ruhr,Luxemburgerstr. 305,D-50354 Huerth, 50.8667 6.8667

1456 http://www.corsairmemory.com/cfgrtr_keyword.asmx?WSDL corsairmemory.com 8.25.207.132 Germany 135909252 AS3356 Level 3 Communications 38 -97

1457 http://www.currencyserver.de/webservice/currencyserverwebservice.asmx?WSDL currencyserver.de 62.96.6.3 Germany 1046480387 AS8220 COLT Technology Services 51.5 -0.13

1458 http://dotnet-snippets.de/dns/Webservice.asmx?WSDL dotnet-snippets.de 176.28.49.94 Germany 2954637662 null 51.65 6.1833

1459 http://blog.easyciel.net/api/BlogImporter.asmx?WSDL easyciel.net null Germany 0 null null null

1460 http://www.events-planner.de/search/AutoComplete.asmx?WSDL events-planner.de null Germany 0 null null null

1461 http://film-shop-home.de/temp/nusoap/try2/server1.php?wsdl film-shop-home.de null Germany 0 null null null

1462 http://www.gammareifen.com/wsorders.asmx?wsdl gammareifen.com 85.197.112.2 Germany 1439002626 AS29471 WebJanssen ISP ltd & Co KG 51 9

1463 http://www.getreide-aktuell.de/Bonituren.asmx?WSDL getreide-aktuell.de 165.160.13.20 Germany 2778729748 AS19574 Corporation Service Company 39.7351 -75.6684

1464 http://www.glycosciences.de/services/KeywordService?wsdl glycosciences.de 134.176.43.25 Germany 2259692313 AS680 service G-WiN 50.5833 8.65

1465 http://guidgen.com/WS.asmx?WSDL guidgen.com 217.160.24.237 Germany 3651148013 AS8560 1&1 Internet AG 51 9

1466 http://www.g-vo.org/rosat/soap/RASSConeSearches?wsdl g-vo.org 129.206.112.252 Germany 2177790204 AS553 Landeshochschulnetz Baden-Wuerttemberg (BelWue) 49.4167 8.7

1467 http://www.hauser-wenz.de/playground/ws/eBayWatcherService.wsdl hauser-wenz.de 91.184.32.134 Germany 1538793606 AS34225 SpeedPartner GmbH IPv4 & IPv6 51 9

1468 http://webservices.ifap.de/ifapWebService.asmx?WSDL ifap.de 213.252.2.175 Germany 3590062767 AS12907 ip&more GmbH 51 9

1469 http://info-messenger.de/sms.asmx?wsdl info-messenger.de 195.243.107.140 Germany 3287509900 AS3320 Deutsche Telekom AG 50.1167 8.6833

1470 http://www.itatservice.de/itatservice/itatservice1.asmx?WSDL itatservice.de 195.234.228.210 Germany 3286951122 AS25260 QualityHosting AG 50.2 9.1833

1471 http://www.klammeraffe.org/wsdl/guestbook_server.php?wsdl klammeraffe.org 178.63.61.72 Germany 2990488904 AS24940 Hetzner Online AG RZ 51 9

1472 http://ivr.konfigserver.de/ideawise/testservice/service.asmx?WSDL konfigserver.de 83.141.3.208 Germany 1401750480 AS29551 Aixit GmbH 51 9

1473 http://www.location-guide-europe.com/search/AutoComplete.asmx?WSDL location-guide-europe.com 82.98.104.214 Germany 1382181078 AS12306 Plus.Line AG 51 9

1474 http://www.locationguideeurope.de/search/AutoComplete.asmx?WSDL locationguideeurope.de 82.98.104.214 Germany 1382181078 AS12306 Plus.Line AG 51 9

1475 http://www.mediestrategi.dk/services/sms_server.php?wsdl mediestrategi.dk 78.47.241.59 Germany 1311764795 AS24940 Hetzner Online AG RZ 51 9

1476 http://www.mice-guide-europe.com/search/AutoComplete.asmx?WSDL mice-guide-europe.com 82.98.104.214 Germany 1382181078 AS12306 Plus.Line AG 51 9

1477 http://msiggi.de/Blog/api/BlogImporter.asmx?WSDL msiggi.de 87.106.137.146 Germany 1466599826 AS8560 1&1 Internet AG 51 9

1478 http://tnorad.tn.ohost.de/rshare/rwpcws.wsdl ohost.de 213.202.225.245 Germany 3586843125 AS13301 Autonomous System of unitedcolo.de 51 9

1479 http://www.opaltrans.de/mec/html/ecourierIN.php5?wsdl opaltrans.de 62.116.143.11 Germany 1047826187 AS15456 InterNetX GmbH 51 9

1480 http://www.openligadb.de/Webservices/Sportsdata.asmx?wsdl openligadb.de 85.214.205.163 Germany 1440140707 AS6724 STRATO STRATO AG 52.5167 13.4

1481 http://www.pegel-mainz.de/WSDL/iLON100.WSDL pegel-mainz.de null Germany 0 null null null

1482 http://www.pegelstaende.de/webservices/version2_2/2007/04/10/PegelonlineWebservice?WSDL pegelstaende.de null Germany 0 null null null

1483 http://www.slkv.net/transfer/trans.asmx?WSDL slkv.net null Germany 0 null null null

1484 http://smsbug.com/api/webservice.asmx?wsdl smsbug.com 74.3.236.99 Germany 1241771107 AS26228 ServePath, LLC 37.7898 -122.3942

1485 http://soap.smsworkx.de/send.asmx?wsdl smsworkx.de 46.252.31.212 Germany 788275156 null 52.5167 13.4

1486 http://www.stiftunglesen.de/Search.asmx?WSDL stiftunglesen.de 82.165.146.70 Germany 1386582598 AS8560 1&1 Internet AG 51 9

1487 http://studihelp.de/Services/SearchService.asmx?WSDL studihelp.de 85.14.218.193 Germany 1427036865 AS13301 Autonomous System of unitedcolo.de 51 9

1488 http://studihelp.net/Services/SearchService.asmx?WSDL studihelp.net 85.14.218.193 Germany 1427036865 AS13301 Autonomous System of unitedcolo.de 51 9

1489 http://carhire.thomsonfly.com/webServices/RegionService.asmx?WSDL thomsonfly.com 95.174.80.102 Germany 1605259366 null 51 9

1490 http://www.toggo-cleverclub.de/WebServices/Tracking/wsTracking.asmx?WSDL toggo-cleverclub.de 85.25.247.101 Germany 1427765093 AS8972 PlusServer AG, Germany 51 9

1491 http://webclu.bio.wzw.tum.de:18080/complex/services/profcom?wsdl tum.de null Germany 0 null null null

1492 http://services.unitedplanet.de/blz/BlzService.asmx?WSDL unitedplanet.de 212.185.176.183 Germany 3568939191 AS3320 Deutsche Telekom AG 48 7.85

1493 http://wsvc.vianova-net.de/public/quartier.asmx?WSDL vianova-net.de 81.20.84.236 Germany 1360286956 AS25260 QualityHosting AG 51 9

1494 http://www.vistalog.de/logsvc/logsvc1.asmx?WSDL vistalog.de 80.252.104.128 Germany 1358719104 AS8893 Artfiles New Media GmbH 51 9

1495 http://ald.yellowmap.de/MTE/MessagingService.asmx?WSDL yellowmap.de 213.144.5.240 Germany 3582985712 AS12843 TelemaxX Telekommunikation GmbH Autonomous System 49.0078 8.4198

1496 http://api.stats.yellowpages.pl/stats.php?wsdl yellowpages.pl 46.4.84.176 Germany 772035760 AS24940 Hetzner Online AG RZ 51 9

1497 http://yoosing.com/services/portletService.php?wsdl yoosing.com 87.106.73.83 Germany 1466583379 AS8560 1&1 Internet AG 51 9

1498 http://museum.zib.de/museumsvokabular/webservice/museumvok-ws0.4.wsdl zib.de null Germany 0 null null null

1499 http://www.kakos.com.gr/webservices/WebServiceSolve2ndGrade.asmx?WSDL kakos.com.gr 62.103.159.150 Greece 1046978454 AS6799 OTEnet S.A. Multiprotocol Backbone & ISP 39 22

1500 http://www.kakos.com.gr/webservices/WebService2.asmx?WSDL kakos.com.gr 62.103.159.150 Greece 1046978454 AS6799 OTEnet S.A. Multiprotocol Backbone & ISP 39 22

1501 http://www.argos.net.gr/Argoswebservice/service.asmx?WSDL argos.net.gr null Greece 0 null null null

1502 http://ebook.service.atcom.gr/pdftools.asmx?WSDL atcom.gr 62.1.42.51 Greece 1040263731 AS1241 FORTHnet 39 22

1503 http://eserver.aviareps.gr/daedalus/FlightsWS.asmx?wsdl aviareps.gr 31.207.4.114 Greece 533660786 null 49.75 15.5

1504 http://remath.cti.gr/MathDiLS/MathDiLS.asmx?WSDL cti.gr 150.140.1.11 Greece 2525757707 AS6744 Research Academic Computer Technology Institute (CTI) 38.2444 21.7344

1505 http://ws.ngi.gr/NGIMapServer/wsdl/all ngi.gr 193.92.77.188 Greece 3244051900 AS1241 FORTHnet 37.9833 23.7333

1506 http://banguat.gob.gt/variables/ws/TipoCambio.asmx?WSDL banguat.gob.gt 181.114.17.37 Guatemala 3044151589 null 14.6211 -90.5269

1507 http://banguat.gob.gt/variables/ws/BDEF.asmx?WSDL banguat.gob.gt 200.12.46.201 Guatemala 3356241609 AS6487 CyberNet de Guatemala S.A. 14.6211 -90.5269

1508 http://www2.comp.polyu.edu.hk:8090/cssiunam/time-deploy/time?WSDL polyu.edu.hk 158.132.82.94 Hong kong 2659471966 AS4616 Pristine Communications Limited Information Technology Services 22.25 114.1667

1509 http://etrade.dimerco.com.hk/peter-2007oct04-001/service.asmx?WSDL dimerco.com.hk null Hong kong 0 null null null

1510 http://www.meteorsis.com/missms/missms.asmx?wsdl meteorsis.com 61.93.232.34 Hong kong 1029564450 AS9269 City Telecom (H.K.) Ltd. 22.3167 114.2167

1511 http://hkbeta.morningstar.com/webservices/venhouse/venhouse_datafeed.asmx?WSDL morningstar.com 216.228.225.51 Hong kong 3638878515 AS25721 13494 - Morningstar, Inc. 41.8826 -87.6292

1512 http://www.gdf.hu/_vti_bin/BusinessDataCatalog.asmx?wsdl gdf.hu 81.0.70.250 Hungary 1358972666 AS12301 Invitel Tavkozlesi Zrt. 47 20

1513 http://www.gdf.hu/_vti_bin/People.asmx?wsdl gdf.hu 81.0.70.250 Hungary 1358972666 AS12301 Invitel Tavkozlesi Zrt. 47 20

1514 http://www.gdf.hu/_vti_bin/Authentication.asmx?wsdl gdf.hu 81.0.70.250 Hungary 1358972666 AS12301 Invitel Tavkozlesi Zrt. 47 20

1515 http://portal.bme.hu/_vti_bin/Lists.asmx?wsdl bme.hu 152.66.115.203 Hungary 2554491851 AS2547 Budapest University of Technology and Economics 47.5 19.0833

1516 http://interpressfact.net/webservices/getjoke.asmx?WSDL interpressfact.net 193.142.153.110 Hungary 3247348078 AS41075 ATW Internet Kft. 47 20

1517 http://interpressfact.net/webservices/getAds.asmx?WSDL interpressfact.net 193.142.153.110 Hungary 3247348078 AS41075 ATW Internet Kft. 47 20

1518 http://interpressfact.net/webservices/promotionservice.asmx?WSDL interpressfact.net 193.142.153.110 Hungary 3247348078 AS41075 ATW Internet Kft. 47 20

1519 http://tv.animare.hu/webservice/tvguide.asmx?WSDL animare.hu 212.40.65.11 Hungary 3559407883 AS21229 TVNETWORK 47 20

1520 http://tv.animare.hu/webservice/command.asmx?WSDL animare.hu 212.40.65.11 Hungary 3559407883 AS21229 TVNETWORK 47 20

1521 http://automanager.hu/Ws/Listing.asmx?WSDL automanager.hu 195.70.57.200 Hungary 3276159432 AS8358 InterWare Autonomus System 47 20

1522 http://automenedzser.hu/Ws/Listing.asmx?WSDL automenedzser.hu 195.70.57.200 Hungary 3276159432 AS8358 InterWare Autonomus System 47 20

1523 http://newkibteszt.bet.hu/NewsManager?wsdl bet.hu 80.249.172.72 Hungary 1358539848 AS15555 Magyar Telekom plc. 47 20

1524 http://boomerangservices.ro/WorldAirports.asmx?WSDL boomerangservices.ro 79.172.211.128 Hungary 1336726400 AS29278 Deninet KFT 47 20

1525 http://www.ittvoltam.hu/webservice/command.asmx?WSDL ittvoltam.hu 212.40.65.11 Hungary 3559407883 AS21229 TVNETWORK 47 20

1526 http://www.mnb.hu/arfolyamok.asmx?wsdl mnb.hu 195.56.171.138 Hungary 3275271050 AS3340 DataNet Telecommunication Ltd. 47 20

1527 http://www.morphologic.hu/OfficeResearch/MoBiDic/Registration.asmx?WSDL morphologic.hu 195.228.254.20 Hungary 3286564372 AS5483 Hungarian Telecom ; Magyar Telekom 47 20

1528 http://jogviszony.oep.hu/ojote/TAJInfoSoapHttpPort?WSDL oep.hu 84.206.45.70 Hungary 1422798150 AS31581 Kopint-Datorg Co. 47.5 19.0833

1529 http://broadcast.dev.nepal.is/TestWebServicesFTP/EinarTest.asmx?WSDL nepal.is 194.105.250.3 Iceland 3261725187 AS6677 Siminn hf 64.5468 -21.9143

1530 http://broadcast.dev.nepal.is/InstallationService.asmx?WSDL nepal.is 194.105.250.3 Iceland 3261725187 AS6677 Siminn hf 64.5468 -21.9143

1531 http://throun.kogun.is/starfandi3/HeraWebService/Service.asmx?WSDL kogun.is 82.221.28.195 Iceland 1390222531 AS30818 SKYRR hf Armula 2 - Skyrr ISP Network 65 -18

1532 http://forlagid.is/WebServices/AutoCompleteService.asmx?WSDL forlagid.is 79.171.98.173 Iceland 1336631981 AS43892 Basis ehf. 65 -18

1533 http://jpv.is/WebServices/AutoCompleteService.asmx?WSDL jpv.is 79.171.98.173 Iceland 1336631981 AS43892 Basis ehf. 65 -18

1534 http://voruhus.landlaeknir.is/vhus_heilsufarsgagna/VHGService.asmx?WSDL landlaeknir.is 80.248.29.165 Iceland 1358437797 AS39418 Nyherji hf 65 -18

1535 http://203.199.178.89:9090/axis/services/Ffiledisplay?wsdl 203.199.178.89 203.199.178.89 India 3418862169 AS4755 TATA Communications formerly VSNL is Leading ISP 17.3753 78.4744

1536 http://203.199.178.89:9090/axis/services/Review?wsdl 203.199.178.89 203.199.178.89 India 3418862169 AS4755 TATA Communications formerly VSNL is Leading ISP 17.3753 78.4744

1537 http://203.199.178.89:9090/axis/services/approveorder?wsdl 203.199.178.89 203.199.178.89 India 3418862169 AS4755 TATA Communications formerly VSNL is Leading ISP 17.3753 78.4744

1538 http://203.199.178.89:9090/axis/services/captionurl?wsdl 203.199.178.89 203.199.178.89 India 3418862169 AS4755 TATA Communications formerly VSNL is Leading ISP 17.3753 78.4744

1539 http://203.199.178.89:9090/axis/services/iwfs?wsdl 203.199.178.89 203.199.178.89 India 3418862169 AS4755 TATA Communications formerly VSNL is Leading ISP 17.3753 78.4744

1540 http://203.199.178.89:9090/axis/services/ReceiveNoteService?wsdl 203.199.178.89 203.199.178.89 India 3418862169 AS4755 TATA Communications formerly VSNL is Leading ISP 17.3753 78.4744

1541 http://203.199.178.89:9090/axis/services/Sendfilesservice?wsdl 203.199.178.89 203.199.178.89 India 3418862169 AS4755 TATA Communications formerly VSNL is Leading ISP 17.3753 78.4744

1542 http://203.199.178.89:9090/axis/services/ReceiveNotingService?wsdl 203.199.178.89 203.199.178.89 India 3418862169 AS4755 TATA Communications formerly VSNL is Leading ISP 17.3753 78.4744

1543 http://203.199.178.89:9090/axis/services/ReceiveService?wsdl 203.199.178.89 203.199.178.89 India 3418862169 AS4755 TATA Communications formerly VSNL is Leading ISP 17.3753 78.4744

1544 http://203.199.178.89:9090/axis/services/Allnotings?wsdl 203.199.178.89 203.199.178.89 India 3418862169 AS4755 TATA Communications formerly VSNL is Leading ISP 17.3753 78.4744

1545 http://203.199.178.89:9090/axis/services/recnoting?wsdl 203.199.178.89 203.199.178.89 India 3418862169 AS4755 TATA Communications formerly VSNL is Leading ISP 17.3753 78.4744

1546 http://203.199.178.89:9090/axis/services/linkedfiles?wsdl 203.199.178.89 203.199.178.89 India 3418862169 AS4755 TATA Communications formerly VSNL is Leading ISP 17.3753 78.4744

1547 http://www.gorecroot.com/webservices/RRWebService.asmx?WSDL gorecroot.com 209.235.193.202 India 3521888714 AS20284 INETu, Inc. 40.6452 -75.4374

1548 http://www.gorecroot.com/callback/webservices/SearchRes.asmx?WSDL gorecroot.com 209.235.193.202 India 3521888714 AS20284 INETu, Inc. 40.6452 -75.4374

1549 http://www.gorecroot.com/callback/webservices/JobberTalk.asmx?WSDL gorecroot.com 209.235.193.202 India 3521888714 AS20284 INETu, Inc. 40.6452 -75.4374

1550 http://www.gorecroot.com/callback/webservices/USResumeSearch.asmx?WSDL gorecroot.com 209.235.193.202 India 3521888714 AS20284 INETu, Inc. 40.6452 -75.4374

1551 http://www.gorecroot.com/callback/webservices/Subscription.asmx?WSDL gorecroot.com 209.235.193.202 India 3521888714 AS20284 INETu, Inc. 40.6452 -75.4374

1552 http://www.gorecroot.com/callback/webservices/tellafriend.asmx?WSDL gorecroot.com 209.235.193.202 India 3521888714 AS20284 INETu, Inc. 40.6452 -75.4374

1553 http://www.gorecroot.com/callback/webservices/PIP.asmx?WSDL gorecroot.com 209.235.193.202 India 3521888714 AS20284 INETu, Inc. 40.6452 -75.4374

1554 http://www.gorecroot.com/callback/webservices/LoginLogout.asmx?WSDL gorecroot.com 209.235.193.202 India 3521888714 AS20284 INETu, Inc. 40.6452 -75.4374

1555 http://carwale.com/webservices/carvalues.asmx?WSDL carwale.com 124.153.77.15 India 2090421519 AS17439 Netmagic Datacenter Mumbai 20 77

1556 http://services.myiris.com/icerswebtest/SAST/sast.asmx?wsdl myiris.com 202.154.161.210 India 3399131602 AS4755 TATA Communications formerly VSNL is Leading ISP 20 77

1557 http://58.68.25.35/Tajelite/TravelPortal/TajWebV2.asmx?WSDL 58.68.25.35 58.68.25.35 India 977541411 AS10201 Dishnet Wireless Limited. Broadband Wireless 13.0833 80.2833

1558 http://webconnect.akbartravelsonline.com/service.asmx?WSDL akbartravelsonline.com 180.179.137.204 India 3031665100 null 20 77

1559 http://www.bangaloreone.gov.in/bOneWS/MgrService.asmx?WSDL bangaloreone.gov.in 210.212.203.180 India 3537161140 AS9829 National Internet Backbone 12.9833 77.5833

1560 http://www.elbee.in/eelwsdl/service1.asmx?wsdl elbee.in 82.98.86.162 India 1382176418 AS12306 Plus.Line AG 51 9

1561 http://jhaverisec.net/fileuploadws.asmx?WSDL jhaverisec.net null India 0 null null null

1562 http://offerings.nic.in/finalcomponent/stateinfosrv.asmx?WSDL offerings.nic.in 164.100.52.120 India 2758030456 AS4758 National Informatics Centre 20 77

1563 http://www.popularcarworld.com/UploadDownload.asmx?WSDL popularcarworld.com 180.179.132.193 India 3031663809 null 20 77

1564 http://smscountry.com/service.asmx?WSDL smscountry.com 182.18.132.7 India 3054666759 AS18229 Pioneer Elabs Ltd. 17.45 78.5

1565 http://www.vrllogistics.in/webs/ws/vrlbookingservice.asmx?WSDL vrllogistics.in 210.212.198.5 India 3537159685 AS9829 National Internet Backbone 14.5167 75.8

1566 http://www.egovernance.ie/Services/MyBoardService.asmx?WSDL egovernance.ie 137.191.244.221 Ireland 2311058653 AS15806 Irish Government 53.3331 -6.2489

1567 http://www.egovernance.ie/Services/CountyService.asmx?WSDL egovernance.ie 137.191.244.221 Ireland 2311058653 AS15806 Irish Government 53.3331 -6.2489

1568 http://www.egovernance.ie/Services/PeopleService.asmx?WSDL egovernance.ie 137.191.244.221 Ireland 2311058653 AS15806 Irish Government 53.3331 -6.2489

1569 http://staging.myrma.net/webservices/ClientService.asmx?WSDL myrma.net 91.103.1.23 Ireland 1533477143 AS47720 Chip Electronic Services Limited 51.8986 -8.4958

1570 http://staging.myrma.net/webservices/ServiceCentreService.asmx?WSDL myrma.net 91.103.1.23 Ireland 1533477143 AS47720 Chip Electronic Services Limited 51.8986 -8.4958

1571 http://staging.myrma.net/webservices/WarrantorService.asmx?WSDL myrma.net 91.103.1.23 Ireland 1533477143 AS47720 Chip Electronic Services Limited 51.8986 -8.4958

1572 http://193.95.154.227/AlertsWebService/AlertsWS.asmx?WSDL 193.95.154.227 193.95.154.227 Ireland 3244268259 AS2110 BT Ireland Backbone 53 -8

1573 http://193.95.154.227/FeedsWebService/FeedsWS.asmx?WSDL 193.95.154.227 193.95.154.227 Ireland 3244268259 AS2110 BT Ireland Backbone 53 -8

1574 http://www.digitalriver.com/v2.0-doc/wsdl/DigitalRiverDT.wsdl digitalriver.com 209.87.182.60 Ireland 3512186428 AS8182 Digital River, Inc. 44.98 -93.2638

1575 http://www.limerickcity.ie/WebServices/MediaNotices/MediaNotices.asmx?wsdl limerickcity.ie 137.191.226.163 Ireland 2311053987 AS15806 Irish Government 52.6647 -8.6231

1576 http://www.limerickcity.ie/webservices/planningapps/planningapps.asmx?wsdl limerickcity.ie 137.191.226.163 Ireland 2311053987 AS15806 Irish Government 52.6647 -8.6231

1577 http://www.limerickcorp.ie/WebServices/MediaNotices/MediaNotices.asmx?WSDL limerickcorp.ie 137.191.226.163 Ireland 2311053987 AS15806 Irish Government 52.6647 -8.6231

1578 http://www.limerickcorp.ie/webservices/planningapps/planningapps.asmx?WSDL limerickcorp.ie 137.191.226.163 Ireland 2311053987 AS15806 Irish Government 52.6647 -8.6231

1579 http://www.airside-nissan.com/web_services/ajaxUtilities.asmx?WSDL airside-nissan.com 89.101.142.175 Ireland 1499827887 AS6830 UPC Broadband 53.3331 -6.2489

1580 http://soap.bind.ca/wsdl/bind.wsdl bind.ca 84.18.180.105 Ireland 1410511977 AS34004 Thomson Corporation 53 -8

1581 http://cbg.ie/newcars/ajaxSearchService.asmx?WSDL cbg.ie 212.126.36.161 Ireland 3565036705 AS31122 Digiweb Ltd. 52.6333 1.3

1582 http://www.donegalflytrade.com/availability.php?wsdl donegalflytrade.com 195.26.90.35 Ireland 3273284131 AS31727 Node4 Ltd, UK 52.9333 -1.2333

1583 http://www.greatvessel.com/PortalService.asmx?WSDL greatvessel.com 82.195.154.72 Ireland 1388550728 AS29650 Sungard Availability Services (Ireland) Ltd. 53 -8

1584 http://www.nissan-dundalk.com/web_services/ajaxUtilities.asmx?WSDL nissan-dundalk.com 89.101.142.175 Ireland 1499827887 AS6830 UPC Broadband 53.3331 -6.2489

1585 http://www.recipewitch.com/RecipeService.asmx?WSDL recipewitch.com 63.156.206.202 Ireland 1067241162 AS209 Qwest Communications Company, LLC 38 -97

1586 http://www.roomex.com/services/LocationService.asmx?WSDL roomex.com 84.51.238.82 Ireland 1412689490 AS29650 Sungard Availability Services (Ireland) Ltd. 53 -8

1587 http://tehpain.com/SudokuSolverWS.asmx?WSDL tehpain.com null Ireland 0 null null null

1588 http://www.sapco.com/_vti_bin/BusinessDataCatalog.asmx?wsdl sapco.com 193.105.2.31 Islamic Republic of Iran 3244884511 AS42915 Engeneering Designing and Supply the parts of Iran Khodro PJSC 32 53

1589 http://www.sapco.com/_vti_bin/Authentication.asmx?wsdl sapco.com 193.105.2.31 Islamic Republic of Iran 3244884511 AS42915 Engeneering Designing and Supply the parts of Iran Khodro PJSC 32 53

1590 http://www.sapco.com/_vti_bin/People.asmx?wsdl sapco.com 193.105.2.31 Islamic Republic of Iran 3244884511 AS42915 Engeneering Designing and Supply the parts of Iran Khodro PJSC 32 53

1591 http://webservice.magfa.com/services/Version?wsdl magfa.com 217.11.25.234 United Kingdom 3641383402 AS25184 AFRANET Co. Tehran, Iran 35.7261 51.3304

1592 http://80.253.147.99/services/Version?wsdl 80.253.147.99 80.253.147.99 United Kingdom 1358795619 AS24631 Azadnet Autonomous System 32 53

1593 http://81.91.129.80/DialupWS/LotteryService.asmx?WSDL 81.91.129.80 81.91.129.80 Islamic Republic of Iran 1364951376 AS25124 DATAK Telecom Autonomous System 32 53

1594 http://81.91.129.80/DialupWS/SecurityService.asmx?WSDL 81.91.129.80 81.91.129.80 Islamic Republic of Iran 1364951376 AS25124 DATAK Telecom Autonomous System 32 53

1595 http://profiles.roshd.ir/security.asmx?WSDL roshd.ir 217.218.177.18 Islamic Republic of Iran 3654988050 AS12880 DCI Autonomous System 32 53

1596 http://www.taavonportal.ir/OstanShahrestan.asmx?WSDL taavonportal.ir null Islamic Republic of Iran 0 null null null

1597 http://slk.sulam.org.il/_vti_bin/SharepointEmailWS.asmx?wsdl sulam.org.il null Israel 0 null null null

1598 http://slk.sulam.org.il/_vti_bin/People.asmx?wsdl sulam.org.il null Israel 0 null null null

1599 http://slk.sulam.org.il/_vti_bin/Authentication.asmx?wsdl sulam.org.il null Israel 0 null null null

1600 http://portal.idc.ac.il/en/main/about_idc/news_events/_vti_bin/BusinessDataCatalog.asmx?wsdl idc.ac.il 194.153.101.3 Israel 3264832771 AS34886 The Insterdisciplinary Center Hertzelia 31.5 34.75

1601 http://portal.idc.ac.il/_vti_bin/SharepointEmailWS.asmx?wsdl idc.ac.il 194.153.101.3 Israel 3264832771 AS34886 The Insterdisciplinary Center Hertzelia 31.5 34.75

1602 http://portal.idc.ac.il/en/main/about_idc/news_events/_vti_bin/Authentication.asmx?wsdl idc.ac.il 194.153.101.3 Israel 3264832771 AS34886 The Insterdisciplinary Center Hertzelia 31.5 34.75

1603 http://portal.idc.ac.il/en/main/about_idc/news_events/_vti_bin/People.asmx?wsdl idc.ac.il 194.153.101.3 Israel 3264832771 AS34886 The Insterdisciplinary Center Hertzelia 31.5 34.75

1604 http://www.invoice4u.co.il/Public/w_company.asmx?wsdl invoice4u.co.il 213.8.27.246 Israel 3574078454 AS5486 Euronet Digital Communications, (1992) LTD, Israel 31.5 34.75

1605 http://www.invoice4u.co.il/Public/w_invoicereceipt.asmx?wsdl invoice4u.co.il 213.8.27.246 Israel 3574078454 AS5486 Euronet Digital Communications, (1992) LTD, Israel 31.5 34.75

1606 http://www.invoice4u.co.il/Public/w_items.asmx?WSDL invoice4u.co.il 213.8.27.246 Israel 3574078454 AS5486 Euronet Digital Communications, (1992) LTD, Israel 31.5 34.75

1607 http://www.invoice4u.co.il/Public/w_invoice.asmx?WSDL invoice4u.co.il 213.8.27.246 Israel 3574078454 AS5486 Euronet Digital Communications, (1992) LTD, Israel 31.5 34.75

1608 http://www.invoice4u.co.il/Public/w_receipt.asmx?WSDL invoice4u.co.il 213.8.27.246 Israel 3574078454 AS5486 Euronet Digital Communications, (1992) LTD, Israel 31.5 34.75

1609 http://www.invoice4u.co.il/Public/w_currency.asmx?WSDL invoice4u.co.il 213.8.27.246 Israel 3574078454 AS5486 Euronet Digital Communications, (1992) LTD, Israel 31.5 34.75

1610 http://www.invoice4u.co.il/Public/w_invoicedeal.asmx?WSDL invoice4u.co.il 213.8.27.246 Israel 3574078454 AS5486 Euronet Digital Communications, (1992) LTD, Israel 31.5 34.75

1611 http://www.invoice4u.co.il/Public/w_invoicecredit.asmx?WSDL invoice4u.co.il 213.8.27.246 Israel 3574078454 AS5486 Euronet Digital Communications, (1992) LTD, Israel 31.5 34.75

1612 http://b144.co.il/Services/CityService.asmx?WSDL b144.co.il 147.235.246.154 Israel 2481714842 AS6810 Bezek The Israeli Communications Company Ltd 31.5 34.75

1613 http://sms.cellcom.co.il/SmsGate/SmsGate.asmx?wsdl cellcom.co.il 192.118.28.52 Israel 3228965940 AS9117 Cellcom Israel Ltd. 31.5 34.75

1614 http://sms.cellcom.co.il/SmsGate/SmsGate2.asmx?WSDL cellcom.co.il 192.118.28.52 Israel 3228965940 AS9117 Cellcom Israel Ltd. 31.5 34.75

1615 http://ws.d.co.il/wallaapi/service1.asmx?wsdl d.co.il 212.117.156.55 Israel 3564477495 AS9116 012 Smile Communications Main Autonomous System 31.5 34.75

1616 http://hook.co.il/webServices/mngMyProfile.asmx?WSDL hook.co.il 213.8.145.111 Israel 3574108527 AS5486 Euronet Digital Communications, (1992) LTD, Israel 31.5 34.75

1617 http://hook.co.il/webServices/blogBookmarkService.asmx?WSDL hook.co.il 213.8.145.111 Israel 3574108527 AS5486 Euronet Digital Communications, (1992) LTD, Israel 31.5 34.75

1618 http://wap.icellcom.co.il/SmsGate/SmsGate2.asmx?wsdl icellcom.co.il 192.118.28.11 Israel 3228965899 AS9117 Cellcom Israel Ltd. 31.5 34.75

1619 http://wap.icellcom.co.il/SmsGate/SmsGate.asmx?WSDL icellcom.co.il 192.118.28.11 Israel 3228965899 AS9117 Cellcom Israel Ltd. 31.5 34.75

1620 http://www.inforu.co.il/inforufrontend/CPMWebService/service.asmx?WSDL inforu.co.il 192.114.70.93 Israel 3228714589 AS8551 Bezeqint Internet Backbone 31.5 34.75

1621 http://www.inforu.co.il/inforufrontend/webinterface/webservice.asmx?WSDL inforu.co.il 192.114.70.93 Israel 3228714589 AS8551 Bezeqint Internet Backbone 31.5 34.75

1622 http://webservices.macam.ac.il/MacamMailer/MacamMailer.asmx?wsdl macam.ac.il 192.115.100.173 Israel 3228787885 AS3288 MACAM-MOFET Institute, Israel 31.5 34.75

1623 http://webservices.macam.ac.il/librarySearch/QueryBuilder.asmx?wsdl macam.ac.il 192.115.100.173 Israel 3228787885 AS3288 MACAM-MOFET Institute, Israel 31.5 34.75

1624 http://1444.co.il/WSAjax.asmx?WSDL 1444.co.il null Israel 0 null null null

1625 http://orange.3gxxx.co.il/Ivr23gWs/wService.asmx?WSDL 3gxxx.co.il 213.8.153.99 Israel 3574110563 AS5486 Euronet Digital Communications, (1992) LTD, Israel 31.5 34.75

1626 http://www.arikbensimhon.com/SlideShow.asmx?WSDL arikbensimhon.com 173.247.246.5 Israel 2918708741 null 34.1173 -118.26

1627 http://www.chat.beok.co.il/ws/BeOK_2_CB.asmx?WSDL beok.co.il 82.80.198.195 Israel 1381025475 AS8551 Bezeqint Internet Backbone 31.5 34.75

1628 http://www.derech-haim.co.il/ClalitService/ClalitService.asmx?wsdl derech-haim.co.il 62.90.118.176 Israel 1046116016 AS1680 013 NetVision Ltd. 31.5 34.75

1629 http://www.ibit.co.il/ibit/IBitStorage.asmx?WSDL ibit.co.il 213.8.193.60 Israel 3574120764 AS5486 Euronet Digital Communications, (1992) LTD, Israel 31.5 34.75

1630 http://www.motocar.co.il/MotocarWS/PriceList.asmx?WSDL motocar.co.il 80.178.161.21 Israel 1353883925 AS9116 012 Smile Communications Main Autonomous System 31.5 34.75

1631 http://www.salkkl.org.il/KklShoppingWs/ClientService.asmx?wsdl salkkl.org.il 192.114.182.155 Israel 3228743323 AS5486 Euronet Digital Communications, (1992) LTD, Israel 31.8969 34.8186

1632 http://www.sii.org.il/Templates/PRODUCT_APPROVAL/ProductsAndManufacturerWS.asmx?WSDL sii.org.il 199.203.226.2 Israel 3352027650 AS1680 013 NetVision Ltd. 32.1508 34.8883

1633 http://lobbyreal.spadester.com:4300/LobbyWS.asmx?WSDL spadester.com 174.132.162.155 Israel 2927927963 AS21844 ThePlanet.com Internet Services, Inc. 29.7523 -95.367

1634 http://usm.unicell.co.il/sms.asmx?WSDL unicell.co.il 212.179.112.110 Israel 3568529518 AS8551 Bezeqint Internet Backbone 31.5 34.75

1635 http://www.zim.co.il/SendMail.asmx?WSDL zim.co.il 91.235.35.36 Israel 1542136612 null 51 9

1636 http://bioinformatics.istge.it:8080/axis/services/cabri.getYeastsById.derived?wsdl istge.it null Italy 0 null null null

1637 http://bioinformatics.istge.it:8080/axis/services/o2i.getP53SampleNames.derived?wsdl istge.it null Italy 0 null null null

1638 http://bioinformatics.istge.it:8080/axis/services/o2i.getP53MutationFunctionIdsByMutaa.derived?wsdl istge.it null Italy 0 null null null

1639 http://bioinformatics.istge.it:8080/axis/services/p53_getz_services.getP53IdsByField.derived?wsdl istge.it null Italy 0 null null null

1640 http://bioinformatics.istge.it:8080/axis/services/o2i.getP53MutationsByIds.derived?wsdl istge.it null Italy 0 null null null

1641 http://bioinformatics.istge.it:8080/axis/services/o2i.getP53WtaaByIds.derived?wsdl istge.it null Italy 0 null null null

1642 http://bioinformatics.istge.it:8080/axis/services/o2i.getP53RefIdByIds.derived?wsdl istge.it null Italy 0 null null null

1643 http://bioinformatics.istge.it:8080/axis/services/o2i.getP53PubMedIdByIds.derived?wsdl istge.it null Italy 0 null null null

1644 http://bioinformatics.istge.it:8080/axis/services/o2i.getP53MutationsBySampleName.derived?wsdl istge.it null Italy 0 null null null

1645 http://bioinformatics.istge.it:8080/axis/services/o2i.getP53MutationIdsByType.derived?wsdl istge.it null Italy 0 null null null

1646 http://bioinformatics.istge.it:8080/axis/services/o2i.getP53MutationIdsByTumorOrigin.derived?wsdl istge.it null Italy 0 null null null

1647 http://bioinformatics.istge.it:8080/axis/services/o2i.getP53MutationIdsByProperty.derived?wsdl istge.it null Italy 0 null null null

1648 http://bioinformatics.istge.it:8080/axis/services/o2i.getP53MutationIdsByMetastasisLocalization.derived?wsdl istge.it null Italy 0 null null null

1649 http://bioinformatics.istge.it:8080/axis/services/o2i.getP53MutationIdsByExon.derived?wsdl istge.it null Italy 0 null null null

1650 http://bioinformatics.istge.it:8080/axis/services/o2i.getP53MutationIdsByEffect.derived?wsdl istge.it null Italy 0 null null null

1651 http://bioinformatics.istge.it:8080/axis/services/o2i.getP53MutationIdsByCpgSite.derived?wsdl istge.it null Italy 0 null null null

1652 http://bioinformatics.istge.it:8080/axis/services/o2i.getP53MutationIdsByCodonNumber.derived?wsdl istge.it null Italy 0 null null null

1653 http://bioinformatics.istge.it:8080/axis/services/o2i.getP53MutationFunctionIdsByCodonNumber.derived?wsdl istge.it null Italy 0 null null null

1654 http://bioinformatics.istge.it:8080/axis/services/o2i.getP53MutationFunctionEntryById.derived?wsdl istge.it null Italy 0 null null null

1655 http://bioinformatics.istge.it:8080/axis/services/o2i.getP53MutationFunctionEntry.derived?wsdl istge.it null Italy 0 null null null

1656 http://bioinformatics.istge.it:8080/axis/services/o2i.getP53MutaaByIds.derived?wsdl istge.it null Italy 0 null null null

1657 http://bioinformatics.istge.it:8080/axis/services/cabri.getYeastIdsByName.derived?wsdl istge.it null Italy 0 null null null

1658 http://bioinformatics.istge.it:8080/axis/services/o2i.getP53CodonNumberByIds.derived?wsdl istge.it null Italy 0 null null null

1659 http://bioinformatics.istge.it:8080/axis/services/cabri_getz_services.getCabriFieldByField.derived?wsdl istge.it null Italy 0 null null null

1660 http://bioinformatics.istge.it:8080/axis/services/cabri.getPhagesById.derived?wsdl istge.it null Italy 0 null null null

1661 http://bioinformatics.istge.it:8080/axis/services/o2i.getMutaaByIds.derived?wsdl istge.it null Italy 0 null null null

1662 http://bioinformatics.istge.it:8080/axis/services/cabri.getResourcesById.derived?wsdl istge.it null Italy 0 null null null

1663 http://bioinformatics.istge.it:8080/axis/services/gowlab.echo.derived?wsdl istge.it null Italy 0 null null null

1664 http://bioinformatics.istge.it:8080/axis/services/classic.helloworld.derived?wsdl istge.it null Italy 0 null null null

1665 http://bioinformatics.istge.it:8080/axis/services/cabri_getz_services.getCabriIdsByField.derived?wsdl istge.it null Italy 0 null null null

1666 http://bioinformatics.istge.it:8080/axis/services/o2i.getP53CellPropertyByRefIdAndSampleName.derived?wsdl istge.it null Italy 0 null null null

1667 http://bioinformatics.istge.it:8080/axis/services/cabri.getFungiById.derived?wsdl istge.it null Italy 0 null null null

1668 http://bioinformatics.istge.it:8080/axis/services/cabri.getCellLineByName.derived?wsdl istge.it null Italy 0 null null null

1669 http://bioinformatics.istge.it:8080/axis/services/o2i.getIdsByMutaa.derived?wsdl istge.it null Italy 0 null null null

1670 http://bioinformatics.istge.it:8080/axis/services/cabri_getz_services.getCabriCellLinesEntriesByIds.derived?wsdl istge.it null Italy 0 null null null

1671 http://bioinformatics.istge.it:8080/axis/services/AnalysisFactory?wsdl istge.it null Italy 0 null null null

1672 http://bioinformatics.istge.it:8080/axis/services/o2i.getP53CodonNumbersWtaaMutaaById.derived?wsdl istge.it null Italy 0 null null null

1673 http://bioinformatics.istge.it:8080/axis/services/cabri_getz_services.getCabriEntriesByField.derived?wsdl istge.it null Italy 0 null null null

1674 http://bioinformatics.istge.it:8080/axis/services/cabri.getFungiIdsByName.derived?wsdl istge.it null Italy 0 null null null

1675 http://bioinformatics.istge.it:8080/axis/services/Version?wsdl istge.it null Italy 0 null null null

1676 http://bioinformatics.istge.it:8080/axis/services/cabri_getz_services.getCabriBacteriaEntriesByIds.derived?wsdl istge.it null Italy 0 null null null

1677 http://bioinformatics.istge.it:8080/axis/services/cabri.getBacteriaIdsByProperty.derived?wsdl istge.it null Italy 0 null null null

1678 http://bioinformatics.istge.it:8080/axis/services/cabri.getCellLinesById.derived?wsdl istge.it null Italy 0 null null null

1679 http://bioinformatics.istge.it:8080/axis/services/cabri.getPhageIdsByProperty.derived?wsdl istge.it null Italy 0 null null null

1680 http://bioinformatics.istge.it:8080/axis/services/o2i.getCodonNumberByIds.derived?wsdl istge.it null Italy 0 null null null

1681 http://bioinformatics.istge.it:8080/axis/services/o2i.getP53CellLinesStatusEntryById.derived?wsdl istge.it null Italy 0 null null null

1682 http://bioinformatics.istge.it:8080/axis/services/graphics.dot.derived?wsdl istge.it null Italy 0 null null null

1683 http://bioinformatics.istge.it:8080/axis/services/cabri.getPlasmidIdsByName.derived?wsdl istge.it null Italy 0 null null null

1684 http://bioinformatics.istge.it:8080/axis/services/cabri.getPlasmidIdsByProperty.derived?wsdl istge.it null Italy 0 null null null

1685 http://bioinformatics.istge.it:8080/axis/services/gowlab.helloworld.derived?wsdl istge.it null Italy 0 null null null

1686 http://bioinformatics.istge.it:8080/axis/services/cabri.getCellLineNames.derived?wsdl istge.it null Italy 0 null null null

1687 http://bioinformatics.istge.it:8080/axis/services/o2i.getIdsByCodonNumber.derived?wsdl istge.it null Italy 0 null null null

1688 http://bioinformatics.istge.it:8080/axis/services/cabri.getBacteriaIdsByName.derived?wsdl istge.it null Italy 0 null null null

1689 http://bioinformatics.istge.it:8080/axis/services/cabri.getPlasmidsById.derived?wsdl istge.it null Italy 0 null null null

1690 http://bioinformatics.istge.it:8080/axis/services/cabri.getResourceIdsByName.derived?wsdl istge.it null Italy 0 null null null

1691 http://bioinformatics.istge.it:8080/axis/services/cabri.getYeastIdsByProperty.derived?wsdl istge.it null Italy 0 null null null

1692 http://bioinformatics.istge.it:8080/axis/services/cabri.getPhageIdsByName.derived?wsdl istge.it null Italy 0 null null null

1693 http://bioinformatics.istge.it:8080/axis/services/cabri.getCellLineIdsByName.derived?wsdl istge.it null Italy 0 null null null

1694 http://bioinformatics.istge.it:8080/axis/services/cabri.getP53MutationsBySampleName.derived?wsdl istge.it null Italy 0 null null null

1695 http://bioinformatics.istge.it:8080/axis/services/cabri.getCellLineIdsByProperty.derived?wsdl istge.it null Italy 0 null null null

1696 http://bioinformatics.istge.it:8080/axis/services/cabri.getFungiIdsByProperty.derived?wsdl istge.it null Italy 0 null null null

1697 http://bioinformatics.istge.it:8080/axis/services/testing.yeastgrid.derived?wsdl istge.it null Italy 0 null null null

1698 http://bioinformatics.istge.it:8080/axis/services/testing.medlinesrs.derived?wsdl istge.it null Italy 0 null null null

1699 http://bioinformatics.istge.it:8080/axis/services/testing.emblsrs.derived?wsdl istge.it null Italy 0 null null null

1700 http://www.confcooperative.it/_vti_bin/searchwsdl.aspx confcooperative.it 89.97.191.53 Italy 1499578165 AS12874 Fastweb SpA 42.8333 12.8333

1701 http://www.confcooperative.it/_vti_bin/AreaServicewsdl.aspx confcooperative.it 89.97.191.53 Italy 1499578165 AS12874 Fastweb SpA 42.8333 12.8333

1702 http://www.confcooperative.it/_vti_bin/Webswsdl.aspx confcooperative.it 89.97.191.53 Italy 1499578165 AS12874 Fastweb SpA 42.8333 12.8333

1703 http://www.confcooperative.it/_vti_bin/versionswsdl.aspx confcooperative.it 89.97.191.53 Italy 1499578165 AS12874 Fastweb SpA 42.8333 12.8333

1704 http://www.confcooperative.it/_vti_bin/OutlookAdapterwsdl.aspx confcooperative.it 89.97.191.53 Italy 1499578165 AS12874 Fastweb SpA 42.8333 12.8333

1705 http://www.confcooperative.it/_vti_bin/DWSwsdl.aspx confcooperative.it 89.97.191.53 Italy 1499578165 AS12874 Fastweb SpA 42.8333 12.8333

1706 http://www.confcooperative.it/_vti_bin/Imagingwsdl.aspx confcooperative.it 89.97.191.53 Italy 1499578165 AS12874 Fastweb SpA 42.8333 12.8333

1707 http://www.confcooperative.it/_vti_bin/UserGroupwsdl.aspx confcooperative.it 89.97.191.53 Italy 1499578165 AS12874 Fastweb SpA 42.8333 12.8333

1708 http://www.confcooperative.it/_vti_bin/Viewswsdl.aspx confcooperative.it 89.97.191.53 Italy 1499578165 AS12874 Fastweb SpA 42.8333 12.8333

1709 http://www.confcooperative.it/_vti_bin/Meetingswsdl.aspx confcooperative.it 89.97.191.53 Italy 1499578165 AS12874 Fastweb SpA 42.8333 12.8333

1710 http://www.confcooperative.it/_vti_bin/Formswsdl.aspx confcooperative.it 89.97.191.53 Italy 1499578165 AS12874 Fastweb SpA 42.8333 12.8333

1711 http://www.confcooperative.it/_vti_bin/Permissionswsdl.aspx confcooperative.it 89.97.191.53 Italy 1499578165 AS12874 Fastweb SpA 42.8333 12.8333

1712 http://www.confcooperative.it/_vti_bin/alertswsdl.aspx confcooperative.it 89.97.191.53 Italy 1499578165 AS12874 Fastweb SpA 42.8333 12.8333

1713 http://www.confcooperative.it/_vti_bin/webpartpageswsdl.aspx confcooperative.it 89.97.191.53 Italy 1499578165 AS12874 Fastweb SpA 42.8333 12.8333

1714 http://www.confcooperative.it/_vti_bin/DspStswsdl.aspx confcooperative.it 89.97.191.53 Italy 1499578165 AS12874 Fastweb SpA 42.8333 12.8333

1715 http://www.confcooperative.it/_vti_bin/Listswsdl.aspx confcooperative.it 89.97.191.53 Italy 1499578165 AS12874 Fastweb SpA 42.8333 12.8333

1716 http://www.agrotrace.coop/_vti_bin/AreaServicewsdl.aspx agrotrace.coop null Italy 0 null null null

1717 http://www.agrotrace.coop/_vti_bin/searchwsdl.aspx agrotrace.coop null Italy 0 null null null

1718 http://www.agrotrace.coop/_vti_bin/DspStswsdl.aspx agrotrace.coop null Italy 0 null null null

1719 http://www.agrotrace.coop/_vti_bin/Viewswsdl.aspx agrotrace.coop null Italy 0 null null null

1720 http://www.agrotrace.coop/_vti_bin/Webswsdl.aspx agrotrace.coop null Italy 0 null null null

1721 http://www.agrotrace.coop/_vti_bin/UserGroupwsdl.aspx agrotrace.coop null Italy 0 null null null

1722 http://www.agrotrace.coop/_vti_bin/versionswsdl.aspx agrotrace.coop null Italy 0 null null null

1723 http://www.agrotrace.coop/_vti_bin/Permissionswsdl.aspx agrotrace.coop null Italy 0 null null null

1724 http://www.agrotrace.coop/_vti_bin/DWSwsdl.aspx agrotrace.coop null Italy 0 null null null

1725 http://www.agrotrace.coop/_vti_bin/webpartpageswsdl.aspx agrotrace.coop null Italy 0 null null null

1726 http://www.agrotrace.coop/_vti_bin/OutlookAdapterwsdl.aspx agrotrace.coop null Italy 0 null null null

1727 http://www.agrotrace.coop/_vti_bin/Listswsdl.aspx agrotrace.coop null Italy 0 null null null

1728 http://www.agrotrace.coop/_vti_bin/Meetingswsdl.aspx agrotrace.coop null Italy 0 null null null

1729 http://www.agrotrace.coop/_vti_bin/alertswsdl.aspx agrotrace.coop null Italy 0 null null null

1730 http://www.agrotrace.coop/_vti_bin/Imagingwsdl.aspx agrotrace.coop null Italy 0 null null null

1731 http://www.serviziocivile.coop/_vti_bin/UserProfileServicewsdl.aspx serviziocivile.coop null Italy 0 null null null

1732 http://www.serviziocivile.coop/_vti_bin/AreaServicewsdl.aspx serviziocivile.coop null Italy 0 null null null

1733 http://www.serviziocivile.coop/_vti_bin/DWSwsdl.aspx serviziocivile.coop null Italy 0 null null null

1734 http://www.serviziocivile.coop/_vti_bin/Listswsdl.aspx serviziocivile.coop null Italy 0 null null null

1735 http://www.serviziocivile.coop/_vti_bin/versionswsdl.aspx serviziocivile.coop null Italy 0 null null null

1736 http://www.serviziocivile.coop/_vti_bin/OutlookAdapterwsdl.aspx serviziocivile.coop null Italy 0 null null null

1737 http://www.serviziocivile.coop/_vti_bin/Formswsdl.aspx serviziocivile.coop null Italy 0 null null null

1738 http://www.serviziocivile.coop/_vti_bin/UserGroupwsdl.aspx serviziocivile.coop null Italy 0 null null null

1739 http://www.serviziocivile.coop/_vti_bin/Viewswsdl.aspx serviziocivile.coop null Italy 0 null null null

1740 http://www.serviziocivile.coop/_vti_bin/alertswsdl.aspx serviziocivile.coop null Italy 0 null null null

1741 http://www.serviziocivile.coop/_vti_bin/webpartpageswsdl.aspx serviziocivile.coop null Italy 0 null null null

1742 http://www.serviziocivile.coop/_vti_bin/DspStswsdl.aspx serviziocivile.coop null Italy 0 null null null

1743 http://www.serviziocivile.coop/_vti_bin/Imagingwsdl.aspx serviziocivile.coop null Italy 0 null null null

1744 http://www.serviziocivile.coop/_vti_bin/Permissionswsdl.aspx serviziocivile.coop null Italy 0 null null null

1745 http://www.serviziocivile.coop/_vti_bin/Webswsdl.aspx serviziocivile.coop null Italy 0 null null null

1746 http://www.serviziocivile.coop/_vti_bin/Meetingswsdl.aspx serviziocivile.coop null Italy 0 null null null

1747 http://rotor.di.unipi.it/_vti_bin/People.asmx?wsdl unipi.it null Italy 0 null null null

1748 http://rotor.di.unipi.it/_vti_bin/Authentication.asmx?wsdl unipi.it null Italy 0 null null null

1749 http://webservices.tiscali.com/EmailServices.asmx?wsdl tiscali.com 213.205.32.10 Italy 3586990090 AS8612 Tiscali Italia SpA. 42.8333 12.8333

1750 http://webservices.tiscali.com/CreditCardServices.asmx?wsdl tiscali.com 213.205.32.10 Italy 3586990090 AS8612 Tiscali Italia SpA. 42.8333 12.8333

1751 http://webservices.tiscali.com/SecurityServices.asmx?wsdl tiscali.com 213.205.32.10 Italy 3586990090 AS8612 Tiscali Italia SpA. 42.8333 12.8333

1752 http://sec.ts.astro.it/sec_server2.php?wsdl astro.it null Italy 0 null null null

1753 http://vamos.na.astro.it:8080/axis/services/solarnetNode?wsdl astro.it null Italy 0 null null null

1754 http://vamos.na.astro.it:8080/axis/services/Version?wsdl astro.it null Italy 0 null null null

1755 http://vamos.na.astro.it:8080/axis/services/vamosws?wsdl astro.it null Italy 0 null null null

1756 http://vamos.na.astro.it:8080/axis/services/dsows?wsdl astro.it null Italy 0 null null null

1757 http://services.laureloak.it/TicketSystem.asmx?WSDL laureloak.it 62.149.209.116 Italy 1050005876 AS31034 Aruba S.p.A. - Network 42.8333 12.8333

1758 http://services.laureloak.it/MyMailingService.asmx?WSDL laureloak.it 62.149.209.116 Italy 1050005876 AS31034 Aruba S.p.A. - Network 42.8333 12.8333

1759 http://services.laureloak.it/SmsSender.asmx?WSDL laureloak.it 62.149.209.116 Italy 1050005876 AS31034 Aruba S.p.A. - Network 42.8333 12.8333

1760 http://services.laureloak.it/LocalIndex.asmx?WSDL laureloak.it 62.149.209.116 Italy 1050005876 AS31034 Aruba S.p.A. - Network 42.8333 12.8333

1761 http://services.laureloak.it/BillingLicense.asmx?WSDL laureloak.it 62.149.209.116 Italy 1050005876 AS31034 Aruba S.p.A. - Network 42.8333 12.8333

1762 http://services.laureloak.it/MyContactService.asmx?WSDL laureloak.it 62.149.209.116 Italy 1050005876 AS31034 Aruba S.p.A. - Network 42.8333 12.8333

1763 http://unione.collidivini.it/Knos/ws/Folder.asmx?WSDL collidivini.it null Italy 0 null null null

1764 http://www.esfresh.com/ServeiLastFm.asmx?wsdl esfresh.com 64.74.223.45 Italy 1078648621 AS21740 eNom, Incorporated 33.7516 -84.3915

1765 http://www.esfresh.com/ServeiLyricWiki.asmx?WSDL esfresh.com 64.74.223.45 Italy 1078648621 AS21740 eNom, Incorporated 33.7516 -84.3915

1766 http://www.esfresh.com/ServeiYoutube.asmx?WSDL esfresh.com 64.74.223.45 Italy 1078648621 AS21740 eNom, Incorporated 33.7516 -84.3915

1767 http://www.esfresh.com/ServeiBD.asmx?WSDL esfresh.com 64.74.223.45 Italy 1078648621 AS21740 eNom, Incorporated 33.7516 -84.3915

1768 http://www.esfresh.com/ServeisAuxiliars.asmx?WSDL esfresh.com 64.74.223.45 Italy 1078648621 AS21740 eNom, Incorporated 33.7516 -84.3915

1769 http://www.sms.mio.it/eniwrapper/sendmessages.asmx?wsdl mio.it 62.149.193.143 Italy 1050001807 AS31034 Aruba S.p.A. - Network 42.8333 12.8333

1770 http://www.sms.mio.it/eniwrapper/sendmessagesex.asmx?wsdl mio.it 62.149.193.143 Italy 1050001807 AS31034 Aruba S.p.A. - Network 42.8333 12.8333

1771 http://www.sms.mio.it/cwswrapper/credit.asmx?WSDL mio.it 62.149.193.143 Italy 1050001807 AS31034 Aruba S.p.A. - Network 42.8333 12.8333

1772 http://www.sms.mio.it/cwswrapper/users.asmx?WSDL mio.it 62.149.193.143 Italy 1050001807 AS31034 Aruba S.p.A. - Network 42.8333 12.8333

1773 http://webservices.mio.it/sendsms.asmx?WSDL mio.it 62.149.193.143 Italy 1050001807 AS31034 Aruba S.p.A. - Network 42.8333 12.8333

1774 http://62.108.228.60:8080/axis/services/Version?wsdl 62.108.228.60 62.108.228.60 Italy 1047323708 AS25518 ZUCCHETTI SPA 42.8333 12.8333

1775 http://62.108.228.60:8080/axis/services/urn:WebServiceServerGW?wsdl 62.108.228.60 62.108.228.60 Italy 1047323708 AS25518 ZUCCHETTI SPA 42.8333 12.8333

1776 http://glue.cefriel.it/glue/services/PublishWSD2?WSDL cefriel.it 85.18.70.232 Italy 1427261160 AS12874 Fastweb SpA 45.4667 9.2

1777 http://glue.cefriel.it/glue/services/SubmitGoal2?WSDL cefriel.it 85.18.70.232 Italy 1427261160 AS12874 Fastweb SpA 45.4667 9.2

1778 http://www.cm-ponzone.al.it/Knos/ws/Folder.asmx?WSDL cm-ponzone.al.it null Italy 0 null null null

1779 http://devlab.devleap.it/PaoloPi/wsCached/wsCached.asmx?wsdl devleap.it null Italy 0 null null null

1780 http://devlab.devleap.it/PaoloPi/ws01/wsTest01.asmx?WSDL devleap.it null Italy 0 null null null

1781 http://clienti.skypost.it/users.asmx?wsdl skypost.it 50.17.185.255 Italy 840022527 null 39.0437 -77.4875

1782 http://clienti.skypost.it/sendmessages.asmx?WSDL skypost.it 50.17.185.255 Italy 840022527 null 39.0437 -77.4875

1783 http://ws.smshosting.it/smsWS/services/smsService?wsdl smshosting.it 194.242.61.15 Italy 3270655247 AS24994 genesys informatica srl 43.7667 11.25

1784 http://ws.smshosting.it/smsWebService/ManageUser?WSDL smshosting.it 194.242.61.15 Italy 3270655247 AS24994 genesys informatica srl 43.7667 11.25

1785 http://ws.smshosting.it/smsWebService/ManagePhoneBook?WSDL smshosting.it 194.242.61.15 Italy 3270655247 AS24994 genesys informatica srl 43.7667 11.25

1786 http://ws.smshosting.it/smsWebService/ManageSms?wsdl smshosting.it 194.242.61.15 Italy 3270655247 AS24994 genesys informatica srl 43.7667 11.25

1787 http://www.unionedeicastelli.it/Knos/ws/Folder.asmx?WSDL unionedeicastelli.it 198.1.124.182 Italy 3321986230 null 40.2181 -111.6133

1788 http://www.cmlemmeovadese.it/Knos/ws/Folder.asmx?WSDL cmlemmeovadese.it 83.103.23.208 Italy 1399265232 AS12874 Fastweb SpA 44.4167 8.95

1789 http://www.collinarevalcerrina.it/Knos/ws/Folder.asmx?WSDL collinarevalcerrina.it 83.103.23.208 Italy 1399265232 AS12874 Fastweb SpA 44.4167 8.95

1790 http://demo.istat.it/sodi/rapida.php?wsdl istat.it null Italy 0 null null null

1791 http://miz.it/GeoServices.asmx?WSDL miz.it 83.103.36.222 Italy 1399268574 AS12874 Fastweb SpA 45.05 7.6667

1792 http://www.sipeaa.it/wset/ServiceET.asmx?wsdl sipeaa.it 213.174.178.114 Italy 3584995954 AS21309 ACANTHO SPA 44.4833 11.3333

1793 http://www.sipeaa.it/gsrad/CRA.clima.webservices.GSRad.asmx?wsdl sipeaa.it 213.174.178.114 Italy 3584995954 AS21309 ACANTHO SPA 44.4833 11.3333

1794 http://www.sipeaa.it/rain/CRA.clima.webservices.rain.asmx?wsdl sipeaa.it 213.174.178.114 Italy 3584995954 AS21309 ACANTHO SPA 44.4833 11.3333

1795 http://62.149.230.17/webservicecustomer/service1.asmx?WSDL 62.149.230.17 62.149.230.17 Italy 1050011153 AS31034 Aruba S.p.A. - Network 42.8333 12.8333

1796 http://62.149.230.17/remoteinterface.asmx?WSDL 62.149.230.17 62.149.230.17 Italy 1050011153 AS31034 Aruba S.p.A. - Network 42.8333 12.8333

1797 http://drm.esperia.com/dvdit/licenseservice.asmx?WSDL esperia.com null Italy 0 null null null

1798 http://drm.esperia.com/mibservice.asmx?wsdl esperia.com null Italy 0 null null null

1799 http://www.ilpalio.siena.it/Palio.asmx?wsdl ilpalio.siena.it 62.149.128.151 Italy 1049985175 AS31034 Aruba S.p.A. - Network 42.8333 12.8333

1800 http://sms.smspoint.net:8080/special/services/Version?wsdl smspoint.net 46.28.2.27 Italy 773587483 AS1267 Infostrada S.p.A. 41.9 12.4833

1801 http://www.tou.it/WebServices/webserv.asmx?WSDL tou.it 85.94.212.234 Italy 1432278250 AS12637 Seeweb Srl 42.8333 12.8333

1802 http://www.tou.it/WebServices/MathService.asmx?WSDL tou.it 85.94.212.234 Italy 1432278250 AS12637 Seeweb Srl 42.8333 12.8333

1803 http://mymemory.translated.net/otms/?wsdl translated.net 31.169.105.126 Italy 531196286 null 42.8333 12.8333

1804 http://www.translated.net/hts/soap.php?wsdl translated.net 31.169.105.126 Italy 531196286 null 42.8333 12.8333

1805 http://api.transvector2.it/products.asmx?Wsdl transvector2.it 2.228.17.162 Italy 48501154 null 45.4667 9.2

1806 http://sms.admdomini.com/ws.php?wsdl admdomini.com 195.225.171.26 Italy 3286346522 AS31034 Aruba S.p.A. - Network 42.8333 12.8333

1807 http://www.alongoni.it/vatitaws/VatitaWS.asmx?WSDL alongoni.it 62.149.128.166 Italy 1049985190 AS31034 Aruba S.p.A. - Network 42.8333 12.8333

1808 http://www.anagrafenazionalericerche.it/arianna/App_Services/WebServiceAutocomplete/Autocomplete.asmx?WSDL anagrafenazionalericerche.it null Italy 0 null null null

1809 http://configs.bancadellarete.it/newwsgetconfig/wsgetconfig.asmx?WSDL bancadellarete.it null Italy 0 null null null

1810 http://www.boxinfo.net/cyberWeb.asmx?WSDL boxinfo.net 62.149.128.154 Italy 1049985178 AS31034 Aruba S.p.A. - Network 42.8333 12.8333

1811 http://braocaffe.it/services/Services.asmx?WSDL braocaffe.it 95.110.204.193 Italy 1601096897 AS31034 Aruba S.p.A. - Network 42.8333 12.8333

1812 http://www.capezzano.org/cyberweb.asmx?WSDL capezzano.org 62.149.128.154 Italy 1049985178 AS31034 Aruba S.p.A. - Network 42.8333 12.8333

1813 http://sms.comi.it/ws.php?wsdl comi.it 194.185.119.234 Italy 3266934762 AS3313 I.NET S.p.A. 42.8333 12.8333

1814 http://www.dominopoint.it/dominopoint/download/web_service_mag.nsf/controllomagazzino?WSDL dominopoint.it 194.79.57.47 Italy 3259971887 AS39217 ELMEC-AS 45.8 8.8333

1815 http://api.eurocv.eu/euroserver.php?wsdl eurocv.eu 109.168.116.234 Italy 1839756522 AS5602 KPNQwest Italia S.p.a 42.8333 12.8333

1816 http://www.fborghi.it/Services/Office.asmx?wsdl fborghi.it 62.149.128.72 Italy 1049985096 AS31034 Aruba S.p.A. - Network 42.8333 12.8333

1817 http://beta.fibs.it/WS/AC.asmx?WSDL fibs.it 81.29.201.73 Italy 1360906569 AS15830 TELECITYGROUP INTERNATIONAL LIMITED 42.8333 12.8333

1818 http://www.gesacom.it/info.asmx?WSDL gesacom.it 62.149.128.157 Italy 1049985181 AS31034 Aruba S.p.A. - Network 42.8333 12.8333

1819 http://www.giochisportivi.it/gsws_nic.asmx?WSDL giochisportivi.it null Italy 0 null null null

1820 http://monitor.gruppodev.it/monitor.asmx?WSDL gruppodev.it 2.234.36.237 Italy 48899309 null 44.4833 11.3333

1821 http://guru4.net/articoli/javascript-soap-client/demo/webservicedemo.asmx?WSDL guru4.net 93.57.10.88 Italy 1564019288 AS12874 Fastweb SpA 42.8333 12.8333

1822 http://webservices.imel.eu/MastercardAutenticazione.asmx?WSDL imel.eu 82.115.173.114 Italy 1383312754 AS30958 BASCOM S.p.a. - Bergamo 45.6833 9.7167

1823 http://www.inevoluzione.com/webservices/prodotti.asmx?WSDL inevoluzione.com 37.59.12.67 Italy 624626755 null 48.86 2.35

1824 http://www.info-me-sms.it/ws.php?wsdl info-me-sms.it 212.31.225.62 Italy 3558859070 AS8220 COLT Technology Services 45.4667 9.2

1825 http://www.maitaly.it/maws20/default.asmx?WSDL maitaly.it 193.148.38.146 Italy 3247711890 AS13180 CedacriNord S.p.a. 44.75 10.2167

1826 http://manhattanshop.it/webservices/DynamicWebParts.asmx?WSDL manhattanshop.it 46.137.92.78 Italy 780753998 null 53 -8

1827 http://mide.mkom.it/ws_ti/service.asmx?wsdl mkom.it null Italy 0 null null null

1828 http://www.mutuiplanet.it/PlanetService/Service.asmx?WSDL mutuiplanet.it 81.29.196.118 Italy 1360905334 AS15830 TELECITYGROUP INTERNATIONAL LIMITED 42.8333 12.8333

1829 http://www.palestranewline.com/cyberWeb.asmx?WSDL palestranewline.com 62.149.128.157 Italy 1049985181 AS31034 Aruba S.p.A. - Network 42.8333 12.8333

1830 http://www.piccadillyhotel.it/NWs.asmx?wsdl piccadillyhotel.it 93.95.219.125 Italy 1566563197 AS3313 I.NET S.p.A. 46.55 11.7667

1831 http://www.portalemediatori.it/custom/locale/LocaleComuni.asmx?WSDL portalemediatori.it 213.140.5.248 Italy 3582723576 AS12874 Fastweb SpA 42.8333 12.8333

1832 http://www.progamma.com/NWindWS/NorthwindWS.asmx?WSDL progamma.com 95.110.197.154 Italy 1601095066 AS31034 Aruba S.p.A. - Network 42.8333 12.8333

1833 http://www.pubblicaamministrazione.net/wsScript.asmx?WSDL pubblicaamministrazione.net 151.1.244.178 Italy 2533487794 AS3242 ITnet S.p.A. Autonomous System 44.4167 8.95

1834 http://www.robertobeccari.it/webservices/examples/helloworld.asmx?WSDL robertobeccari.it 62.149.128.151 Italy 1049985175 AS31034 Aruba S.p.A. - Network 42.8333 12.8333

1835 http://www.robertobertoni.com/cyberWeb.asmx?WSDL robertobertoni.com 62.149.128.160 Italy 1049985184 AS31034 Aruba S.p.A. - Network 42.8333 12.8333

1836 http://www.sestanteinformatica.it/cyberWeb.asmx?WSDL sestanteinformatica.it 62.149.128.157 Italy 1049985181 AS31034 Aruba S.p.A. - Network 42.8333 12.8333

1837 http://www.stemband.net/cyberWeb.asmx?WSDL stemband.net null Italy 0 null null null

1838 http://www.studioat.eu/ArcGIS/Services?wsdl studioat.eu 195.110.128.11 Italy 3278798859 AS5396 MC-link Spa 42.8333 12.8333

1839 http://www.studiobasso.com/ws/cifrelettere.asmx?wsdl studiobasso.com 62.149.128.151 Italy 1049985175 AS31034 Aruba S.p.A. - Network 42.8333 12.8333

1840 http://www.subitosms.it/ws.php?wsdl subitosms.it 212.31.238.82 Italy 3558862418 AS8220 COLT Technology Services 45.4667 9.2

1841 http://www.superenal8.it/ws/ws.php?wsdl superenal8.it null Italy 0 null null null

1842 http://www.vdeejay.com/WSVDJ.ASMX?wsdl vdeejay.com null Italy 0 null null null

1843 http://biomoby.org/services/wsdl/www.nias.affrc.go.jp/getGeneByGO affrc.go.jp null Japan 0 null null null

1844 http://pc120.narc.affrc.go.jp/jboss-net/services/CountryServer?WSDL affrc.go.jp null Japan 0 null null null

1845 http://pc120.narc.affrc.go.jp/jboss-net/services/ResourceServerUserService?wsdl affrc.go.jp null Japan 0 null null null

1846 http://fsdb.dc.affrc.go.jp/axis/services/Version?wsdl affrc.go.jp null Japan 0 null null null

1847 http://fsdb.dc.affrc.go.jp/axis/services/FSDBService?wsdl affrc.go.jp null Japan 0 null null null

1848 http://fsdb.dc.affrc.go.jp/axis/services/FertilizerService?wsdl affrc.go.jp null Japan 0 null null null

1849 http://fsdb.dc.affrc.go.jp/axis/services/NouyakuService?wsdl affrc.go.jp null Japan 0 null null null

1850 http://www.vietden.net/WebService/Dictionary100.asmx?WSDL vietden.net null Japan 0 null null null

1851 http://www.vietden.net/WebService/ZipSearch100.asmx?WSDL vietden.net null Japan 0 null null null

1852 http://www.vietden.net/WebService/SimpleDic.asmx?WSDL vietden.net null Japan 0 null null null

1853 http://www.vietden.net/WebService/RomajiKanaConvert100.asmx?WSDL vietden.net null Japan 0 null null null

1854 http://www.vietden.net/WebService/Calendar100.asmx?WSDL vietden.net null Japan 0 null null null

1855 http://www.vietden.net/WebService/Calendar.asmx?WSDL vietden.net null Japan 0 null null null

1856 http://www.vietden.net/WebService/VnCodeConvert.asmx?WSDL vietden.net null Japan 0 null null null

1857 http://210.170.63.7/cs/Xcelerate/wsdl/SitePlan.wsdl 210.170.63.7 210.170.63.7 Japan 3534372615 AS2519 VECTANT Ltd. 35.69 139.69

1858 http://210.170.63.7/cs/Xcelerate/wsdl/AssetSet.wsdl 210.170.63.7 210.170.63.7 Japan 3534372615 AS2519 VECTANT Ltd. 35.69 139.69

1859 http://210.170.63.7/cs/Xcelerate/wsdl/AssetType.wsdl 210.170.63.7 210.170.63.7 Japan 3534372615 AS2519 VECTANT Ltd. 35.69 139.69

1860 http://210.170.63.7/cs/Xcelerate/wsdl/Miscellaneous.wsdl 210.170.63.7 210.170.63.7 Japan 3534372615 AS2519 VECTANT Ltd. 35.69 139.69

1861 http://www.agmodel.org/jboss-net/services/CountryServer?wsdl agmodel.org 150.26.5.120 Japan 2518287736 AS18125 Ministry of Agriculture, Forestry and Fisheries Research Network 35.69 139.69

1862 http://www.agmodel.org/jboss-net/services/ResourceServerUserService?wsdl agmodel.org 150.26.5.120 Japan 2518287736 AS18125 Ministry of Agriculture, Forestry and Fisheries Research Network 35.69 139.69

1863 http://www.agmodel.org/jboss-net/services/Version?wsdl agmodel.org 150.26.5.120 Japan 2518287736 AS18125 Ministry of Agriculture, Forestry and Fisheries Research Network 35.69 139.69

1864 http://www.agmodel.org/MetBroker/MetSOAP/MetSOAP.wsdl agmodel.org 150.26.5.120 Japan 2518287736 AS18125 Ministry of Agriculture, Forestry and Fisheries Research Network 35.69 139.69

1865 http://www.iwebmethod.net/icd1.0/icd.asmx?wsdl iwebmethod.net 210.230.192.182 Japan 3538337974 AS4732 DION KDDI CORPORATION 35.685 139.7514

1866 http://www.iwebmethod.net/netdict/003/whatstime2.asmx?WSDL iwebmethod.net 210.230.192.182 Japan 3538337974 AS4732 DION KDDI CORPORATION 35.685 139.7514

1867 http://www.iwebmethod.net/helloworld/helloworld.asmx?wsdl iwebmethod.net 210.230.192.182 Japan 3538337974 AS4732 DION KDDI CORPORATION 35.685 139.7514

1868 http://www.iwebmethod.net/counter1.0/counter.asmx?WSDL iwebmethod.net 210.230.192.182 Japan 3538337974 AS4732 DION KDDI CORPORATION 35.685 139.7514

1869 http://biomoby.org/services/wsdl/rings.t.soka.ac.jp/getSimilarGlycansWithScoreFromKCF soka.ac.jp 150.37.112.110 Japan 2519036014 AS4725 SOFTBANK TELECOM Corp. 35.69 139.69

1870 http://biomoby.org/services/wsdl/rings.t.soka.ac.jp/getSimilarGlycansWithScore soka.ac.jp 150.37.63.6 Japan 2519023366 AS4725 SOFTBANK TELECOM Corp. 35.69 139.69

1871 http://biomoby.org/services/wsdl/rings.t.soka.ac.jp/getSimilarGlycans soka.ac.jp 150.37.56.48 Japan 2519021616 AS4725 SOFTBANK TELECOM Corp. 35.685 139.7514

1872 http://biomoby.org/services/wsdl/rings.t.soka.ac.jp/RINGStest soka.ac.jp 150.37.34.6 Japan 2519015942 AS4725 SOFTBANK TELECOM Corp. 35.69 139.69

1873 http://biomoby.org/services/wsdl/rings.t.soka.ac.jp/getKCFFromGlycanID soka.ac.jp 150.37.160.54 Japan 2519048246 AS4725 SOFTBANK TELECOM Corp. 35.69 139.69

1874 http://pdbjs3.protein.osaka-u.ac.jp/gash/soap/gash.wsdl osaka-u.ac.jp null Japan 0 null null null

1875 http://www.ps.noda.tus.ac.jp/axis/services/Version?wsdl tus.ac.jp null Japan 0 null null null

1876 http://www.ps.noda.tus.ac.jp/axis/services/metadb?wsdl tus.ac.jp null Japan 0 null null null

1877 http://bearmini.net/publicholidays/JP.asmx?WSDL bearmini.net 204.93.156.223 Japan 3428687071 AS23352 Server Central Network 41.8776 -87.6272

1878 http://dev.geoap.jp/geoap_match/geoap_trial.asmx?wsdl geoap.jp 210.166.222.110 Japan 3534151278 AS7678 Prox System Design Inc. 35.69 139.69

1879 http://dev.geoap.jp/GeOAP_Trial/GeOAP_Trial.asmx?wsdl geoap.jp 210.166.222.110 Japan 3534151278 AS7678 Prox System Design Inc. 35.69 139.69

1880 http://www.loftwork.com/common/service/PortfolioService.asmx?WSDL loftwork.com 54.248.104.44 Japan 922249260 null 35.685 139.7514

1881 http://www.dis.h.u-tokyo.ac.jp/webservices/dpcsearch.asmx?WSDL u-tokyo.ac.jp null Japan 0 null null null

1882 http://www.dis.h.u-tokyo.ac.jp/webservices/byomeisearch.asmx?wsdl u-tokyo.ac.jp null Japan 0 null null null

1883 http://1175.jp:8080/google/PostalCodes.asmx?WSDL 1175.jp null Japan 0 null null null

1884 http://s.ebeta.jp/shipping/service.wsdl ebeta.jp null Japan 0 null null null

1885 http://www.hi-ho.ne.jp/cgi-bin/user/illusia/cgibin20_test.cgi?op=WSDL hi-ho.ne.jp 202.224.157.71 Japan 3403717959 AS2497 Internet Initiative Japan Inc. 35.69 139.69

1886 http://hasegawa.la-piace.jp/ShopService.asmx?wsdl la-piace.jp null Japan 0 null null null

1887 http://www.one-five.info/PublicWebService/Service.asmx?wsdl one-five.info null Japan 0 null null null

1888 http://pdbj.protein.osaka-u.ac.jp/SOAP/PDBjSoapService.wsdl pdbj.org 202.223.160.83 Japan 3403653203 AS2907 Research Organization of Information and Systems, National Institute of Informatics 35.69 139.69

1889 http://www.rarestyle.net/QRService/QRService.asmx?WSDL rarestyle.net 219.117.197.108 Japan 3681928556 AS2514 NTT PC Communications, Inc. 35.69 139.69

1890 http://www.s-parts.co.jp/Math/FormulaService.asmx?WSDL s-parts.co.jp 202.143.64.63 Japan 3398385727 AS17941 Bit-isle Co.,Ltd. 35.685 139.7514

1891 http://tekisaku.jp/tekisakuws/invokeHyouka.asmx?WSDL tekisaku.jp 150.26.38.38 Japan 2518296102 AS18125 Ministry of Agriculture, Forestry and Fisheries Research Network 35.69 139.69

1892 http://wikipedia-lab.org:8080/WikipediaOntologyAPIv3/Service.asmx?wsdl wikipedia-lab.org 176.34.48.35 Japan 2955030563 null 35.685 139.7514

1893 http://www.yo-ki.com/software/yzipcode/YZipCode.asmx?wsdl yo-ki.com 202.171.136.26 Japan 3400239130 AS2514 NTT PC Communications, Inc. 35.685 139.7514

1894 http://www.medinfo.kz/jaxrpc-dps/dps?WSDL medinfo.kz 89.218.40.44 Kazakhstan 1507469356 AS9198 JSC Kazakhtelecom 43.25 76.95

1895 http://62.215.172.203/GatewayWebService.asmx?WSDL 62.215.172.203 62.215.172.203 Kuwait 1054321867 AS21050 Fast Telecommunications Company W.L.L. 29.3697 47.9783

1896 http://mail.future.lv:8080/digitaltimesarchive/DocumentService.asmx?wsdl future.lv 193.108.185.101 Latvia 3245128037 AS12993 Digitalas Ekonomikas Attistibas Centrs Autonomous System 57 25

1897 http://distance.ktu.lt/moodle/sqi/sessionmgt.php?wsdl ktu.lt 158.129.0.8 Lithuania 2659254280 AS2847 LITNET, Lithuanian Academic and Research Network 54.9 23.9

1898 http://vertimas.vdu.lt/twsas/Services/TWService.asmx?WSDL vdu.lt 193.219.38.37 Lithuania 3252364837 AS2847 LITNET, Lithuanian Academic and Research Network 54.9 23.9

1899 http://dataconnector.eea.europa.eu/SoapServices/Services.asmx?wsdl europa.eu 147.67.119.102 Denmark 2470672230 AS42848 European Commission 49.75 6.1667

1900 http://www.mca.org.my/_vti_bin/People.asmx?wsdl mca.org.my 210.5.43.123 Malaysia 3523554171 AS45352 IP ServerOne Solutions Sdn Bhd, 2.5 112.5

1901 http://www.mca.org.my/_vti_bin/Authentication.asmx?wsdl mca.org.my 210.5.43.123 Malaysia 3523554171 AS45352 IP ServerOne Solutions Sdn Bhd, 2.5 112.5

1902 http://58.71.128.51:9300/mm7/services/Version?wsdl 58.71.128.51 58.71.128.51 Malaysia 977764403 AS9534 Binariang Berhad 2.5 112.5

1903 http://www.guinness.com.my/staging/databaseWS.asmx?WSDL guinness.com.my 124.150.140.116 Malaysia 2090241140 AS24218 Global Transit Communications - Malaysia 2.5 112.5

1904 http://blogs.timovil.com/_vti_bin/SharepointEmailWS.asmx?wsdl timovil.com 207.248.251.138 Mexico 3489201034 AS11172 Alestra, S. de R.L. de C.V. 25.6667 -100.4

1905 http://blogs.timovil.com/_vti_bin/BusinessDataCatalog.asmx?wsdl timovil.com 207.248.251.138 Mexico 3489201034 AS11172 Alestra, S. de R.L. de C.V. 25.6667 -100.4

1906 http://blogs.timovil.com/_vti_bin/People.asmx?wsdl timovil.com 207.248.251.138 Mexico 3489201034 AS11172 Alestra, S. de R.L. de C.V. 25.6667 -100.4

1907 http://blogs.timovil.com/_vti_bin/Authentication.asmx?wsdl timovil.com 207.248.251.138 Mexico 3489201034 AS11172 Alestra, S. de R.L. de C.V. 25.6667 -100.4

1908 http://tvazteca.viajez.com/WServicesDev/TicketRetrieve?WSDL viajez.com 107.21.26.39 Mexico 1796545063 null 39.0437 -77.4875

1909 http://tvazteca.viajez.com/WServicesDev/RetrievePNR?WSDL viajez.com 107.21.26.39 Mexico 1796545063 null 39.0437 -77.4875

1910 http://tvazteca.viajez.com/WServicesDev/CancelPNR?WSDL viajez.com 107.21.26.39 Mexico 1796545063 null 39.0437 -77.4875

1911 http://www.iingen.unam.mx/Contactenos/_vti_bin/dws.asmx?WSDL iingen.unam.mx null Mexico 0 null null null

1912 http://www.iingen.unam.mx/_vti_bin/Lists.asmx?wsdl iingen.unam.mx null Mexico 0 null null null

1913 http://maya.ccg.unam.mx:8080/axis/services/Version?wsdl ccg.unam.mx null Mexico 0 null null null

1914 http://maya.ccg.unam.mx:8080/axis/TFmodellerService.jws?WSDL ccg.unam.mx null Mexico 0 null null null

1915 http://avaluos.infonavit.gob.mx/DirectorioWebServices/services/EnvioOfertas/wsdl/EnvioOfertas.wsdl infonavit.gob.mx null Mexico 0 null null null

1916 http://200.13.89.5/CHKDOR_Service/CHKDOR.asmx?wsdl 200.13.89.5 200.13.89.5 Mexico 3356317957 AS21603 Universidad La Salle, AC 19.4342 -99.1386

1917 http://200.77.237.252/seshat2/ws/adminUsuario.php?wsdl 200.77.237.252 200.77.237.252 Mexico 3360550396 AS22908 Sixsigma Networks Mexico, S.A. de C.V. 17.8833 -91.4667

1918 http://200.79.42.56/WebServiceSCG/services/WebServiceSCG/wsdl/WebServiceSCG.wsdl 200.79.42.56 200.79.42.56 Mexico 3360631352 AS8151 Uninet S.A. de C.V. 19.4342 -99.1386

1919 http://201.147.155.231/RespuestaSeps/service.asmx?WSDL 201.147.155.231 201.147.155.231 Mexico 3381894119 AS8151 Uninet S.A. de C.V. 19.43 -99.13

1920 http://smn2.cna.gob.mx/webservicessmn/service1.asmx?WSDL cna.gob.mx 201.116.60.80 Mexico 3379838032 AS8151 Uninet S.A. de C.V. 19.4342 -99.1386

1921 http://correo.ucol.mx/webservice/wscorreo.php?wsdl correo.ucol.mx 148.213.1.5 Mexico 2496987397 AS15236 Universidad de Colima 19.2333 -103.7167

1922 http://www.edc.com.mx/EdcTotalWebService/EdcTotalWebService.asmx?wsdl edc.com.mx 148.243.35.11 Mexico 2498962187 AS6503 Axtel, S.A.B. de C.V. 25.6667 -100.4

1923 http://www.pfizer.co.cr/B2B/webservice/b2bwssincronizacion.asmx?WSDL pfizer.co.cr null Mexico 0 null null null

1924 http://creed.vera.net/Autentica/autentica.asmx?WSDL vera.net null Mexico 0 null null null

1925 http://www.expeditiesos.eu/_vti_bin/SpellCheck.asmx?wsdl expeditiesos.eu null Netherlands 0 null null null

1926 http://www.expeditiesos.eu/_vti_bin/BusinessDataCatalog.asmx?wsdl expeditiesos.eu null Netherlands 0 null null null

1927 http://www.expeditiesos.eu/_vti_bin/SharepointEmailWS.asmx?wsdl expeditiesos.eu null Netherlands 0 null null null

1928 http://www.expeditiesos.eu/_vti_bin/People.asmx?wsdl expeditiesos.eu null Netherlands 0 null null null

1929 http://www.expeditiesos.eu/_vti_bin/Authentication.asmx?wsdl expeditiesos.eu null Netherlands 0 null null null

1930 http://www.onderwijsweb.nl/_vti_bin/Authentication.asmx?wsdl onderwijsweb.nl 213.154.250.220 Netherlands 3583703772 AS12859 BIT BV 52.5 5.75

1931 http://www.onderwijsweb.nl/_vti_bin/People.asmx?wsdl onderwijsweb.nl 213.154.250.220 Netherlands 3583703772 AS12859 BIT BV 52.5 5.75

1932 http://ws.adaptivedisclosure.org/axis/services/tokenize?wsdl adaptivedisclosure.org 86.89.110.167 Netherlands 1448701607 AS8737 KPN Internet Solutions 52.3297 4.9248

1933 http://ws.adaptivedisclosure.org/axis/services/getWordnetSynWS?wsdl adaptivedisclosure.org 86.89.110.167 Netherlands 1448701607 AS8737 KPN Internet Solutions 52.3297 4.9248

1934 http://ws.adaptivedisclosure.org/axis/services/NERecognizerService?wsdl adaptivedisclosure.org 86.89.110.167 Netherlands 1448701607 AS8737 KPN Internet Solutions 52.3297 4.9248

1935 http://ws.adaptivedisclosure.org/axis/services/CRFtrain?wsdl adaptivedisclosure.org 86.89.110.167 Netherlands 1448701607 AS8737 KPN Internet Solutions 52.3297 4.9248

1936 http://ws.adaptivedisclosure.org/axis/services/getTermVector?wsdl adaptivedisclosure.org 86.89.110.167 Netherlands 1448701607 AS8737 KPN Internet Solutions 52.3297 4.9248

1937 http://ws.adaptivedisclosure.org/axis/services/CRFapply?wsdl adaptivedisclosure.org 86.89.110.167 Netherlands 1448701607 AS8737 KPN Internet Solutions 52.3297 4.9248

1938 http://ws.adaptivedisclosure.org/axis/services/getFields?wsdl adaptivedisclosure.org 86.89.110.167 Netherlands 1448701607 AS8737 KPN Internet Solutions 52.3297 4.9248

1939 http://ws.adaptivedisclosure.org/axis/services/synonym?wsdl adaptivedisclosure.org 86.89.110.167 Netherlands 1448701607 AS8737 KPN Internet Solutions 52.3297 4.9248

1940 http://ws.adaptivedisclosure.org/axis/services/TestModel?wsdl adaptivedisclosure.org 86.89.110.167 Netherlands 1448701607 AS8737 KPN Internet Solutions 52.3297 4.9248

1941 http://ws.adaptivedisclosure.org/axis/services/getOnlineAcronymsWS?wsdl adaptivedisclosure.org 86.89.110.167 Netherlands 1448701607 AS8737 KPN Internet Solutions 52.3297 4.9248

1942 http://ws.adaptivedisclosure.org/axis/services/Version?wsdl adaptivedisclosure.org 86.89.110.167 Netherlands 1448701607 AS8737 KPN Internet Solutions 52.3297 4.9248

1943 http://ws.adaptivedisclosure.org/axis/services/spellCheck?wsdl adaptivedisclosure.org 86.89.110.167 Netherlands 1448701607 AS8737 KPN Internet Solutions 52.3297 4.9248

1944 http://ws.adaptivedisclosure.org/axis/services/RelationExtractor?wsdl adaptivedisclosure.org 86.89.110.167 Netherlands 1448701607 AS8737 KPN Internet Solutions 52.3297 4.9248

1945 http://www.avs.nl/_vti_bin/BusinessDataCatalog.asmx?wsdl avs.nl 46.23.73.139 Netherlands 773278091 null 52.35 4.9167

1946 http://www.avs.nl/_vti_bin/People.asmx?wsdl avs.nl 46.23.73.139 Netherlands 773278091 null 52.35 4.9167

1947 http://www.avs.nl/_vti_bin/Authentication.asmx?wsdl avs.nl 46.23.73.139 Netherlands 773278091 null 52.35 4.9167

1948 http://blogs.code-counsel.net/_vti_bin/SharepointEmailWS.asmx?wsdl code-counsel.net 157.55.238.140 Netherlands 2637688460 AS3598 Microsoft Corp 38 -97

1949 http://blogs.code-counsel.net/_vti_bin/UserProfileService.asmx?wsdl code-counsel.net 157.55.238.140 Netherlands 2637688460 AS3598 Microsoft Corp 38 -97

1950 http://blogs.code-counsel.net/_vti_bin/BusinessDataCatalog.asmx?wsdl code-counsel.net 157.55.238.140 Netherlands 2637688460 AS3598 Microsoft Corp 38 -97

1951 http://blogs.code-counsel.net/_vti_bin/People.asmx?wsdl code-counsel.net 157.55.238.140 Netherlands 2637688460 AS3598 Microsoft Corp 38 -97

1952 http://blogs.code-counsel.net/_vti_bin/Authentication.asmx?wsdl code-counsel.net 157.55.238.140 Netherlands 2637688460 AS3598 Microsoft Corp 38 -97

1953 http://www.gvb.nl/_vti_bin/BusinessDataCatalog.asmx?wsdl gvb.nl 195.193.209.15 Netherlands 3284259087 AS702 Verizon Business EMEA - Commercial IP service provider in Europe 52.35 4.9167

1954 http://www.gvb.nl/_vti_bin/Authentication.asmx?wsdl gvb.nl 195.193.209.15 Netherlands 3284259087 AS702 Verizon Business EMEA - Commercial IP service provider in Europe 52.35 4.9167

1955 http://www.gvb.nl/_vti_bin/People.asmx?wsdl gvb.nl 195.193.209.15 Netherlands 3284259087 AS702 Verizon Business EMEA - Commercial IP service provider in Europe 52.35 4.9167

1956 http://www.opf.nl/_vti_bin/BusinessDataCatalog.asmx?wsdl opf.nl null Netherlands 0 null null null

1957 http://www.opf.nl/_vti_bin/People.asmx?wsdl opf.nl null Netherlands 0 null null null

1958 http://www.opf.nl/_vti_bin/Authentication.asmx?wsdl opf.nl null Netherlands 0 null null null

1959 http://www.slinger.nu/_vti_bin/People.asmx?wsdl slinger.nu 217.148.85.99 Netherlands 3650377059 AS16237 Nxs Internet BV 51.7862 4.4377

1960 http://www.slinger.nu/_vti_bin/Authentication.asmx?wsdl slinger.nu 217.148.85.99 Netherlands 3650377059 AS16237 Nxs Internet BV 51.7862 4.4377

1961 http://www.cadcompany.nl/_vti_bin/People.asmx?wsdl cadcompany.nl 82.201.35.23 Netherlands 1388913431 AS15879 IS Interned Services BV Autonomous System 51.7862 4.4377

1962 http://www.cadcompany.nl/_vti_bin/Authentication.asmx?wsdl cadcompany.nl 82.201.35.23 Netherlands 1388913431 AS15879 IS Interned Services BV Autonomous System 51.7862 4.4377

1963 http://www.enkeltjebangkok.nl/_vti_bin/Lists.asmx?wsdl enkeltjebangkok.nl 188.93.150.39 Netherlands 3160249895 AS21155 ProServe B.V. Networks 52.5 5.75

1964 http://ict1.tbm.tudelft.nl:81/spm4341/meteo.asmx?WSDL tudelft.nl 131.180.77.102 Netherlands 2209631590 AS1128 DTO TUDELFT, The Netherlands - AS 52.0186 4.3782

1965 http://ict1.tbm.tudelft.nl:81/spm4341/hazmat.asmx?wsdl tudelft.nl 131.180.77.102 Netherlands 2209631590 AS1128 DTO TUDELFT, The Netherlands - AS 52.0186 4.3782

1966 http://ict1.tbm.tudelft.nl:81/spm4341/inventory.asmx?WSDL tudelft.nl 131.180.77.102 Netherlands 2209631590 AS1128 DTO TUDELFT, The Netherlands - AS 52.0186 4.3782

1967 http://ict1.tbm.tudelft.nl:81/spm4341/recommendation.asmx?wsdl tudelft.nl 131.180.77.102 Netherlands 2209631590 AS1128 DTO TUDELFT, The Netherlands - AS 52.0186 4.3782

1968 http://ict1.tbm.tudelft.nl:81/spm4341/geo.asmx?WSDL tudelft.nl 131.180.77.102 Netherlands 2209631590 AS1128 DTO TUDELFT, The Netherlands - AS 52.0186 4.3782

1969 http://www.echtebakker.nl/_vti_bin/Lists.asmx?wsdl echtebakker.nl 213.193.247.26 Netherlands 3586258714 AS15703 TrueServer BV AS number 52.5 5.75

1970 http://webservices.daehosting.com/services/TemperatureConversions.wso?WSDL daehosting.com null Netherlands 0 null null null

1971 http://webservices.daehosting.com/services/datesservice.wso?WSDL daehosting.com null Netherlands 0 null null null

1972 http://webservices.daehosting.com/services/eleventest.wso?wsdl daehosting.com null Netherlands 0 null null null

1973 http://www.html2xml.nl/Services/Calculator/Version1/Calculator.asmx?WSDL html2xml.nl 77.94.248.183 Netherlands 1298069687 AS21155 ProServe B.V. Networks 52.5 5.75

1974 http://www.flexcourier.nl/TopContent/(A(OsBGrTe0DKuAkxcH3axzyMaroSILT5HcPDgvU2sQErd8cJXgaZ4xEqWU0-mCbHH5PfYsjGJlnxfMuOeswT7eIkblftTMgCoo5EUCsPggdxc1))/Webservices/Public.asmx?WSDL flexcourier.nl 213.125.99.194 Netherlands 3581764546 AS9143 Ziggo - tv, internet, telefoon 52.4607 4.6483

1975 http://www.flexcourier.nl/TopContent/(A(DlbntOlLmhq-sB5RlmI8tu1alHZ8RY3gdkaECM7t4fl3FyjGLz8EzKa7YZdLAhY_M3_QkLsVy5ITYqSjOZUnoyykYe1IF7cApi_Sl_bksT81))/DataFrames/Data.asmx?WSDL flexcourier.nl 213.125.99.194 Netherlands 3581764546 AS9143 Ziggo - tv, internet, telefoon 52.4607 4.6483

1976 http://www.flexrider.eu/TopContent/(A(CTUI1HhwxzaNzsq82Hpt3e_x5Zmfkmh2gjkzLCl3AfgdbF3K0usgyhcdJ0HrBhCQ-b91ERzzO2HvbKd-axtyWtYakXU2Du4NMMFIEi5A-mU1))/Webservices/Public.asmx?WSDL flexrider.eu 213.125.99.194 Netherlands 3581764546 AS9143 Ziggo - tv, internet, telefoon 52.4607 4.6483

1977 http://www.flexrider.eu/TopContent/(A(o0Op0H3ffveb1eQtzUiyWhwQ_v2MFKVRVWD7OVryM7LoO0X54NLqCDAWNVyJyruQTyuQHjxbuMBxMGJm8rZT0rIyDu9wKs1VsZ--lxGT7qY1))/DataFrames/Data.asmx?WSDL flexrider.eu 213.125.99.194 Netherlands 3581764546 AS9143 Ziggo - tv, internet, telefoon 52.4607 4.6483

1978 http://www.flexrider.nl/TopContent/(A(XLa5Bf687mLUoP3HHTozJwE1Y97bQ9iVEqk5eqKoIKU_8R_VE6fdpDNgjybv6KXNRIprlqpYd8ATfuqtpUrNeKu-BF3Tg_YuT7431YYQroQ1))/Webservices/Public.asmx?WSDL flexrider.nl 213.125.99.194 Netherlands 3581764546 AS9143 Ziggo - tv, internet, telefoon 52.4607 4.6483

1979 http://www.flexrider.nl/TopContent/(A(8FwWM5-JdcxkXs6eeVdVkgRasAdOHdXyqs12-C4SQNX5p0kRUEtBstB-9hlu-zyyTRmCSPZ6NLIf_7Z8_V_baIhJDkRKg6Auee6EtR8LXyI1))/DataFrames/Data.asmx?WSDL flexrider.nl 213.125.99.194 Netherlands 3581764546 AS9143 Ziggo - tv, internet, telefoon 52.4607 4.6483

1980 http://www.independer.com/thema/autoverzekering/webservices/autotypeservice.asmx?WSDL independer.com 213.171.136.81 Netherlands 3584788561 AS44253 Nines B.V. 52.5 5.75

1981 http://www.independer.com/thema/common/webservices/contentproviderservice.asmx?WSDL independer.com 213.171.136.81 Netherlands 3584788561 AS44253 Nines B.V. 52.5 5.75

1982 http://www.oorsprong.org/websamples.countryinfo/CountryInfoService.wso?WSDL oorsprong.org 62.177.194.231 Netherlands 1051837159 AS15670 BBNED-AS#1 BBned NV 52.5 5.75

1983 http://www.oorsprong.org/websamples.arendsoog/ArendsoogbooksService.wso?WSDL oorsprong.org 62.177.194.231 Netherlands 1051837159 AS15670 BBNED-AS#1 BBned NV 52.5 5.75

1984 http://www.oorsprong.org/websamples.anagram/anagramservice.wso?WSDL oorsprong.org 62.177.194.231 Netherlands 1051837159 AS15670 BBNED-AS#1 BBned NV 52.5 5.75

1985 http://wetten.overheid.nl/BWBIdService/BWBIdService.wsdl overheid.nl 62.112.232.27 Netherlands 1047586843 AS29311 ASP4all Hosting B.V. 52.5 5.75

1986 http://www.wagenvoort.net/gpcos/webservices/webservice_get_teamsdrivers.php?wsdl wagenvoort.net 77.94.249.159 Netherlands 1298069919 AS21155 ProServe B.V. Networks 52.5 5.75

1987 http://www.wagenvoort.net/gpcos/webservices/webservice_get_standings.php?wsdl wagenvoort.net 77.94.249.159 Netherlands 1298069919 AS21155 ProServe B.V. Networks 52.5 5.75

1988 http://www.wagenvoort.net/gpcos/webservices/webservice_get_seasonresults.php?wsdl wagenvoort.net 77.94.249.159 Netherlands 1298069919 AS21155 ProServe B.V. Networks 52.5 5.75

1989 http://romanadvies.bibliotheek.nl/CompleteAuthorOrTitle.asmx?WSDL bibliotheek.nl 145.100.53.13 Netherlands 2439263501 AS1103 SURFnet, The Netherlands 52.35 4.9167

1990 http://www.flexcourier.eu/TopContent/(A(msRLKNm6vA4Exq5QmclfeTadPW8Xa6350Ctu5snvPwm5ymMFGfQMcTqGJjMht_JSHM9J-MjM2m62Z7V3we8u9ATistT--3omitXnfCIsBAA1))/DataFrames/Data.asmx?WSDL flexcourier.eu 213.125.99.194 Netherlands 3581764546 AS9143 Ziggo - tv, internet, telefoon 52.4607 4.6483

1991 http://www.flextrack.nl/TopContent/(A(wuAB-F21GZeigKVUsaTYliE7XRlqFKA40IKGAmSHkV0EAvQ55LmkmEFL3dYAHPVq0d2y-ty4XxYcK99_J28JR6t9qrcxcJOy6pQFxsfl3RA1))/DataFrames/Data.asmx?WSDL flextrack.nl 213.125.99.194 Netherlands 3581764546 AS9143 Ziggo - tv, internet, telefoon 52.4607 4.6483

1992 http://athena-hi.fontys.nl/users/879014/dotnet/Rekenmachine_server/Service1.asmx?WSDL fontys.nl 145.85.2.220 Netherlands 2438267612 AS1103 SURFnet, The Netherlands 51.45 5.4667

1993 http://athena-hi.fontys.nl/users/169344/Dotnet/Dotnet3/Service.asmx?wsdl fontys.nl 145.85.2.220 Netherlands 2438267612 AS1103 SURFnet, The Netherlands 51.45 5.4667

1994 http://www.icstats.nl/webservices/rapportage.asmx?wsdl icstats.nl 195.20.9.59 Netherlands 3272870203 AS42949 Eatserver.nl 52.5 5.75

1995 http://www.icstats.nl/webservices/beheer.asmx?wsdl icstats.nl 195.20.9.59 Netherlands 3272870203 AS42949 Eatserver.nl 52.5 5.75

1996 http://www.gosolution.com.ar/test_cli/veraz.server.wsdl mine.nu 204.13.248.119 United States 3423467639 AS33517 Dynamic Network Services, Inc. 40.7904 -74.0246

1997 http://hometownnerd.mine.nu/phrasebook/ch09-communication/wsdl-nusoap-server.php/phrasebook/ch09-communication/wsdl-nusoap-server.php?wsdl mine.nu 204.13.248.119 United States 3423467639 AS33517 Dynamic Network Services, Inc. 40.7904 -74.0246

1998 http://www.mircules.com/WS/LanguageWebService/LanguageWebService.asmx?WSDL mircules.com null Netherlands 0 null null null

1999 http://pp2g.tv/home/videoservice.asmx?WSDL pp2g.tv 213.163.83.245 Netherlands 3584250869 AS49544 Interactive3D 52.5 5.75

2000 http://pp2g.tv/tv/userservice1.asmx?WSDL pp2g.tv 213.163.83.245 Netherlands 3584250869 AS49544 Interactive3D 52.5 5.75

2001 http://topsauctions.com/WebServices/LanguageService.asmx?WSDL topsauctions.com 81.93.54.74 Netherlands 1365063242 AS25542 Denit Internet Services B.V. 52.5 5.75

2002 http://topsauctions.com/WebServices/AuctionClosingService.asmx?WSDL topsauctions.com 81.93.54.74 Netherlands 1365063242 AS25542 Denit Internet Services B.V. 52.5 5.75

2003 http://www.ibi.vu.nl/programs/domainationwww/domaination.wsdl vu.nl 62.112.242.16 Netherlands 1047589392 AS29311 ASP4all Hosting B.V. 52.5 5.75

2004 http://www.antonicknight.com/services/grades/Grades.asmx?WSDL antonicknight.com null Netherlands 0 null null null

2005 http://www.asastudent.com/AsaStudentCampus/5031/Frontend.asmx?WSDL asastudent.com 89.105.199.101 Netherlands 1500104549 AS24875 Cleanport B.V. 52.5 5.75

2006 http://shipfinder.bampro.nl/service/gpstrackingwebservice.asmx?WSDL bampro.nl 31.200.209.172 Netherlands 533254572 null 52.5 5.75

2007 http://imac4ritz.cmd-heerlen.nl/services/test123.php?wsdl cmd-heerlen.nl 87.233.1.87 Netherlands 1474888023 AS15703 TrueServer BV AS number 52.5 5.75

2008 http://www.d2is.nl/SlideService.asmx?WSDL d2is.nl null Netherlands 0 null null null

2009 http://www.dataaccess.nl/wk2006/footballpoolwebservice.wso?WSDL dataaccess.nl 84.241.178.196 Netherlands 1425126084 AS20847 IntroWeb Nederland BV, Hengelo (OV), The Netherlands 52.2674 6.7897

2010 http://www.dmcreseller.nl/callcenter/webservice/MYService.asmx?wsdl dmcreseller.nl 213.207.96.77 Netherlands 3587137613 AS9150 InterConnect Services BV 51.7167 5.3667

2011 http://test.dynapax.nl/index.asmx?wsdl dynapax.nl null Netherlands 0 null null null

2012 http://webservice.globalsportsmedia.com/index.php?wsdl globalsportsmedia.com 87.83.27.82 Netherlands 1465064274 AS4589 Easynet Global Services 52.6333 -2.5

2013 http://www.imperasoft.be/webservicegeneral.asmx?wsdl imperasoft.be 77.94.248.199 Netherlands 1298069703 AS21155 ProServe B.V. Networks 52.5 5.75

2014 http://www.kartpagina.nl/KPWebservice.asmx?wsdl kartpagina.nl 77.94.248.253 Netherlands 1298069757 AS21155 ProServe B.V. Networks 52.5 5.75

2015 http://wshc.libralab.nl/hc.asmx?WSDL libralab.nl 82.201.100.166 Netherlands 1388930214 AS15879 IS Interned Services BV Autonomous System 51.7862 4.4377

2016 http://seadatanet.maris2.nl/ws/ws_edmo.asmx?wsdl maris2.nl 212.189.40.84 Netherlands 3569166420 AS12469 Infonet Netherlands Internet Services 52.5 5.75

2017 http://mds-cd.net/services/MDSFlashService?wsdl mds-cd.net 213.206.102.201 Netherlands 3587073737 AS12573 WideXS / ion-ip 52.35 4.9167

2018 http://www.objectmap.nl/WebService/MyMapService.asmx?WSDL objectmap.nl 85.17.170.28 Netherlands 1427221020 AS16265 LEASEWEB LEASEWEB AS 52.35 4.9167

2019 http://www.partypeeps2000.com/pp2g/userservice1.asmx?WSDL partypeeps2000.com 213.163.83.245 Netherlands 3584250869 AS49544 Interactive3D 52.5 5.75

2020 http://www.pggmlevensloopcalculator.nl/CalculatorService.asmx?WSDL pggmlevensloopcalculator.nl null Netherlands 0 null null null

2021 http://www.pp2g.com/pp2g/userservice1.asmx?WSDL pp2g.com 213.163.83.245 Netherlands 3584250869 AS49544 Interactive3D 52.5 5.75

2022 http://www.simmetric.nl/Blogs/BlogService.asmx?WSDL simmetric.nl 77.94.248.253 Netherlands 1298069757 AS21155 ProServe B.V. Networks 52.5 5.75

2023 http://smellinckx.be/Webservices/Smellinckx.asmx?WSDL smellinckx.be 77.94.248.197 Netherlands 1298069701 AS21155 ProServe B.V. Networks 52.5 5.75

2024 http://12use.stoas.nl/12Use-WS/12/12UseSOAPServices.asmx?WSDL stoas.nl 213.136.6.104 Netherlands 3582461544 AS12859 BIT BV 52.5 5.75

2025 http://www.sukelluskohteet.com/soap-server.php?wsdl sukelluskohteet.com null Netherlands 0 null null null

2026 http://demo.totalweb.nl/DVService/DVService.asmx?WSDL totalweb.nl 81.93.54.74 Netherlands 1365063242 AS25542 Denit Internet Services B.V. 52.5 5.75

2027 http://www.vedior.com/Custom/Services/JobSearchHelper.asmx?WSDL vedior.com 212.83.216.177 Netherlands 3562264753 AS9150 InterConnect Services BV 52.5 5.75

2028 http://api.veritate.net/axis/services/Version?wsdl veritate.net 80.69.72.62 Netherlands 1346717758 AS20857 TransIP BV 52.5 5.75

2029 http://lithp.websystems.nl/wsdl/hallo.wsdl websystems.nl 195.190.0.39 Netherlands 3284008999 AS15879 IS Interned Services BV Autonomous System 52.5 5.75

2030 http://desktop.zoekwekker.nl/DesktopSearchService.asmx?WSDL zoekwekker.nl 193.93.173.155 Netherlands 3244141979 AS39700 Serverius AS 52.5 5.75

2031 http://www.kinetics.co.nz/_vti_bin/BusinessDataCatalog.asmx?wsdl kinetics.co.nz 116.199.216.100 New Zealand 1959254116 AS55454 Business Online Ltd -36.8667 174.7667

2032 http://www.kinetics.co.nz/_vti_bin/People.asmx?wsdl kinetics.co.nz 116.199.216.100 New Zealand 1959254116 AS55454 Business Online Ltd -36.8667 174.7667

2033 http://www.kinetics.co.nz/_vti_bin/Authentication.asmx?wsdl kinetics.co.nz 116.199.216.100 New Zealand 1959254116 AS55454 Business Online Ltd -36.8667 174.7667

2034 http://www.wssdemo.com/_vti_bin/SharepointEmailWS.asmx?wsdl wssdemo.com null New Zealand 0 null null null

2035 http://www.wssdemo.com/_vti_bin/BusinessDataCatalog.asmx?wsdl wssdemo.com null New Zealand 0 null null null

2036 http://www.wssdemo.com/_vti_bin/People.asmx?wsdl wssdemo.com null New Zealand 0 null null null

2037 http://www.wssdemo.com/_vti_bin/Authentication.asmx?wsdl wssdemo.com null New Zealand 0 null null null

2038 http://www.chandima.net/_vti_bin/People.asmx?wsdl chandima.net null New Zealand 0 null null null

2039 http://www.chandima.net/_vti_bin/Authentication.asmx?wsdl chandima.net null New Zealand 0 null null null

2040 http://dcregistry.natlib.govt.nz/axis/services/recycleLog?wsdl natlib.govt.nz 192.122.171.222 New Zealand 3229264862 AS9338 National Library of New Zealand -41.3 174.7833

2041 http://dcregistry.natlib.govt.nz/axis/services/itemDetail?wsdl natlib.govt.nz 192.122.171.222 New Zealand 3229264862 AS9338 National Library of New Zealand -41.3 174.7833

2042 http://dcregistry.natlib.govt.nz/axis/services/vocabularyTermsSummary?wsdl natlib.govt.nz 192.122.171.222 New Zealand 3229264862 AS9338 National Library of New Zealand -41.3 174.7833

2043 http://dcregistry.natlib.govt.nz/axis/services/elementSummary?wsdl natlib.govt.nz 192.122.171.222 New Zealand 3229264862 AS9338 National Library of New Zealand -41.3 174.7833

2044 http://dcregistry.natlib.govt.nz/axis/services/refinementsSummary?wsdl natlib.govt.nz 192.122.171.222 New Zealand 3229264862 AS9338 National Library of New Zealand -41.3 174.7833

2045 http://dcregistry.natlib.govt.nz/axis/services/termUpdates?wsdl natlib.govt.nz 192.122.171.222 New Zealand 3229264862 AS9338 National Library of New Zealand -41.3 174.7833

2046 http://dcregistry.natlib.govt.nz/axis/services/languagesSummary?wsdl natlib.govt.nz 192.122.171.222 New Zealand 3229264862 AS9338 National Library of New Zealand -41.3 174.7833

2047 http://dcregistry.natlib.govt.nz/axis/services/Version?wsdl natlib.govt.nz 192.122.171.222 New Zealand 3229264862 AS9338 National Library of New Zealand -41.3 174.7833

2048 http://dcregistry.natlib.govt.nz/axis/services/encodingSchemesSummary?wsdl natlib.govt.nz 192.122.171.222 New Zealand 3229264862 AS9338 National Library of New Zealand -41.3 174.7833

2049 http://service.bulletinconnect.net/services/BulletinClient?wsdl bulletinconnect.net 219.88.240.75 New Zealand 3680038987 AS17746 Orcon Internet -36.8667 174.7667

2050 http://ws.eoddata.com/endofday.asmx?WSDL eoddata.com 216.19.206.207 United States 3625176783 AS5784 Getnet International 33.5083 -112.0717

2051 http://ws.eoddata.com/Customers.asmx?WSDL eoddata.com 216.19.206.207 United States 3625176783 AS5784 Getnet International 33.5083 -112.0717

2052 http://ws.eoddata.com/Services.asmx?WSDL eoddata.com 216.19.206.207 United States 3625176783 AS5784 Getnet International 33.5083 -112.0717

2053 http://www.securelink.co.nz/errorloggerdynamo/WebServices/WSFeedback.asmx?WSDL securelink.co.nz 202.0.37.102 New Zealand 3389007206 AS4768 TelstraClear Ltd -43.5333 172.6333

2054 http://www.securelink.co.nz/errorloggerdynamo/WebServices/WSError.asmx?WSDL securelink.co.nz 202.0.37.102 New Zealand 3389007206 AS4768 TelstraClear Ltd -43.5333 172.6333

2055 http://www.champions.co.nz/shop/products2.asmx?WSDL champions.co.nz 202.124.99.34 New Zealand 3397149474 AS23655 Snap Internet Limited -41 174

2056 http://www.craggyrange.co.nz/service.asmx?wsdl craggyrange.co.nz 119.47.125.181 New Zealand 1999601077 AS45459 Web Drive Limited -36.8667 174.7667

2057 http://www.elba.co.nz/services/product.cfc?wsdl elba.co.nz 202.12.105.27 New Zealand 3389810971 AS2687 AT&T Global Network Services - AP -36.8667 174.7667

2058 http://www.goingtothegame.co.nz/AffiliateServices.asmx?wsdl goingtothegame.co.nz null New Zealand 0 null null null

2059 http://andrewsimpson.harcourts.com.au/Controls/Autocomplete.asmx?WSDL harcourts.com.au 103.20.88.1 New Zealand 1729386497 null -43.5333 172.6333

2060 http://pwlcentral.co.nz/onlineorder.asmx?WSDL pwlcentral.co.nz 210.54.238.234 New Zealand 3526815466 AS4771 Netgate -36.8667 174.7667

2061 http://services.rightaddress.co.nz/rightaddress.asmx?wsdl rightaddress.co.nz 202.89.55.103 New Zealand 3394844519 AS9889 Auckland -36.8667 174.7667

2062 http://www.russellhall.com.au/Controls/Autocomplete.asmx?WSDL russellhall.com.au 202.91.7.245 New Zealand 3394963445 AS17477 Macquarie Telecom -27 133

2063 http://www.smirk.co.nz/bizsoap.asmx?WSDL smirk.co.nz null New Zealand 0 null null null

2064 http://youtxt.co.nz/CoreServices.asmx?wsdl youtxt.co.nz 202.89.45.211 New Zealand 3394842067 AS9889 Auckland -36.8667 174.7667

2065 http://www.nybydel.no/_vti_bin/People.asmx?wsdl nybydel.no 80.232.122.94 Norway 1357412958 AS3292 TDC Data Networks 59.9167 10.75

2066 http://www.nybydel.no/_vti_bin/Authentication.asmx?wsdl nybydel.no 80.232.122.94 Norway 1357412958 AS3292 TDC Data Networks 59.9167 10.75

2067 http://www.selvaag.no/_vti_bin/People.asmx?wsdl selvaag.no 80.232.122.94 Norway 1357412958 AS3292 TDC Data Networks 59.9167 10.75

2068 http://www.selvaag.no/_vti_bin/Authentication.asmx?wsdl selvaag.no 80.232.122.94 Norway 1357412958 AS3292 TDC Data Networks 59.9167 10.75

2069 http://prosjekter.selvaagpluss.no/_vti_bin/Authentication.asmx?wsdl selvaagpluss.no 62.148.57.70 Norway 1049901382 AS13243 ErgoGroup AS 59.9167 10.75

2070 http://prosjekter.selvaagpluss.no/Alfadelsol/_vti_bin/People.asmx?wsdl selvaagpluss.no 62.148.57.70 Norway 1049901382 AS13243 ErgoGroup AS 59.9167 10.75

2071 http://www.vintervoll.no/_vti_bin/SharepointEmailWS.asmx?wsdl vintervoll.no 194.63.248.47 Norway 3258972207 AS12996 Domeneshop AS 62 10

2072 http://www.vintervoll.no/_vti_bin/Authentication.asmx?wsdl vintervoll.no 194.63.248.47 Norway 3258972207 AS12996 Domeneshop AS 62 10

2073 http://www.vintervoll.no/_vti_bin/People.asmx?wsdl vintervoll.no 194.63.248.47 Norway 3258972207 AS12996 Domeneshop AS 62 10

2074 http://api.bioinfo.no/wsdl/ELMInstanceMapper.wsdl bioinfo.no 129.177.120.172 Norway 2175891628 AS224 UNINETT, The Norwegian University & Research Network 60.3911 5.3247

2075 http://api.bioinfo.no/wsdl/ELMMatcher.wsdl bioinfo.no 129.177.120.172 Norway 2175891628 AS224 UNINETT, The Norwegian University & Research Network 60.3911 5.3247

2076 http://api.legiomedia.com/Products.asmx?WSDL legiomedia.com 216.121.88.36 United States 3631831076 AS26228 ServePath, LLC 37.7898 -122.3942

2077 http://api.legiomedia.com/Users.asmx?WSDL legiomedia.com 216.121.88.36 United States 3631831076 AS26228 ServePath, LLC 37.7898 -122.3942

2078 http://api.legiomedia.com/Content.asmx?WSDL legiomedia.com 216.121.88.36 United States 3631831076 AS26228 ServePath, LLC 37.7898 -122.3942

2079 http://api.legiomedia.com/Service1.asmx?WSDL legiomedia.com 216.121.88.36 United States 3631831076 AS26228 ServePath, LLC 37.7898 -122.3942

2080 http://api.legiomedia.com/membermanager.asmx?WSDL legiomedia.com 216.121.88.36 United States 3631831076 AS26228 ServePath, LLC 37.7898 -122.3942

2081 http://www.serverside.no/webservices/Currency/Currency.asmx?WSDL serverside.no 81.27.45.29 Norway 1360735517 AS8542 BKK Marked AS 62 10

2082 http://www.serverside.no/webservices/Stock/Stock.asmx?WSDL serverside.no 81.27.45.29 Norway 1360735517 AS8542 BKK Marked AS 62 10

2083 http://www.serverside.no/webservices/Google/Google.asmx?WSDL serverside.no 81.27.45.29 Norway 1360735517 AS8542 BKK Marked AS 62 10

2084 http://www.serverside.no/webservices/ZipCode/ZipCode.asmx?WSDL serverside.no 81.27.45.29 Norway 1360735517 AS8542 BKK Marked AS 62 10

2085 http://webservices.serverside.no/BrReg/BrReg.asmx?WSDL serverside.no 81.27.45.29 Norway 1360735517 AS8542 BKK Marked AS 62 10

2086 http://www.terra.no/_vti_bin/SpellCheck.asmx?wsdl terra.no 94.246.120.159 Norway 1593211039 AS12552 IP-Only Telecommunication Networks AB 62 15

2087 http://www.langlo.no/Services/SearchService.asmx?WSDL langlo.no 81.26.52.50 Norway 1360671794 AS43200 PC Support AS 62.4667 6.15

2088 http://www.langlo.no/Services/ProductService.asmx?WSDL langlo.no 81.26.52.50 Norway 1360671794 AS43200 PC Support AS 62.4667 6.15

2089 http://www.langlo.no/PhotoCatalogService.asmx?WSDL langlo.no 81.26.52.50 Norway 1360671794 AS43200 PC Support AS 62.4667 6.15

2090 http://ksms.webservice.keyteq.no/wappush.php?wsdl keyteq.no 85.200.218.2 Norway 1439226370 AS8542 BKK Marked AS 62 10

2091 http://ams.smsc.vianett.no/v3/cpa/cpawebservice.asmx?WSDL vianett.no 193.142.108.196 Norway 3247336644 AS3307 BaneTele AS (formerly Enitel), Norway 59.4367 10.6692

2092 http://extra.vianett.no/webservices/vianett_sms_service/SMS_Service.asmx?WSDL vianett.no 193.142.108.196 Norway 3247336644 AS3307 BaneTele AS (formerly Enitel), Norway 59.4367 10.6692

2093 http://artskart.artsdatabanken.no/AJAXWS/NavnSok.asmx?WSDL artsdatabanken.no 129.241.18.166 Norway 2180059814 AS224 UNINETT, The Norwegian University & Research Network 63.4167 10.4167

2094 http://donau.hiof.no/borres/dn/service1/ServiceSonette.asmx?WSDL hiof.no 158.39.172.229 Norway 2653400293 AS224 UNINETT, The Norwegian University & Research Network 62 10

2095 http://dom6utv.item.no/Develop/beate/nummernd7.nsf/mywebservice?wsdl item.no 77.88.106.162 Norway 1297640098 AS41572 Hafslund Telekom Nettjenester AS 59.9167 10.75

2096 http://www.item.no/ws/nummernd7.nsf/itemNumber?WSDL item.no 77.88.106.162 Norway 1297640098 AS41572 Hafslund Telekom Nettjenester AS 59.9167 10.75

2097 http://kragstad.com/WebServices/SearchCard.asmx?WSDL kragstad.com 213.188.134.201 Norway 3585902281 AS12994 Active ISP AS 62 10

2098 http://www11.nrk.no/urort/_websvc/TagCompletionWebService.asmx?WSDL nrk.no 160.68.205.231 Norway 2688863719 AS21293 NRK Autonomous System 62 10

2099 http://www11.nrk.no/urort/_websvc/Authentication.asmx?WSDL nrk.no 160.68.205.231 Norway 2688863719 AS21293 NRK Autonomous System 62 10

2100 http://pollenvarslingen.no/webservice/varsel.asmx?WSDL pollenvarslingen.no 128.39.104.113 Norway 2150066289 AS224 UNINETT, The Norwegian University & Research Network 62 10

2101 http://xml.pollenvarslingen.no/pollenvarsel.asmx?WSDL pollenvarslingen.no 128.39.104.113 Norway 2150066289 AS224 UNINETT, The Norwegian University & Research Network 62 10

2102 http://www.audnedal.kommune.no/Layout/Controls/GlobalSearchService.asmx?WSDL audnedal.kommune.no 159.171.96.190 Norway 2678808766 AS3292 TDC Data Networks 58.3167 6.95

2103 http://ferieogfritid.sas.no/GO/WebService/ContentServices_white.asmx?wsdl basefarm.net null Norway 0 null null null

2104 http://ebs.be.no/services/Integration?Wsdl be.no 193.69.20.101 Norway 3242529893 AS2116 Ventelo 59.9167 10.75

2105 http://www.bowling.no/soap/orgdb.wsdl bowling.no 178.79.163.216 Norway 2991563736 AS15830 TELECITYGROUP INTERNATIONAL LIMITED 51.5 -0.13

2106 http://connectorretail.com/wsdrm/service.asmx?wsdl connectorretail.com 88.87.56.30 Norway 1482111006 AS31283 FastHost AS - Norwegian based ISP 62 10

2107 http://elearn.datapower.no/fag/scormapi.asmx?WSDL datapower.no 178.164.2.153 Norway 2997093017 AS34087 NTE Bredband, Norway 63.75 11.3

2108 http://projects.dnv.com/reference_data/RD4Browser/RD4WebServices.asmx?WSDL dnv.com 193.212.132.124 Norway 3251930236 AS2119 T.net 59.9167 10.75

2109 http://www.eidsvoll.kommune.no/Layout/Controls/GlobalSearchService.asmx?WSDL eidsvoll.kommune.no 194.60.75.50 Norway 3258731314 AS42022 Digitale Gardermoen IKS 60.15 11.1833

2110 http://webservices.eschenker.no/PublicMethodes.asmx?WSDL eschenker.no null Norway 0 null null null

2111 http://www.exerto.no/Flash.asmx?wsdl exerto.no 193.30.0.135 Norway 3239968903 AS25351 Broadnet Norge AS, Oslo, Norway 59.9167 10.75

2112 http://services.ifd-library.org/api/2.0?wsdl ifd-library.org 69.27.117.7 Norway 1159427335 AS20218 BlackSun Inc. 52.1193 -106.6594

2113 http://inview.no/Service.asmx?WSDL inview.no 83.143.81.102 Norway 1401901414 AS34989 ServeTheWorld AS - ISP Norway 62 10

2114 http://www.justervesenet.no/Layout/Controls/GlobalSearchService.asmx?WSDL justervesenet.no 77.40.226.114 Norway 1294525042 AS5381 PowerTech Information Systems AS 59.9167 10.75

2115 http://kurl.no/ws/index.php?wsdl kurl.no 129.241.61.61 Norway 2180070717 AS224 UNINETT, The Norwegian University & Research Network 63.4167 10.4167

2116 http://www.livsit.no/service.asmx?wsdl livsit.no 93.94.10.8 Norway 1566444040 AS6829 Sogn og Fjordane fylke 61.1667 6.9167

2117 http://www.lyngdal.kommune.no/Layout/Controls/GlobalSearchService.asmx?WSDL lyngdal.kommune.no 159.171.96.190 Norway 2678808766 AS3292 TDC Data Networks 58.3167 6.95

2118 http://www.nasjonaltjenestekatalog.no/ntkws12/abnsvc?WSDL nasjonaltjenestekatalog.no 195.159.101.192 Norway 3282003392 AS5381 PowerTech Information Systems AS 59.9167 10.75

2119 http://norwegianmafia.no/api/v1.0/norwegianmafia.asmx?WSDL norwegianmafia.no 176.111.193.56 Norway 2960113976 null 62 10

2120 http://procdev.com/zcalcs/zcalcsvc.asmx?WSDL procdev.com 85.252.49.31 Norway 1442591007 AS3307 BaneTele AS (formerly Enitel), Norway 62 10

2121 http://www.sd.no/webservices/onixclws/Query.asmx?WSDL sd.no 195.159.172.111 Norway 3282021487 AS5381 PowerTech Information Systems AS 59.9167 10.75

2122 http://www.tjenestekatalog.no/agent4/service?WSDL tjenestekatalog.no 195.159.101.186 Norway 3282003386 AS5381 PowerTech Information Systems AS 59.9167 10.75

2123 http://www.msm.gov.om/msmwebservices/marketwatch.asmx?wsdl msm.gov.om 82.178.29.19 Oman 1387404563 AS28885 OmanTel NAP 21 57

2124 http://msm.gov.om/ws/MarketSummaryData.asmx?wsdl msm.gov.om 82.178.29.19 Oman 1387404563 AS28885 OmanTel NAP 21 57

2125 http://msm.gov.om/ws/TickerData.asmx?WSDL msm.gov.om 82.178.29.19 Oman 1387404563 AS28885 OmanTel NAP 21 57

2126 http://201.230.192.40/swperu/edsservice.asmx?WSDL 201.230.192.40 201.230.192.40 Peru 3387342888 AS6147 Telefonica del Peru S.A.A. -12.05 -77.05

2127 http://www.coes.org.pe/wsDespacho/wsDespacho.asmx?WSDL coes.org.pe 190.81.112.156 Peru 3193008284 AS12252 Telmex Peru S.A. -12.05 -77.05

2128 http://www.infocajacentral.com/AppBnetEC1/Service1.asmx?WSDL infocajacentral.com 190.81.174.51 Peru 3193024051 AS12252 Telmex Peru S.A. -12.05 -77.05

2129 http://58.69.188.58:8181/WebService/srvcAcknowledgement.asmx?WSDL 58.69.188.58 58.69.188.58 Philippines 977648698 AS9299 Philippine Long Distance Telephone Company 16.45 120.55

2130 http://58.69.188.58:8181/WebService/srvcWorkstationConfiguration.asmx?WSDL 58.69.188.58 58.69.188.58 Philippines 977648698 AS9299 Philippine Long Distance Telephone Company 16.45 120.55

2131 http://58.69.188.58:8181/WebService/srvcRentableTypes.asmx?WSDL 58.69.188.58 58.69.188.58 Philippines 977648698 AS9299 Philippine Long Distance Telephone Company 16.45 120.55

2132 http://58.69.188.58:8181/WebService/srvcRetailsAndRentalsGroup.asmx?WSDL 58.69.188.58 58.69.188.58 Philippines 977648698 AS9299 Philippine Long Distance Telephone Company 16.45 120.55

2133 http://58.69.188.58:8181/WebService/srvcPosition.asmx?WSDL 58.69.188.58 58.69.188.58 Philippines 977648698 AS9299 Philippine Long Distance Telephone Company 16.45 120.55

2134 http://58.69.188.58:8181/WebService/srvcRetailsAndRentals.asmx?WSDL 58.69.188.58 58.69.188.58 Philippines 977648698 AS9299 Philippine Long Distance Telephone Company 16.45 120.55

2135 http://58.69.188.58:8181/WebService/srvcProductAndRetailsLink.asmx?WSDL 58.69.188.58 58.69.188.58 Philippines 977648698 AS9299 Philippine Long Distance Telephone Company 16.45 120.55

2136 http://58.69.188.58:8181/WebService/srvcResources.asmx?WSDL 58.69.188.58 58.69.188.58 Philippines 977648698 AS9299 Philippine Long Distance Telephone Company 16.45 120.55

2137 http://58.69.188.58:8181/WebService/srvcRoyalty.asmx?WSDL 58.69.188.58 58.69.188.58 Philippines 977648698 AS9299 Philippine Long Distance Telephone Company 16.45 120.55

2138 http://58.69.188.58:8181/WebService/srvcUnits.asmx?WSDL 58.69.188.58 58.69.188.58 Philippines 977648698 AS9299 Philippine Long Distance Telephone Company 16.45 120.55

2139 http://58.69.188.58:8181/WebService/srvcEmployees.asmx?WSDL 58.69.188.58 58.69.188.58 Philippines 977648698 AS9299 Philippine Long Distance Telephone Company 16.45 120.55

2140 http://58.69.188.58:8181/WebService/srvcEmployeeGroupsAndEmployeesLink.asmx?WSDL 58.69.188.58 58.69.188.58 Philippines 977648698 AS9299 Philippine Long Distance Telephone Company 16.45 120.55

2141 http://58.69.188.58:8181/WebService/srvcEmployeeGroups.asmx?WSDL 58.69.188.58 58.69.188.58 Philippines 977648698 AS9299 Philippine Long Distance Telephone Company 16.45 120.55

2142 http://58.69.188.58:8181/WebService/srvcDepartments.asmx?WSDL 58.69.188.58 58.69.188.58 Philippines 977648698 AS9299 Philippine Long Distance Telephone Company 16.45 120.55

2143 http://58.69.188.58:8181/WebService/srvcBranch.asmx?WSDL 58.69.188.58 58.69.188.58 Philippines 977648698 AS9299 Philippine Long Distance Telephone Company 16.45 120.55

2144 http://58.69.188.58:8181/WebService/srvcBandwidthReports.asmx?WSDL 58.69.188.58 58.69.188.58 Philippines 977648698 AS9299 Philippine Long Distance Telephone Company 16.45 120.55

2145 http://58.69.188.58:8181/WebService/srvcAuthentication.asmx?WSDL 58.69.188.58 58.69.188.58 Philippines 977648698 AS9299 Philippine Long Distance Telephone Company 16.45 120.55

2146 http://58.69.188.58:8181/WebService/srvcApplicationandUrlLookUp.asmx?WSDL 58.69.188.58 58.69.188.58 Philippines 977648698 AS9299 Philippine Long Distance Telephone Company 16.45 120.55

2147 http://58.69.188.58:8181/WebService/srvcApplicationUrlGroup.asmx?WSDL 58.69.188.58 58.69.188.58 Philippines 977648698 AS9299 Philippine Long Distance Telephone Company 16.45 120.55

2148 http://58.69.188.58:8181/WebService/srvcApplicationUrl.asmx?WSDL 58.69.188.58 58.69.188.58 Philippines 977648698 AS9299 Philippine Long Distance Telephone Company 16.45 120.55

2149 http://58.69.188.58:8181/WebService/srvcAppNotAllowedToKill.asmx?WSDL 58.69.188.58 58.69.188.58 Philippines 977648698 AS9299 Philippine Long Distance Telephone Company 16.45 120.55

2150 http://58.69.188.58:8181/WebService/srvcAgeGroups.asmx?WSDL 58.69.188.58 58.69.188.58 Philippines 977648698 AS9299 Philippine Long Distance Telephone Company 16.45 120.55

2151 http://58.69.188.58:8181/WebService/srvcAds.asmx?WSDL 58.69.188.58 58.69.188.58 Philippines 977648698 AS9299 Philippine Long Distance Telephone Company 16.45 120.55

2152 http://58.69.188.58:8181/WebService/srvcAccountType.asmx?WSDL 58.69.188.58 58.69.188.58 Philippines 977648698 AS9299 Philippine Long Distance Telephone Company 16.45 120.55

2153 http://58.69.188.58:8181/WebService/srvcAccountSegment.asmx?WSDL 58.69.188.58 58.69.188.58 Philippines 977648698 AS9299 Philippine Long Distance Telephone Company 16.45 120.55

2154 http://58.69.188.58:8181/WebService/srvcAccessRights.asmx?WSDL 58.69.188.58 58.69.188.58 Philippines 977648698 AS9299 Philippine Long Distance Telephone Company 16.45 120.55

2155 http://58.69.188.58:8181/WebService/svrcInventory.asmx?WSDL 58.69.188.58 58.69.188.58 Philippines 977648698 AS9299 Philippine Long Distance Telephone Company 16.45 120.55

2156 http://58.69.188.58:8181/WebService/srvcWorkstationConfigurationItems.asmx?WSDL 58.69.188.58 58.69.188.58 Philippines 977648698 AS9299 Philippine Long Distance Telephone Company 16.45 120.55

2157 http://58.69.188.58:8181/WebService/srvcWorkstationConfigurationGroupsLink.asmx?WSDL 58.69.188.58 58.69.188.58 Philippines 977648698 AS9299 Philippine Long Distance Telephone Company 16.45 120.55

2158 http://58.69.188.58:8181/WebService/srvcWorkstationConfigurationGroups.asmx?WSDL 58.69.188.58 58.69.188.58 Philippines 977648698 AS9299 Philippine Long Distance Telephone Company 16.45 120.55

2159 http://biomoby.org/services/wsdl/www.iris.irri.org/getStudies irri.org 23.23.183.47 Philippines 387430191 null 39.0437 -77.4875

2160 http://biomoby.org/services/wsdl/www.iris.irri.org/getAccessionByGermplasm irri.org 23.23.183.47 Philippines 387430191 null 39.0437 -77.4875

2161 http://biomoby.org/services/wsdl/www.iris.irri.org/getGermplasmByAccession irri.org 23.23.183.47 Philippines 387430191 null 39.0437 -77.4875

2162 http://biomoby.org/services/wsdl/www.iris.irri.org/getGermplasmDetails irri.org 23.23.183.47 Philippines 387430191 null 39.0437 -77.4875

2163 http://biomoby.org/services/wsdl/www.iris.irri.org/getGermplasmByName irri.org 23.23.183.47 Philippines 387430191 null 39.0437 -77.4875

2164 http://beta.transcendsystem.com/WSTranscend/WSTranscend.asmx?WSDL transcendsystem.com null Philippines 0 null null null

2165 http://www.partnerstwodlaprzyszlosci.edu.pl/_vti_bin/BusinessDataCatalog.asmx?wsdl partnerstwodlaprzyszlosci.edu.pl 64.4.6.100 Poland 1074005604 AS8075 Microsoft Corp 47.6801 -122.1206

2166 http://www.partnerstwodlaprzyszlosci.edu.pl/_vti_bin/People.asmx?wsdl partnerstwodlaprzyszlosci.edu.pl 65.55.39.10 Poland 1094133514 AS8075 Microsoft Corp 47.6801 -122.1206

2167 http://www.partnerstwodlaprzyszlosci.edu.pl/_vti_bin/Authentication.asmx?wsdl partnerstwodlaprzyszlosci.edu.pl 64.4.6.100 Poland 1074005604 AS8075 Microsoft Corp 47.6801 -122.1206

2168 http://dydaktyka.polsl.pl/_vti_bin/Lists.asmx?wsdl polsl.pl 157.158.143.1 Poland 2644414209 AS8508 SILWEB Autonomous System - Academic 52 20

2169 http://doktoranci.polsl.pl/_vti_bin/DWS.asmx?WSDL polsl.pl 157.158.3.2 Poland 2644378370 AS8508 SILWEB Autonomous System - Academic 52 20

2170 http://las.aei.polsl.pl/las2/las2.asmx?WSDL polsl.pl 157.158.3.1 Poland 2644378369 AS8508 SILWEB Autonomous System - Academic 52 20

2171 http://streemo.pl/Portal/WebServices/SongService.asmx?WSDL streemo.pl 91.199.48.136 Poland 1539780744 AS44249 COMTICA Sp. z o.o. 52 20

2172 http://streemo.pl/Portal/WebServices/ViewService.asmx?WSDL streemo.pl 91.199.48.136 Poland 1539780744 AS44249 COMTICA Sp. z o.o. 52 20

2173 http://streemo.pl/Portal/WebServices/WebmasterNotificationService.asmx?WSDL streemo.pl 91.199.48.136 Poland 1539780744 AS44249 COMTICA Sp. z o.o. 52 20

2174 http://streemo.pl/Portal/WebServices/SendToFriendService.asmx?WSDL streemo.pl 91.199.48.136 Poland 1539780744 AS44249 COMTICA Sp. z o.o. 52 20

2175 http://streemo.pl/Portal/WebServices/MediaRatingService.asmx?WSDL streemo.pl 91.199.48.136 Poland 1539780744 AS44249 COMTICA Sp. z o.o. 52 20

2176 http://streemo.pl/Portal/WebServices/InappropriateService.asmx?WSDL streemo.pl 91.199.48.136 Poland 1539780744 AS44249 COMTICA Sp. z o.o. 52 20

2177 http://streemo.pl/Portal/WebServices/UserCommentService.asmx?WSDL streemo.pl 91.199.48.136 Poland 1539780744 AS44249 COMTICA Sp. z o.o. 52 20

2178 http://streemo.pl/Portal/WebServices/CommentService.asmx?WSDL streemo.pl 91.199.48.136 Poland 1539780744 AS44249 COMTICA Sp. z o.o. 52 20

2179 http://streemo.pl/Portal/WebServices/PlayListService.asmx?WSDL streemo.pl 91.199.48.136 Poland 1539780744 AS44249 COMTICA Sp. z o.o. 52 20

2180 http://streemo.pl/Portal/WebServices/VoteService.asmx?WSDL streemo.pl 91.199.48.136 Poland 1539780744 AS44249 COMTICA Sp. z o.o. 52 20

2181 http://crossmatch.dnsalias.com/axis/services/Version?wsdl dnsalias.com 204.13.248.119 United States 3423467639 AS33517 Dynamic Network Services, Inc. 40.7904 -74.0246

2182 http://crossmatch.dnsalias.com/axis/services/SOAPMonitorService?wsdl dnsalias.com 204.13.248.119 United States 3423467639 AS33517 Dynamic Network Services, Inc. 40.7904 -74.0246

2183 http://serwis.sgh.waw.pl/test/wddataservice.php?wsdl sgh.waw.pl null Poland 0 null null null

2184 http://apps.ais.pl/fojet/services/FOProcessor?wsdl ais.pl 158.75.202.2 Poland 2655767042 AS35686 Torun Regional Computer Network 53.0358 18.6048

2185 http://www.cts.com.pl/webservices/rt_info.asmx?wsdl cts.com.pl 77.55.36.34 Poland 1295459362 AS15967 NetArt Spolka Akcyjna Spolka Komandytowo-Akcyjna 52 20

2186 http://www.dateblocker.com/Availability.asmx?WSDL dateblocker.com 77.79.220.20 Poland 1297079316 AS15694 ATMAN Autonomous System 52.25 21

2187 http://waluty.k2.pl/ws/NBPRates.asmx?WSDL k2.pl 193.200.67.208 Poland 3251127248 AS42503 K2 Internet S.A. 52 20

2188 http://www.awd.kolporter.com.pl/KOLEX2.php?wsdl kolporter.com.pl 91.197.153.24 Poland 1539676440 AS43638 Kolporter Info SA 50.8333 20.6667

2189 http://d.webnews.web2.wtc.pl/api/webnews.wsdl wtc.pl 62.146.30.96 Poland 1049763424 AS15598 IP Exchange GmbH 51 9

2190 http://www.iefp.pt/_vti_bin/BusinessDataCatalog.asmx?wsdl iefp.pt null Portugal 0 null null null

2191 http://www.iefp.pt/_vti_bin/Authentication.asmx?wsdl iefp.pt null Portugal 0 null null null

2192 http://www.iefp.pt/_vti_bin/People.asmx?wsdl iefp.pt null Portugal 0 null null null

2193 http://ws.keyfortravel.com/webservices/K4TAirSell.asmx?wsdl keyfortravel.com 82.102.4.79 Portugal 1382417487 AS25137 NFSi Telecom, Lda. 38.7167 -9.1333

2194 http://netservices.sapo.pt/adsl/adsl.asmx?wsdl sapo.pt 213.13.146.140 Portugal 3574436492 AS3243 PT Comunicacoes, S.A. 39.5 -8

2195 http://webservices.sapo.pt/auth/index.php?wsdl sapo.pt 213.13.146.140 Portugal 3574436492 AS3243 PT Comunicacoes, S.A. 39.5 -8

2196 http://www.7maravilhas.sapo.pt/ws/wsSeteMaravilhas.asmx?wsdl sapo.pt 213.13.146.140 Portugal 3574436492 AS3243 PT Comunicacoes, S.A. 39.5 -8

2197 http://118.sercultur.pt/ws.asmx?WSDL sercultur.pt 82.102.10.104 Portugal 1382419048 AS25137 NFSi Telecom, Lda. 39.5 -8

2198 http://ws.sercultur.pt/rss/ws.asmx?WSDL sercultur.pt 82.102.10.104 Portugal 1382419048 AS25137 NFSi Telecom, Lda. 39.5 -8

2199 http://ws.sercultur.pt/ws.asmx?wsdl sercultur.pt 82.102.10.104 Portugal 1382419048 AS25137 NFSi Telecom, Lda. 39.5 -8

2200 http://sws.sercultur.pt/ews.asmx?WSDL sercultur.pt 82.102.10.104 Portugal 1382419048 AS25137 NFSi Telecom, Lda. 39.5 -8

2201 http://netlab.ulusofona.pt/cd/praticas/ws/calcserver.php?wsdl ulusofona.pt null Portugal 0 null null null

2202 http://epl.di.uminho.pt/el07_g3/WebServices/server.php?wsdl uminho.pt null Portugal 0 null null null

2203 http://epl.di.uminho.pt/el07_g2/WebServices/server.php?wsdl uminho.pt null Portugal 0 null null null

2204 http://193.136.40.176/idmwebservice/IDMService.asmx?WSDL 193.136.40.176 193.136.40.176 Portugal 3246926000 AS1930 Rede Ciencia Tecnologia e Sociedade (RCTS) 41.3006 -7.7441

2205 http://194.8.30.116/gh/ConceptBEWS/Bookings.asmx?WSDL 194.8.30.116 194.8.30.116 Portugal 3255311988 AS33876 Flesk Lda 37.0194 -7.9322

2206 http://hm.comprafacil.pt/SIBSClickTeste/webservice/clicksmsV4.asmx?WSDL comprafacil.pt 195.22.4.13 Portugal 3272999949 AS8426 ClaraNET 38.7167 -9.1333

2207 http://www.esoperadores.com/reservassoap/devel/reservassoap.php?wsdl esoperadores.com null Portugal 0 null null null

2208 http://w2ks.dei.isep.ipp.pt/123456/ServicoDeDatas/Service1.asmx?wsdl ipp.pt 193.136.60.122 Portugal 3246931066 AS1930 Rede Ciencia Tecnologia e Sociedade (RCTS) 41.1496 -8.611

2209 http://misi.edu.pt/services/isis.asmx?WSDL misi.edu.pt 212.55.185.13 Portugal 3560421645 AS3243 PT Comunicacoes, S.A. 39.5 -8

2210 http://www.sporttv.pt/WSSporttv/WSSporttv.asmx?wsdl sporttv.pt 178.79.131.247 Portugal 2991555575 AS15830 TELECITYGROUP INTERNATIONAL LIMITED 51.5 -0.13

2211 http://www.uvtc.com/UVTCWebServices/swedishadsl.asmx?WSDL uvtc.com 87.236.91.27 Portugal 1475107611 AS35591 Provider-one.net Limited - www.provider-one.net 62 15

2212 http://www.salud.gov.pr/_vti_bin/BusinessDataCatalog.asmx?wsdl salud.gov.pr 136.145.14.10 Puerto Rico 2291207690 AS5786 University of Puerto Rico 18.25 -66.5

2213 http://www.salud.gov.pr/_vti_bin/People.asmx?wsdl salud.gov.pr 136.145.14.10 Puerto Rico 2291207690 AS5786 University of Puerto Rico 18.25 -66.5

2214 http://www.salud.gov.pr/_vti_bin/Authentication.asmx?wsdl salud.gov.pr 136.145.14.10 Puerto Rico 2291207690 AS5786 University of Puerto Rico 18.25 -66.5

2215 http://tech.tangunsoft.com/_vti_bin/BusinessDataCatalog.asmx?wsdl tangunsoft.com 112.220.30.11 Republic of Korea 1893473803 AS3786 LG DACOM Corporation 37.5985 126.9783

2216 http://acronis.tangunsoft.com/_vti_bin/People.asmx?wsdl tangunsoft.com 112.220.30.11 Republic of Korea 1893473803 AS3786 LG DACOM Corporation 37.5985 126.9783

2217 http://acronis.tangunsoft.com/_vti_bin/Authentication.asmx?wsdl tangunsoft.com 112.220.30.11 Republic of Korea 1893473803 AS3786 LG DACOM Corporation 37.5985 126.9783

2218 http://kiprict4.kipris.or.kr:7780/gateway/services/TMBibliographic?WSDL kipris.or.kr 152.99.204.81 Republic of Korea 2556677201 AS17841 MIC E-GOVERNMENT 36.3214 127.4197

2219 http://kiprict4.kipris.or.kr:7780/gateway/services/Statistic?WSDL kipris.or.kr 152.99.204.81 Republic of Korea 2556677201 AS17841 MIC E-GOVERNMENT 36.3214 127.4197

2220 http://kiprict4.kipris.or.kr:7780/gateway/services/SearchViennaCode?WSDL kipris.or.kr 152.99.204.81 Republic of Korea 2556677201 AS17841 MIC E-GOVERNMENT 36.3214 127.4197

2221 http://kiprict4.kipris.or.kr:7780/gateway/services/TMChangedInfo?WSDL kipris.or.kr 152.99.204.81 Republic of Korea 2556677201 AS17841 MIC E-GOVERNMENT 36.3214 127.4197

2222 http://kiprict4.kipris.or.kr:7780/gateway/services/TMImage?WSDL kipris.or.kr 152.99.204.81 Republic of Korea 2556677201 AS17841 MIC E-GOVERNMENT 36.3214 127.4197

2223 http://kiprict4.kipris.or.kr:7780/gateway/services/TMPublicationFullText?WSDL kipris.or.kr 152.99.204.81 Republic of Korea 2556677201 AS17841 MIC E-GOVERNMENT 36.3214 127.4197

2224 http://kiprict4.kipris.or.kr:7780/gateway/services/PopularKeyword?WSDL kipris.or.kr 152.99.204.81 Republic of Korea 2556677201 AS17841 MIC E-GOVERNMENT 36.3214 127.4197

2225 http://suggest.auction.co.kr/Suggest/SuggestWebService.asmx?WSDL auction.co.kr 211.233.17.11 Republic of Korea 3555266827 AS3786 LG DACOM Corporation 37.57 126.98

2226 http://www.inswave.com/ws/Admin?wsdl inswave.com 222.112.181.210 Republic of Korea 3731928530 AS4766 Korea Telecom 37.5985 126.9783

2227 http://inswave.com/ws/Tool?wsdl inswave.com 222.112.181.210 Republic of Korea 3731928530 AS4766 Korea Telecom 37.5985 126.9783

2228 http://www.inswave.com/ws/CCC?wsdl inswave.com 222.112.181.210 Republic of Korea 3731928530 AS4766 Korea Telecom 37.5985 126.9783

2229 http://webservice.youiwe.co.kr/SMS.v.2/ServiceSMS.asmx?WSDL youiwe.co.kr 218.38.19.213 Republic of Korea 3659928533 AS9318 Hanaro Telecom Inc. 37.57 126.98

2230 http://webservice.youiwe.co.kr/SMS.v.1/SMS_Process.asmx?WSDL youiwe.co.kr 218.38.19.213 Republic of Korea 3659928533 AS9318 Hanaro Telecom Inc. 37.57 126.98

2231 http://webservice.youiwe.co.kr/ZipCode.v.1/zipCode.asmx?WSDL youiwe.co.kr 218.38.19.213 Republic of Korea 3659928533 AS9318 Hanaro Telecom Inc. 37.57 126.98

2232 http://www.adecco.co.kr/WebService/CheckID.asmx?WSDL adecco.co.kr 61.109.248.170 Republic of Korea 1030617258 AS9848 Enterprise Networks 37.57 126.98

2233 http://www.adecco.co.kr/WebService/Vote.asmx?WSDL adecco.co.kr 61.109.248.170 Republic of Korea 1030617258 AS9848 Enterprise Networks 37.57 126.98

2234 http://www.cabal.co.kr/Helper/CabalClientHelper.asmx?WSDL cabal.co.kr 222.122.130.12 Republic of Korea 3732570636 AS4766 Korea Telecom 37.57 126.98

2235 http://webservice.direct.co.kr/post1.x/post.asmx?WSDL direct.co.kr 61.100.5.96 Republic of Korea 1029965152 AS9848 Enterprise Networks 37.57 126.98

2236 http://webservice.direct.co.kr/directsms1.x/directsms.asmx?WSDL direct.co.kr 61.100.5.96 Republic of Korea 1029965152 AS9848 Enterprise Networks 37.57 126.98

2237 http://websvc.dotnetpia.co.kr/DpNameCheck/DpNameCheck.asmx?WSDL dotnetpia.co.kr 121.189.62.216 Republic of Korea 2042445528 AS4766 Korea Telecom 37.57 126.98

2238 http://websvc.dotnetpia.co.kr/DpSms/DpSms.asmx?WSDL dotnetpia.co.kr 121.189.62.216 Republic of Korea 2042445528 AS4766 Korea Telecom 37.57 126.98

2239 http://interviewer.inha.ac.kr/stpWebServer/pathwayDBSer.asmx?WSDL inha.ac.kr 165.246.10.210 Republic of Korea 2784365266 AS9317 Inha University 37.57 126.98

2240 http://websvc.nesolution.com/DpSMS/DpSMS.asmx?WSDL nesolution.com 121.189.62.216 Republic of Korea 2042445528 AS4766 Korea Telecom 37.57 126.98

2241 http://soap.siriussoft.co.kr/En/PWS/WebService.asmx?WSDL siriussoft.co.kr 112.166.138.37 Republic of Korea 1889962533 AS4766 Korea Telecom 37.57 126.98

2242 http://www.bmskorea.co.kr/WebService/MemberJoin.asmx?WSDL bmskorea.co.kr 218.38.12.171 Republic of Korea 3659926699 AS9318 Hanaro Telecom Inc. 37.57 126.98

2243 http://www.devpia.com/nsoftware/Mail/Event/EventMail/mail_action.asmx?WSDL devpia.com 221.143.40.5 Australia 3717146629 AS9318 Hanaro Telecom Inc. 38.1833 127.8167

2244 http://dstts.co.kr/product/WebService.asmx?WSDL dstts.co.kr 210.121.204.3 Republic of Korea 3531197443 AS4766 Korea Telecom 37.57 126.98

2245 http://www.golibrary.go.kr/AutoCompleteWebService.asmx?WSDL golibrary.go.kr 211.181.46.47 Republic of Korea 3551866415 AS3786 LG DACOM Corporation 37.57 126.98

2246 http://isboard.net/ServiceMemberCheck.asmx?WSDL isboard.net 174.37.175.246 Republic of Korea 2921705462 AS36351 SoftLayer Technologies Inc. 47.6062 -122.3321

2247 http://version.neboard.co.kr/vercheck.asmx?WSDL neboard.co.kr 115.71.239.211 Republic of Korea 1934094291 null 36.724 126.8082

2248 http://www.samsung-emp.com/emp/services/RMSService?wsdl samsung-emp.com null Republic of Korea 0 null null null

2249 http://webservice.tongkni.co.kr/sms.1/ServiceSMS.asmx?WSDL tongkni.co.kr 218.38.12.183 Republic of Korea 3659926711 AS9318 Hanaro Telecom Inc. 37.57 126.98

2250 http://www.tarya.co.uk/ws/RSAkeygen.asmx?WSDL tarya.co.uk 89.38.214.108 Romania 1495717484 AS41635 LIFE SOFT SRL 45.7494 21.2272

2251 http://www.tarya.co.uk/ws/HashValues.asmx?WSDL tarya.co.uk 89.38.214.108 Romania 1495717484 AS41635 LIFE SOFT SRL 45.7494 21.2272

2252 http://www.tarya.co.uk/ws/Ip2countryws.asmx?WSDL tarya.co.uk 89.38.214.108 Romania 1495717484 AS41635 LIFE SOFT SRL 45.7494 21.2272

2253 http://www.archeus.ro/lingvistica/services/Lemmatizer?wsdl archeus.ro 216.154.210.119 United States 3634025079 AS20141 Quality Technology Services, LLC. 33.9681 -84.2304

2254 http://www.archeus.ro/lingvistica/services/Inflector?wsdl archeus.ro 216.154.210.119 United States 3634025079 AS20141 Quality Technology Services, LLC. 33.9681 -84.2304

2255 http://www.rasd.ro/BSEFinancialsWS/financials.asmx?WSDL rasd.ro null Romania 0 null null null

2256 http://195.28.176.87/BSEDelayedWs/Intraday.asmx?WSDL 195.28.176.87 195.28.176.87 Romania 3273437271 AS33821 Bursa de Valori Bucuresti SA 46 25

2257 http://www.agermag.ro/service.asmx?WSDL agermag.ro null Romania 0 null null null

2258 http://bvb.ro/BSEDelayedWs/Intraday.asmx?wsdl bvb.ro 195.28.176.87 Romania 3273437271 AS33821 Bursa de Valori Bucuresti SA 46 25

2259 http://focus.sai.msu.ru:8080/axis/services/Version?wsdl msu.ru 93.180.0.18 Russian Federation 1572077586 null 55.7522 37.6156

2260 http://solar.viniti.ru:8080/ScsMetaService/TreeService.asmx?WSDL viniti.ru 194.226.39.46 Russian Federation 3269601070 AS3316 RELARN RELARN-MSK 55.7522 37.6156

2261 http://solar.viniti.ru:8080/ScsMetaService/TreeTypeService.asmx?WSDL viniti.ru 194.226.39.46 Russian Federation 3269601070 AS3316 RELARN RELARN-MSK 55.7522 37.6156

2262 http://solar.viniti.ru:8080/ScsMetaService/TreePropertyTypeService.asmx?WSDL viniti.ru 194.226.39.46 Russian Federation 3269601070 AS3316 RELARN RELARN-MSK 55.7522 37.6156

2263 http://solar.viniti.ru:8080/ScsMetaService/TreePropertyService.asmx?WSDL viniti.ru 194.226.39.46 Russian Federation 3269601070 AS3316 RELARN RELARN-MSK 55.7522 37.6156

2264 http://www.cbr.ru/DailyInfoWebServ/DailyInfo.asmx?WSDL cbr.ru 212.40.192.49 Russian Federation 3559440433 AS8904 Autonomous System 55.7522 37.6156

2265 http://www.cbr.ru/secinfo/secinfo.asmx?wsdl cbr.ru 212.40.192.49 Russian Federation 3559440433 AS8904 Autonomous System 55.7522 37.6156

2266 http://www.cbr.ru/CreditInfoWebServ/CreditOrgInfo.asmx?wsdl cbr.ru 212.40.192.49 Russian Federation 3559440433 AS8904 Autonomous System 55.7522 37.6156

2267 http://www.cbr.ru/CBRFQuery/query.asmx?WSDL cbr.ru 212.40.192.49 Russian Federation 3559440433 AS8904 Autonomous System 55.7522 37.6156

2268 http://www.cbr.ru/RegionWebServ/regional.asmx?WSDL cbr.ru 212.40.192.49 Russian Federation 3559440433 AS8904 Autonomous System 55.7522 37.6156

2269 http://www.radikal.ru/integrforum.asmx?WSDL radikal.ru 81.176.238.246 Russian Federation 1370550006 AS8342 OJSC RTComm.RU 60 100

2270 http://radikal.ru/ImgLinksService.asmx?WSDL radikal.ru 81.176.238.246 Russian Federation 1370550006 AS8342 OJSC RTComm.RU 60 100

2271 http://kbyte.ru/3w/WebServices/MIMEReader.asmx?WSDL kbyte.ru 93.95.103.211 Russian Federation 1566533587 AS48347 JSC MediaSoft Ekspert 55.7522 37.6156

2272 http://kbyte.ru/webservice/newforum.asmx?WSDL kbyte.ru 93.95.103.211 Russian Federation 1566533587 AS48347 JSC MediaSoft Ekspert 55.7522 37.6156

2273 http://kbyte.ru/_ajaxServices/mainKbyteDotRuServices/KbyteDotRu.asmx?WSDL kbyte.ru 93.95.103.211 Russian Federation 1566533587 AS48347 JSC MediaSoft Ekspert 55.7522 37.6156

2274 http://www.mian.ru/WebServices/CartService.asmx?WSDL mian.ru 91.103.107.206 Russian Federation 1533504462 AS42032 ZAO Telecom 24 55.7522 37.6156

2275 http://office2003research.translate.ru/ResearchService/ITranslateSvc.asmx?wsdl translate.ru 62.152.52.123 Russian Federation 1050162299 AS29076 Citytelecom.ru 60 100

2276 http://office2003research.translate.ru/ResearchService/TranslateSvc.asmx?wsdl translate.ru 62.152.52.123 Russian Federation 1050162299 AS29076 Citytelecom.ru 60 100

2277 http://webservices.aeroflot.ru/flightSearch.wsdl aeroflot.ru 213.219.244.91 Russian Federation 3587961947 AS12695 Digital Network JSC 60 100

2278 http://webservices.aeroflot.ru/flightstatus.wsdl aeroflot.ru 89.208.149.200 Russian Federation 1506842056 AS12695 Digital Network JSC 60 100

2279 http://www.allysoft.ru/XML/Connector.asmx?WSDL allysoft.ru 87.236.31.154 Russian Federation 1475092378 AS12714 Fairlie Holding & Finance Limited 55.7522 37.6156

2280 http://allysoft.ru/BScurrency/currency.asmx?WSDL allysoft.ru 87.236.31.154 Russian Federation 1475092378 AS12714 Fairlie Holding & Finance Limited 55.7522 37.6156

2281 http://www.ozon.ru/webservices/OzonWebSvcFIP.asmx?WSDL ozon.ru 194.186.179.68 Russian Federation 3267015492 AS44386 LLC Internet Solutions 55.7522 37.6156

2282 http://www.ozon.ru/webservices/OzonWebSvc.asmx?WSDL ozon.ru 194.186.179.68 Russian Federation 3267015492 AS44386 LLC Internet Solutions 55.7522 37.6156

2283 http://activation1.abbyy.ru/productactivation.asmx?WSDL abbyy.ru 62.213.108.134 Russian Federation 1054174342 AS15756 CJSC Caravan-Telecom 60 100

2284 http://www.agrobase.ru/ActivationService.asmx?WSDL agrobase.ru 81.177.160.59 Russian Federation 1370595387 AS8342 OJSC RTComm.RU 55.7522 37.6156

2285 http://roomfinder.aparti.ru/RoomFinder.asmx?WSDL aparti.ru 77.222.61.227 Russian Federation 1306410467 AS44112 SpaceWeb JSC 60 100

2286 http://typograf.artlebedev.ru/webservices/typograf.asmx artlebedev.ru 195.218.200.11 Russian Federation 3285895179 AS3216 EDN Sovintel LLC 60 100

2287 http://vo.astronet.ru/ws/wcsfix/wcsfix.wsdl astronet.ru 93.180.27.7 Russian Federation 1572084487 null 55.7522 37.6156

2288 http://www.ataev.net/He/WebService1.asmx?WSDL ataev.net 195.128.123.149 Russian Federation 3279977365 AS5537 Garant-Park-Telecom ISP network, 60 100

2289 http://www.beesoft.ru/BScurrency/currency.asmx?WSDL beesoft.ru 82.98.86.167 Russian Federation 1382176423 AS12306 Plus.Line AG 51 9

2290 http://ims.cross-edu.ru/Services.asmx?WSDL cross-edu.ru null Russian Federation 0 null null null

2291 http://ws.darkagesworld.com/info.asmx?WSDL darkagesworld.com 89.208.140.32 Russian Federation 1506839584 AS12695 Digital Network JSC 60 100

2292 http://halton-climate.ru/catalog.asmx?WSDL halton-climate.ru 81.177.24.104 Russian Federation 1370560616 AS8342 OJSC RTComm.RU 60 100

2293 http://www.jdesign.ru/server.php?wsdl jdesign.ru 62.152.34.48 Russian Federation 1050157616 AS29076 Citytelecom.ru 60 100

2294 http://www.monitel.ru/WS/MonitelWebServices.asmx?WSDL monitel.ru 195.208.1.118 Russian Federation 3285188982 AS25535 Autonomous Non-commercial Organization 'Regional Network Information Center' 60 100

2295 http://integration.novelti.ru/IntegrationService.asmx?WSDL novelti.ru 195.128.121.12 Russian Federation 3279976716 AS5537 Garant-Park-Telecom ISP network, 60 100

2296 http://sg.n-sms.uz/flashgateway.asmx?WSDL n-sms.uz 89.236.220.2 Russian Federation 1508695042 AS34718 ISP TPS - BGP ASN of TEXNOPROSISTEM Ltd 41.3167 69.25

2297 http://www.otpravka.ru/WEBServices/WSRequests.asmx?WSDL otpravka.ru null Russian Federation 0 null null null

2298 http://perevozki.ru/WEBServices/WSRequests.asmx?WSDL perevozki.ru 62.213.111.241 Russian Federation 1054175217 AS15756 CJSC Caravan-Telecom 60 100

2299 http://www.ras.ru/RSS_upload.asmx?WSDL ras.ru 83.149.246.161 Russian Federation 1402336929 AS3058 Joint SuperComputer Center of the Russian Academy of Sciences 60 100

2300 http://ws.dice.spb.ru/default.asmx?WSDL spb.ru 212.193.242.155 Russian Federation 3569480347 AS25535 Autonomous Non-commercial Organization 'Regional Network Information Center' 60 100

2301 http://www.volgainfo.ru/GAR4/GAR.asmx?WSDL volgainfo.ru 213.234.29.138 Russian Federation 3588889994 AS39707 OOO SvyazInform 48.7194 44.5018

2302 http://mts.webcrm.ru/ContentProvidersService?WSDL webcrm.ru 83.222.4.120 Russian Federation 1407059064 AS25532 .masterhost autonomous system 60 100

2303 http://zhd.ru/WEBServices/WSRequests.asmx?WSDL zhd.ru 62.213.111.241 Russian Federation 1054175217 AS15756 CJSC Caravan-Telecom 60 100

2304 http://www.ksu.edu.sa/_vti_bin/BusinessDataCatalog.asmx?wsdl ksu.edu.sa 212.57.194.200 Saudi Arabia 3560555208 AS50517 King Saud University 25 45

2305 http://www.ksu.edu.sa/_vti_bin/People.asmx?wsdl ksu.edu.sa 212.57.194.200 Saudi Arabia 3560555208 AS50517 King Saud University 25 45

2306 http://www.ksu.edu.sa/_vti_bin/Authentication.asmx?wsdl ksu.edu.sa 212.57.194.200 Saudi Arabia 3560555208 AS50517 King Saud University 25 45

2307 http://www.gaca.gov.sa/Common/WebService/GaCA.asmx?WSDL gaca.gov.sa 62.149.106.154 Saudi Arabia 1049979546 AS25019 Autonomus System Number for SaudiNet 21.5169 39.2192

2308 http://www.nesmahajj.com/ws/service1.asmx?wsdl nesmahajj.com 212.71.42.3 Saudi Arabia 3561433603 AS24731 Cyberia is an Internet Service provider in SA Serving 25 45

2309 http://e-belgrade.net/FilterService.asmx?WSDL e-belgrade.net 176.28.45.144 Serbia and Montenegro 2954636688 null 51.65 6.1833

2310 http://hipagservice.bisinter.net/HipagServices.asmx?WSDL bisinter.net 194.106.162.42 Serbia and Montenegro 3261768234 AS6700 BeotelNet-ISP d.o.o 44 21

2311 http://212.200.25.178:8200/BulkSMS/BulkSMS/Distributed.asmx?WSDL 212.200.25.178 212.200.25.178 Serbia and Montenegro 3569883570 AS8400 TELEKOM SRBIJA a.d. 44 21

2312 http://mape.b92.net/FilterService.asmx?WSDL b92.net 91.222.6.80 Serbia and Montenegro 1541277264 null 44 21

2313 http://avalon.bitsyu.net/GamaSMS/SMSService.asmx?WSDL bitsyu.net null Serbia and Montenegro 0 null null null

2314 http://www.profitim.com/profitim.asmx?WSDL profitim.com 217.26.208.146 Serbia and Montenegro 3642413202 AS8771 YUnet International d.o.o. 44.8186 20.4681

2315 http://www.psit.co.yu/PSITResearchServices/registration.asmx?WSDL psit.co.yu null Serbia and Montenegro 0 null null null

2316 http://whampoa-staging.muvee.com:8080/axis/EchoHeaders.jws?wsdl muvee.com 69.93.158.212 Singapore 1163763412 AS21844 ThePlanet.com Internet Services, Inc. 29.7523 -95.367

2317 http://whampoa-staging.muvee.com:8080/axis/services/Version?wsdl muvee.com 69.93.158.212 Singapore 1163763412 AS21844 ThePlanet.com Internet Services, Inc. 29.7523 -95.367

2318 http://www.softwaremaker.net/webservices/swm/wswhereamI/index.asmx?wsdl softwaremaker.net 223.25.232.76 Singapore 3743017036 null 1.3667 103.8

2319 http://www.softwaremaker.net/webservices/swm/Convertor/Convertor.asmx?wsdl softwaremaker.net 223.25.232.76 Singapore 3743017036 null 1.3667 103.8

2320 http://www.softwaremaker.net/webservices/swm/CustomSWMSMS/index.asmx?WSDL softwaremaker.net 223.25.232.76 Singapore 3743017036 null 1.3667 103.8

2321 http://phaam.panasonic.com.my/barcode/po.asmx?wsdl panasonic.com.my 202.68.210.54 Singapore 3393507894 AS24038 Fujitsu Asia Pte Ltd 1.3667 103.8

2322 http://phaam.panasonic.com.my/barcode/user.asmx?wsdl panasonic.com.my 202.68.210.54 Singapore 3393507894 AS24038 Fujitsu Asia Pte Ltd 1.3667 103.8

2323 http://webservices.wilsonyau.com/public/csCreditCardValidator.asmx?WSDL wilsonyau.com 203.117.178.26 Singapore 3413488154 AS4657 StarHub Internet Exchange 1.2931 103.8558

2324 http://webservices.wilsonyau.com/public/csNRICChecksum.asmx?WSDL wilsonyau.com 203.117.178.26 Singapore 3413488154 AS4657 StarHub Internet Exchange 1.2931 103.8558

2325 http://truenorth.hubb.com/truenorth.asmx?WSDL hubb.com 202.167.244.39 Singapore 3400004647 AS17819 Equinix Asia Pacific -27 133

2326 http://www.smsdome.com/portalvbvs/services/smsgateway.asmx?wsdl smsdome.com 202.157.168.87 Singapore 3399329879 AS9892 Webvisions Pte Ltd 1.3667 103.8

2327 http://www.spellodrome.com/wsHomePage.asmx?WSDL spellodrome.com 202.167.245.160 Singapore 3400005024 AS17819 Equinix Asia Pacific 1.3667 103.8

2328 http://spellodrome.com.au/wsHomePage.asmx?WSDL spellodrome.com.au 202.167.245.160 Singapore 3400005024 AS17819 Equinix Asia Pacific 1.3667 103.8

2329 http://www.spellodrome.co.nz/wsHomePage.asmx?WSDL spellodrome.co.nz 202.167.245.160 Singapore 3400005024 AS17819 Equinix Asia Pacific 1.3667 103.8

2330 http://results.worldmathsday.com/webservices/ResultService.asmx?WSDL worldmathsday.com 202.9.83.185 Singapore 3389608889 null -27 133

2331 http://titan.fri.uniza.sk/ObuConnectorWS/Service.asmx?WSDL uniza.sk null Slovakia 0 null null null

2332 http://webservices.gama-system.com/exchangerates.asmx?WSDL gama-system.com 89.212.80.193 Slovenia 1507086529 AS34779 AS set propagated by T-2, d.o.o. 46.2197 14.6094

2333 http://webservices.gama-system.com/stockquotes.asmx?WSDL gama-system.com 89.212.80.193 Slovenia 1507086529 AS34779 AS set propagated by T-2, d.o.o. 46.2197 14.6094

2334 http://webservices.gama-system.com/servertime.asmx?WSDL gama-system.com 89.212.80.193 Slovenia 1507086529 AS34779 AS set propagated by T-2, d.o.o. 46.2197 14.6094

2335 http://www.gama-system.com/webservices/searchnajdisi.asmx?wsdl gama-system.com 89.212.80.193 Slovenia 1507086529 AS34779 AS set propagated by T-2, d.o.o. 46.2197 14.6094

2336 http://webservices.gama-system.com/numberguess.asmx?WSDL gama-system.com 89.212.80.193 Slovenia 1507086529 AS34779 AS set propagated by T-2, d.o.o. 46.2197 14.6094

2337 http://webservices.gama-system.com/officeresearch.asmx?WSDL gama-system.com 89.212.80.193 Slovenia 1507086529 AS34779 AS set propagated by T-2, d.o.o. 46.2197 14.6094

2338 http://webservices.gama-system.com/pingpong.asmx?WSDL gama-system.com 89.212.80.193 Slovenia 1507086529 AS34779 AS set propagated by T-2, d.o.o. 46.2197 14.6094

2339 http://apl.ess.gov.si/eSvetovanje/Services/TrgDela/Statistika.asmx?WSDL gov.si null Slovenia 0 null null null

2340 http://apl.ess.gov.si/eSvetovanje/Services/TrgDela/PoklicIzobrazba.asmx?WSDL gov.si null Slovenia 0 null null null

2341 http://apl.ess.gov.si/eSvetovanje/Services/TrgDela/IzobrazbaPoklic.asmx?WSDL gov.si null Slovenia 0 null null null

2342 http://livescreen.mojdenar.com/servic/BorzavZivo.asmx?WSDL mojdenar.com 93.103.3.110 Slovenia 1567032174 AS34779 AS set propagated by T-2, d.o.o. 46 15

2343 http://tis.telekom.si/TisSearch.asmx?WSDL telekom.si 213.229.249.246 Slovenia 3588618742 AS29276 Mobitel d.d. 46 15

2344 http://tis.telekom.si/formfieldsautocompletionservice.asmx?WSDL telekom.si 213.229.249.246 Slovenia 3588618742 AS29276 Mobitel d.d. 46 15

2345 http://bizi.si/Services/RssService.asmx?WSDL bizi.si 213.250.3.243 Slovenia 3589932019 AS5603 Telekom Slovenije d.d. 46.0553 14.5144

2346 http://research.caspis.net/webservices/flightdetail.asmx?wsdl caspis.net 93.103.8.33 Slovenia 1567033377 AS34779 AS set propagated by T-2, d.o.o. 46 15

2347 http://alchemist.ijs.si/ASP.NETv1.1/DMozClassify/OntoService.asmx?WSDL ijs.si 193.2.4.17 Slovenia 3238134801 AS2107 ARNES 46.0553 14.5144

2348 http://ddv.inetis.com/Iskalnik.asmx?wsdl inetis.com 85.10.40.2 Slovenia 1426728962 AS8591 AMiS 46 15

2349 http://moj.siol.net/sporocanje/komunikator/_eyeballws/EB_FriendlyName.asmx?WSDL siol.net 89.143.249.230 Slovenia 1502607846 AS5603 Telekom Slovenije d.d. 46 15

2350 http://temperatura.ziri.info/soap.php?wsdl ziri.info null Slovenia 0 null null null

2351 http://www.informationworker.co.za/_vti_bin/BusinessDataCatalog.asmx?wsdl informationworker.co.za 196.30.133.97 South Africa 3290334561 AS16637 MTNNS-AS -26.2052 28.0498

2352 http://www.informationworker.co.za/_vti_bin/Authentication.asmx?wsdl informationworker.co.za 196.30.133.97 South Africa 3290334561 AS16637 MTNNS-AS -26.2052 28.0498

2353 http://www.informationworker.co.za/_vti_bin/People.asmx?wsdl informationworker.co.za 196.30.133.97 South Africa 3290334561 AS16637 MTNNS-AS -26.2052 28.0498

2354 http://interfaces.bercoexpress.co.za/BercoServices/PostalcodeService.asmx?wsdl=0 bercoexpress.co.za 196.41.208.236 South Africa 3291074796 AS11845 DPBOL -26.2052 28.0498

2355 http://oracle.clickatell.com/push/index.php?wsdl clickatell.com null South Africa 0 null null null

2356 http://www.smsworx.co.za/sendsms3.asmx?WSDL smsworx.co.za null United States 0 null null null

2357 http://dsl.smsworx.co.za/ConsoleServices.asmx?WSDL smsworx.co.za 84.22.178.191 South Africa 1410773695 AS20860 Iomart 51.5 -0.13

2358 http://wsp.24.com/messenger/jabber.asmx?WSDL 24.com 41.86.110.143 South Africa 693530255 null -29 24

2359 http://csg.dla.gov.za/esio/imageViewer.wsdl dla.gov.za null South Africa 0 null null null

2360 http://www.facilities.co.za/DMA/Services/SuburbService.asmx?WSDL facilities.co.za null South Africa 0 null null null

2361 http://www.infocountry.co.za/za/default/webservice/SMSService.asmx?WSDL infocountry.co.za 208.116.12.217 South Africa 3497266393 AS25653 FortressITX 40.8326 -74.1307

2362 http://www.netcash.co.za/netserv/ncUpload/service.asmx?WSDL netcash.co.za 41.0.18.194 South Africa 687870658 AS36994 Vodacom-VB -29 24

2363 http://b2b.pnp.co.za/pnpservices/services/EchoService?wsdl pnp.co.za 196.33.17.33 South Africa 3290501409 AS3741 IS -33.9258 18.4232

2364 http://www.spoc.co.za/Dossiertest/DossierWS.asmx?WSDL spoc.co.za 196.41.213.15 South Africa 3291075855 AS11845 DPBOL -26.1353 28.0784

2365 http://wiptreasury.co.za/Shares.asmx?WSDL wiptreasury.co.za 196.41.9.83 South Africa 3291023699 AS11845 DPBOL -26.215 28.2547

2366 http://biomoby.org/services/wsdl/genome.imim.es/fromMetaAlignmentsToTextScoreMatrix imim.es 193.144.6.167 Spain 3247441575 AS766 RedIRIS Autonomous System 41.3984 2.1741

2367 http://biomoby.org/services/wsdl/genome.imim.es/fromMetaAlignmentsToScoreMatrix imim.es 193.144.6.167 Spain 3247441575 AS766 RedIRIS Autonomous System 41.3984 2.1741

2368 http://biomoby.org/services/wsdl/mmb.pcb.ub.es/showPDBFromFasta ub.es 161.116.1.1 Spain 2708734209 AS13041 CESCA - Anella Cientifica RREN Autonomous System 41.3984 2.1741

2369 http://biomoby.org/services/wsdl/mmb.pcb.ub.es/show3DFeature ub.es 161.116.1.1 Spain 2708734209 AS13041 CESCA - Anella Cientifica RREN Autonomous System 41.3984 2.1741

2370 http://biomoby.org/services/wsdl/mmb.pcb.ub.es/parseFeatureAASequenceFromFSOLVText ub.es 161.116.1.1 Spain 2708734209 AS13041 CESCA - Anella Cientifica RREN Autonomous System 41.3984 2.1741

2371 http://biomoby.org/services/wsdl/mmb.pcb.ub.es/getPDBLigandStructure ub.es 161.116.1.1 Spain 2708734209 AS13041 CESCA - Anella Cientifica RREN Autonomous System 41.3984 2.1741

2372 http://biomoby.org/services/wsdl/mmb.pcb.ub.es/parsePropertySequenceFromPMUTText ub.es 161.116.1.1 Spain 2708734209 AS13041 CESCA - Anella Cientifica RREN Autonomous System 41.3984 2.1741

2373 http://biomoby.org/services/wsdl/mmb.pcb.ub.es/runProStar_GFF_FromFasta ub.es 161.116.1.1 Spain 2708734209 AS13041 CESCA - Anella Cientifica RREN Autonomous System 41.3984 2.1741

2374 http://biomoby.org/services/wsdl/mmb.pcb.ub.es/plotFeatureAASequence ub.es 161.116.1.1 Spain 2708734209 AS13041 CESCA - Anella Cientifica RREN Autonomous System 41.3984 2.1741

2375 http://biomoby.org/services/wsdl/mmb.pcb.ub.es/getPDBIdsFromLigandId ub.es 161.116.1.1 Spain 2708734209 AS13041 CESCA - Anella Cientifica RREN Autonomous System 41.3984 2.1741

2376 http://biomoby.org/services/wsdl/mmb.pcb.ub.es/parseAccessibilityFromPHDText ub.es 161.116.1.1 Spain 2708734209 AS13041 CESCA - Anella Cientifica RREN Autonomous System 41.3984 2.1741

2377 http://biomoby.org/services/wsdl/mmb.pcb.ub.es/runEmbossCpGreport_GFF_FromFasta ub.es 161.116.1.1 Spain 2708734209 AS13041 CESCA - Anella Cientifica RREN Autonomous System 41.3984 2.1741

2378 http://biomoby.org/services/wsdl/mmb.pcb.ub.es/runEmbossTFscan_GFF_FromFasta ub.es 161.116.1.1 Spain 2708734209 AS13041 CESCA - Anella Cientifica RREN Autonomous System 41.3984 2.1741

2379 http://biomoby.org/services/wsdl/mmb.pcb.ub.es/getActiveSiteInterfaceFromPDBId ub.es 161.116.1.1 Spain 2708734209 AS13041 CESCA - Anella Cientifica RREN Autonomous System 41.3984 2.1741

2380 http://biomoby.org/services/wsdl/mmb.pcb.ub.es/getPDBLigand ub.es 161.116.1.1 Spain 2708734209 AS13041 CESCA - Anella Cientifica RREN Autonomous System 41.3984 2.1741

2381 http://biomoby.org/services/wsdl/mmb.pcb.ub.es/getPDBFromTransfac ub.es 161.116.1.1 Spain 2708734209 AS13041 CESCA - Anella Cientifica RREN Autonomous System 41.3984 2.1741

2382 http://biomoby.org/services/wsdl/mmb.pcb.ub.es/showFSOLVonStructure ub.es 161.116.1.1 Spain 2708734209 AS13041 CESCA - Anella Cientifica RREN Autonomous System 41.3984 2.1741

2383 http://biomoby.org/services/wsdl/mmb.pcb.ub.es/getComponentsFromPDBId ub.es 161.116.1.1 Spain 2708734209 AS13041 CESCA - Anella Cientifica RREN Autonomous System 41.3984 2.1741

2384 http://biomoby.org/services/wsdl/mmb.pcb.ub.es/parseGFFFromFeatureAASequence ub.es 161.116.1.1 Spain 2708734209 AS13041 CESCA - Anella Cientifica RREN Autonomous System 41.3984 2.1741

2385 http://biomoby.org/services/wsdl/mmb.pcb.ub.es/parseSecondaryStructureFromPHDText ub.es 161.116.1.1 Spain 2708734209 AS13041 CESCA - Anella Cientifica RREN Autonomous System 41.3984 2.1741

2386 http://biomoby.org/services/wsdl/mmb.pcb.ub.es/getProtProtInterfaceFromPDBId ub.es 161.116.1.1 Spain 2708734209 AS13041 CESCA - Anella Cientifica RREN Autonomous System 41.3984 2.1741

2387 http://biomoby.org/services/wsdl/mmb.pcb.ub.es/runSimpleTfbs ub.es 161.116.1.1 Spain 2708734209 AS13041 CESCA - Anella Cientifica RREN Autonomous System 41.3984 2.1741

2388 http://biomoby.org/services/wsdl/mmb.pcb.ub.es/runBindProteins ub.es 161.116.1.1 Spain 2708734209 AS13041 CESCA - Anella Cientifica RREN Autonomous System 41.3984 2.1741

2389 http://biomoby.org/services/wsdl/mmb.pcb.ub.es/getLigandIdFromPDBId ub.es 161.116.1.1 Spain 2708734209 AS13041 CESCA - Anella Cientifica RREN Autonomous System 41.3984 2.1741

2390 http://biomoby.org/services/wsdl/mmb.pcb.ub.es/parsePropertySequenceFromFSOLVText ub.es 161.116.1.1 Spain 2708734209 AS13041 CESCA - Anella Cientifica RREN Autonomous System 41.3984 2.1741

2391 http://biomoby.org/services/wsdl/mmb.pcb.ub.es/showPMUTonStructure ub.es 161.116.1.1 Spain 2708734209 AS13041 CESCA - Anella Cientifica RREN Autonomous System 41.3984 2.1741

2392 http://blogs.solidq.com/_vti_bin/BusinessDataCatalog.asmx?wsdl solidq.com 62.22.64.134 Spain 1041645702 AS702 Verizon Business EMEA - Commercial IP service provider in Europe 40.3084 -3.706

2393 http://blogs.solidq.com/_vti_bin/SharepointEmailWS.asmx?wsdl solidq.com 62.22.64.134 Spain 1041645702 AS702 Verizon Business EMEA - Commercial IP service provider in Europe 40.3084 -3.706

2394 http://blogs.solidq.com/_vti_bin/People.asmx?wsdl solidq.com 62.22.64.134 Spain 1041645702 AS702 Verizon Business EMEA - Commercial IP service provider in Europe 40.3084 -3.706

2395 http://blogs.solidq.com/_vti_bin/Authentication.asmx?wsdl solidq.com 62.22.64.134 Spain 1041645702 AS702 Verizon Business EMEA - Commercial IP service provider in Europe 40.3084 -3.706

2396 http://www.sp.upcomillas.es/_vti_bin/BusinessDataCatalog.asmx?wsdl upcomillas.es 130.206.70.225 Spain 2194556641 AS766 RedIRIS Autonomous System 40.4086 -3.6922

2397 http://www.sp.upcomillas.es/_vti_bin/Authentication.asmx?wsdl upcomillas.es 130.206.70.225 Spain 2194556641 AS766 RedIRIS Autonomous System 40.4086 -3.6922

2398 http://www.sp.upcomillas.es/_vti_bin/People.asmx?wsdl upcomillas.es 130.206.70.225 Spain 2194556641 AS766 RedIRIS Autonomous System 40.4086 -3.6922

2399 http://pt.germobanco.eu/_vti_bin/Authentication.asmx?wsdl germobanco.eu null Spain 0 null null null

2400 http://pt.germobanco.eu/_vti_bin/People.asmx?wsdl germobanco.eu null Spain 0 null null null

2401 http://ubio.bioinfo.cnio.es/biotools/iHOP/wsdl/iHOP-FuncNet.wsdl cnio.es null Spain 0 null null null

2402 http://eriste.aragob.es/webServiceEtiquetas/etiquetas.asmx?WSDL aragob.es null Spain 0 null null null

2403 http://eriste.aragob.es/WebServiceMedioAmbiente/MedioAmbiente.asmx?WSDL aragob.es null Spain 0 null null null

2404 http://eriste.aragob.es/WebServiceCdeCalidad/CdeCalidad.asmx?WSDL aragob.es null Spain 0 null null null

2405 http://eriste.aragob.es/WebServiceEstadistica/WebServiceEstadistica.asmx?WSDL aragob.es null Spain 0 null null null

2406 http://www.geoservicios.com/V1.0/sv/sv.asmx?wsdl geoservicios.com null Spain 0 null null null

2407 http://www.geoservicios.com/V1.0/sn/sn.asmx?wsdl geoservicios.com null Spain 0 null null null

2408 http://www.geoservicios.com/V2.0/sr/sr.asmx?wsdl geoservicios.com null Spain 0 null null null

2409 http://www.geoservicios.com/V2.0/sh/sh.asmx?wsdl geoservicios.com null Spain 0 null null null

2410 http://sas-d.sermepa.es/TPV_PC/wsdl/SerClsPeticionSOAPRF.wsdl sermepa.es null Spain 0 null null null

2411 http://sas-d.sermepa.es/TPV_PC/wsdl/prueba2.wsdl sermepa.es null Spain 0 null null null

2412 http://sas-d.sermepa.es/TPV_PC/wsdl/SerClsEntradaTPVPCv6d0.wsdl sermepa.es null Spain 0 null null null

2413 http://sas-d.sermepa.es/TPV_PC/wsdl/SerClsConsultasSOAPv2d0.wsdl sermepa.es null Spain 0 null null null

2414 http://servicioweb.bdigrupodifusion.com/Difusion/DifusionBib.asmx?WSDL bdigrupodifusion.com 82.165.147.245 Spain 1386583029 AS8560 1&1 Internet AG 51 9

2415 http://servicioweb.bdigrupodifusion.com/Difusion/DifusionJur.asmx?wsdl bdigrupodifusion.com 82.165.147.245 Spain 1386583029 AS8560 1&1 Internet AG 51 9

2416 http://servicioweb.bdigrupodifusion.com/Difusion/DifusionLeg.asmx?WSDL bdigrupodifusion.com 82.165.147.245 Spain 1386583029 AS8560 1&1 Internet AG 51 9

2417 http://elguille.info/NET/WebServices/HolaMundoWebS.asmx?WSDL elguille.info 213.27.202.67 Spain 3575368259 AS8220 COLT Technology Services 40 -4

2418 http://in2test.lsi.uniovi.es/sqlmutationws/?WSDL uniovi.es 156.35.33.105 Spain 2619548009 AS766 RedIRIS Autonomous System 43.3632 -5.8531

2419 http://petra.euitio.uniovi.es/~labra/ws/suma.php?wsdl uniovi.es 156.35.33.105 Spain 2619548009 AS766 RedIRIS Autonomous System 43.3632 -5.8531

2420 http://petra.euitio.uniovi.es/~labra/ws/ingredientes.wsdl uniovi.es 156.35.33.105 Spain 2619548009 AS766 RedIRIS Autonomous System 43.3632 -5.8531

2421 http://webserv.pki.gva.es:8080/axis/services/serviciospki?wsdl gva.es 193.144.127.85 Spain 3247472469 AS766 RedIRIS Autonomous System 39.4667 -0.3667

2422 http://www.cma.gva.es/areas/calidad_ambiental/CTL/buscador_reccas/AutoCompleteService.asmx?WSDL gva.es 193.144.127.85 Spain 3247472469 AS766 RedIRIS Autonomous System 39.4667 -0.3667

2423 http://mundoprogramacion.com/NET/WebServices/Conversor.asmx?WSDL mundoprogramacion.com 213.27.202.39 Spain 3575368231 AS8220 COLT Technology Services 40 -4

2424 http://mundoprogramacion.com/NET/WebServices/HolaMundoWebS.asmx?wsdl mundoprogramacion.com 213.27.202.39 Spain 3575368231 AS8220 COLT Technology Services 40 -4

2425 http://194.140.11.91:21111/servicios_integracion/services/DServicios?wsdl 194.140.11.91 194.140.11.91 Spain 3263957851 AS3324 TECHNOLOGY SOLUTIONS, S.A. 40 -4

2426 http://212.95.222.53/centraliaxmlnuevo2/service.asmx?WSDL 212.95.222.53 212.95.222.53 Spain 3563052597 AS6739 Cableuropa - ONO 41.6552 -4.7237

2427 http://62.15.105.120/soa/colaboraciones/anuntis/offerservice.asmx?WSDL 62.15.105.120 62.15.105.120 Spain 1041197432 AS12715 Jazz Telecom S.A. 43.2198 -2.7332

2428 http://62.204.194.45:8080/fedoragsearch/services/FgsOperations?wsdl 62.204.194.45 62.204.194.45 Spain 1053606445 AS15630 UNED Autonomous System 40.4086 -3.6922

2429 http://82.223.168.52/service.asmx?WSDL 82.223.168.52 82.223.168.52 Spain 1390389300 AS20718 arsys.es 40 -4

2430 http://91.117.122.10/services/soap.wsdl.php 91.117.122.10 91.117.122.10 Spain 1534425610 AS12334 R Cable y Telecomunicaciones Galicia S.A. 42.2896 -8.1134

2431 http://guia.efactura.asimelec.es/ws/service.wsdl asimelec.es 212.115.97.134 Spain 3564331398 AS12845 AS InforNet Systems null null

2432 http://www.atsoftline.com/updatePDA/update.asmx?WSDL atsoftline.com 81.184.3.221 Spain 1371014109 AS6739 Cableuropa - ONO 41.3984 2.1741

2433 http://fullfilment.buscpersonip.com/fullfilment.asmx?wsdl buscpersonip.com 89.140.80.58 Spain 1502367802 AS16338 Cableuropa - ONO 40 -4

2434 http://www.comunishopping.com/CS/GestionPersonas.asmx?WSDL comunishopping.com null Spain 0 null null null

2435 http://marte.cindoc.csic.es/swpeticiones/serviciopeticiones.asmx?WSDL csic.es 161.111.10.242 Spain 2708409074 AS766 RedIRIS Autonomous System 40.4086 -3.6922

2436 http://sms.dcod.com.mx/dcodsms/smsdcodserver.php?wsdl dcod.com.mx null Spain 0 null null null

2437 http://www.diode.es/serviciolp/milista.asmx?WSDL diode.es 85.118.247.29 Spain 1433859869 AS35171 UNIWAY TECHNOLOGIES SL 40 -4

2438 http://teletipo.europapress.es/wsepNoticias2006/Noticias.asmx?WSDL europapress.es 212.170.237.10 Spain 3567971594 AS6813 Autonomous System 40.45 -3.6892

2439 http://competiciones.feb.es/ncadminws/ncadmin.asmx?WSDL feb.es 96.45.82.4 Spain 1613582852 null 38.9311 -77.3489

2440 http://tpv.hellehollis.com/service.asmx?WSDL hellehollis.com 62.81.188.210 Spain 1045544146 AS6739 Cableuropa - ONO 41.5646 0.5247

2441 http://www.infobustussam.com:9001/services/dinamica.asmx?WSDL infobustussam.com 94.198.88.158 Spain 1590057118 AS48221 EMPRESAS MUNICIPALES DE SEVILLA, AIE - AS 37.3824 -5.9761

2442 http://www.inkolan.com/inkolan/wsServicio.asmx?WSDL inkolan.com 212.55.8.49 Spain 3560376369 AS12338 Euskaltel S.A. 43.301 -2.878

2443 http://www.matiasmasso.es/WebServices/eShops.asmx?wsdl matiasmasso.es 176.28.109.26 Spain 2954652954 null 40 -4

2444 http://sms.mexhico.com/smsdcodserver.php?wsdl mexhico.com 134.0.11.141 Spain 2248149901 null 40 -4

2445 http://www.saniline.com/sespa/notifications.asmx?WSDL saniline.com 213.171.253.150 Spain 3584818582 AS16206 NEO-SKY 2002 Autonomous System 1 41.3984 2.1741

2446 http://www.seglan.com/OpenBankingGateway/GateWay.asmx?wsdl seglan.com 213.171.251.83 Spain 3584818003 AS16206 NEO-SKY 2002 Autonomous System 1 40.4554 -3.4697

2447 http://blog.todoexpertos.com/api/BlogImporter.asmx?WSDL todoexpertos.com 64.27.57.24 Spain 1075525912 AS30475 WeHostWebSites.com 39.7525 -104.9995

2448 http://tu.tv/servicioTUTV/servicioTUTV.phtml?wsdl tu.tv 208.51.7.90 United States 3493005146 AS3549 Global Crossing Ltd. 38.7942 -117.1173

2449 http://einfnt2.uoc.edu/Csf/SoapManager.asmx?wsdl uoc.edu null Spain 0 null null null

2450 http://www.upcplus.com/BackEnd/User/User.asmx?wsdl upcplus.com 213.229.186.216 Spain 3588602584 AS8220 COLT Technology Services 41.3984 2.1741

2451 http://panel.webactiva.com/soap/soap.Webactiva.php?wsdl webactiva.com 85.112.9.226 Spain 1433405922 AS23148 Terremark 40.4086 -3.6922

2452 http://www.webhotelplus.com/hotelmercado_ws/hotelmercado_ws.asmx?wsdl webhotelplus.com 37.59.44.102 Spain 624634982 null 48.86 2.35

2453 http://polfarsktbrod.polfarskt.se/_vti_bin/SharepointEmailWS.asmx?wsdl polfarskt.se null Sweden 0 null null null

2454 http://polfarsktbrod.polfarskt.se/_vti_bin/BusinessDataCatalog.asmx?wsdl polfarskt.se null Sweden 0 null null null

2455 http://polfarsktbrod.polfarskt.se/_vti_bin/People.asmx?wsdl polfarskt.se null Sweden 0 null null null

2456 http://polfarsktbrod.polfarskt.se/_vti_bin/Authentication.asmx?wsdl polfarskt.se null Sweden 0 null null null

2457 http://www.pfizer.co.uk/Media/_vti_bin/BusinessDataCatalog.asmx?wsdl pfizer.co.uk 165.160.13.20 Sweden 2778729748 AS19574 Corporation Service Company 39.7351 -75.6684

2458 http://www.pfizer.co.uk/Media/_vti_bin/People.asmx?wsdl pfizer.co.uk 165.160.15.20 Sweden 2778730260 AS19574 Corporation Service Company 39.7351 -75.6684

2459 http://www.pfizer.co.uk/Media/_vti_bin/Authentication.asmx?wsdl pfizer.co.uk 165.160.13.20 Sweden 2778729748 AS19574 Corporation Service Company 39.7351 -75.6684

2460 http://www.sharepointforum.com/_vti_bin/BusinessDataCatalog.asmx?wsdl sharepointforum.com 88.80.168.222 Sweden 1481681118 AS15830 TELECITYGROUP INTERNATIONAL LIMITED 62 15

2461 http://www.sharepointforum.com/_vti_bin/People.asmx?wsdl sharepointforum.com 88.80.168.222 Sweden 1481681118 AS15830 TELECITYGROUP INTERNATIONAL LIMITED 62 15

2462 http://www.sharepointforum.com/_vti_bin/Authentication.asmx?wsdl sharepointforum.com 88.80.168.222 Sweden 1481681118 AS15830 TELECITYGROUP INTERNATIONAL LIMITED 62 15

2463 http://www.sp.se/_vti_bin/SharepointEmailWS.asmx?wsdl sp.se 62.119.40.164 Sweden 1047996580 AS8434 Telenor Sweden 57.7167 12.9167

2464 http://www.sp.se/_vti_bin/BusinessDataCatalog.asmx?wsdl sp.se 62.119.40.164 Sweden 1047996580 AS8434 Telenor Sweden 57.7167 12.9167

2465 http://www.sp.se/_vti_bin/People.asmx?wsdl sp.se 62.119.40.164 Sweden 1047996580 AS8434 Telenor Sweden 57.7167 12.9167

2466 http://www.sp.se/_vti_bin/Authentication.asmx?wsdl sp.se 62.119.40.164 Sweden 1047996580 AS8434 Telenor Sweden 57.7167 12.9167

2467 http://chem-fade.ad.umu.se/intranet/service/itradet/_vti_bin/SharepointEmailWS.asmx?wsdl umu.se 130.239.8.30 Sweden 2196703262 AS2833 Umea Universitet 63.8333 20.25

2468 http://chem-fade.ad.umu.se/intranet/service/itradet/_vti_bin/People.asmx?wsdl umu.se 130.239.8.30 Sweden 2196703262 AS2833 Umea Universitet 63.8333 20.25

2469 http://chem-fade.ad.umu.se/intranet/service/itradet/_vti_bin/Authentication.asmx?wsdl umu.se 130.239.8.30 Sweden 2196703262 AS2833 Umea Universitet 63.8333 20.25

2470 http://www.kuriren.nu/arkivet/arkiv/ws/TipService.asmx?WSDL kuriren.nu 212.214.212.197 Sweden 3570848965 AS3292 TDC Data Networks 62 15

2471 http://www.kuriren.nu/rss/default/ws/LoginService.asmx?WSDL kuriren.nu 212.214.212.197 Sweden 3570848965 AS3292 TDC Data Networks 62 15

2472 http://www.kuriren.nu/rss/default/ws/PassWordReminder.asmx?WSDL kuriren.nu 212.214.212.197 Sweden 3570848965 AS3292 TDC Data Networks 62 15

2473 http://www.kuriren.nu/arkivet/arkiv/ws/RegistrationService.asmx?WSDL kuriren.nu 212.214.212.197 Sweden 3570848965 AS3292 TDC Data Networks 62 15

2474 http://www.kuriren.nu/familj/default/ws/FamService.asmx?WSDL kuriren.nu 212.214.212.197 Sweden 3570848965 AS3292 TDC Data Networks 62 15

2475 http://www.kuriren.nu/arkivet/arkiv/ws/ForumService.asmx?WSDL kuriren.nu 212.214.212.197 Sweden 3570848965 AS3292 TDC Data Networks 62 15

2476 http://www.kuriren.nu/rss/default/ws/ArtListService.asmx?WSDL kuriren.nu 212.214.212.197 Sweden 3570848965 AS3292 TDC Data Networks 62 15

2477 http://www.nsd.se/blogg/bloggare/ws/BlogService.asmx?WSDL nsd.se 212.214.212.204 Sweden 3570848972 AS3292 TDC Data Networks 62 15

2478 http://www.nsd.se/blogg/bloggare/ws/LoginService.asmx?WSDL nsd.se 212.214.212.204 Sweden 3570848972 AS3292 TDC Data Networks 62 15

2479 http://www.nsd.se/blogg/bloggare/ws/PasswordReminder.asmx?WSDL nsd.se 212.214.212.204 Sweden 3570848972 AS3292 TDC Data Networks 62 15

2480 http://www.nsd.se/noje/artikel/ws/ForumService.asmx?WSDL nsd.se 212.214.212.204 Sweden 3570848972 AS3292 TDC Data Networks 62 15

2481 http://www.nsd.se/tjanster/debatt/default/ws/ForumList.asmx?WSDL nsd.se 212.214.212.204 Sweden 3570848972 AS3292 TDC Data Networks 62 15

2482 http://www.nsd.se/noje/artikel/ws/TipService.asmx?WSDL nsd.se 212.214.212.204 Sweden 3570848972 AS3292 TDC Data Networks 62 15

2483 http://www.nsd.se/tjanster/abonnera/default/ws/FormGen.asmx?WSDL nsd.se 212.214.212.204 Sweden 3570848972 AS3292 TDC Data Networks 62 15

2484 http://helagotland.se/tv/default/ws/LoginService.asmx?WSDL helagotland.se 212.214.212.198 Sweden 3570848966 AS3292 TDC Data Networks 62 15

2485 http://ga.helagotland.se/blogg/bloggentry/ws/ForumService.asmx?WSDL helagotland.se 212.214.212.198 Sweden 3570848966 AS3292 TDC Data Networks 62 15

2486 http://helagotland.se/tv/default/ws/PassWordReminder.asmx?WSDL helagotland.se 212.214.212.198 Sweden 3570848966 AS3292 TDC Data Networks 62 15

2487 http://ga.helagotland.se/sport/default/ws/ArtListServiceS.asmx?WSDL helagotland.se 212.214.212.198 Sweden 3570848966 AS3292 TDC Data Networks 62 15

2488 http://helagotland.se/jobb/default/ws/ArtListService.asmx?WSDL helagotland.se 212.214.212.198 Sweden 3570848966 AS3292 TDC Data Networks 62 15

2489 http://mellbourn.com/WebServices/PasswordGeneratorWebService/PasswordGeneratorService.asmx?WSDL mellbourn.com 194.9.94.86 Sweden 3255393878 AS39570 Loopia AB 59.6167 16.55

2490 http://www.mellbourn.com/ASP.NET/PersonalExperiments/70-320/HelloWorldWebService/HelloWorldService.asmx?wsdl mellbourn.com 194.9.94.85 Sweden 3255393877 AS39570 Loopia AB 59.6167 16.55

2491 http://www.mellbourn.com/ASP.NET/PersonalExperiments/70-320/TestWebService/Service1.asmx?wsdl mellbourn.com 194.9.94.86 Sweden 3255393878 AS39570 Loopia AB 59.6167 16.55

2492 http://artedi.ebc.uu.se/course/temperatures.wsdl uu.se null Sweden 0 null null null

2493 http://artedi.ebc.uu.se/course/Demo.wsdl uu.se null Sweden 0 null null null

2494 http://svep.epc.ub.uu.se/ffr/ws/FFRWebService?wsdl uu.se null Sweden 0 null null null

2495 http://epc.volvopenta.se/www/robot/PckExportService.asmx?WSDL volvopenta.se null Sweden 0 null null null

2496 http://epc.volvopenta.se/www/robot/SnlService.asmx?wsdl volvopenta.se null Sweden 0 null null null

2497 http://epc.volvopenta.se/www/robot/RobotService.asmx?WSDL volvopenta.se null Sweden 0 null null null

2498 http://en.white.se/Services/tipafriend.asmx?WSDL white.se 31.222.190.175 Sweden 534691503 null 51.4 0.05

2499 http://en.white.se/Services/experience.asmx?WSDL white.se 62.119.134.0 Sweden 1048020480 AS8434 Telenor Sweden 59.3333 18.05

2500 http://en.white.se/Services/live.asmx?WSDL white.se 31.222.190.175 Sweden 534691503 null 51.4 0.05

2501 http://easy.apsis.se/anp.asmx?wsdl apsis.se 54.246.105.22 Sweden 922118422 null 53.3331 -6.2489

2502 http://statistik.apsis.se/ajax/ajaxws.asmx?WSDL apsis.se 54.246.105.22 United States 922118422 null 53.3331 -6.2489

2503 http://www.bradspel.net/company/webservices/companyService.asmx?WSDL bradspel.net 188.95.227.31 Sweden 3160400671 AS42695 City Network Hosting AB 62 15

2504 http://bradspel.net/dataService.asmx?WSDL bradspel.net 188.95.227.31 Sweden 3160400671 AS42695 City Network Hosting AB 62 15

2505 http://malgruppskarta.citymail.se/CityGisWeb/Services.asmx?WSDL citymail.se 193.15.235.67 Sweden 3239045955 AS1257 TELE2 59.3333 18.05

2506 http://utv1-srv.compose.se/test/webservi.nsf/getsubject?WSDL compose.se 193.188.248.11 Sweden 3250386955 AS3301 TeliaNet Sweden 59.2833 15.2167

2507 http://utv1-srv.compose.se/test/webservi.nsf/getPerson?WSDL compose.se 193.188.248.11 Sweden 3250386955 AS3301 TeliaNet Sweden 59.2833 15.2167

2508 http://www.silobreaker.com/Code/WidgetService.asmx?WSDL silobreaker.com 213.136.38.100 Sweden 3582469732 AS8473 Bahnhof AB 59.3333 18.05

2509 http://www.silobreaker.com/EntityAutoCompleteProxy.asmx?WSDL silobreaker.com 213.136.38.100 Sweden 3582469732 AS8473 Bahnhof AB 59.3333 18.05

2510 http://www.121.nu/onetoone/AutoCompleteOmrade.asmx?WSDL 121.nu 62.13.13.89 Sweden 1041042777 AS8434 Telenor Sweden 57.7833 14.1833

2511 http://www.adjanitor.com/WS/ADJanitor/MainService.asmx?WSDL adjanitor.com 63.251.171.81 Sweden 1073458001 AS14744 Internap Network Services Corporation 47.6062 -122.3321

2512 http://jpp.xml.amv.se/jpprequest/jpprequest.asmx?wsdl amv.se 192.121.232.102 Sweden 3229214822 AS8434 Telenor Sweden 62 15

2513 http://api.anp.se/anp.asmx?WSDL anp.se 88.131.62.197 Sweden 1484996293 AS3292 TDC Data Networks 62 15

2514 http://www.reflect.aprinova.se/RaticsServices.asmx?WSDL aprinova.se 83.168.204.247 Sweden 1403571447 AS35041 Crystone Autonomous Network Stockholm 62 15

2515 http://www.autogiro.se/WebService.asmx?WSDL autogiro.se 80.76.152.138 Sweden 1347197066 AS8523 Basefarm AB. Stockholm - Sweden 62 15

2516 http://bellevue.se/vbmf/badminton/tournaments.asmx?WSDL bellevue.se 213.136.33.75 Sweden 3582468427 AS8473 Bahnhof AB 62 15

2517 http://bowlstream.tv/Misc/DirectorySearch.asmx?WSDL bowlstream.tv 176.34.244.115 Sweden 2955080819 null 53 -8

2518 http://webservices.brightpoint.se/wsOrder/wsorder.asmx?WSDL brightpoint.se 217.16.194.192 Sweden 3641754304 AS15793 Donator main networks. Located in Gothenburg, Sweden 62 15

2519 http://www.certification.nu/ecomedia/certificateUploadService.asmx?WSDL certification.nu 93.158.121.79 Sweden 1570666831 AS39369 Phonera Networks AB 62 15

2520 http://www.epostservice.com/ws/api_10.asmx?wsdl epostservice.com 213.115.215.156 Sweden 3581138844 AS2119 T.net 62 15

2521 http://fly.flyprice.dk/AJAXWebService.asmx?WSDL flyprice.dk 85.235.250.10 Sweden 1441528330 AS9167 WEBPARTNER A/S is a Danish Internet Service Provider 55.6667 12.5833

2522 http://hmisoftwareupdate.com/web/BexFilePileAUT.nsf/installerupdateservice?WSDL hmisoftwareupdate.com 195.67.87.149 Sweden 3275970453 AS3301 TeliaNet Sweden 55.6 13

2523 http://www.hultsfred.se/OpenWebServices/TeleSearch.asmx?WSDL hultsfred.se 194.23.137.20 Sweden 3256322324 AS3301 TeliaNet Sweden 62 15

2524 http://www.ifknorrkoping.se/ws/RelatedArticles.asmx?WSDL ifknorrkoping.se 195.88.130.89 Sweden 3277357657 AS196704 Ventiro AB 58.6 16.1833

2525 http://www.jm.se/CarreraFramework/CarreraServices.asmx?WSDL jm.se 194.23.77.7 Sweden 3256306951 AS3301 TeliaNet Sweden 62 15

2526 http://www.lundachark.se/Register.asmx?wsdl lundachark.se 194.9.95.102 Sweden 3255394150 AS39570 Loopia AB 62 15

2527 http://www.novasoftware.se/schedulewebservice/service.asmx?WSDL novasoftware.se 212.181.17.82 Sweden 3568636242 AS3301 TeliaNet Sweden 62 15

2528 http://www.offentligajobb.se/jobadsws/jobpositionposting.asmx?WSDL offentligajobb.se 62.181.197.139 Sweden 1052099979 AS21195 DGC One AB Autonomous System 62 15

2529 http://satellite1.periscopeit.co.uk:81/satelliteservice.asmx?WSDL periscopeit.co.uk 109.228.16.24 Sweden 1843662872 AS15418 Fasthosts Internet Ltd. Gloucester, UK. 51.8333 -2.25

2530 http://www.proberza.com/datameal/ws2/index.php?wsdl proberza.com 194.9.94.7 Sweden 3255393799 AS39570 Loopia AB 59.6167 16.55

2531 http://www.pt-academy.se/ScriptLibrary/sip_service.asmx?WSDL pt-academy.se null Sweden 0 null null null

2532 http://www3.skolverket.se/snws/sn.asmx?wsdl skolverket.se null Sweden 0 null null null

2533 http://mail.thomson.nu/Services/svcAliasAdmin.asmx?WSDL thomson.nu 195.43.38.58 Sweden 3274384954 AS35041 Crystone Autonomous Network Stockholm 62 15

2534 http://taps.timecut.se/taps/tapsservice.asmx?WSDL timecut.se 217.70.32.136 Sweden 3645251720 AS41175 Internet Border Technolgies AB 59.3333 18.05

2535 http://portal.gr.ch/_vti_bin/BusinessDataCatalog.asmx?wsdl gr.ch null Switzerland 0 null null null

2536 http://portal.gr.ch/_vti_bin/People.asmx?wsdl gr.ch null Switzerland 0 null null null

2537 http://portal.gr.ch/_vti_bin/Authentication.asmx?wsdl gr.ch null Switzerland 0 null null null

2538 http://www.leuchterag.ch/_vti_bin/BusinessDataCatalog.asmx?wsdl leuchterag.ch 217.26.54.18 Switzerland 3642373650 AS29097 Hostpoint AG, Switzerland 47.3667 8.55

2539 http://www.leuchterag.ch/_vti_bin/Authentication.asmx?wsdl leuchterag.ch 217.26.54.18 Switzerland 3642373650 AS29097 Hostpoint AG, Switzerland 47.3667 8.55

2540 http://www.leuchterag.ch/_vti_bin/People.asmx?wsdl leuchterag.ch 217.26.54.18 Switzerland 3642373650 AS29097 Hostpoint AG, Switzerland 47.3667 8.55

2541 http://www.ofwi.ch/_vti_bin/People.asmx?wsdl ofwi.ch 212.47.171.102 Switzerland 3559893862 AS9100 Aspectra AG 47.4353 8.5652

2542 http://www.ofwi.ch/_vti_bin/Authentication.asmx?wsdl ofwi.ch 212.47.171.102 Switzerland 3559893862 AS9100 Aspectra AG 47.4353 8.5652

2543 http://tb.ohchr.org/_vti_bin/BusinessDataCatalog.asmx?wsdl ohchr.org 193.194.138.68 Switzerland 3250752068 AS8659 AS8659 UNICC 47 8

2544 http://tb.ohchr.org/_vti_bin/SharepointEmailWS.asmx?WSDL ohchr.org 193.194.138.68 Switzerland 3250752068 AS8659 AS8659 UNICC 47 8

2545 http://tb.ohchr.org/_vti_bin/Authentication.asmx?wsdl ohchr.org 193.194.138.68 Switzerland 3250752068 AS8659 AS8659 UNICC 47 8

2546 http://tb.ohchr.org/_vti_bin/People.asmx?wsdl ohchr.org 193.194.138.68 Switzerland 3250752068 AS8659 AS8659 UNICC 47 8

2547 http://www.snf.ch/D/Faq/_vti_bin/People.asmx?wsdl snf.ch null Switzerland 0 null null null

2548 http://sharepoint.bpa-solutions.net/_vti_bin/SharepointEmailWS.asmx?wsdl bpa-solutions.net 80.237.185.42 Switzerland 1357756714 AS20773 AS of Hosteurope Germany / Cologne 51.65 6.1833

2549 http://sharepoint.bpa-solutions.net/_vti_bin/Authentication.asmx?wsdl bpa-solutions.net 80.237.185.42 Switzerland 1357756714 AS20773 AS of Hosteurope Germany / Cologne 51.65 6.1833

2550 http://sharepoint.bpa-solutions.net/_vti_bin/People.asmx?wsdl bpa-solutions.net 80.237.185.42 Switzerland 1357756714 AS20773 AS of Hosteurope Germany / Cologne 51.65 6.1833

2551 http://eument-net.gendercampus.ch/_vti_bin/SharepointEmailWS.asmx?wsdl gendercampus.ch null Switzerland 0 null null null

2552 http://eument-net.gendercampus.ch/_vti_bin/People.asmx?wsdl gendercampus.ch null Switzerland 0 null null null

2553 http://eument-net.gendercampus.ch/_vti_bin/Authentication.asmx?wsdl gendercampus.ch null Switzerland 0 null null null

2554 http://www.migros.ch/_vti_bin/SharepointEmailWS.asmx?wsdl migros.ch 146.67.140.37 Switzerland 2453900325 AS8803 Migros-Genossenschafts-Bund 47.3667 8.55

2555 http://www.migros.ch/_vti_bin/Authentication.asmx?wsdl migros.ch 146.67.140.37 Switzerland 2453900325 AS8803 Migros-Genossenschafts-Bund 47.3667 8.55

2556 http://www.migros.ch/_vti_bin/People.asmx?wsdl migros.ch 146.67.140.37 Switzerland 2453900325 AS8803 Migros-Genossenschafts-Bund 47.3667 8.55

2557 http://webservice.genotec.ch/geo.asmx?WSDL genotec.ch 82.195.225.46 Switzerland 1388568878 AS16215 Genotec AG 47 8

2558 http://webservice.genotec.ch/genotec.asmx?WSDL genotec.ch 82.195.225.46 Switzerland 1388568878 AS16215 Genotec AG 47 8

2559 http://webservice.genotec.ch/conversion.asmx?WSDL genotec.ch 82.195.225.46 Switzerland 1388568878 AS16215 Genotec AG 47 8

2560 http://webservice.genotec.ch/network.asmx?WSDL genotec.ch 82.195.225.46 Switzerland 1388568878 AS16215 Genotec AG 47 8

2561 http://webservice.genotec.ch/security.asmx?WSDL genotec.ch 82.195.225.46 Switzerland 1388568878 AS16215 Genotec AG 47 8

2562 http://webservice.genotec.ch/sharepoint_sbg.asmx?WSDL genotec.ch 82.195.225.46 Switzerland 1388568878 AS16215 Genotec AG 47 8

2563 http://www.professional.ch/_vti_bin/Lists.asmx?wsdl professional.ch null Switzerland 0 null null null

2564 http://zefix.admin.ch/webservices/zefixSvc/ZefixSvc.asmx?WSDL admin.ch null Switzerland 0 null null null

2565 http://www.local24.de/Services/AdUpsellingKeywords.asmx?WSDL local24.de 194.126.145.182 Switzerland 3263074742 AS34960 Netcetera AG 47 8

2566 http://www.local24.de/Services/AutoCompleteWhere.asmx?WSDL local24.de 194.126.145.182 Switzerland 3263074742 AS34960 Netcetera AG 47 8

2567 http://www.local24.de/Services/AutoCompleteWhat.asmx?WSDL local24.de 194.126.145.182 Switzerland 3263074742 AS34960 Netcetera AG 47 8

2568 http://ws.easymonitoring.ch/client.asmx?WSDL easymonitoring.ch 91.135.71.2 Switzerland 1535592194 AS41913 Computerline, Schlierbach, Switzerland 47.1724 8.5174

2569 http://myhits.isb-sib.ch/doc/FuncNet.wsdl isb-sib.ch null Switzerland 0 null null null

2570 http://www.ausbildungsbeitraege.info/prognoswspb/stipendium.asmx?WSDL ausbildungsbeitraege.info 80.86.194.180 Switzerland 1347863220 AS1836 green.ch AG The Internet Company Autonomous System 47 8

2571 http://www.cdsoft.ch/SwissCities.asmx?wsdl cdsoft.ch 92.43.216.115 Switzerland 1546377331 AS25563 Webland AG, Autonomous System 47 8

2572 http://www.dci.ch/app1/Zitate.asmx?WSDL dci.ch 92.43.216.113 Switzerland 1546377329 AS25563 Webland AG, Autonomous System 47 8

2573 http://www.dc-informatik.ch/app1/Zitate.asmx?wsdl dc-informatik.ch 92.43.216.113 Switzerland 1546377329 AS25563 Webland AG, Autonomous System 47 8

2574 http://ws.exsila.com/WSMember.asmx?WSDL exsila.com 91.135.71.6 Switzerland 1535592198 AS41913 Computerline, Schlierbach, Switzerland 47.1724 8.5174

2575 http://www.faustball-ins.ch/FbinsService.asmx?WSDL faustball-ins.ch 213.160.60.250 Switzerland 3584048378 AS21232 GGA Maur, Binz, Switzerland 47.3393 8.6714

2576 http://dexws.immomarktschweiz.ch/advgisexport.asmx?WSDL immomarktschweiz.ch null Switzerland 0 null null null

2577 http://webservices.interhome.com/WebService.asmx?WSDL interhome.com 193.201.123.174 Switzerland 3251207086 AS3303 Swisscom (Switzerland) Ltd 47 8

2578 http://booking.swiss.com/web/ws/CugAjaxService.asmx?WSDL swiss.com 80.77.218.200 Switzerland 1347279560 AS8549 Lufthansa Systems 51 9

2579 http://ecodo.cdps.tpc.edu.tw/ecodo/Ctrl/EduDoc/EduDocService.asmx?WSDL tpc.edu.tw null Taiwan 0 null null null

2580 http://www.ecs.com.tw/ECSWebSite/WS/Support/clsCpuModelSearchWS.asmx?WSDL ecs.com.tw null Taiwan 0 null null null

2581 http://www.ecs.com.tw/ECSWebSite/WS/Support/clsFaqSearchWS.asmx?WSDL ecs.com.tw null Taiwan 0 null null null

2582 http://www.ecs.com.tw/ECSWebSite/WS/Media/clsAwardSearchWS.asmx?WSDL ecs.com.tw null Taiwan 0 null null null

2583 http://www.ecs.com.tw/ECSWebSite/WS/Support/clsCpuProcessorSearchWS.asmx?WSDL ecs.com.tw null Taiwan 0 null null null

2584 http://www.ecs.com.tw/ECSWebSite/WS/DownloadSearch/clsDownloadSearchWS.asmx?WSDL ecs.com.tw null Taiwan 0 null null null

2585 http://140.129.79.217/WS/GetNYMUEmployeeEMailWS.exe/wsdl/INYMUEmployeeEMail 140.129.79.217 140.129.79.217 Taiwan 2357284825 AS1659 Tiawan Academic Network (TANet) Information Center 25.0392 121.525

2586 http://e-trans.iot.gov.tw/FIDSWebS/services/FIDSWS?wsdl iot.gov.tw null Taiwan 0 null null null

2587 http://e-trans.iot.gov.tw/FIDSWebS/services/Version?wsdl iot.gov.tw null Taiwan 0 null null null

2588 http://ap.ydu.edu.tw/LDAP_WS/SN_CROSS.asmx?WSDL ydu.edu.tw null Taiwan 0 null null null

2589 http://ap.ydu.edu.tw/LDAP_WS/LDAP_AUT.asmx?WSDL ydu.edu.tw null Taiwan 0 null null null

2590 http://ap.ydu.edu.tw/LDAP_WS/Ret_Data_Service.asmx?WSDL ydu.edu.tw null Taiwan 0 null null null

2591 http://www.cp.gov.tw/SEWebApplication/AAMediator.asmx?WSDL cp.gov.tw null Taiwan 0 null null null

2592 http://siteserver.cybertutor.com.tw/Ast.Passport.Web/WebServices/Passport.asmx?WSDL cybertutor.com.tw null Taiwan 0 null null null

2593 http://www.csie.ntu.edu.tw/~r92106/hello.php?wsdl ntu.edu.tw null Taiwan 0 null null null

2594 http://www.advantech.co.jp/webservice/AdvantechWebServiceLocal.asmx?WSDL advantech.co.jp null United Kingdom 0 null null null

2595 http://www.advantech.com.my/webservice/AdvantechWebServiceLocal.asmx?WSDL advantech.com.my null Taiwan 0 null null null

2596 http://www.advantech.in/webservice/AdvantechWebServiceLocal.asmx?WSDL advantech.in 219.90.3.201 Taiwan 3680109513 AS703 MCI Communications Services, Inc. d/b/a Verizon Business 23.5 121

2597 http://www.advantechkorea.co.kr/webservice/AdvantechWebServiceLocal.asmx?WSDL advantechkorea.co.kr null Taiwan 0 null null null

2598 http://www.advantechsg.com.sg/webservice/AdvantechWebServiceLocal.asmx?WSDL advantechsg.com.sg null Taiwan 0 null null null

2599 http://webservice.anicebook.com.tw/epaper/paper.asmx?WSDL anicebook.com.tw 60.199.248.217 Taiwan 1019738329 AS9924 Taiwan Fixed Network, Telco and Network Service Provider. 25.0392 121.525

2600 http://ecpa.cpa.gov.tw/Ecpa/StudyAreaReceiver.asmx?WSDL cpa.gov.tw null Taiwan 0 null null null

2601 http://etradegroup.money888.com.tw/wsINTABF/processINTABF.asmx?WSDL money888.com.tw null Taiwan 0 null null null

2602 http://www.morgenstern.com.tw/TBPWin/WS_GetTbps.asmx?WSDL morgenstern.com.tw null Taiwan 0 null null null

2603 http://www.industry.go.th/_vti_bin/BusinessDataCatalog.asmx?wsdl industry.go.th 119.63.78.47 Thailand 2000637487 AS38794 BB-Broadband Co., Ltd. Transit AS 15 100

2604 http://www.industry.go.th/_vti_bin/People.asmx?wsdl industry.go.th 119.63.78.47 Thailand 2000637487 AS38794 BB-Broadband Co., Ltd. Transit AS 15 100

2605 http://monitoring.industry.go.th/tuscada/ClearService.asmx?WSDL industry.go.th 119.63.78.47 Thailand 2000637487 AS38794 BB-Broadband Co., Ltd. Transit AS 15 100

2606 http://www.industry.go.th/_vti_bin/Authentication.asmx?wsdl industry.go.th 119.63.78.47 Thailand 2000637487 AS38794 BB-Broadband Co., Ltd. Transit AS 15 100

2607 http://www.price.moc.go.th/webservices/BteiCpiService.asmx?WSDL moc.go.th 110.164.176.0 Thailand 1856286720 null 15 100

2608 http://www.price.moc.go.th/webservices/BteiCpiGService.asmx?WSDL moc.go.th 110.164.176.30 Thailand 1856286750 AS38550 TTGN , INTERNATIONAL INTERNET GATEWAY, THAILAND 15 100

2609 http://www.price.moc.go.th/webservices/BteiCmiService.asmx?WSDL moc.go.th 110.164.176.16 Thailand 1856286736 AS38550 TTGN , INTERNATIONAL INTERNET GATEWAY, THAILAND 15 100

2610 http://www.price.moc.go.th/webservices/BteiCpiUService.asmx?WSDL moc.go.th 110.164.176.0 Thailand 1856286720 null 15 100

2611 http://www.price.moc.go.th/webservices/BteiCpiLService.asmx?WSDL moc.go.th 110.164.176.30 Thailand 1856286750 AS38550 TTGN , INTERNATIONAL INTERNET GATEWAY, THAILAND 15 100

2612 http://www.price.moc.go.th/webservices/BteiPpiService.asmx?WSDL moc.go.th 110.164.176.16 Thailand 1856286736 AS38550 TTGN , INTERNATIONAL INTERNET GATEWAY, THAILAND 15 100

2613 http://www.price.moc.go.th/webservices/BteiCpiPService.asmx?WSDL moc.go.th 110.164.176.0 Thailand 1856286720 null 15 100

2614 http://app.nic.go.th/NICWebservices/CLS/CLSServices.asmx?WSDL nic.go.th null Thailand 0 null null null

2615 http://app.nic.go.th/NICWebservices/houseHoldServices/ICTHouseHold.asmx?WSDL nic.go.th null Thailand 0 null null null

2616 http://app.nic.go.th/NICWebservices/MDGs/MDGsServices.asmx?WSDL nic.go.th null Thailand 0 null null null

2617 http://app.nic.go.th/egmsProvider/egmsServices.asmx?WSDL nic.go.th null Thailand 0 null null null

2618 http://www.creditbangkok.com/WebService/CreditCardUtil.asmx?WSDL creditbangkok.com 202.142.221.148 Thailand 3398360468 AS7654 Internet Service Provider Co., Ltd. 15 100

2619 http://www.creditthai.net/WebService/CreditCardUtil.asmx?WSDL creditthai.net 202.142.221.148 Thailand 3398360468 AS7654 Internet Service Provider Co., Ltd. 15 100

2620 http://www.creditthai.net/WebService/CreditCardProductControl.asmx?WSDL creditthai.net 202.142.221.148 Thailand 3398360468 AS7654 Internet Service Provider Co., Ltd. 15 100

2621 http://smsgateway.sanook.com/Gatewayout/Dataservice.asmx?WSDL sanook.com 203.151.129.133 Thailand 3415703941 AS4618 Internet Thailand Company Limited 15 100

2622 http://wallet.sanook.com/sanookWebservice/walletProperty.asmx?wsdl sanook.com 203.151.130.199 Thailand 3415704263 AS4618 Internet Thailand Company Limited 15 100

2623 http://www.paysbuy.com/getinvoice/getinvoicestatus.asmx?WSDL paysbuy.com 61.90.197.234 Thailand 1029359082 AS9287 ASIA INFONET Co.,Ltd. 13.754 100.5014

2624 http://ws.contentlib.mweb.co.th/ContentLibrary.WS/WSMusic.asmx?wsdl mweb.co.th 203.151.133.199 Thailand 3415705031 AS4618 Internet Thailand Company Limited 15 100

2625 http://www.pttplc.com/pttinfo.asmx?wsdl pttplc.com 202.44.4.97 Thailand 3391882337 AS55403 PTTICT Solutions Co.,Ltd. 13.754 100.5014

2626 http://th.sbuypayment.com/webservices/Pay.php?wsdl sbuypayment.com 27.254.40.193 Thailand 469641409 AS4750 CS LOXINFO PUBLIC COMPANY LIMITED 15 100

2627 http://student.labs.ii.edu.mk/ii9263/slaveProject/Service1.asmx?WSDL ii.edu.mk 194.149.135.31 The Former Yugoslav Republic of Macedonia 3264579359 AS5379 MARNET-AS 41.8333 22

2628 http://www.nbrm.gov.mk/klservice/kurs.asmx?wsdl nbrm.gov.mk 91.226.21.195 The Former Yugoslav Republic of Macedonia 1541543363 null 41.8333 22

2629 http://ihracat.ssm.gov.tr/_vti_bin/Authentication.asmx?wsdl ssm.gov.tr null Turkey 0 null null null

2630 http://hastane.akdeniz.edu.tr/_vti_bin/Permissionswsdl.aspx akdeniz.edu.tr null Turkey 0 null null null

2631 http://hastane.akdeniz.edu.tr/_vti_bin/Meetingswsdl.aspx akdeniz.edu.tr null Turkey 0 null null null

2632 http://hastane.akdeniz.edu.tr/_vti_bin/Listswsdl.aspx akdeniz.edu.tr null Turkey 0 null null null

2633 http://hastane.akdeniz.edu.tr/_vti_bin/Imagingwsdl.aspx akdeniz.edu.tr null Turkey 0 null null null

2634 http://hastane.akdeniz.edu.tr/_vti_bin/Formswsdl.aspx akdeniz.edu.tr null Turkey 0 null null null

2635 http://hastane.akdeniz.edu.tr/_vti_bin/DspStswsdl.aspx akdeniz.edu.tr null Turkey 0 null null null

2636 http://hastane.akdeniz.edu.tr/_vti_bin/DspSqlwsdl.aspx akdeniz.edu.tr null Turkey 0 null null null

2637 http://hastane.akdeniz.edu.tr/_vti_bin/alertswsdl.aspx akdeniz.edu.tr null Turkey 0 null null null

2638 http://hastane.akdeniz.edu.tr/_vti_bin/Webswsdl.aspx akdeniz.edu.tr null Turkey 0 null null null

2639 http://hastane.akdeniz.edu.tr/_vti_bin/webpartpageswsdl.aspx akdeniz.edu.tr null Turkey 0 null null null

2640 http://hastane.akdeniz.edu.tr/_vti_bin/versionswsdl.aspx akdeniz.edu.tr null Turkey 0 null null null

2641 http://hastane.akdeniz.edu.tr/_vti_bin/UserGroupwsdl.aspx akdeniz.edu.tr null Turkey 0 null null null

2642 http://hastane.akdeniz.edu.tr/_vti_bin/DWS.asmx?WSDL akdeniz.edu.tr null Turkey 0 null null null

2643 http://portal.huap.org/_vti_bin/Lists.asmx?wsdl huap.org 208.87.149.250 Turkey 3495400954 AS40634 FirstLook, Inc. 33.9164 -118.4041

2644 http://www.tjk.org/WebService/Sabitler.asmx?wsdl tjk.org 195.200.222.241 Turkey 3284721393 AS41417 Turkiye Jokey Kulubu 41.0186 28.9647

2645 http://sgs.turkcell.com.tr/SendSMS/SendSMS?WSDL turkcell.com.tr 86.108.161.36 Turkey 1449959716 AS16135 Turkcell A.S. 39 35

2646 http://sgs.turkcell.com.tr/Register/Register?WSDL turkcell.com.tr 86.108.161.36 Turkey 1449959716 AS16135 Turkcell A.S. 39 35

2647 http://sgs.turkcell.com.tr/GetStatus/GetStatus?WSDL turkcell.com.tr 86.108.161.36 Turkey 1449959716 AS16135 Turkcell A.S. 39 35

2648 http://213.243.33.26:8888/ivrpool/ivr.asmx?WSDL 213.243.33.26 213.243.33.26 Turkey 3589480730 AS12978 Dogan Iletisim Elektronik Servis Hizmetleri ve Yayincilik Anonim Sirketi 37.7528 42.798

2649 http://213.243.33.26:8888/ivrpool/ivr_get.asmx?WSDL 213.243.33.26 213.243.33.26 Turkey 3589480730 AS12978 Dogan Iletisim Elektronik Servis Hizmetleri ve Yayincilik Anonim Sirketi 37.7528 42.798

2650 http://sbu2.saglik.gov.tr/mkys/mkysServis.asmx?WSDL saglik.gov.tr 212.175.169.156 Turkey 3568282012 AS9121 Turk Telekomunikasyon Anonim Sirketi 39 35

2651 http://www.takiponline.com/takiponline.webservice/Service.asmx?WSDL takiponline.com null Turkey 0 null null null

2652 http://www.takiponline.com/WebVersionService.asmx?WSDL takiponline.com null Turkey 0 null null null

2653 http://bitest.tarsim.org.tr:7777/TARSIM/InfoTarsimV6.jws?wsdl tarsim.org.tr null Turkey 0 null null null

2654 http://bitest.tarsim.org.tr:7777/TARSIM/InfoTahsilat.jws?wsdl tarsim.org.tr null Turkey 0 null null null

2655 http://service.bigpara.com/Views/Public/BPDataWebServices.asmx?wsdl bigpara.com 83.66.129.113 Turkey 1396867441 AS12978 Dogan Iletisim Elektronik Servis Hizmetleri ve Yayincilik Anonim Sirketi 37.7528 42.798

2656 http://www.dogus.k12.tr/duwebservices/service1.asmx?WSDL dogus.k12.tr 89.106.5.131 Turkey 1500120451 AS39582 Grid Bilisim Teknolojileri A.S. 41.0186 28.9647

2657 http://www.emecmua.com/nreader/Service1.asmx?WSDL emecmua.com 213.243.39.242 Turkey 3589482482 AS12978 Dogan Iletisim Elektronik Servis Hizmetleri ve Yayincilik Anonim Sirketi 37.7528 42.798

2658 http://emek.emekli.gov.tr/emekws/services/EmekNet/wsdl/EmekNet.wsdl emekli.gov.tr null Turkey 0 null null null

2659 http://www.maradit.net/kcm/service.asmx?WSDL maradit.net 94.73.138.103 Turkey 1581877863 AS34619 Cizgi Telekomunikasyon Hizmetleri Sanayi Ve Ticaret Limited Sirketi 36.9031 30.6991

2660 http://blog.milliyet.com.tr/blogkategorigetir.asmx?WSDL milliyet.com.tr 85.111.27.167 Turkey 1433344935 AS9121 Turk Telekomunikasyon Anonim Sirketi 39 35

2661 http://chat.siberalem.com/SMS.asmx?wsdl siberalem.com 212.154.28.52 Turkey 3566869556 AS12735 TurkNet Iletisim Hizmetleri A.S 39 35

2662 http://www.smsexplorer.com/webservices/city.asmx?WSDL smsexplorer.com 94.73.138.100 Turkey 1581877860 AS34619 Cizgi Telekomunikasyon Hizmetleri Sanayi Ve Ticaret Limited Sirketi 36.9031 30.6991

2663 http://www.tgd.com.tr/ws/get_price.php?wsdl tgd.com.tr 213.155.100.119 Turkey 3583730807 AS8685 Doruk Iletisim ve Otomasyon Sanayi ve Ticaret A.S. 37.7035 40.7921

2664 http://uyg.jandarma.tsk.mil.tr/kbs_tesis/webservis/kbsup.asmx?WSDL tsk.mil.tr null Turkey 0 null null null

2665 http://www.ucyildizmakina.com.tr/UrunStok.asmx?WSDL ucyildizmakina.com.tr 213.142.137.19 Turkey 3582888211 AS16265 LEASEWEB LEASEWEB AS 39 35

2666 http://www.turne.com.ua/WebServices/TourService.asmx?wsdl turne.com.ua 213.169.64.180 Ukraine 3584639156 AS13121 Navigator Online TOV 50.4333 30.5167

2667 http://www.turne.com.ua/WebServices/CountryResort.asmx?WSDL turne.com.ua 213.169.64.180 Ukraine 3584639156 AS13121 Navigator Online TOV 50.4333 30.5167
[truncated: 475,203 more chars]
